# Item response theory and differential test functioning analysis of the HBSC-Symptom-Checklist across 46 countries

**DOI:** 10.1186/s12874-022-01698-3

**Published:** 2022-09-29

**Authors:** Andreas Heinz, Philipp E. Sischka, Carolina Catunda, Alina Cosma, Irene García-Moya, Nelli Lyyra, Anne Kaman, Ulrike Ravens-Sieberer, William Pickett

**Affiliations:** 1 Department of Social Sciences, University of Luxembourg, Maison des Sciences Humaines, 11, Porte des Sciences, L-4366 Esch-sur-Alzette, Luxembourg; 2 Department of Health, IU Internationale Hochschule, Erfurt, Germany; 3 Department of Behavioural and Cognitive Sciences, University of Luxembourg, Esch-sur-Alzette, Luxembourg; 4 Sts Cyril and Methodius Faculty of Theology, Olomouc University Social Health Institute, Palacky University in Olomouc, Olomouc, Czech Republic; 5 Department of Sociology, Trinity College Dublin, Dublin, Ireland; 6 Department of Developmental and Educational Psychology, Universidad de Sevilla, Seville, Spain; 7 Faculty of Sport and Health Sciences, University of Jyväskylä, Jyväskylä, Finland; 8 Department of Child and Adolescent Psychiatry, Psychotherapy, and Psychosomatics, University Medical Center Hamburg-Eppendorf, Hamburg, Germany; 9 Department of Health Sciences, Brock University, St Catharines, Canada; 10 Department of Public Health Sciences, Queen’s University, Kingston, Canada

**Keywords:** Differential item functioning, Health behaviour in school-aged children, Psychosomatic health complaints, Measurement invariance, Self-reported health complaints, HBSC symptom checklist, Subjective health complaints, Cross-national, Adolescents

## Abstract

**Background:**

The Symptom Checklist (SCL) developed by the Health Behaviour in School-aged Children (HBSC) study is a non-clinical measure of psychosomatic complaints (e.g., headache and feeling low) that has been used in numerous studies. Several studies have investigated the psychometric characteristics of this scale; however, some psychometric properties remain unclear, among them especially a) dimensionality, b) adequacy of the Graded Response Model (GRM), and c) measurement invariance across countries.

**Methods:**

Data from 229,906 adolescents aged 11, 13 and 15 from 46 countries that participated in the 2018 HBSC survey were analyzed. Adolescents were selected using representative sampling and surveyed by questionnaire in the classroom. Dimensionality was investigated using exploratory graph analysis. In addition, we investigated whether the GRM provided an adequate description of the data. Reliability over the latent variable continuum and differential test functioning across countries were also examined.

**Results:**

Exploratory graph analyses showed that SCL can be considered as one-dimensional in 16 countries. However, a comparison of the unidimensional with a post-hoc bifactor GRM showed that deviation from a hypothesized one-dimensional structure was negligible in most countries. Multigroup invariance analyses supported configural and metric invariance, but not scalar invariance across 32 countries. Alignment analysis showed non-invariance especially for the items irritability, feeling nervous/bad temper and feeling low.

**Conclusion:**

HBSC-SCL appears to represent a consistent and reliable unidimensional instrument across most countries. This bodes well for population health analyses that rely on this scale as an early indicator of mental health status.

**Supplementary Information:**

The online version contains supplementary material available at 10.1186/s12874-022-01698-3.

## Introduction

Psychosomatic complaints can affect the health of adolescents. Such health complaints can range from typical somatic symptoms such as headache and backache, to psychological-related ones such as sadness and anxious feelings, each of which can negatively impact adolescent health and well-being. Cross-sectional studies have shown that such complaints are associated with outcomes such as low well-being at school [1], loneliness [2], schoolwork pressure [3] and insufficient sleep [4]. Longitudinal studies have shown that psychosomatic complaints in adolescence may result in lower educational attainment [5] and that they predict mental disorders in adulthood [6, 7].

### Psychosomatic complaints and the HBSC symptom checklist

Guided by the perception that psychosomatic complaints are important indicators of mental health status during adolescence, the international Health Behaviour in School-aged Children (HBSC) study developed the Symptom Checklist (SCL), a non-clinical measure of psychosomatic health measuring the prevalence of eight complaints that are common in youth: headache, stomachache, backache, feeling low, irritability/bad temper, feeling nervous, difficulties in getting to sleep and feeling dizzy [8, 9]. Since its development in the 1990s, this scale has been used extensively for analyses in peer-reviewed publications, national and international reports and associated policy analyses [10]. Despite its wide application cross-nationally (i.e., 47 countries and regions in the 2017/18 HBSC survey), there is limited contemporary evidence with regards to the psychometric properties of the HBSC-SCL and its measurement invariance across countries.

### Previous research on the psychometric properties of HBSC-SCL

Most existing studies examining the psychometric properties of the HBSC-SCL have focused on evaluating the instrument’s dimensionality (one vs two factors), validity (mostly convergent and discriminant), reliability (usually investigated by means of Cronbach’s α), and differential item functioning (DIF). This evidence is summarized below whereby some psychometric aspects have not been investigated at all.

#### Dimensionality

The dimensionality of the HBSC-SCL (especially in a cross-cultural context) is still under debate. Whereas some studies suggested a single factor solution [3, 11, 12], others proposed a two-factor solution [13–16] usually with a *somatic complaints* factor, including the items headache, stomachache, backache, and “feeling dizzy”, and a *psychological complaints* factor, including the items feeling low, irritable, feeling nervous, and difficulties in getting to sleep. However, studies implementing a two-factor solution always found considerable high inter-factor correlations (e.g., between 0.64 and 0.83 across countries; [13, 15]), questioning the usefulness (or discriminant validity) of a two-factor solution differentiating between somatic complaints and psychological complaints. Nevertheless, these studies indicate that it might be necessary to account for multidimensionality of the HBSC-SCL. However, there are different approaches to account for it. For instance, instead of employing a correlated factor model, multidimensionality could also be accounted for with a bifactor model [17]. As many different underlying causal models can generate the same set of statistical relations among indicators (known as the problem of equivalent models), direct inference from the statistical model to the causal (factor) model is inadmissible [18]. Thus, whether a bifactor or a correlated factor model is more appropriate cannot be answered by statistical model fit alone, but must be justified by theoretical considerations. It is also important to note that indicators typically contain multiple sources of variance that can reflect different levels of construct hierarchy [19]. For instance, “headache” can be caused by a physical factor, e.g. brain injury [20], but can also be a manifestation of an anxiety or depressive disorder. Thus, the item “headache” can represent a narrower construct, i.e., “physical complaints”, but also a wider construct, i.e., “psychosomatic complaints”. When a correlated factor model is employed, the HBSC-SCL items reflect two narrower constructs. Contrary, applying a bifactor model with a general factor and two specific factors conceptualizes the HBSC-SCL as representing one overall factor that might be best described as psychosomatic complaints, i.e., the bodily reactions of mental ill health while at the same time controlling for the *specific parts* of each indicator. These psychosomatic complaints can be viewed as expressions of personal suffering that are inserted in a cultural and social context [21]. Thus, culture might shape the symptom formation which might also be an explanation why studies with different cultural samples come to different conclusion regarding the dimensionality of the HBSC-SCL.

#### Adequacy of the graded response model for HBSC-SCL

HBSC-SCL was tested for DIF in a variety of ways, as explained in more detail in the next paragraph. DIF can be analysed using several statistical methods, whereby methods based on item response theory (IRT) have some methodological advantages over other methods [22]. The few DIF analyses on HBSC-SCL that are based on IRT all use the ordinal Rasch model which is also known as Partial Credit Model (PCM) [23–25]. PCM assumes a constant discrimination parameter across items. To our knowledge, HBSC-SCL has not yet been studied using other IRT models such as the Generalized Partial Credit Model (GPCM) or Graded Response Model (GRM) which estimates discrimination parameters for each item separately. Since HBSC-SCL is an instrument with ordered response categories and since items are known to differ in terms of discrimination [14, 25], GRM in particular might be an adequate IRT model [26] which is also the IRT workhorse of the Patient-Reported Outcomes Measurement Information System psychometric team [27]. However, whether GRM is also suitable in the case of HBSC-SCL has not yet been investigated.

#### Differential item functioning

HBSC-SCL was tested for DIF in terms of differences over time, countries, gender and languages. Differences over time were found in some countries, such as Switzerland [15] and Finland [24], but not in Sweden, Norway and Denmark [24, 28]. Furthermore, the item on ‘stomachache’ was found to show DIF between boys and girls [23], which was attributed in part to menstruation [8, 29].

DIF associated with survey language was studied for the four Scandinavian languages Finnish, Danish, Swedish and Norwegian in the respective countries [24], as well as for the three languages German, French and Italian used in the Swiss HBSC study [15]. In the Scandinavian countries the item on ‘feeling low’ did not appear to operate in the same manner across countries with high DIF in Finland, whereas in Switzerland the item on ‘feeling dizzy’ showed DIF across language versions. In both studies, difficulties in translating the items were discussed as possible explanations.

A cross-national study based on the 1997/98 HBSC data from 29 countries concluded that DIF is not a threat to the validity of the results [30]. However, a comparison of the 35 countries that participated in the follow-up study in 2001/02 revealed meaningful country DIF for the item “difficulties in getting to sleep” with a *R*^2^-change of 0.045 [25].

#### Reliability

In a variety of studies from countries across North America, Europe, and Asia, reliability of the HBSC-SCL in terms of Cronbach’s α has been described as acceptable (> 0.7) or even good (> 0.8); this applies to the two subscales [31] as well as to the entire list of 8 items [3, 13, 32]. However, beside the fact that Cronbach’s α has some problematic properties [33], it rests on the assumption that the standard error of measurement is uniform across the latent variable continuum, i.e., it is an empirical estimate of a measure’s *marginal reliability*. Within IRT models, in addition to marginal reliability, it is also possible to determine the *conditional reliability*, i.e., the reliability across the latent continuum.

#### Validity

In an early validation study, qualitative interviews were conducted with 38 adolescents, which demonstrated good content validity of the items. A subsequent quantitative component of this study with 344 adolescents showed adequate test–retest reliability with an intraclass correlation coefficient of 0.79 [8]. Using the Canadian sample from 2010, Gariepy et al. [14] demonstrated the convergent and discriminant validity of the psychological symptoms subscale as it correlated highly with emotional problems (*r* = 0.79), moderately with emotional well-being (*r* = 0.48), while correlation with behavioural problems was low and negative (*r* = -0.17). The validity of the somatic subscale has not yet been investigated in a similar manner.

To summarize the aforementioned psychometric studies, the validity of HBSC-SCL has been well investigated. Regarding reliability, it is unclear whether there is a uniform standard error of measurement across the latent variable continuum. Furthermore, it is still unclear whether HBSC-SCL is one-dimensional or two-dimensional. Whether HBSC-SCL can be adequately described with GRM in the context of IRT has never been investigated. Furthermore, current information on measurement invariance is lacking.

### Aim of the present study

While there has been considerable attention paid to the psychometric properties of the HBSC-SCL, most of the aforementioned studies were conducted using data from single countries or small groups of countries. Only the studies based on the 1997/98 data from 29 countries [30] and the 2001/02 data from 35 countries [12] were cross-national in nature. This is important, as the universality of the scale to capture psychosomatic complaints has not been established across countries. Since these early sentinel studies, however, the number of HBSC member countries has continued to grow to 47 countries and regions that took part in its most recent 2017/18 cycle. We therefore used the 2017/18 international data to explore several psychometric properties of HBSC-SCL. Based on the previously identified research gaps, this study has three aims. Firstly, we check whether HBSC-SCL exhibits a one-dimensional or a multidimensional factor structure within each country. In the case of multidimensionality, the extent to which the data deviates from a one-dimensional structure is assessed using a bifactor approach. Secondly, we explore whether a GRM provides an adequate IRT model that fits the data closely and we evaluate the reliability of the HBSC-SCL over the latent variable continuum. The third aim is the examination of measurement invariance (configural, metric and scalar) and differential item and test functioning across countries for which a consensus baseline model is obtained.

## Method

### Data collection and survey design

HBSC is a World Health Organization Regional Office for Europe collaborative cross-sectional study conducted every 4 years in countries across Europe and North America. The HBSC network has provided us with the data from the 2017/2018 HBSC survey, in which 47 countries or regions participated by collecting self-reported data on nationally representative samples of 11-, 13-, and 15-year-old adolescents using a standardized study protocol. Samples were drawn using cluster sampling, with school classes or the whole school as the primary sampling unit. Ethics approvals were granted by lead institutions and agencies within the participating countries. More detailed information on the methods of the HBSC study is reported elsewhere [34].

### Participants

The initial sample consisted of *N* = 244,097 adolescents from 47 countries. However, the data from North Macedonia with *n* = 4,658 respondents had to be excluded because one item of the HBSC-SCL was not incorporated as per the international protocol, reducing the sample size to *N* = 239,439 respondents from 46 countries. Of these, 4.0% (*N* = 9,533) of the records were excluded from the analyses due to incomplete data (i.e., one or more missing values on the HBSC-SCL items). Therefore, the effective sample consisted of *N* = 229,906 adolescents. The number of respondents per country ranged between 1,002 (Greenland) and 15,328 (Wales). See Table A1 in the Electronic supplement for further sample details.

### Measures

#### HBSC-SCL

HBSC-SCL comprises eight items. Adolescents were asked to indicate how often they had experienced the following complaints in the past six months: (1) headache, (2) stomachache, (3) backache, (4) feeling low, (5) irritability/bad temper, (6) feeling nervous, (7) difficulties in getting to sleep, (8) feeling dizzy. Response options were “rarely or never” (recoded as 0), “about every month” (1), “about every week” (2), “more than once a week” (3), and “about every day” (4).

### Statistical analysis

To achieve the three aims of the study, it was necessary to apply different statistical methods. In order to check the dimensionality (aim 1), exploratory graph analysis was applied. The suitability of GRM as an IRT model and reliability (aim 2) was checked by means of several measures. To check measurement invariance (aim 3), multigroup IRT and the alignment method were used. The exact procedures are explained in more detail hereafter.

The assumption of unidimensionality was evaluated by submitting the polychoric correlation matrix to exploratory graph analysis (EGA) using the glasso algorithm, a recently proposed network psychometric method for dimensionality assessment. EGA has been shown to perform well with unidimensional and multidimensional structures and to outperform many other approaches, especially in scenarios with highly correlated factors [35, 36]. The EGA assesses the number of dimensions and the relation between the indicators and the dimension in a single step [35].

The appropriateness of the unidimensional GRM was evaluated with goodness of fit statistics (RMSEA, SRMR, CFI, TLI) relying on the limited-information test statistic C_2_ developed within the IRT context [37, 38]. However, the limited-information test statistic C_2_ is relatively new (and accordingly goodness of fit statistics based on it), and this test statistic has not been evaluated deeply. In fact, research on goodness of fit statistics (based on C_2_) has shown that the RMSEA is positively correlated with the number of response categories, impeding a clear interpretation [38]. Thus, model fit should not be assessed using only these statistics alone but also using local model fit evaluation [38–41]. The assumption of local independency was investigated by means of standardized residuals in terms of (signed) Cramer’s V. Item fit was assessed with the generalized S-X^2^ item fit index [42] and corresponding item-level RMSEA values as measure of effect size. The assumption of monotonicity was investigated with raw residual plots [43]. In a next step, item and test characteristic curves (ICC, TCC) were created. Item and test information functions (IIF, TIF) were derived, and empirical marginal reliability (ρ) was calculated as summary measure of score precision [44] to evaluate the reliability of the measure and its indicators. Finally, person-item maps (also called Wright Maps) [45] were created.

As some studies found that HBSC-SCL maps on to two dimensions, we hypothesized that the HBSC-SCL would demonstrate a two-dimensional structure for at least some countries. In these cases, we applied post-hoc bifactor IRT models to investigate the degree to which ignoring the multidimensionality degrades the unidimensional solution [46, 47], or in other words, whether the HBSC-SCL items are “unidimensional enough for IRT” [17]. The factor structure of the bifactor model for each country is informed by the results of the EGA.^1^ Then, we compared the model-data fit between the unidimensional and bifactor models for each country to see whether the bifactor models are more appropriate than the unidimensional models [17]. After that, the item discrimination parameters of the unidimensional models and the marginal item discrimination parameters from the bifactor models were compared [46, 49] and the average relative bias for each country was assessed. Large differences between the discrimination parameters may indicate that a unidimensional IRT model might not be suitable or might lead to serious parameter bias. We also compared test characteristic curves and test information functions between the unidimensional models and the bifactor models to investigate whether and to what degree ignoring multidimensionality might affect person score estimates and person score reliability [46, 47]. Finally, we computed key bifactor indices [50] to further evaluate the respective bifactor models, that is explained common variance (ECV), proportion of uncontaminated correlations (PUC), construct replicability (H), and factor determinacy (FD). ECV-G represents the proportion of common variance across items explained by the general factor; thus, higher values indicate a strong general factor [47, 50]. ECV-S represents the proportion of common variance explained by the specific factor. PUC represents “the number of unique correlations in a correlation matrix that are influenced by a single factor divided by the total number of unique correlations” [50]. Higher values of PUC are indicative of less biased parameter estimates in a unidimensional model. The H index conveys information on how well the items reflect the variance of the latent variable. Finally, the FD represents the correlation between factor score estimates and factors [50]. With this procedure we followed Ten Berge and Sočan’s line of reasoning that “assessing how close is a given test to unidimensionality is far more interesting than testing whether or not the test is unidimensional” [51].

In a next step, the HBSC-SCL was tested for measurement invariance across those countries that exhibit a data structure for IRT that is unidimensional enough. First, a multigroup analysis with increasingly restrictive nested models was conducted. In the configural invariance model, the factor variances were fixed to one and the factor means were fixed to zero, whereas the discrimination and difficulty parameters were freely estimated in all countries. In the next more restrictive metric invariance model, the discrimination parameters were constrained to be equal across all countries, whereas the factor variance was fixed to one only for the first group. The factor means and difficulty parameters remained unchanged. In the third model, the scalar invariance model, in addition to the discrimination parameters the difficulty parameters are also constrained to be equal across countries. The factor variance was fixed to one and the factor mean was fixed to zero only for the first group, whereas they were freely estimated in all other countries.

To identify non-invariant parameters, the alignment optimization method [52–55] with maximum likelihood estimator with robust standard errors (MLR) and numerical integration was employed. Starting from the configural invariance model and based on a simplicity function, the alignment method searches for parameters that can be constrained across countries without loss in model fit. Thus, the aligned model has the same model fit as the configural invariance model [53] and “serves the joint purposes of scale linking and purification, without literally deleting items from the linking” [52]. In a first step, the alignment procedure first determines a starting set of invariant parameters by a pairwise significance test (α = 0.01) for each pair of groups. In a next step, significance tests (α = 0.001) are conducted to compare the parameter values for each group with the parameter average of the invariant groups. The alignment procedure also provides *R*^2^-like measures that represent variations in parameters across groups in the configural model that are not the result of differential item functioning but can be explained by variation in factor mean and factor variance across groups, thus, are the result of a different metric. Therefore, higher *R*^2^ values imply a higher degree of invariance.

Two alignment optimization methods can be used. In the FIXED approach, the factor mean and factor variance of a reference group is set to 0 and 1, respectively. Typically, the group with factor mean closest to 0 is used as reference group, to avoid misspecification and estimation biases. In the FREE approach there is no constraint on the first group’s factor mean, and it is freely estimated [53]. Asparouhov and Muthén recommend starting with the FREE approach when more than two groups are being compared and when measurement non-invariance exists. However, under certain conditions (e.g., insufficient measurement non-invariance), the FREE method might be poorly identified, then Asparouhov and Muthén recommend switching to the FIXED method. For methodological and technical details such as the computation of the simplicity function, see [53, 54, 56]. Because the reliability of the alignment method depends on the quality of the factor mean group ranking, Muthén and Asparouhov [55] recommend a Monte Carlo simulation study if more than 25% of parameters are non-invariant. A near-perfect correlation (i.e., *r* = 0.98 or higher) between the generated factor means (computed over groups and averaged over replications) and estimated factor means is required for the ordering of countries with respect to factor means to be trustworthy [55]. Thus, we checked the stability of the country ranking with a simulation study with 500 simulation runs as this number has been shown to be sufficient to check the reliability of the alignment results [53]. The aligned IRT parameters were used to further analyze country pairwise differential test functioning with the compensatory (sDRF) and non-compensatory differential response functioning (uDRF) statistics [57].

## Results

### Descriptives

The items of the HBSC-SCL showed some amount of skewness and kurtosis (M_skewness_ = 1.06, SD_skewness_ = 0.55, Min_skewness_ = -0.37, Max_skewness_ = 2.95, M_kurtosis_ = 0.27, SD_kurtosis_ = 1.50, Min_kurtosis_ = -1.66, Max_kurtosis_ = 8.46; see Table A2/A3 and Figure A1 in the Electronic supplement). The items’ polychoric correlations ranged between 0.20 and 0.82 (M_polycor_ = 0.43, SD_polycor_ = 0.10). Bulgaria showed the lowest average item intercorrelations and the highest item intercorrelations variation (M_polycor_ = 0.31, SD_polycor_ = 0.12, Min_polycor_ = 0.20, Max_polycor_ = 0.76) whereas Israel showed the highest average item intercorrelations (M_polycor_ = 0.57, SD_polycor_ = 0.09, Min_polycor_ = 0.46, Max_polycor_ = 0.82, see Figure A2 in the Electronic supplement). The polychoric correlation matrix for some countries further indicated that a unidimensional IRT model might not be suitable for all countries, with the items feeling low, irritability/bad temper, and feeling nervous (i.e., psychological complaints) associate with higher inter-item correlations.

### Assessment of dimensionality and the unidimensional GRM

**Fig. 1:**
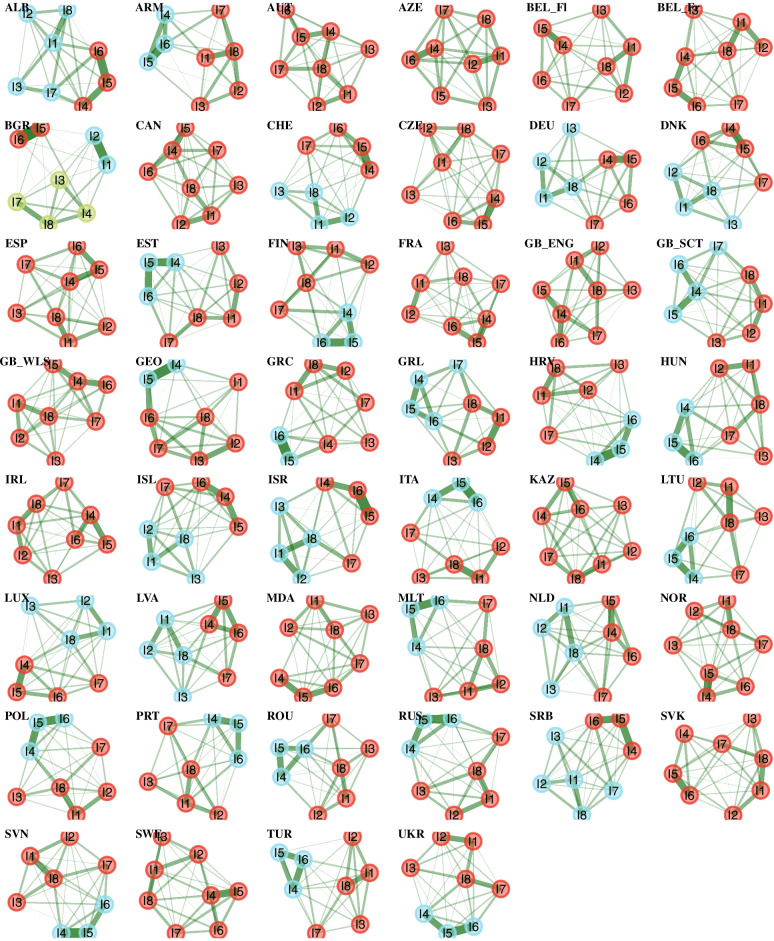
Exploratory graph analysis. Note. Each color represents a cluster of items (latent dimension). Nodes (circles) represent observed variables, and edges (lines) represent partial correlations. The magnitude of the partial correlation is represented by the thickness of the edges. Items (1) headache, (2) stomachache, (3) backache, (4) feeling low, (5) irritability/bad temper, (6) feeling nervous, (7) difficulties in getting to sleep, (8) feeling dizzy. The abbreviations of the country names can be found in Table 1

The EGA indicated a one-factor solution for 16 countries, a two-factor solution for 29 countries, and a three-factor solution for Bulgaria (see Fig. 1).

Although the unidimensionality assumption was violated for most of the countries according to EGA, we employed a unidimensional GRM for all countries that served as a baseline model for the (post-hoc) bifactor GRM for those countries that showed a violation of the unidimensionality assumption. Table 1 shows the goodness of fit statistics based on the test statistic C_2_ for the unidimensional GRM. These statistics are assessed based on commonly used thresholds, i.e., RMSEA ≤ 0.08 is considered acceptable and ≤ 0.05 is good, both CFI and TLI ≥ 0.90 are acceptable and ≥ 0.95 are good, SRMR ≤ 0.10 is acceptable and ≤ 0.08 is good [58–60]. CFI indicated a poor model fit for Bulgaria, whereas Italy just misses the threshold of 0.90. For the TLI, the model fit for Bulgaria was also poor, whereas Italy, Armenia, Greece, Israel and Turkey fall less short of the 0.90 threshold. The SRMR showed values above 0.10 for Armenia, Bulgaria, Georgia, Israel, and Italy. Finally, the RMSEA showed values above 0.10 for 17 countries. The standardized residuals (in terms of signed Cramer’s V coefficients) ranged between 0.23 and 0.35 (M_res_cor_ = -0.01, SD_res_cor_ = 0.08, see Figure A3 in the Electronic supplement). Especially the items (4) feeling low, (5) irritability/bad temper, and (6) feeling nervous (i.e., the psychological complaints) showed often higher residuals. The generalized S-X^2^ item fit index flagged most of the items to deviate from the GRM curves (see Figure A4 in the Electronic supplement). However, corresponding item-level RMSEA were quite small (M_RMSEA_ = 0.014, SD_RMSEA_ = 0.009, Min_RMSEA_ = 0.000, Max_RMSEA_ = 0.053), indicating low to medium deviation of the items from the GRM. The raw residual plots indicated no strong deviation from the GRM curves, except for Bulgaria and Georgia (see Figure A5,A6, A7, A8, A9, A10, A11, A12, A13, A14, A15, A16, A17, A18, A19, A20, A21, A22, A23, A24, A25, A26, A27, A28, A29, A30, A31, A32, A33, A34, A35, A36, A37, A38, A39, A40, A41, A42, A43, A44, A45, A46, A47, A48, A49 and A50 in the Electronic supplement).

**Table 1:** Goodness of fit statistics for the unidimensional graded response model

Country	C_2_	p	RMSEA [90% CI]	SRMR	TLI	CFI
ALB—Albania	221.415	.000	.078 [.069; .087]	.053	.951	.965
ARM—Armenia	890.237	.000	.106 [.100; .112]	.081	.879	.913
AUT—Austria	239.046	.000	.052 [.046; .058]	.033	.978	.984
AZE—Azerbaijan	443.771	.000	.070 [.064; .076]	.062	.965	.975
BEL-FL—Belgium- Flemish	460.351	.000	.072 [.066; .078]	.048	.933	.952
BEL-FR—Belgium-French	839.095	.000	.087 [.082; .093]	.055	.908	.934
BGR—Bulgaria	2725.184	.000	.172 [.167; .178]	.132	.576	.697
CAN—Canada	1384.652	.000	.074 [.071; .078]	.044	.966	.976
CHE—Switzerland	1674.418	.000	.106 [.102; .110]	.064	.906	.933
CZE—Czechia	817.068	.000	.061 [.057; .064]	.046	.960	.971
DEU—Germany	661.651	.000	.086 [.081; .092]	.054	.933	.952
DNK—Denmark	469.405	.000	.085 [.079; .092]	.055	.938	.955
ESP—Spain	357.661	.000	.063 [.057; .069]	.043	.966	.976
EST—Estonia	927.106	.000	.098 [.093; .104]	.062	.943	.959
FIN—Finland	793.860	.000	.112 [.105; .118]	.062	.934	.953
FRA—France	795.871	.000	.067 [.063; .071]	.045	.953	.966
GB-ENG England	329.519	.000	.069 [.062; .076]	.043	.961	.972
GB-SCT Scotland	565.362	.000	.075 [.069; .080]	.048	.963	.974
GB-WLS Wales	1379.060	.000	.067 [.064; .070]	.040	.966	.976
GEO—Georgia	1247.363	.000	.128 [.122; .134]	.085	.914	.938
GRC—Greece	1022.136	.000	.115 [.109; .121]	.071	.878	.913
GRL—Greenland	250.647	.000	.107 [.096; .119]	.069	.917	.941
HRV—Croatia	1056.063	.000	.104 [.099; .110]	.075	.924	.946
HUN—Hungary	1062.855	.000	.119 [.113; .125]	.066	.920	.943
IRL—Ireland	408.206	.000	.073 [.066; .079]	.044	.964	.974
ISL—Iceland	1115.015	.000	.090 [.085; .094]	.045	.959	.970
ISR—Israel	5048.693	.000	.181 [.176; .185]	.084	.868	.906
ITA—Italy	1701.896	.000	.143 [.138; .149]	.090	.840	.885
KAZ—Kazakhstan	227.914	.000	.048 [.043; .054]	.040	.983	.988
LTU—Lithuania	847.127	.000	.106 [.100; .112]	.070	.935	.953
LUX—Luxembourg	503.568	.000	.078 [.072; .084]	.049	.939	.956
LVA—Latvia	669.419	.000	.087 [.081; .092]	.049	.957	.969
MDA—Republic of Moldova	613.553	.000	.082 [.076; .087]	.052	.933	.952
MLT—Malta	748.913	.000	.121 [.114; .128]	.065	.907	.934
NLD—Netherland	475.433	.000	.070 [.065; .075]	.042	.963	.973
NOR—Norway	367.071	.000	.076 [.069; .083]	.048	.958	.970
POL—Poland	1096.477	.000	.103 [.098; .108]	.068	.905	.932
PRT—Portugal	667.676	.000	.073 [.069; .078]	.054	.952	.966
ROU—Romania	594.334	.000	.081 [.075; .087]	.053	.941	.958
RUS—Russian Federation	1300.431	.000	.124 [.118; .130]	.069	.913	.938
SRB—Serbia	410.702	.000	.072 [.066; .078]	.049	.954	.967
SVK—Slovakia	491.252	.000	.073 [.067; .079]	.045	.941	.958
SVN—Slovenia	1055.592	.000	.097 [.092; .102]	.070	.938	.956
SWE—Sweden	504.601	.000	.078 [.072; .084]	.043	.960	.971
TUR—Turkey	1217.266	.000	.103 [.098; .108]	.066	.888	.920
UKR—Ukraine	1404.931	.000	.105 [.100; .109]	.064	.913	.938

Notes. *df* 20, *RMSEA* Root mean squared error of approximation, *SRMR* Standardized root mean square residual, *TLI* Tucker-Lewis index, *CFI* Comparative fit index

**Fig. 2:**
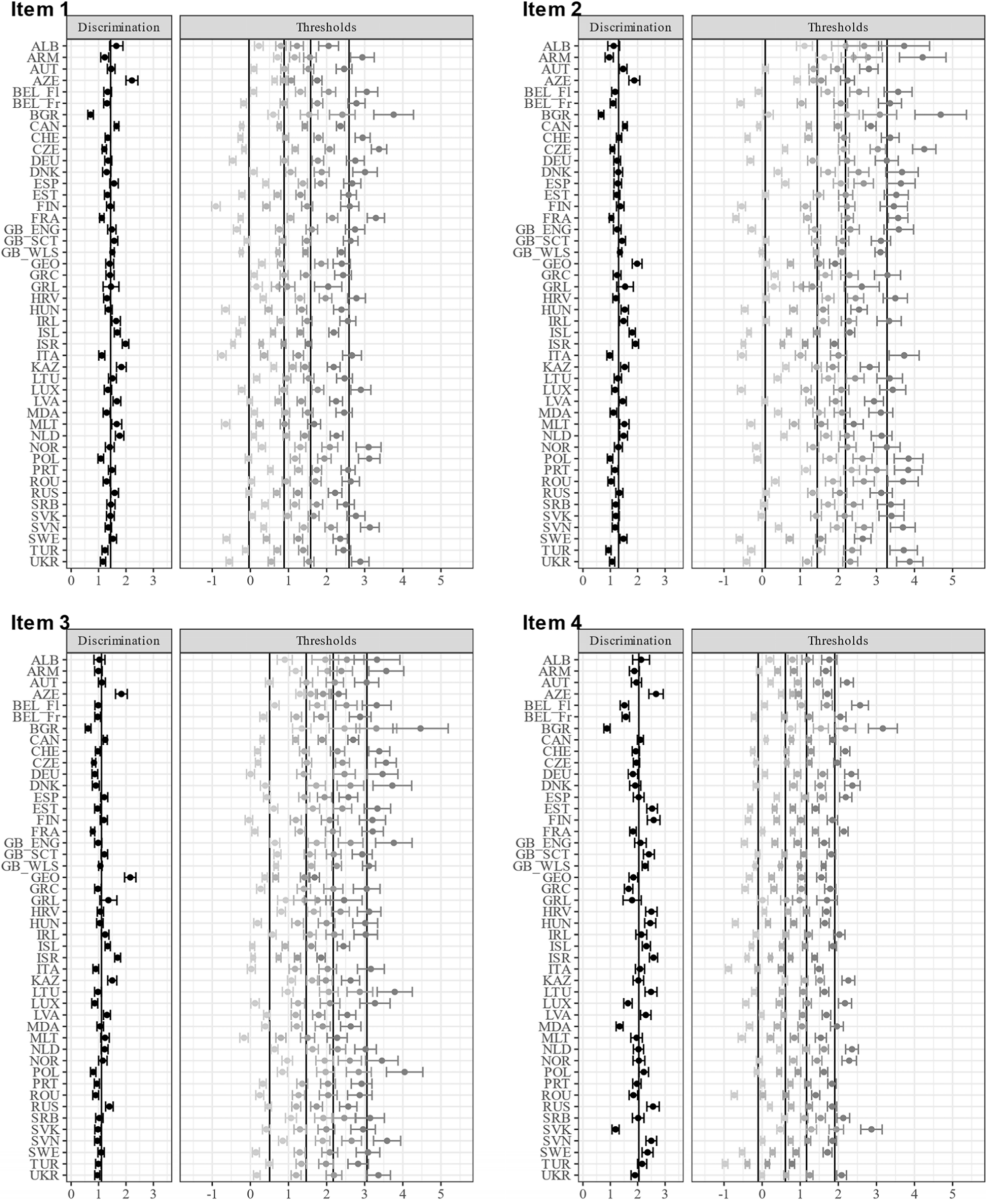
Item parameter for the unidimensional GRM (Items 1–4).Notes. Item discrimination and threshold parameters with 95% CI. Items (1) headache, (2) stomachache, (3) backache, (4) feeling low. Item 1: mean = 1.44, SD = 0.25, minimum = 0.71, maximum = 2.22. Item 2: mean = 1.30, SD = 0.27, minimum = 0.66, maximum = 1.98. Item 3: mean = 1.11, SD = 0.28, minimum = 0.62, maximum = 2.15. Item 4: mean = 2.04, SD = 0.38, minimum = 0.88, maximum = 2.67. The abbreviations of the country names can be found in Table 1

**Fig. 3:**
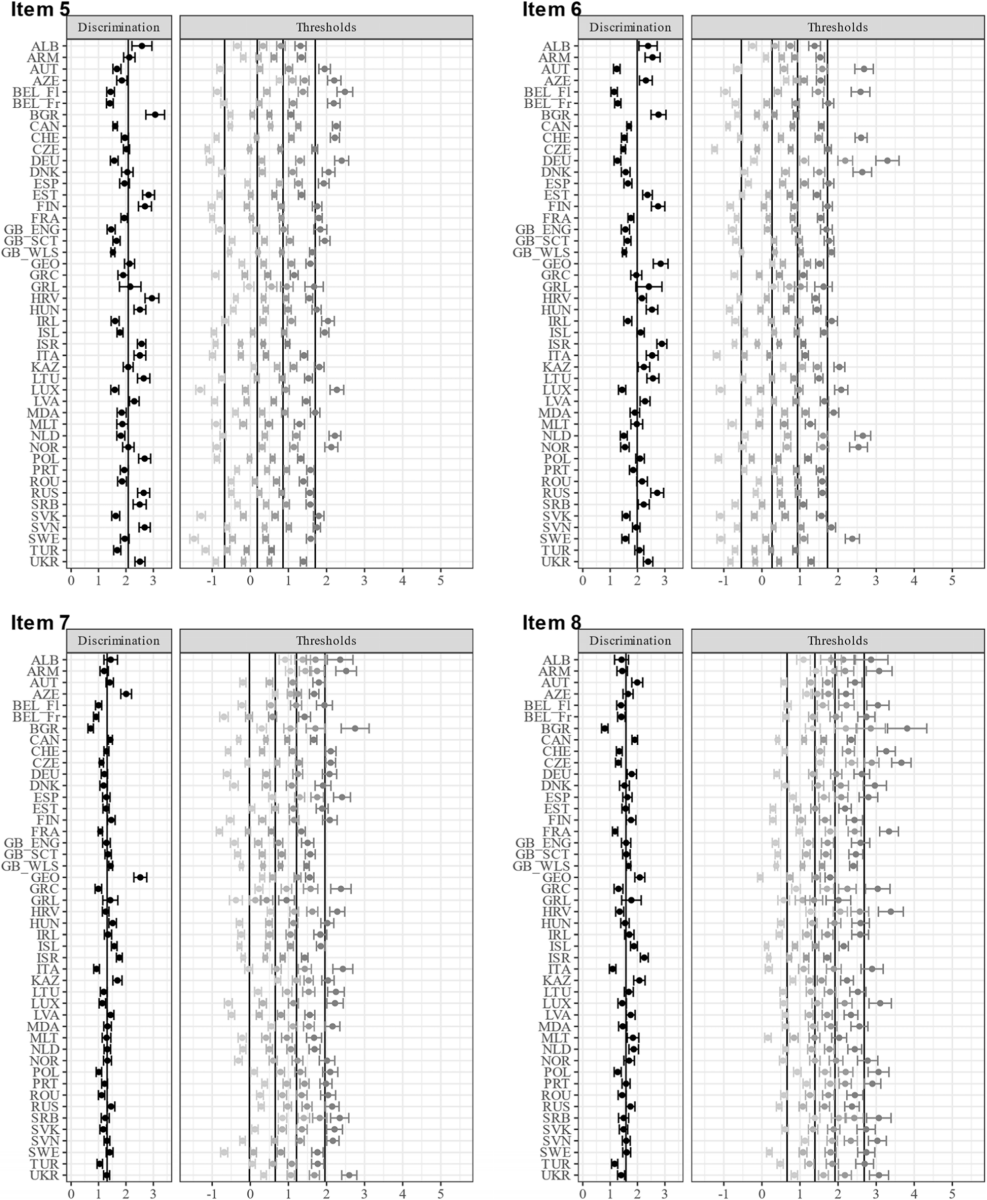
Item parameter for the unidimensional GRM (Items 5–8). Notes. Item discrimination and threshold parameters with 95% CI. Items (5) irritability/bad temper, (6) feeling nervous, (7) difficulties in getting to sleep, (8) feeling dizzy. Item 5: mean = 2.08, SD = 0.45, minimum = 1.41, maximum = 3.07. Item 6: mean = 1.99, SD = 0.49, minimum = 1.14, maximum = 2.89. Item 7: mean = 1.31, SD = 0.29, minimum = 0.71, maximum = 2.52. Item 8: mean = 1.58, SD = 0.28, minimum = 0.80, maximum = 2.25. The abbreviations of the country names can be found in Table 1

**Fig. 4:**
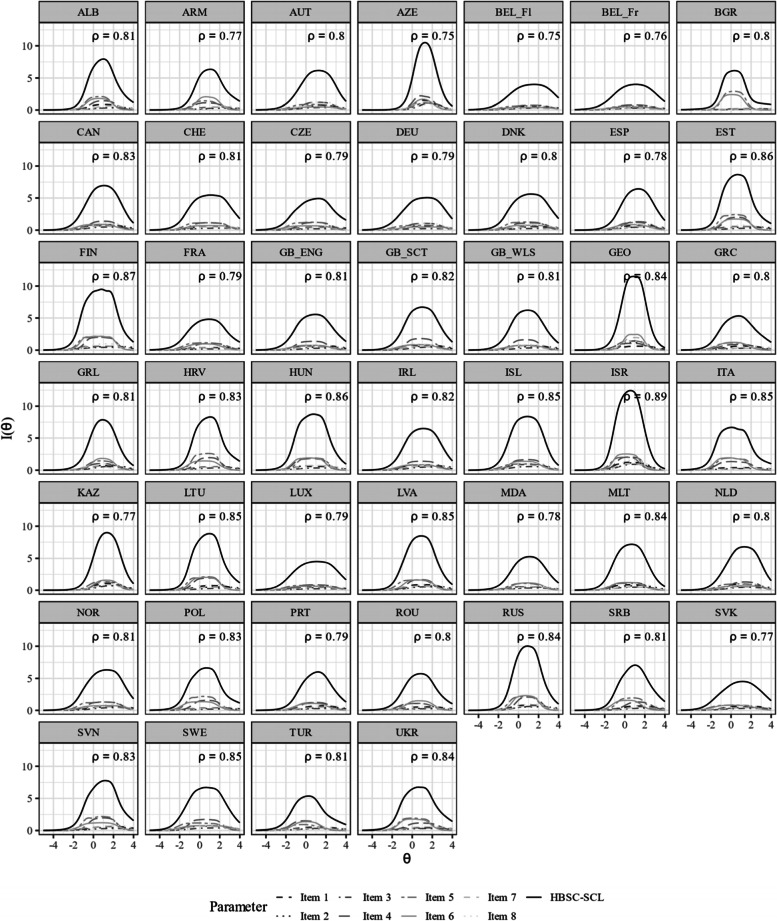
Item and test information functions for the unidimensional GRM. Notes. ρ represents the empirical marginal reliability. Items (1) headache, (2) stomachache, (3) backache, (4) feeling low, (5) irritability/bad temper, (6) feeling nervous, (7) difficulties in getting to sleep, (8) feeling dizzy. The abbreviations of the country names can be found in Table 1

Item characteristic curves revealed quite a variation in discriminatory power within some countries (see Figure A51 and A52 for the item and test characteristic curves in the electronic supplement). This variation is partly explained by the violation of the local independency assumption (e.g., Bulgaria). Figures 2 and 3 show the item parameter of the unidimensional GRM for each country. The items (4) feeling low, (5) irritability/bad temper, and (6) feeling nervous yielded on average higher discrimination parameters. Figure 4 shows the item and test information functions (see Figure A53 in the Electronic supplement for the item information functions in greater detail and in color). Clearly, the items differed regarding the information provided. The items (4) feeling low, (5) irritability/bad temper, and (6) feeling nervous provide the highest amount of test information for the majority of the countries. Again, this is partly explained by the violation of the local independency assumption. The empirical marginal reliability ranged between 0.75 and 0.89. The person-item maps show the distribution of the factor scores and item thresholds (see Figure A54, A55, A56 and A57 in the Electronic supplement).

### Comparing the unidimensional GRM with a post-hoc bifactor GRM

Beside the results of the EGA, several other indicators of the IRT analysis indicated a need to account for multidimensionality for several countries. Thus, we employed a post-hoc bifactor GRM for all countries where EGA indicated multidimensionality of the data.^2^ The bifactor GRM for each country was informed by the factor structure suggested from EGA. For instance, the bifactor model for Albania consisted of all items being explained by a general factor, and then each subset of items to be explained by a specific factor (i.e., the five complaints (1) headache, (2) stomachache, (3) backache, (7) problems falling asleep and (8) feeling dizzy were allowed to load on one specific factor, and the complaints (4) feeling low, (5) irritability/bad temper, and (6) feeling nervous were allowed to load on another specific factor). The general and the specific factors were not allowed to covary [46].

The goodness of fit statistics of the bifactor GRM indicated remarkably good model fit (see Table A4 in the Electronic supplement for all goodness of fit statistics). The RMSEA ranged between 0.000 and 0.044, the SRMR between 0.012 and 0.058, the TLI between 0.990 and 1.000, and the CFI between 0.996 and 1.000. The standardized residuals (in terms of signed Cramer’s V coefficients) ranged between -0.30 and 0.35 (M_res_cor_ = -0.03, SD_res_cor_ = 0.08, see Figure A58 in the Electronic supplement). Many standardized residuals between items switched from positive to negative. Negative residuals are typically ignored, because they do not inflate discrimination parameters, but underestimate it [61]. The S-X^2^ item fit indices and corresponding item-level RMSEA remained largely unchanged between the unidimensional and the bifactor models (see Figure A59 in the Electronic supplement). However, it is important to note that the item fit indices are only accurate under the assumption that the number of latent variables are correctly specified by the respective IRT model [46]. Thus, the item fit indices may not be valid for the unidimensional GRM.

Comparing the item discrimination parameters of the unidimensional GRM with the marginal item discrimination parameters from the bifactor GRM (see Figure A60 in the Electronic supplement) revealed only minor differences for some countries (e.g., Netherlands with M_Difference_ = 0.14, SD_Difference_ = 0.10, Min_Difference_ = 0.02, Max_Difference_ = 0.31), whereas other countries showed quite large differences (e.g., Georgia with M_Difference_ = 1.06, SD_Difference_ = 0.65, Min_Difference_ = 0.324, Max_Difference_ = 2.57). The average relative bias ranged between 7.8% and 49.6% where bias up to 10%-15% is often considered negligible [50, 62]. With regard to person score estimates, the test characteristic curves (see Figure A61 in the Electronic supplement) and the scatter plots (see Figure A62 in the Electronic supplement) indicate considerable differences between the unidimensional and bifactor GRM for Georgia, Italy, Poland and Ukraine (*r* < 0.95). As expected, the average relative bias was strongly correlated with the correlation between the unidimensional GRM scores and the bifactor GRM scores of the general dimension (*r* = -0.97). With regard to person score reliability, the unidimensional GRM showed a higher amount of test information compared to the bifactor GRM (see Figure A63 in the Electronic supplement). The change of the empirical marginal reliability between the unidimensional GRM and the bifactor GRM ranged between -0.02 and -0.13 (M_Δρ_ = -0.08, SD_Δρ_ = 0.03). Thus, ignoring multidimensionality can lead to inflated person score precision [46, 47]. Finally, regarding the bifactor indices, the ECV-G values ranged between 0.64 and 0.82 (M_ECV-G_ = 0.75, SD_ECV-G_ = 0.05), the ECV-S values ranged between 0.00 and 0.29 (M_ECV-S_ = 0.13, SD_ECV-S_ = 0.06), the PUC values between 0.43 and 0.57 (M_PUC_ = 0.54, SD_PUC_ = 0.04), the H-G values between 0.82 and 0.93 (M_H-G_ = 0.86, SD_H-G_ = 0.03), and the FD-G values between 0.86 and 0.96 (M_FD-G_ = 0.91, SD_FD-G_ = 0.03; see Table A5 in the Electronic supplement). Thus, whereas the H-G and FD-G values were all within an acceptable range, ECV-G and PUC values were quite low for some countries [50].

These analyses give an indication of how severe the bias will be, when a unidimensional measurement model is forced on the HBSC-SCL for each country. The amount of parameter bias that is deemed tolerable or acceptable depends highly on the research context. Thus, we refrained from stating which country has “passed” the unidimensionality test. Nevertheless, we assessed measurement invariance/differential test functioning for all countries with an average relative bias of less than 20%,^3^ (i.e., Armenia, Croatia, Denmark, Finland, Germany, Greece, Iceland, Israel, Latvia, Lithuania, Malta, the Netherlands, Portugal, Scotland, Switzerland, and Turkey) together with all countries where EGA indicated unidimensionality.

### Differential test functioning and measurement invariance analysis

The multigroup analysis revealed a very good fit for the configural model (C_2_ = 27,563.346, df = 640, *p* = 0.000, RMSEA [90% CI] = 0.016 [0.015; 0.016], SRMR for each country ranged between 0.033 and 0.084, TLI = 0.941, CFI = 0.958, see Table A6 in the Electronic supplement), indicating that the model structure is the same across countries. Constraining the discrimination parameter to be equal (metric invariance model) across countries had almost no effect on model fit (C_2_ = 30,062.355, df = 888, *p* = 0.000, RMSEA [90% CI] = 0.014 [0.014; 0.014], SRMR for each country ranged between 0.042 and 0.085, TLI = 0.954, CFI = 0.955), indicating the same metric of the HBSC-SCL in the countries. However, constraining the item thresholds to be equal (scalar invariance model) across countries lead to a substantial loss in model fit according to some goodness of fit statistics (C_2_ = 102,507.217, df = 1880, *p* = 0.000, RMSEA [90% CI] = 0.018 [0.017; 0.018], SRMR for each country ranged between 0.049 and 0.181, TLI = 0.925, CFI = 0.843), indicating non-invariance for at least some threshold parameters.

To identify non-invariant parameters, we started the alignment method with the FREE approach which resulted in a poorly identified model. Thus, we switched to the FIXED approach as recommended by Asparouhov and Muthén [53] with Finland as reference group as indicated by Mplus.

**Fig. 5:**
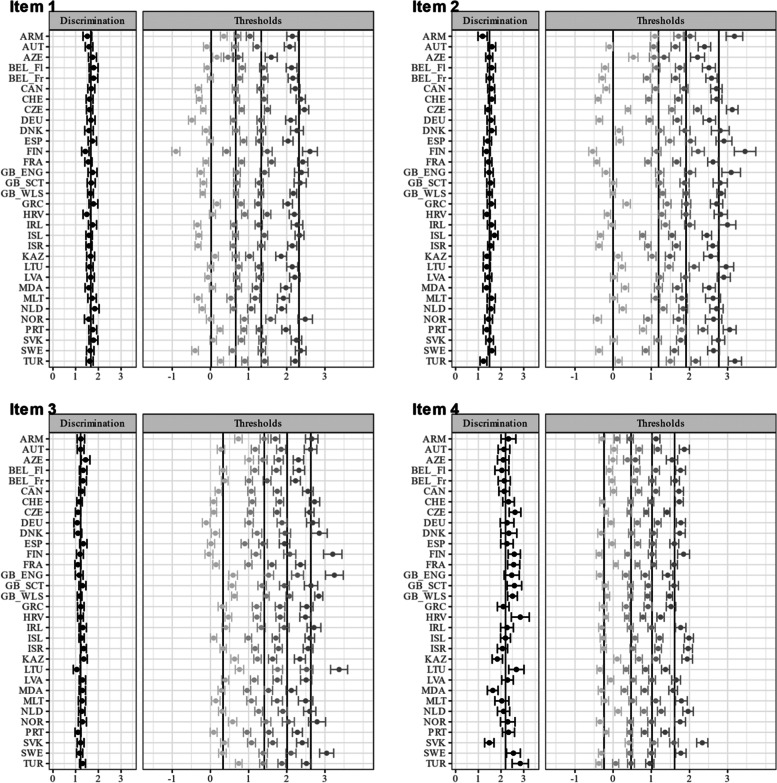
Item parameter for the unidimensional GRM after alignment (Items 1–4). Notes. Item discrimination and threshold parameters with 95% CI. Vertical lines represent weighted average across all groups. Items (1) headache, (2) stomachache, (3) backache, (4) feeling low. The abbreviations of the country names can be found in Table 1

**Fig. 6:**
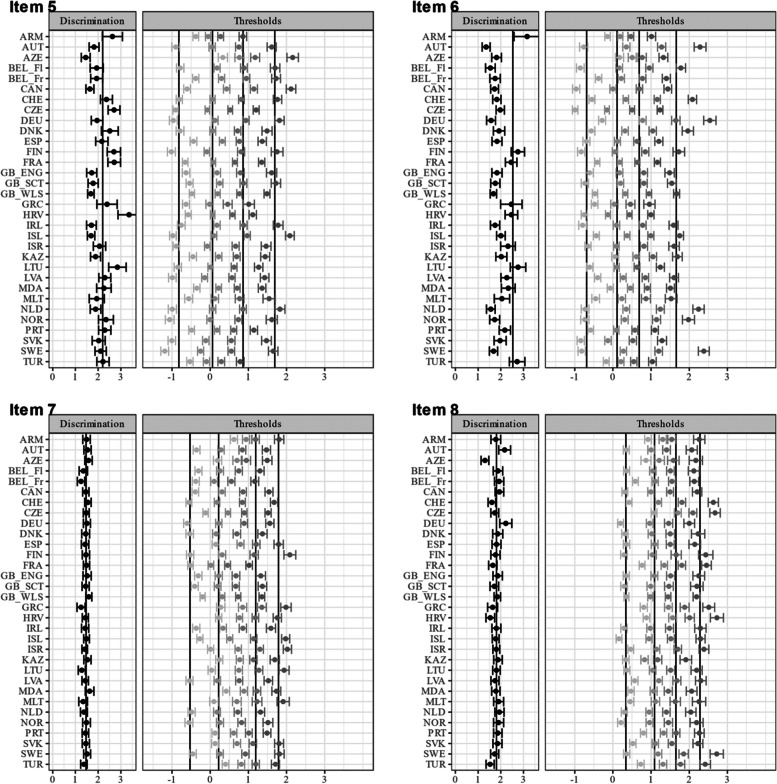
Item parameter for the unidimensional GRM after alignment (Items 5–8). Notes. Item discrimination and threshold parameters with 95% CI. Vertical lines represent weighted average across all groups. Items (5) irritability/bad temper, (6) feeling nervous, (7) difficulties in getting to sleep, (8) feeling dizzy. The abbreviations of the country names can be found in Table 1

Table 2 shows the fit statistics of the alignment analysis with the FIXED approach and with Finland as reference group (with mean fixed to 0 and variance fixed to 1). The average invariance index (mean over all R^2^ values) equaled 0.484, and 50.9% of the parameters were flagged as being non-invariant. The *R*^2^ values for the item discrimination ranged between 0.000 and 0.876 (M_R2_ = 0.589; SD_R2_ = 0.323) and the percentage of approximate invariant countries between 31.2% and 93.8%. Non-invariance was especially prevalent within the item discrimination of items (4) feeling low, (5) irritability/bad temper and (6) feeling nervous, that showed the lowest R^2^ values, the highest variance across all countries, and the lowest percentage of invariant countries. The *R*^2^ values for the item thresholds ranged between 0.040 and. 782 (M_R2_ = 0.458; SD_R2_ = 0.177) and the percentage of approximate invariant countries between 18.8% and 78.1%. The simulation study revealed a very high factor mean country ranking stability (i.e., *r* = 0.997 between the generated and estimated country factor means), indicating reliable alignment results even though more than half of the parameters were flagged as non-invariant. The proportion of replications for which the 95% confidence interval contains the mean ranged between 93.4% and 97.6% (M = 95.4; SD = 0.01; see Table A7 in the Electronic Supplement for all information). Figures 5 and 6 show the item parameters of the unidimensional GRM after the alignment procedure. These parameters can be directly compared because of the scale linking via alignment. These Figures corroborate the finding that the discrimination parameters of (5) irritability/bad temper and (6) feeling nervous showed greater variation, thus, a higher degree of non-invariance across countries.

**Table 2:** Alignment fit statistics

Item	Parameter	R^2^	Weighted Average across invariant groups	Weighted Variance across invariant groups	Weighted Average across all groups	Weighted Variance across all groups	Number (percentage) of approx. invariant groups
Item 1	Discrimination	.863	1.67	0.08	1.66	0.09	30 (93.8%)
	Threshold 1	.575	0.02	0.03	-0.13	0.22	7 (21.9%)
	Threshold 2	.736	0.66	0.04	0.71	0.11	17 (53.1%)
	Threshold 3	.306	1.33	0.07	1.32	0.16	19 (59.4%)
	Threshold 4	.274	2.32	0.13	2.20	0.19	17 (53.1%)
Item 2	Discrimination	.825	1.49	0.07	1.48	0.11	29 (90.6%)
	Threshold 1	.460	0.00	0.02	-0.01	0.35	6 (18.8%)
	Threshold 2	.569	1.19	0.11	1.19	0.25	13 (40.6%)
	Threshold 3	.327	1.91	0.11	1.85	0.23	13 (40.6%)
	Threshold 4	.178	2.77	0.18	2.77	0.25	22 (68.8%)
Item 3	Discrimination	.847	1.22	0.08	1.23	0.09	30 (93.8%)
	Threshold 1	.512	0.33	0.04	0.34	0.25	11 (34.4%)
	Threshold 2	.607	1.41	0.12	1.19	0.19	11 (34.4%)
	Threshold 3	.537	2.01	0.18	1.81	0.21	12 (37.5%)
	Threshold 4	.378	2.63	0.20	2.59	0.27	20 (62.5%)
Item 4	Discrimination	.426	2.20	0.11	2.31	0.29	20 (62.5%)
	Threshold 1	.693	-0.24	0.05	-0.14	0.17	13 (40.6%)
	Threshold 2	.686	0.47	0.07	0.50	0.18	16 (50%)
	Threshold 3	.444	1.02	0.16	0.99	0.21	21 (65.6%)
	Threshold 4	.426	1.61	0.18	1.63	0.26	17 (53.1%)
Item 5	Discrimination	.000	2.20	0.19	2.13	0.43	17 (53.1%)
	Threshold 1	.462	-0.82	0.15	-0.68	0.27	8 (25%)
	Threshold 2	.782	0.06	0.04	0.11	0.19	12 (37.5%)
	Threshold 3	.677	0.86	0.20	0.74	0.21	12 (37.5%)
	Threshold 4	.415	1.69	0.32	1.53	0.34	13 (40.6%)
Item 6	Discrimination	.316	2.53	0.24	2.01	0.39	10 (31.2%)
	Threshold 1	.508	-0.69	0.11	-0.57	0.28	11 (34.4%)
	Threshold 2	.664	0.11	0.05	0.20	0.20	10 (31.2%)
	Threshold 3	.530	0.69	0.16	0.82	0.26	10 (31.2%)
	Threshold 4	.243	1.66	0.32	1.55	0.39	10 (31.2%)
Item 7	Discrimination	.876	1.47	0.06	1.46	0.09	28 (87.5%)
	Threshold 1	.319	-0.53	0.04	-0.17	0.32	9 (28.1%)
	Threshold 2	.448	0.22	0.04	0.44	0.26	12 (37.5%)
	Threshold 3	.475	1.19	0.06	0.93	0.24	11 (34.4%)
	Threshold 4	.365	1.79	0.10	1.58	0.26	11 (34.4%)
Item 8	Discrimination	.561	1.81	0.08	1.79	0.16	26 (81.2%)
	Threshold 1	.454	0.34	0.04	0.50	0.25	15 (46.9%)
	Threshold 2	.439	1.09	0.10	1.15	0.21	18 (56.2%)
	Threshold 3	.117	1.65	0.16	1.61	0.20	17 (53.1%)
	Threshold 4	.040	2.28	0.17	2.30	0.20	25 (78.1%)

Notes. MLR estimator; FIXED approach. Items (1) headache, (2) stomachache, (3) backache, (4) feeling low, (5) irritability/bad temper, (6) feeling nervous, (7) difficulties in getting to sleep, (8) feeling dizzy

**Fig. 7:**
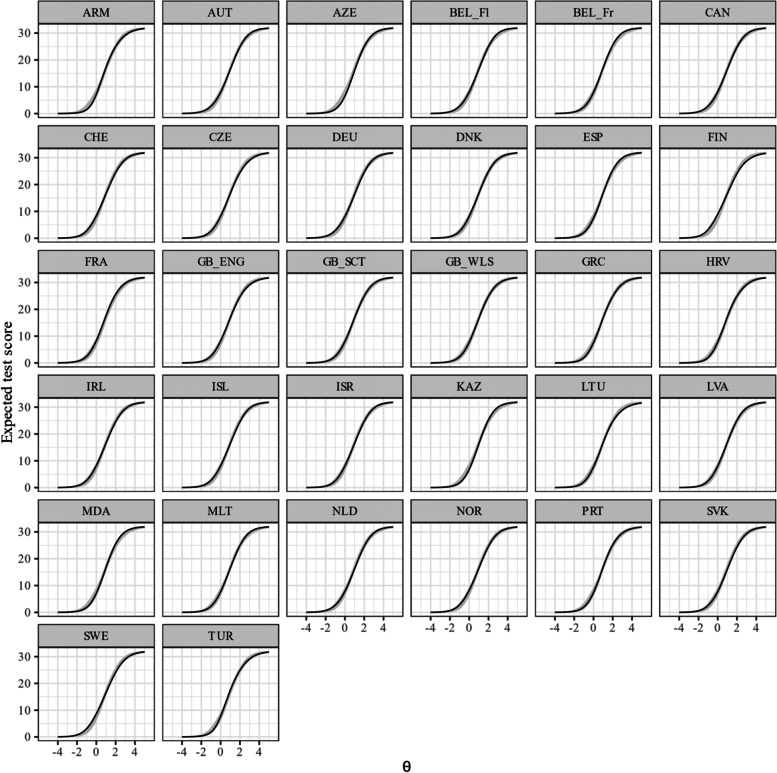
Test characteristic curves for the GRM after alignment. The abbreviations of the country names can be found in Table 1

**Fig. 8:**
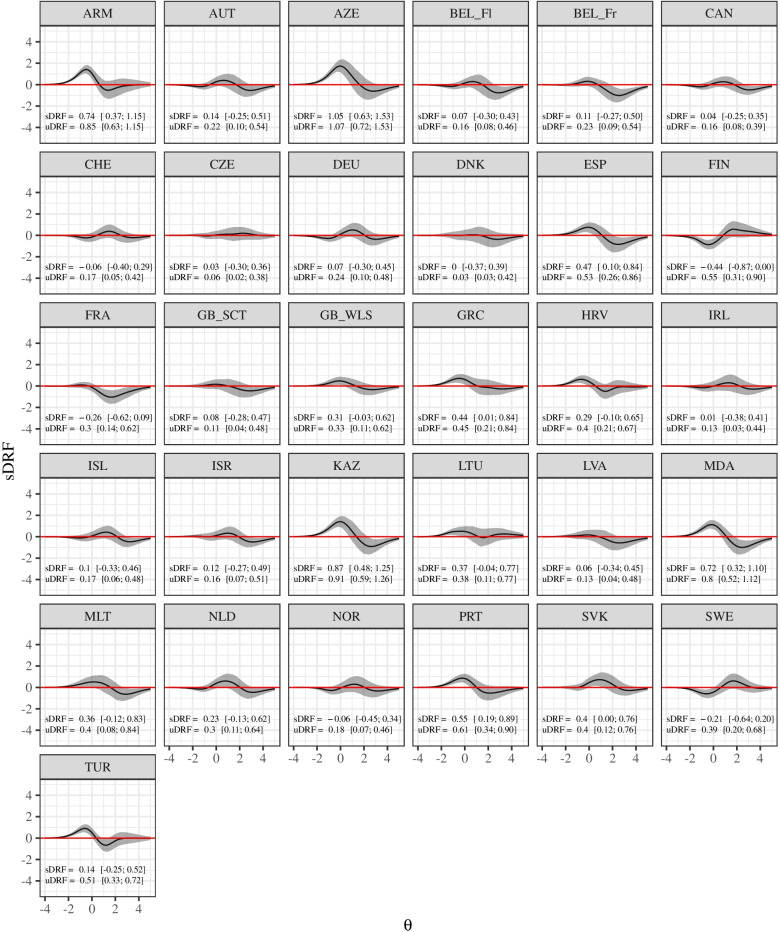
Differential test functioning for the GRM after alignment. Notes. The curves show differences in expected test scores (with 99% CI) dependent on the level of the latent variable (GB_Eng as reference group), sDRF = compensatory differential response functioning statistic with 99% CI, uDRF = non-compensatory differential response functioning statistic with 99% CI. The abbreviations of the country names can be found in Table 1

Figure 7 shows the test characteristic curves of the unidimensional GRM after alignment. Exemplary, it can be seen that at lower levels on the latent variable the expected test scores were especially low for Armenia, whereas at higher levels on the latent variable the expected test scores were especially high for Moldova. Figure 8 gives a more fine-grained insight in the differential test functioning across countries. It shows the difference in expected test scores dependent on the level of the latent variable together with the sDRF and uDRF statistics with England as reference group (for the other country comparisons see Figure A65, A66, A67, A68, A69, A70, A71, A72, A73, A74, A75, A76, A77, A78, A79, A80, A81, A82, A83, A84, A85, A86, A87, A88, A89, A90, A91, A92, A93, A94 and A95 in the Electronic supplement). Positive values indicate that adolescents in England had higher expected test scores, whereas negative values indicate that the other group had higher expected test scores. The sDRF and uDRF statistics summarize the differential test functioning across the full range of the latent variable. For example, when comparing England and the Czech Republic only minor differential test functioning effects occurred, whereas differential test functioning is larger between England and Armenia. Considering all country comparisons, the sDRF statistics ranged between -1.17 and 1.41 (M_sDRF_ = 0.13, SD_sDRF_ = 0.45) and the uDRF statistics between 0.03 and 1.49 (M_uDRF_ = 0.47, SD_uDRF_ = 0.29; see also Figure A96 in the Electronic Supplement).

### Comparing alignment factor scores and manifest sum scores

**Fig. 9:**
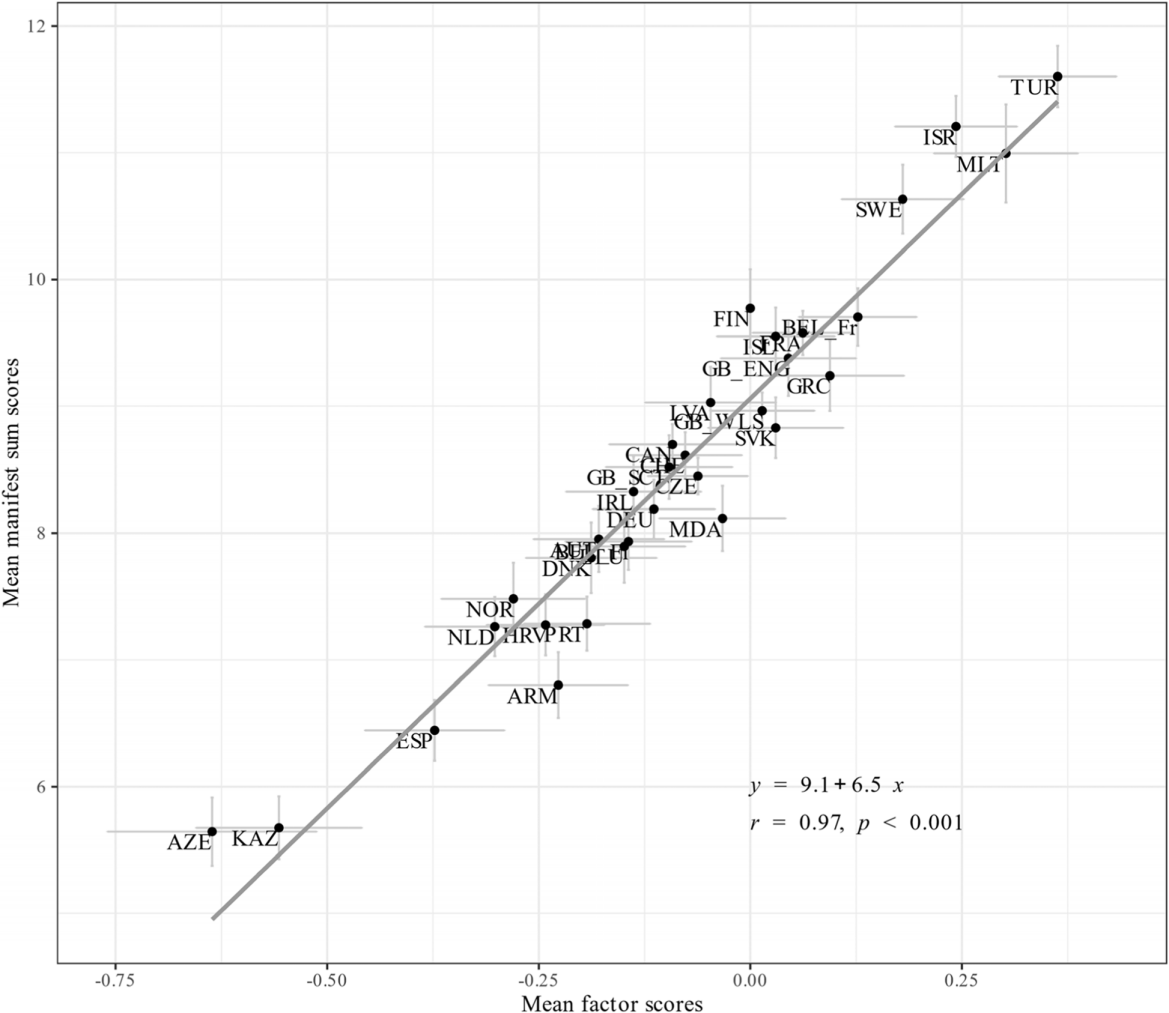
Scatterplot with means of factor scores and manifest sum scores. Notes. Factor scores were estimates via expected a-posterior (EAP) method. The regression equation and correlation coefficient are shown. The abbreviations of the country names can be found in Table 1

The correlations between factor scores and manifest sum scores ranged between 0.94 and 0.98 within each country (see Figure A97 in the Electronic supplement). However, the regression slopes showed quite some variation and ranged between 5.9 and 10 reflecting the different test characteristic curves. Figure 9 shows the association between the means of the manifest sum scores and the means of the factor scores with a correlation of 0.97.

## Discussion

With increasing policy and research interest in cross-national adolescent health, it is vital that research instruments collecting data on latent measures such as health complaints are proved to be valid and suitable for cross-national comparisons. Therefore, this paper focused on analyzing the psychometric properties of HBSC-SCL, including an examination of its factorial structure as well as testing measurement invariance and differential test and item functioning across countries. HBSC-SCL is one of the most used cross-national indicators of adolescent well-being, and has been featured by stakeholders such as the WHO Regional Office for Europe, UNICEF or OECD [64–66].

With respect to the dimensionality of the HBSC-SCL, results from this paper more definitely address the mixed findings cited from previous research, where studies both suggest a good functioning of a single factor solution across 35 countries [25] and comparatively better functioning of a two-correlated factor model [13, 16]. In the present study, EGA confirmed unidimensionality in only 16 out of 46 countries, but showed a two-dimensional structure (with both dimensions being correlated) in most of them. A closer inspection of the models revealed that items 4–6, i.e., feeling low, irritability/bad temper and feeling nervous, seemed to contribute to violations of the local independency assumption. In other words, beyond the scores in the latent variable, there are other factors that make responses to these items not independent. These items have been considered to be indicators of psychological complaints [13], and showed the highest factor loadings in research modelling such factor [14, 15], which may have to do with their tendency to cluster in some countries.

However, the comparison of a unidimensional GRM and a post-hoc bifactor GRM for countries that deviated from unidimensionality showed only minor differences between these models, with no indications of severe bias resulting from using a unidimensional model in most of these countries. Based on that, we conclude that HBSC-SCL can be considered unidimensional in around two thirds of the examined countries. Nevertheless, in some countries (such as Georgia, Italy, Poland, Ukraine, Greenland, and Albania) there is a need to control for specific factors. Given that none of the countries show high values for these specific factors in terms of the proportion of explained variance of the items (values of ECV-S1/ECV-S2 range between 0.0 to 0.29), the subscales are not interpretable after controlling for the general factor. Nevertheless, if analyses conducted within these respective countries did not control for the specific factors, this would result in the reliability of HBSC-SCL being overestimated. In addition, the person parameters would be biased in some of these countries, especially Georgia and Italy.

Measurement invariance analysis was carried out in 32 countries and 14 countries were excluded from this further analysis because they deviated to a greater extent from a one-dimensional structure. Multigroup invariance analyses supported configural and metric invariance indicating a similar factor structure and loadings across countries. Scalar invariance (setting item thresholds to be the same across countries) indicated that there were some non-invariant thresholds. The alignment analysis showed non-invariance especially for items (5) irritability, and (6) feeling nervous/bad temper. Item discrimination values were 0.456 and 0.000, corresponding with 62.5% and 53.1% of invariant groups, respectively. Also, item (4) feeling low showed non-invariant thresholds with discrimination value 0.316 and 31% of non-invariant groups. Results indicate that researchers need to be aware of measurement non-invariance, especially for items measuring psychological health complaints. In addition to translation issues that have been proposed as possible sources for country level non-invariance [15, 24] there might be cultural differences in experiencing and expressing psychological symptoms. Thus, adolescents with the same level of psychosomatic complaints might answer differently to items measuring these complaints. For instance, adolescents from England and from Armenia showed an average deviation of 0.85 (99% CI [0.64; 1.16]) points from the HBSC-SCL expected test scores (ranging between 0 and 32) for the same level of psychosomatic complaints. Adolescents from England yielded scores that were up to 1.4 points lower compared with adolescents from Armenia at the lower level of the latent variable. Although the invariance analyses indicated that approximately half of the parameters are non-invariant across countries, the correlation between the means of the manifest sum scores and the means of the alignment factor scores were quite high (*r* = 0.97). The high correlation is mainly driven by countries with extreme mean values (e.g. Azerbaijan and Turkey) and therefore does not mean that the scaling procedure does not play a role. The scaling procedure becomes highly relevant, when one wants to compare specific country pairs, for instance Norway and Armenia where the sum score mean differences indicated a significant difference between the countries whereas the alignment factor score mean differences indicated no difference. Thus, when comparing countries with lower differences, sum scores and factor scores can lead to different conclusions.

### Study strengths and limitations

One strength of the current study is the large number of countries included across Europe, Asia and North America as well as the large sample size for all included countries (i.e., n ranged between 1,002 and 15,328). In addition, the common survey protocol used by HBSC countries (e.g., translation and back translation procedures) contribute to the comparability of the measure used cross-nationally and the representativeness of these findings across countries and cultures [34]. Furthermore, the present study benefited from the use of sophisticated statistical methods, such as GRM, which is particularly suitable for instruments with ordered response categories [63] but to our knowledge had not been used before in the study of HBSC-SCL. Finally, in addition to providing valuable information for cross-national studies using HBSC-SCL, this study offered a great wealth of data about the scale functioning in each country.

Some limitations of the study must also be taken into consideration. For instance, the need for unidimensionality as a prerequisite for the invariance analyses conducted in the present study meant that we were able to include only 32 countries/regions out of the original 46. Future studies should focus their analysis on these countries in more detail, using a bi-factorial or two-factor structure to get additional information about the psychometric properties of this scale. Another limitation is that the results are only generalizable to the 46 participating countries, which are located in Europe, North America and parts of Asia. In order to be able to replicate the analyses conducted in other countries, we provide the necessary syntax.

### Conclusion and implications

HBSC-SCL is a reliable unidimensional instrument in most countries, showing considerable promise for etiological and population health research. Items measuring psychological health complaints show some non-invariance across countries and researchers should be aware that adolescents with the same latent trait level may answer differently due to cultural differences and difficulties in translation.

### Software information

Data analysis was done in R Version 4.1.0 [67] and Mplus v8.0 [68]. Data transformations were done with the tidyverse [69], car [70], labelled [71], and sjlabelled [72] packages. Descriptive statistics were calculated with the weights [73] and the Weighted.Desc.Stat [74] packages. Dimensionality assessment was done with the EFA.dimensions [75] package, the psych [76] package, the function in Lubbe [77] that was slightly modified to be able to include survey weights and the EGAnet [78] package. Item response analyses were done with the mirt [79] and irtplay [80] packages. The graphs were created with the ggplot2 [81] and ggpubr [82]. The alignment analysis was done in Mplus v8.0 and read in R with the package MplusAutomation [83].

## Supplementary Information

**Additional file 1:** **Table A1.** Sample size,percent females, mean, and standard deviation of age. **Table A2.** Distribution of the HBSC-SCL items (1-4). **Table A3.** Distribution of the HBSC-SCLitems (5-8). **Table A4. **Goodness offit statistics for the bifactor GRM. **Table A5.** Bifactor statistical indices. **Table A6.** Multigroup Model Fit. **Table A7.**Monte Carlo simulation results: Mean parameter stability. **Figure A1.** HBSC-SCL bar charts. **Figure A2. **HBSC-SCL polychoric correlations. **Figure A3.** Test for local dependency of the unidimensional GRM. **Figure A4.** Item fit statistics of theunidimensional GRM. **Figure A5. **Residualplots of the unidimensional GRM for ALB. **FigureA6.** Residual plots of the unidimensional GRM for ARM. **Figure A7. **Residual plots of the unidimensional GRM for AUT. **Figure A8.** Residual plots of theunidimensional GRM for AZE. **Figure A9. **Residual plots of the unidimensional GRM for BEL_FL. **Figure A10.** Residual plots of the unidimensional GRM for BEL_FR. **Figure A11.** Residual plots of theunidimensional GRM for BGR. **Figure A12. **Residual plots of the unidimensional GRM for CAN. **Figure A13.** Residual plots of the unidimensional GRM for CHE. **Figure A14.** Residual plots of theunidimensional GRM for CZE. **Figure A15. **Residual plots of the unidimensional GRM for DEU. **Figure A16.** Residual plots of the unidimensional GRM for DNK. **Figure A17.** Residual plots of theunidimensional GRM for ESP. **Figure A18. **Residual plots of the unidimensional GRM for EST. **Figure A19. **Residual plots of the unidimensional GRM for FIN. **Figure A20.** Residual plots of theunidimensional GRM for FRA. **Figure A21. **Residual plots of the unidimensional GRM for GB_ENG. **Figure A22.** Residual plots of the unidimensional GRM for GB_SCT. **Figure A23.** Residual plots of the unidimensionalGRM for GB_WLS. **Figure A24.** Residualplots of the unidimensional GRM for GEO. **FigureA25.** Residual plots of the unidimensional GRM for GRC. **Figure A26. **Residual plots of the unidimensional GRM for GRL. **Figure A27.** Residual plots of theunidimensional GRM for HRV. **Figure A28. **Residual plots of the unidimensional GRM for HUN. **Figure A29.** Residual plots of the unidimensional GRM for IRL. **Figure A30.** Residual plots of theunidimensional GRM for ISL. **Figure A31. **Residual plots of the unidimensional GRM for ISR. **Figure A32.** Residual plots of the unidimensional GRM for ITA. **Figure A33.** Residual plots of theunidimensional GRM for KAZ. **Figure A34. **Residual plots of the unidimensional GRM for LTU. **Figure A35.** Residual plots of the unidimensional GRM for LUX. **Figure A36.** Residual plots of theunidimensional GRM for LVA. **Figure A37. **Residual plots of the unidimensional GRM for MDA. **Figure A38.** Residual plots of the unidimensional GRM for MLT. **Figure A39.** Residual plots of theunidimensional GRM for NLD. **Figure A40. **Residual plots of the unidimensional GRM for NOR. **Figure A41. **Residual plots of the unidimensional GRM for POL. **Figure A42.** Residual plots of theunidimensional GRM for PRT. **Figure A43**. Residual plots ofthe unidimensional GRM for ROU. **Figure A44. **Residual plots of the unidimensional GRM for RUS. **Figure A45.** Residual plots of the unidimensional GRM for SRB. **Figure A46. **Residual plots of theunidimensional GRM for SVK. **Figure A47. **Residual plots of the unidimensional GRM for SVN. **Figure A48.** Residual plots of the unidimensional GRM for SWE. **Figure A49.** Residual plots of theunidimensional GRM for TUR. **Figure A50. **Residual plots of the unidimensional GRM for UKR. **Figure A51.** Item characteristic curves of the unidimensional GRM. **Figure A52.** Test characteristic curvesof the unidimensional GRM. **Figure A53. **Item information functions of the unidimensional GRM. **Figure A54.** Person-item map for the unidimensional GRM (ALB-DNK). **Figure A55.** Person-item map for theunidimensional GRM (ESP-HUN). **Figure A56.** Person-item map for the unidimensional GRM (IRL-NOR). **Figure A57.** Person-item map for theunidimensional GRM (POL-UKR). **Figure A58.** Comparing local dependency between items for the unidimensional andthe bifactor GRM. **Figure A59. **Comparing item fit between the unidimensional and the bifactor GRM. **Figure A60. **Comparing itemdiscrimination parameters between the unidimensional and the bifactor GRM. **Figure A61.** Comparing testcharacteristic curves between the unidimensional and the bifactor GRM. **Figure A62.** Comparing factor scoresbetween the unidimensional and the bifactor GRM. **Figure A63.** Comparing test information functions between theunidimensional and the bifactor GRM. **Figure A64. **Approximate (non-)invariant parameters across countries. **Figure A65.** Differential testfunctioning: Reference group: ARM. **Figure A66.** Differential test functioning: Reference group: AUT. **Figure A67.** Differential testfunctioning: Reference group: AZE. **Figure A68.** Differential test functioning: Reference group: BEL_FL. **Figure A69.** Differential testfunctioning: Reference group: BEL_FR. **Figure A70.** Differential test functioning: Reference group: CAN. **Figure A71.** Differential testfunctioning: Reference group: CHE. **Figure A72.** Differential test functioning: Reference group: CZE. **Figure A73.** Differential testfunctioning: Reference group: DEU. **Figure A74.** Differential test functioning: Reference group: DNK. **Figure A75.** Differential testfunctioning: Reference group: ESP. **Figure A76.** Differential test functioning: Reference group: FIN. **Figure A77. **Differential testfunctioning: Reference group: FRA. **Figure A78.** Differential test functioning: Reference group: GB_SCT. **Figure A79.** Differential test functioning:Reference group: GB_WLS. **Figure A80. **Differential test functioning: Reference group: GRC. **Figure A81. **Differential test functioning: Reference group: HRV. **Figure A82. **Differential test functioning:Reference group: IRL. **Figure A83. **Differential test functioning: Reference group: ISL. **Figure A84.** Differential test functioning: Reference group: ISR. **Figure A85.** Differential test functioning:Reference group: KAZ. **Figure A86. **Differential test functioning: Reference group: LTU. **Figure A87. **Differential test functioning: Reference group: LVA. **Figure A88. **Differential testfunctioning: Reference group: MDA. **Figure A89.** Differential test functioning: Reference group: MLT. **Figure A90.** Differential testfunctioning: Reference group: NLD. **Figure A91.** Differential test functioning: Reference group: NOR. **Figure A92.** Differential testfunctioning: Reference group: PRT. **Figure A93.** Differential test functioning: Reference group: SVK. **Figure A94.** Differential testfunctioning: Reference group: SWE. **Figure A95.** Differential test functioning: Reference group: TUR. **Figure A96.** Heatmap of *sDRF *and *uDRF*. **Figure A97.** Scatterplot with factorscores and manifest sum scores. **Figure A98.** Means of manifest sum scores and factor scores. **Figure A99.** Factor score distribution. **Figure A100.** Item and test information functions of the alignmentmodel.

## Data Availability

The dataset analysed will become available from October 2022 via the HBSC Data Management Centre (https://www.uib.no/en/hbscdata). Corresponding syntax can be obtained from https://osf.io/u4xzt/.

## Abbreviations

CFI: Comparative fit index
DIF: Differential item functioning
ECV: Explained common variance
ECV-G: Explained common variance—general factor
ECV-S: Explained common variance—specific factor
EGA: Exploratory graph analysis
FD: Factor determinacy
GPCM: Generalized partial credit model
GRM: Graded response model
H: Construct replicability
HBSC: Health Behaviour in School-aged Children
ICC: Item characteristic curves
IIF: Item information function
IRT: Item response theory
M: Mean
Max: Maximum
Min: Minimum
MLR: Robust maximum likelihood estimator
PCM: Partial credit model
PUC: Number of unique correlations
RMSEA: Root Mean Square Error of Approximation
SCL: Symptom Checklist
SD: Standard deviation
sDRF: Compensatory differential response functioning statistic
SRMR: Standardized Root Mean Square Residual
TCC: Test characteristic curves
TIF: Test information function
TLI: Tucker-Lewis Index
uDRF: Non-compensatory differential response functioning statistic

## Footnotes

1 This procedure bears the risk of overfitting with the consequence that some of the results may not cross-validate in future studies [48]. However, our goal was to identify a comparison (bifactor) model that accurately accounts for multidimensionality and not a model that will certainly replicate in future studies [17].

2 However, we did not include Bulgaria in these analyses as the evaluation of the unidimensional GRM already indicated that multidimensionality cannot be ignored in this case. As a control analysis, we also calculated an oblique two-factor solution (with WLSMV estimator) with the items (1) headache, (2) stomachache, (3) backache, (7) difficulties in getting to sleep, and (8) feeling dizzy loading on the first factor, and the items (4) feeling low, (5) irritability/bad temper, and (6) feeling nervous loading on the second factor for all 46 countries. Overall, the model fitted quite well (X2 = 8444.912, df = 19, p = .000, RMSEA = .044, CFI = .989, TLI = .984, SRMR = .017, whereas there are some differences across countries regarding model fit (i.e., RMSEA ranged between .031 and .131 (M = .057, SD = .020); CFI ranged between .894 and .995 (M = .983, SD = .015); TLI ranged between .843 and .992 (M = .974, SD = .022); SRMR ranged between .015 and .071 (M = .024, SD = .009). For the complete data set, the correlation between the two factors equals .792, whereas the correlation between the two factors range between .531 and .904 (M = .791, SD = .068) across countries.

3 We fully acknowledge that the chosen cutoff-point is arbitrary and that we applied the (arbitrary) dichotomization of *whether or not* undimensionality exists. However, to conduct differential test functioning/measurement invariance analyses we had to make the decision which countries exhibited configural invariance – a prerequisite for further differential test functioning/measurement invariance analyses.
