# Supplementary material for: Item response theory and differential test functioning analysis of the HBSC-Symptom-Checklist across 46 countries
Source: BMC Med Res Methodol. 2022 Sep 29;22:253. doi: 10.1186/s12874-022-01698-3 (PMC9520881; doi:10.1186/s12874-022-01698-3)
Supplement: Supplementary file 1 — Additional file 1: Table A1. Sample size,percent females, mean, and standard deviation of age. Table A2. Distribution of the HBSC-SCL items (1-4). Table A3. Distribution of the HBSC-SCLitems (5-8). Table A4. Goodness offit statistics for the bifactor GRM. Table A5. Bifactor statistical indices. Table A6. Multigroup Model Fit. Table A7.Monte Carlo simulation results: Mean parameter stability. Figure A1. HBSC-SCL bar charts. Figure A2. HBSC-SCL polychoric correlations. Figure A3. Test for local dependency of the unidimensional GRM. Figure A4. Item fit statistics of theunidimensional GRM. Figure A5. Residualplots of the unidimensional GRM for ALB. FigureA6. Residual plots of the unidimensional GRM for ARM. Figure A7. Residual plots of the unidimensional GRM for AUT. Figure A8. Residual plots of theunidimensional GRM for AZE. Figure A9. Residual plots of the unidimensional GRM for BEL_FL. Figure A10. Residual plots of the unidimensional GRM for BEL_FR. Figure A11. Residual plots of theunidimensional GRM for BGR. Figure A12. Residual plots of the unidimensional GRM for CAN. Figure A13. Residual plots of the unidimensional GRM for CHE. Figure A14. Residual plots of theunidimensional GRM for CZE. Figure A15. Residual plots of the unidimensional GRM for DEU. Figure A16. Residual plots of the unidimensional GRM for DNK. Figure A17. Residual plots of theunidimensional GRM for ESP. Figure A18. Residual plots of the unidimensional GRM for EST. Figure A19. Residual plots of the unidimensional GRM for FIN. Figure A20. Residual plots of theunidimensional GRM for FRA. Figure A21. Residual plots of the unidimensional GRM for GB_ENG. Figure A22. Residual plots of the unidimensional GRM for GB_SCT. Figure A23. Residual plots of the unidimensionalGRM for GB_WLS. Figure A24. Residualplots of the unidimensional GRM for GEO. FigureA25. Residual plots of the unidimensional GRM for GRC. Figure A26. Residual plots of the unidimensional GRM for GRL. Figure A27. Residual plots of theunidimensi [file 12874_2022_1698_MOESM1_ESM.pdf]

ELECTRONIC SUPPLEMENT FOR:

Item response theory and differential test functioning analysis of the HBSC-Symptom-Checklist across 46 countries

Andreas Heinz[1*], Philipp E. Sischka[2*], Carolina Catunda, Alina Cosma, Irene García, Nelli Lyyra, Anne Kaman, Ulrike Ravens-Sieberer, & William L. Pickett

Abbreviation of country names

ALB	Albania
ARM	Armenia
AUT	Austria
AZE	Azerbaijan
BEL-FL	Belgium (Flemish)
BEL-FR	Belgium (French)
BGR	Bulgaria
CAN	Canada
CHE	Switzerland
CZE	Czechia
DEU	Germany
DNK	Denmark
EST	Estonia
ESP	Spain
FIN	Finland
FRA	France
GB-ENG	England
GB-SCT	Scotland
GB-WLS	Wales
GEO	Georgia
GRL	Greenland
GRC	Greece
HRV	Croatia
HUN	Hungary
IRL	Ireland
ISR	Israel
ISL	Iceland
ITA	Italy
KAZ	Kazakhstan
LTU	Lithuania
LUX	Luxembourg
LVA	Latvia
MDA	Republic of Moldova
MLT	Malta
NLD	Netherlands
NOR	Norway
POL	Poland
PRT	Portugal
ROU	Romania
RUS	Russian Federation
SRB	Serbia
SWE	Sweden
SVN	Slovenia
SVK	Slovakia
TUR	Turkey
UKR	Ukraine

Content

Table A1. Sample size, percent females, mean, and standard deviation of age. 6
Table A2. Distribution of the HBSC-SCL items (1-4). 7
Table A3. Distribution of the HBSC-SCL items (5-8). 8
Table A4. Goodness of fit statistics for the bifactor GRM. 9
Table A5. Bifactor statistical indices. 10
Table A6. Multigroup Model Fit. 11
Table A7. Monte Carlo simulation results: Mean parameter stability............................. 12

Figure A1. HBSC-SCL bar charts.. 13
Figure A2. HBSC-SCL polychoric correlations. 14
Figure A3. Test for local dependency of the unidimensional GRM. 15
Figure A4. Item fit statistics of the unidimensional GRM. 16
Figure A5. Residual plots of the unidimensional GRM for ALB. 17
Figure A6. Residual plots of the unidimensional GRM for ARM. 18
Figure A7. Residual plots of the unidimensional GRM for AUT. 19
Figure A8. Residual plots of the unidimensional GRM for AZE. 20
Figure A9. Residual plots of the unidimensional GRM for BEL_FL. 21
Figure A10. Residual plots of the unidimensional GRM for BEL_FR. 22
Figure A11. Residual plots of the unidimensional GRM for BGR. 23
Figure A12. Residual plots of the unidimensional GRM for CAN. 24
Figure A13. Residual plots of the unidimensional GRM for CHE. 25
Figure A14. Residual plots of the unidimensional GRM for CZE. 26
Figure A15. Residual plots of the unidimensional GRM for DEU. 27
Figure A16. Residual plots of the unidimensional GRM for DNK. 28
Figure A17. Residual plots of the unidimensional GRM for ESP. 29
Figure A18. Residual plots of the unidimensional GRM for EST. 30
Figure A19. Residual plots of the unidimensional GRM for FIN. 31
Figure A20. Residual plots of the unidimensional GRM for FRA. 32
Figure A21. Residual plots of the unidimensional GRM for GB_ENG. 33
Figure A22. Residual plots of the unidimensional GRM for GB_SCT. 34
Figure A23. Residual plots of the unidimensional GRM for GB_WLS. 35
Figure A24. Residual plots of the unidimensional GRM for GEO. 36
Figure A25. Residual plots of the unidimensional GRM for GRC. 37
Figure A26. Residual plots of the unidimensional GRM for GRL. 38
Figure A27. Residual plots of the unidimensional GRM for HRV. 39
Figure A28. Residual plots of the unidimensional GRM for HUN. 40
Figure A29. Residual plots of the unidimensional GRM for IRL. 41
Figure A30. Residual plots of the unidimensional GRM for ISL. 42
Figure A31. Residual plots of the unidimensional GRM for ISR. 43
Figure A32. Residual plots of the unidimensional GRM for ITA. 44
Figure A33. Residual plots of the unidimensional GRM for KAZ. 45
Figure A34. Residual plots of the unidimensional GRM for LTU. 46
Figure A35. Residual plots of the unidimensional GRM for LUX. 47
Figure A36. Residual plots of the unidimensional GRM for LVA. 48
Figure A37. Residual plots of the unidimensional GRM for MDA. 49
Figure A38. Residual plots of the unidimensional GRM for MLT. 50
Figure A39. Residual plots of the unidimensional GRM for NLD. 51

Figure A40. Residual plots of the unidimensional GRM for NOR. 52
Figure A41. Residual plots of the unidimensional GRM for POL. 53
Figure A42. Residual plots of the unidimensional GRM for PRT. 54
Figure A43. Residual plots of the unidimensional GRM for ROU. 55
Figure A44. Residual plots of the unidimensional GRM for RUS. 56
Figure A45. Residual plots of the unidimensional GRM for SRB. 57
Figure A46. Residual plots of the unidimensional GRM for SVK. 58
Figure A47. Residual plots of the unidimensional GRM for SVN. 59
Figure A48. Residual plots of the unidimensional GRM for SWE. 60
Figure A49. Residual plots of the unidimensional GRM for TUR. 61
Figure A50. Residual plots of the unidimensional GRM for UKR. 62
Figure A51. Item characteristic curves of the unidimensional GRM. 63
Figure A52. Test characteristic curves of the unidimensional GRM. 64
Figure A53. Item information functions of the unidimensional GRM. 65
Figure A54. Person-item map for the unidimensional GRM (ALB-DNK). 66
Figure A55. Person-item map for the unidimensional GRM (ESP-HUN). 67
Figure A56. Person-item map for the unidimensional GRM (IRL-NOR). 68
Figure A57. Person-item map for the unidimensional GRM (POL-UKR). 69
Figure A58. Comparing local dependency between items for the unidimensional and the bifactor GRM. 70
Figure A59. Comparing item fit between the unidimensional and the bifactor GRM. 71
Figure A60. Comparing item discrimination parameters between the unidimensional and the bifactor GRM. 72
Figure A61. Comparing test characteristic curves between the unidimensional and the bifactor GRM. 73
Figure A62. Comparing factor scores between the unidimensional and the bifactor GRM. 74
Figure A63. Comparing test information functions between the unidimensional and the bifactor GRM. 75
Figure A64. Approximate (non-)invariant parameters across countries. 76
Figure A65. Differential test functioning: Reference group: ARM. 77
Figure A66. Differential test functioning: Reference group: AUT. 78
Figure A67. Differential test functioning: Reference group: AZE. 79
Figure A68. Differential test functioning: Reference group: BEL_FL. 80
Figure A69. Differential test functioning: Reference group: BEL_FR. 81
Figure A70. Differential test functioning: Reference group: CAN. 82
Figure A71. Differential test functioning: Reference group: CHE. 83
Figure A72. Differential test functioning: Reference group: CZE. 84
Figure A73. Differential test functioning: Reference group: DEU. 85
Figure A74. Differential test functioning: Reference group: DNK. 86
Figure A75. Differential test functioning: Reference group: ESP. 87
Figure A76. Differential test functioning: Reference group: FIN. 88
Figure A77. Differential test functioning: Reference group: FRA. 89
Figure A78. Differential test functioning: Reference group: GB_SCT. 90
Figure A79. Differential test functioning: Reference group: GB_WLS. 91
Figure A80. Differential test functioning: Reference group: GRC. 92
Figure A81. Differential test functioning: Reference group: HRV. 93
Figure A82. Differential test functioning: Reference group: IRL. 94
Figure A83. Differential test functioning: Reference group: ISL. 95
Figure A84. Differential test functioning: Reference group: ISR. 96

Figure A85. Differential test functioning: Reference group: KAZ. 97
Figure A86. Differential test functioning: Reference group: LTU. 98
Figure A87. Differential test functioning: Reference group: LVA. 99
Figure A88. Differential test functioning: Reference group: MDA. 100
Figure A89. Differential test functioning: Reference group: MLT. 101
Figure A90. Differential test functioning: Reference group: NLD. 102
Figure A91. Differential test functioning: Reference group: NOR. 103
Figure A92. Differential test functioning: Reference group: PRT. 104
Figure A93. Differential test functioning: Reference group: SVK. 105
Figure A94. Differential test functioning: Reference group: SWE. 106
Figure A95. Differential test functioning: Reference group: TUR. 107
Figure A96. Heatmap of *sDRF* and *uDRF*. 108
Figure A97. Scatterplot with factor scores and manifest sum scores. 109
Figure A98. Means of manifest sum scores and factor scores. 110
Figure A99. Factor score distribution. 111
Figure A100. Conditional and marginal reliabilities of the alignment model (partial invariance). 112

Tables

Table A1. Sample size, percent females, mean, and standard deviation of age.

Country	*N*	% female	Age *M* (*SD*)
ALB	1670	55.4	13.6 (1.5)
ARM	3894	51.7	13.6 (1.6)
AUT	4034	50.9	13.3 (1.6)
AZE	4338	51.5	13.3 (1.7)
BEL_FL	4247	50.8	13.4 (1.7)
BEL_FR	5352	50.6	13.3 (1.7)
BGR	4548	51.6	13.5 (1.6)
CAN	12301	53.2	13.7 (1.5)
CHE	7375	49.9	13.4 (1.6)
CZE	10858	50.6	13.4 (1.7)
DEU	4299	53.1	13.4 (1.7)
DNK	3093	52.2	13.3 (1.6)
ESP	4248	51.8	13.6 (1.6)
EST	4679	49.9	13.8 (1.6)
FIN	3113	50.8	13.8 (1.6)
FRA	8729	51.4	13.3 (1.6)
GB_ENG	3261	49.1	13.4 (1.5)
GB_SCT	4891	52.1	13.5 (1.6)
GB_WLS	15328	50.3	13.7 (1.7)
GEO	3736	52.0	13.5 (1.7)
GRC	3783	50.3	13.8 (1.7)
GRL	1002	54.1	13.2 (1.5)
HRV	4774	50.2	13.8 (1.7)
HUN	3674	53.2	13.5 (1.6)
IRL	3689	49.8	13.4 (1.6)
ISL	6815	50.4	13.6 (1.6)
ISR	7712	49.6	13.5 (1.6)
ITA	4099	52.0	13.7 (1.6)
KAZ	4439	50.1	13.3 (1.7)
LTU	3705	50.1	13.7 (1.7)
LUX	3979	50.1	13.5 (1.7)
LVA	4334	50.6	13.5 (1.6)
MDA	4455	50.4	13.6 (1.7)
MLT	2495	52.4	13.4 (1.6)
NLD	4658	48.8	13.5 (1.6)
NOR	3014	51.8	13.0 (1.6)
POL	5069	51.5	13.6 (1.7)
PRT	6039	52.2	13.3 (1.5)
ROU	4373	51.7	13.2 (1.6)
RUS	4154	52.6	13.8 (1.7)
SRB	3742	51.1	14.0 (1.7)
SVK	4427	49.0	13.3 (1.5)
SVN	5539	48.8	13.6 (1.6)
SWE	4009	51.0	13.7 (1.6)
TUR	5615	51.7	13.4 (1.7)
UKR	6318	49.2	13.4 (1.7)

Table A2. Distribution of the HBSC-SCL items (1-4).

Country	*I1*				*I2*				*I3*				*I4*			
	M	*SD*	*SK*	*K*	*M*	*SD*	*SK*	*K*	*M*	*SD*	*SK*	*K*	*M*	*SD*	*SK*	*K*
ALB	1.00	1.35	1.04	-0.36	0.48	0.96	2.23	4.24	0.60	1.09	1.91	2.68	0.95	1.33	1.19	0.04
ARM	0.81	1.29	1.27	0.07	0.44	0.99	2.31	4.20	0.58	1.12	1.91	2.38	1.28	1.45	0.66	-1.07
AUT	0.98	1.26	1.07	-0.09	0.80	1.09	1.44	1.33	0.75	1.16	1.52	1.27	0.84	1.20	1.31	0.59
AZE	0.87	1.40	1.23	-0.15	0.58	1.16	1.90	2.22	0.43	1.05	2.46	4.79	0.89	1.38	1.18	-0.22
BEL_FL	0.83	1.10	1.31	0.89	0.79	0.99	1.44	1.76	0.72	1.15	1.63	1.62	0.91	1.18	1.20	0.39
BEL_FR	1.03	1.20	0.97	-0.11	1.08	1.12	0.93	0.05	0.95	1.33	1.15	-0.06	1.18	1.34	0.83	-0.61
BGR	0.92	1.33	1.18	-0.01	0.86	1.16	1.36	0.85	0.70	1.23	1.65	1.39	0.82	1.30	1.39	0.54
CAN	1.07	1.22	0.95	-0.18	0.84	1.05	1.32	1.16	0.85	1.22	1.33	0.59	0.98	1.31	1.13	-0.02
CHE	1.02	1.15	0.98	0.02	0.94	1.02	1.10	0.68	0.87	1.19	1.27	0.56	1.09	1.22	0.90	-0.28
CZE	0.92	1.10	1.11	0.38	0.57	0.91	1.84	3.12	0.91	1.24	1.23	0.35	1.08	1.27	0.98	-0.22
DEU	1.09	1.15	0.95	0.06	0.91	1.05	1.21	0.94	0.94	1.21	1.19	0.40	0.88	1.17	1.25	0.56
DNK	0.90	1.17	1.13	0.22	0.63	0.95	1.67	2.31	0.79	1.14	1.44	1.10	0.97	1.15	1.09	0.29
ESP	0.72	1.11	1.58	1.55	0.54	0.91	2.00	3.80	0.80	1.23	1.50	1.06	0.74	1.15	1.55	1.34
EST	1.15	1.30	0.80	-0.65	0.79	1.05	1.36	1.08	0.75	1.18	1.52	1.19	1.35	1.43	0.66	-0.95
FIN	1.33	1.15	0.61	-0.43	0.97	0.98	1.00	0.65	0.90	1.13	1.18	0.49	1.24	1.26	0.70	-0.64
FRA	0.99	1.14	1.02	0.17	1.06	1.09	0.99	0.32	1.03	1.34	1.07	-0.18	0.98	1.25	1.10	0.05
GB_ENG	1.08	1.16	0.88	-0.18	0.87	1.00	1.20	0.96	0.68	1.09	1.60	1.63	1.36	1.35	0.65	-0.83
GB_SCT	1.00	1.21	0.99	-0.18	0.76	1.03	1.45	1.48	0.67	1.14	1.71	1.85	1.09	1.30	0.94	-0.36
GB_WLS	1.10	1.25	0.90	-0.32	0.82	1.05	1.38	1.28	0.72	1.17	1.60	1.43	1.21	1.37	0.82	-0.67
GEO	0.92	1.24	1.11	0.10	0.98	1.26	1.09	0.08	0.93	1.33	1.17	0.06	1.42	1.41	0.54	-1.02
GRC	1.00	1.29	1.04	-0.23	0.70	1.04	1.61	1.92	0.89	1.24	1.30	0.51	1.35	1.37	0.63	-0.89
GRL	1.14	1.45	0.82	-0.92	0.88	1.26	1.17	-0.06	0.66	1.24	1.72	1.53	1.17	1.43	0.86	-0.75
HRV	0.79	1.16	1.44	1.04	0.74	1.00	1.54	1.95	0.67	1.16	1.71	1.79	1.00	1.31	1.12	-0.01
HUN	1.31	1.26	0.66	-0.64	1.10	1.15	0.95	0.06	0.91	1.24	1.21	0.29	1.50	1.29	0.47	-0.90
IRL	1.04	1.18	0.95	-0.14	0.70	0.95	1.55	2.11	0.68	1.11	1.68	1.88	1.08	1.25	0.94	-0.27
ISL	1.16	1.26	0.83	-0.44	1.10	1.19	0.92	-0.12	0.99	1.28	1.09	-0.04	1.16	1.27	0.85	-0.41
ISR	1.42	1.39	0.58	-0.97	1.25	1.26	0.80	-0.43	1.04	1.35	1.06	-0.23	1.42	1.40	0.56	-1.01
ITA	1.40	1.31	0.51	-0.97	1.10	1.16	0.85	-0.29	1.02	1.29	1.04	-0.16	1.74	1.37	0.17	-1.26
KAZ	0.74	1.24	1.49	0.81	0.65	1.10	1.67	1.71	0.52	1.09	2.12	3.31	0.68	1.16	1.60	1.32
LTU	0.92	1.25	1.16	0.11	0.64	1.00	1.74	2.51	0.57	1.04	1.94	2.92	1.16	1.32	0.91	-0.39
LUX	1.03	1.17	0.95	-0.11	1.03	1.08	1.01	0.32	1.00	1.30	1.09	-0.10	1.25	1.29	0.69	-0.71
LVA	1.05	1.28	0.95	-0.35	0.82	1.08	1.34	1.00	0.82	1.24	1.39	0.69	1.09	1.35	0.95	-0.45
MDA	1.02	1.31	1.03	-0.27	0.79	1.17	1.44	0.97	0.91	1.31	1.26	0.26	1.34	1.43	0.62	-1.03
MLT	1.49	1.38	0.50	-1.03	1.07	1.20	1.00	0.02	1.13	1.34	0.94	-0.39	1.46	1.39	0.54	-1.01
NLD	0.94	1.23	1.16	0.17	0.58	0.98	1.88	2.95	0.66	1.10	1.74	2.12	0.68	1.11	1.62	1.62
NOR	0.74	1.07	1.43	1.18	0.86	1.04	1.26	1.01	0.53	1.01	2.05	3.41	0.94	1.16	1.12	0.27
POL	0.97	1.21	1.10	0.11	0.84	1.04	1.37	1.33	0.71	1.15	1.59	1.41	1.21	1.39	0.79	-0.76
PRT	0.74	1.19	1.53	1.14	0.41	0.87	2.48	6.01	0.93	1.31	1.24	0.22	1.04	1.33	1.05	-0.20
ROU	0.99	1.25	1.07	-0.04	0.71	1.04	1.60	1.92	0.98	1.34	1.15	-0.01	1.67	1.44	0.30	-1.28
RUS	1.10	1.31	0.88	-0.55	0.81	1.08	1.34	1.00	0.78	1.21	1.43	0.81	0.90	1.25	1.21	0.22
SRB	0.81	1.21	1.38	0.71	0.77	1.02	1.52	1.86	0.60	1.15	1.91	2.49	0.68	1.18	1.66	1.52
SVK	0.93	1.19	1.09	0.06	0.84	1.07	1.29	0.89	0.89	1.29	1.25	0.24	0.79	1.21	1.42	0.81
SVN	0.72	1.07	1.51	1.41	0.62	0.96	1.79	2.86	0.64	1.11	1.76	2.08	0.96	1.26	1.16	0.14
SWE	1.31	1.24	0.62	-0.69	1.17	1.16	0.83	-0.22	0.89	1.19	1.25	0.51	1.38	1.31	0.57	-0.86
TUR	1.15	1.34	0.82	-0.65	0.97	1.13	1.15	0.46	0.88	1.31	1.28	0.29	2.12	1.52	-0.13	-1.45
UKR	1.24	1.24	0.66	-0.69	0.98	1.06	0.98	0.20	0.93	1.22	1.14	0.15	1.03	1.28	0.98	-0.30

Notes. *M* = Mean; *SD* = Standard deviation; *SK* = Skewness; *K* = Kurtosis. Items (1) *headache*, (2) *stomachache*, (3) *backache*, (4) *feeling low*.

Table A3. Distribution of the HBSC-SCL items (5-8).

Country	*I5*				*I6*				*I7*				*I8*			
	M	*SD*	*SK*	*K*	*M*	*SD*	*SK*	*K*	*M*	*SD*	*SK*	*K*	*M*	*SD*	*SK*	*K*
ALB	1.39	1.43	0.63	-0.99	1.37	1.46	0.61	-1.09	0.65	1.24	1.74	1.59	0.48	1.02	2.28	4.21
ARM	1.47	1.54	0.45	-1.37	1.14	1.44	0.85	-0.80	0.70	1.30	1.63	1.09	0.40	0.98	2.51	5.17
AUT	1.46	1.29	0.52	-0.85	1.24	1.22	0.73	-0.46	1.28	1.45	0.74	-0.89	0.62	1.08	1.80	2.26
AZE	0.69	1.23	1.58	1.06	0.84	1.41	1.36	0.22	0.82	1.38	1.44	0.48	0.54	1.18	2.05	2.75
BEL_FL	1.34	1.19	0.62	-0.55	1.38	1.24	0.61	-0.64	1.36	1.53	0.65	-1.12	0.60	1.04	1.82	2.47
BEL_FR	1.42	1.31	0.50	-0.95	1.57	1.46	0.40	-1.23	1.77	1.64	0.20	-1.60	0.69	1.15	1.60	1.42
BGR	1.65	1.49	0.34	-1.33	1.87	1.54	0.11	-1.48	1.17	1.52	0.86	-0.87	0.61	1.17	1.85	2.17
CAN	1.24	1.24	0.74	-0.54	1.61	1.37	0.42	-1.07	1.38	1.48	0.63	-1.08	0.73	1.15	1.53	1.26
CHE	1.45	1.17	0.43	-0.74	1.21	1.17	0.70	-0.45	1.41	1.37	0.54	-1.00	0.63	1.04	1.70	2.05
CZE	1.68	1.25	0.34	-0.91	1.75	1.32	0.26	-1.07	1.22	1.46	0.80	-0.85	0.32	0.82	2.95	8.46
DEU	1.41	1.15	0.57	-0.48	0.92	1.07	1.10	0.52	1.37	1.37	0.65	-0.85	0.66	1.05	1.69	2.13
DNK	1.37	1.20	0.58	-0.62	1.14	1.16	0.80	-0.31	1.40	1.46	0.60	-1.08	0.61	1.04	1.76	2.24
ESP	1.05	1.29	1.04	-0.12	1.29	1.38	0.77	-0.72	0.80	1.28	1.47	0.80	0.52	1.00	2.10	3.61
EST	1.66	1.35	0.34	-1.09	1.48	1.40	0.50	-1.08	1.20	1.49	0.83	-0.86	0.91	1.31	1.22	0.11
FIN	1.65	1.18	0.25	-0.80	1.54	1.21	0.39	-0.81	1.35	1.33	0.59	-0.86	0.79	1.15	1.39	0.90
FRA	1.62	1.27	0.34	-0.96	1.54	1.41	0.46	-1.12	1.80	1.59	0.19	-1.54	0.56	1.05	1.95	2.81
GB_ENG	1.55	1.38	0.43	-1.10	1.53	1.38	0.47	-1.04	1.54	1.56	0.43	-1.37	0.76	1.15	1.47	1.06
GB_SCT	1.34	1.34	0.62	-0.87	1.44	1.35	0.59	-0.89	1.46	1.53	0.53	-1.26	0.76	1.19	1.47	1.00
GB_WLS	1.51	1.45	0.47	-1.19	1.38	1.38	0.62	-0.91	1.44	1.55	0.54	-1.28	0.79	1.20	1.40	0.74
GEO	1.32	1.38	0.64	-0.88	1.01	1.36	1.02	-0.31	1.00	1.37	1.04	-0.28	1.02	1.25	1.10	0.16
GRC	1.85	1.46	0.16	-1.36	1.80	1.52	0.19	-1.44	1.08	1.43	0.99	-0.51	0.56	1.06	1.97	2.91
GRL	1.18	1.40	0.81	-0.79	0.97	1.39	1.12	-0.27	1.76	1.70	0.24	-1.66	0.80	1.30	1.43	0.60
HRV	1.30	1.32	0.71	-0.68	1.53	1.41	0.46	-1.09	0.86	1.34	1.34	0.35	0.39	0.90	2.56	5.93
HUN	1.28	1.28	0.72	-0.61	1.69	1.33	0.28	-1.09	1.25	1.36	0.73	-0.78	0.68	1.12	1.66	1.79
IRL	1.38	1.30	0.59	-0.81	1.44	1.34	0.55	-0.90	1.31	1.46	0.70	-0.97	0.71	1.13	1.54	1.30
ISL	1.57	1.26	0.36	-0.94	1.36	1.36	0.64	-0.86	1.29	1.40	0.70	-0.88	0.92	1.24	1.20	0.27
ISR	1.98	1.43	0.00	-1.32	1.79	1.42	0.17	-1.28	1.35	1.50	0.66	-1.07	0.95	1.32	1.18	0.06
ITA	1.84	1.33	0.08	-1.20	2.09	1.34	-0.14	-1.18	1.23	1.46	0.77	-0.91	0.94	1.26	1.15	0.08
KAZ	1.06	1.35	0.98	-0.42	0.71	1.19	1.59	1.28	0.73	1.28	1.60	1.11	0.59	1.13	1.86	2.18
LTU	1.52	1.30	0.51	-0.85	1.40	1.36	0.60	-0.89	1.01	1.38	1.10	-0.20	0.67	1.13	1.68	1.77
LUX	1.66	1.18	0.22	-0.87	1.61	1.27	0.32	-0.96	1.41	1.40	0.54	-1.06	0.63	1.04	1.68	1.96
LVA	1.74	1.32	0.22	-1.11	1.33	1.36	0.63	-0.91	1.52	1.49	0.46	-1.26	0.66	1.15	1.71	1.77
MDA	1.38	1.40	0.58	-1.02	1.11	1.35	0.90	-0.54	0.87	1.36	1.32	0.24	0.70	1.17	1.63	1.46
MLT	1.82	1.43	0.14	-1.31	1.73	1.44	0.26	-1.27	1.37	1.52	0.62	-1.15	0.93	1.27	1.20	0.20
NLD	1.33	1.19	0.61	-0.58	1.12	1.16	0.84	-0.20	1.33	1.50	0.69	-1.03	0.62	1.09	1.80	2.21
NOR	1.39	1.15	0.58	-0.49	1.14	1.16	0.86	-0.12	1.25	1.39	0.79	-0.73	0.62	1.05	1.76	2.23
POL	1.71	1.35	0.30	-1.12	1.93	1.37	0.09	-1.22	1.20	1.51	0.83	-0.88	0.58	1.09	1.89	2.49
PRT	1.32	1.41	0.71	-0.85	1.41	1.42	0.62	-0.97	1.02	1.45	1.10	-0.35	0.42	0.96	2.46	5.15
ROU	1.55	1.48	0.42	-1.27	1.21	1.43	0.79	-0.82	1.12	1.49	0.93	-0.71	0.74	1.22	1.54	1.11
RUS	1.43	1.33	0.51	-0.97	1.20	1.35	0.79	-0.68	0.93	1.32	1.22	0.15	0.75	1.17	1.47	1.01
SRB	1.28	1.35	0.74	-0.72	1.75	1.49	0.27	-1.36	0.73	1.29	1.62	1.17	0.36	0.90	2.77	6.96
SVK	1.79	1.28	0.17	-1.07	1.80	1.37	0.16	-1.21	1.11	1.42	0.94	-0.61	0.67	1.13	1.63	1.56
SVN	1.33	1.24	0.70	-0.53	1.38	1.29	0.62	-0.73	1.19	1.37	0.83	-0.65	0.40	0.92	2.51	5.68
SWE	2.00	1.22	-0.05	-0.98	1.51	1.18	0.38	-0.79	1.58	1.43	0.36	-1.23	0.81	1.12	1.31	0.75
TUR	2.36	1.55	-0.37	-1.39	1.94	1.58	0.03	-1.56	1.33	1.59	0.68	-1.19	0.85	1.27	1.32	0.43
UKR	1.79	1.34	0.14	-1.18	1.80	1.40	0.12	-1.28	0.88	1.26	1.24	0.24	0.55	1.03	1.95	2.87

Notes. *M* = Mean; *SD* = Standard deviation; *SK* = Skewness; *K* = Kurtosis. Items (5) *irritability/bad temper*, (6) *nervousness*, (7) *difficulties in getting to sleep*, (8) *feeling dizzy*.

Table A4. Goodness of fit statistics for the bifactor GRM.

Country	C_2	*p*	*RMSEA* [90% CI]	*SRMR*	*TLI*	*CFI*
ALB	10.842	0.543	.000 [.000; .023]	.019	1.000	1.000
ARM	33.716	0.001	.022 [.013; .030]	.022	.995	.998
CHE	23.340	0.025	.011 [.004; .018]	.012	.999	1.000
DEU	21.377	0.045	.013 [.002; .023]	.019	.998	.999
DNK	28.089	0.005	.021 [.011; .031]	.016	.996	.998
EST	38.529	0.000	.022 [.014; .030]	.017	.997	.999
FIN	37.265	0.000	.026 [.017; .036]	.025	.996	.998
GB_SCT	44.841	0.000	.024 [.017; .031]	.022	.996	.998
GEO	98.880	0.000	.044 [.036; .052]	.058	.990	.996
GRC	36.641	0.000	.023 [.015; .032]	.015	.995	.998
GRL	29.420	0.003	.038 [.021; .056]	.031	.990	.996
HRV	15.649	0.208	.008 [.000; .018]	.016	1.000	1.000
HUN	33.971	0.001	.022 [.014; .031]	.017	.997	.999
ISL	43.800	0.000	.020 [.014; .026]	.012	.998	.999
ISR	111.231	0.000	.033 [.027; .038]	.031	.996	.998
ITA	50.810	0.000	.028 [.020; .036]	.018	.994	.997
LTU	50.670	0.000	.029 [.021; .038]	.023	.995	.998
LUX	23.749	0.022	.016 [.006; .025]	.015	.998	.999
LVA	69.604	0.000	.033 [.026; .041]	.017	.994	.997
MLT	39.491	0.000	.030 [.020; .041]	.023	.994	.997
NLD	41.306	0.000	.023 [.015; .031]	.015	.996	.998
POL	27.459	0.007	.016 [.008; .024]	.013	.998	.999
PRT	30.484	0.002	.016 [.009; .023]	.017	.998	.999
ROU	36.560	0.000	.022 [.014; .030]	.014	.996	.998
RUS	58.995	0.000	.031 [.023; .039]	.027	.995	.998
SRB	20.957	0.051	.014 [.000; .024]	.016	.998	.999
SVN	20.542	0.058	.011 [.000; .019]	.024	.999	1.000
TUR	44.014	0.000	.022 [.015; .029]	.018	.995	.998
UKR	53.988	0.000	.024 [.017; .030]	.015	.996	.998

Notes. *df* = 12; *RMSEA* = root mean squared error of approximation; *SRMR* = standardized root mean square residual; *TLI* = Tucker-Lewis index; *CFI* = comparative fit index.

Table A5. Bifactor statistical indices.

Country	ECV-G	ECV-S1	ECV-S2	PUC	H-G	H-S1	H-S2	FD-G	FD-S1	FD-S2
ALB	.76	.16	.08	.54	.88	.55	.34	.95	.86	.81
ARM	.67	.20	.13	.54	.83	.53	.42	.87	.71	.63
CHE	.70	.14	.16	.57	.82	.43	.47	.87	.65	.68
DEU	.74	.14	.12	.57	.82	.40	.36	.87	.65	.59
DNK	.74	.14	.12	.57	.83	.44	.36	.88	.68	.60
EST	.77	.07	.16	.54	.87	.27	.52	.92	.60	.80
FIN	.79	.08	.14	.54	.89	.31	.48	.92	.59	.76
GB_SCT	.81	.12	.07	.57	.86	.38	.27	.91	.63	.61
GEO	.71	.29	.00	.43	.93	.69	.00	.96	.87	.07
GRC	.65	.24	.11	.43	.83	.59	.38	.87	.76	.66
GRL	.73	.07	.19	.57	.88	.32	.58	.94	.75	.80
HRV	.74	.12	.15	.54	.86	.39	.48	.89	.63	.73
HUN	.77	.07	.16	.54	.88	.27	.54	.93	.61	.80
ISL	.82	.11	.07	.57	.89	.40	.28	.92	.71	.56
ISR	.76	.14	.10	.57	.91	.56	.42	.93	.90	.69
ITA	.64	.11	.25	.54	.82	.38	.68	.88	.67	.87
LTU	.77	.12	.11	.54	.88	.40	.39	.90	.65	.64
LUX	.74	.21	.05	.57	.82	.52	.17	.89	.74	.48
LVA	.82	.13	.05	.57	.88	.44	.20	.92	.69	.48
MLT	.77	.07	.16	.54	.87	.27	.53	.92	.60	.78
NLD	.81	.10	.09	.57	.86	.33	.30	.90	.59	.54
POL	.69	.10	.21	.54	.83	.36	.61	.88	.64	.85
PRT	.78	.06	.16	.54	.85	.23	.47	.91	.52	.72
ROU	.76	.06	.18	.54	.84	.20	.50	.91	.54	.74
RUS	.78	.07	.16	.54	.89	.27	.54	.93	.60	.81
SRB	.78	.16	.06	.54	.87	.52	.23	.93	.82	.63
SVN	.76	.08	.16	.54	.86	.28	.51	.91	.59	.78
TUR	.69	.18	.13	.54	.82	.47	.39	.86	.66	.59
UKR	.73	.08	.19	.54	.85	.31	.59	.91	.63	.84

Notes. ECV-G, ECV-S1, and ECV-S2 = Explained common variance of the general factor and of the two domain specific factors; PUC = Percent uncontaminated correlations; H-G, H-S1, and H-S2 = construct reliability of the general and the two domain specific factors; FD-G, FD-S1, and FD-S2 = factor determinacy of the general and the two domain specific factors.

Table A6. Multigroup Model Fit.

	Configural	Metric	Scalar
C_2	27563.346	30062.355	102507.217
df	640	888	1880
p	.000	.000	.000
RMSEA	.016 [.015; .016]	.014 [.014; .014]	.018 [.017; .018]
TLI	.941	.954	.925
CFI	.958	.955	.843
SRMR			
ARM	.081	.077	.146
AUT	.033	.058	.065
AZE	.062	.082	.181
BEL_Fl	.048	.056	.060
BEL_Fr	.055	.059	.061
CAN	.044	.056	.071
CHE	.064	.064	.091
CZE	.046	.042	.059
DEU	.054	.064	.099
DNK	.055	.055	.074
ESP	.043	.046	.050
FIN	.062	.063	.122
FRA	.045	.046	.049
GB_ENG	.043	.048	.053
GB_SCT	.048	.055	.058
GB_WLS	.040	.050	.053
GRC	.072	.072	.080
HRV	.075	.063	.066
IRL	.044	.054	.068
ISL	.045	.053	.081
ISR	.084	.085	.081
KAZ	.040	.047	.089
LTU	.070	.063	.075
LVA	.049	.048	.053
MDA	.052	.065	.084
MLT	.065	.069	.067
NLD	.042	.052	.063
NOR	.048	.052	.092
PRT	.054	.052	.061
SVK	.045	.057	.061
SWE	.043	.047	.095
TUR	.066	.070	.111

Table A7. Monte Carlo simulation results: Mean parameter stability.

Country	Population	Average	Standard deviation	Mean square error	95% CI coverage
ARM	-0.227	-0.236	0.026	0.001	0.940
AUT	-0.179	-0.185	0.027	0.001	0.952
AZE	-0.636	-0.635	0.039	0.002	0.956
BEL_Fl	-0.144	-0.152	0.024	0.001	0.944
BEL_Fr	0.127	0.120	0.023	0.001	0.962
CAN	-0.092	-0.096	0.022	0.000	0.952
CHE	-0.077	-0.081	0.021	0.000	0.964
CZE	-0.062	-0.066	0.019	0.000	0.960
DEU	-0.114	-0.119	0.024	0.001	0.954
DNK	-0.188	-0.194	0.026	0.001	0.962
ESP	-0.373	-0.380	0.027	0.001	0.950
FRA	0.062	0.056	0.019	0.000	0.934
GB_ENG	0.045	0.040	0.025	0.001	0.962
GB_SCT	-0.096	-0.101	0.024	0.001	0.954
GB_WLS	0.014	0.007	0.020	0.000	0.946
GRC	0.094	0.088	0.025	0.001	0.952
HRV	-0.242	-0.248	0.022	0.000	0.976
IRL	-0.138	-0.144	0.026	0.001	0.962
ISL	0.030	0.023	0.023	0.001	0.958
ISR	0.243	0.235	0.024	0.001	0.942
KAZ	-0.557	-0.559	0.030	0.001	0.962
LTU	-0.149	-0.158	0.026	0.001	0.938
LVA	-0.047	-0.052	0.025	0.001	0.966
MDA	-0.033	-0.042	0.024	0.001	0.934
MLT	0.302	0.290	0.028	0.001	0.940
NLD	-0.302	-0.310	0.026	0.001	0.974
NOR	-0.280	-0.287	0.028	0.001	0.948
PRT	-0.193	-0.198	0.023	0.001	0.956
SVK	0.030	0.023	0.025	0.001	0.958
SWE	0.180	0.173	0.025	0.001	0.952
TUR	0.363	0.358	0.023	0.001	0.950

Notes. Population = mean estimation from the alignment procedure; Average = average of the mean parameter estimates across replications; Standard deviation = standard deviation of the parameter estimates across replications; Mean square error = Mean square error for each parameter; 95% CI coverage = proportion of replications for which the 95% confidence interval contains the mean estimation from the alignment procedure

Figures

Figure A1. HBSC-SCL bar charts.

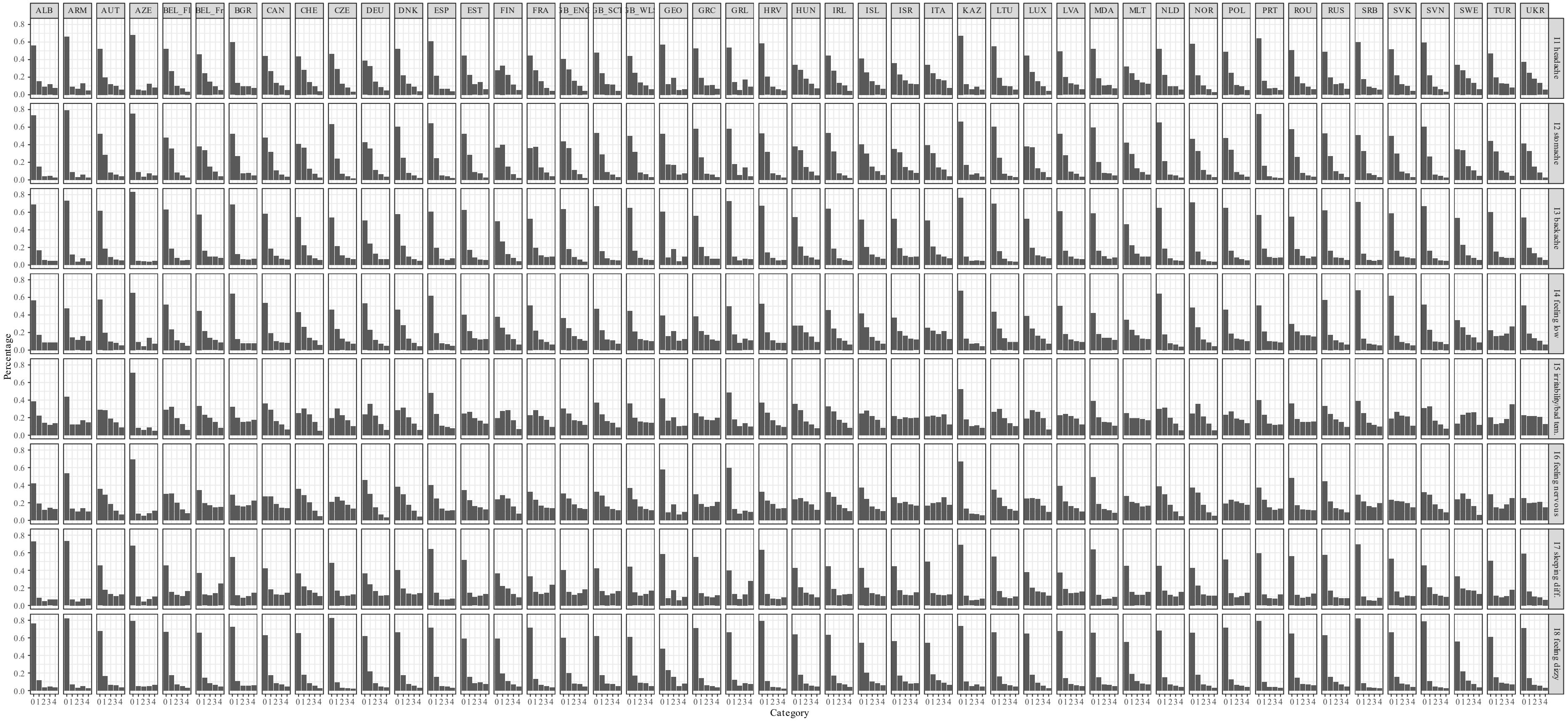

Figure A2. HBSC-SCL polychoric correlations.

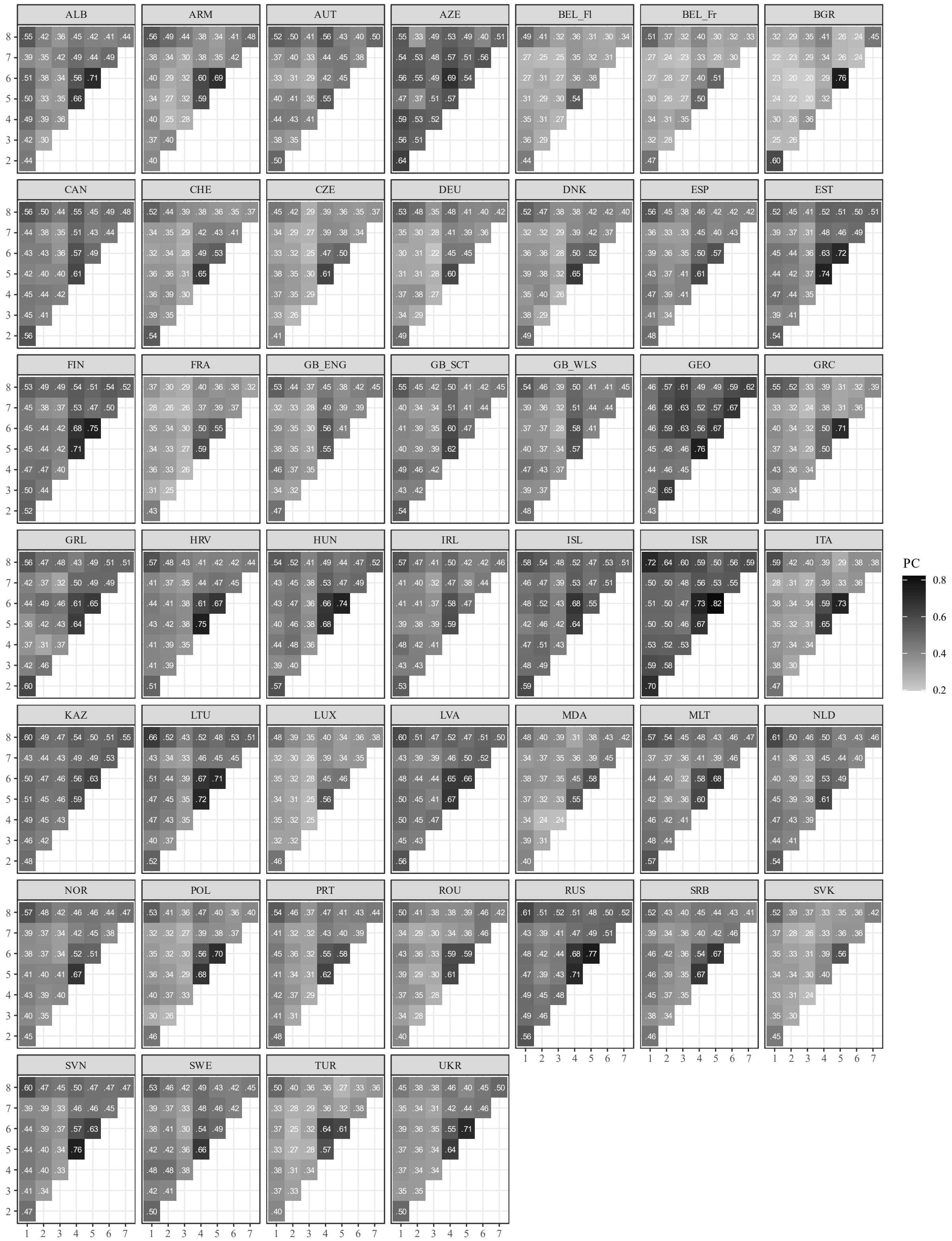

Notes. Items (1) *headache*, (2) *stomachache*, (3) *backache*, (4) *feeling low*, (5) *irritability/bad temper*, (6) *feeling nervous*, (7) *difficulties in getting to sleep*, (8) *feeling dizzy*.

Figure A3. Test for local dependency of the unidimensional GRM.

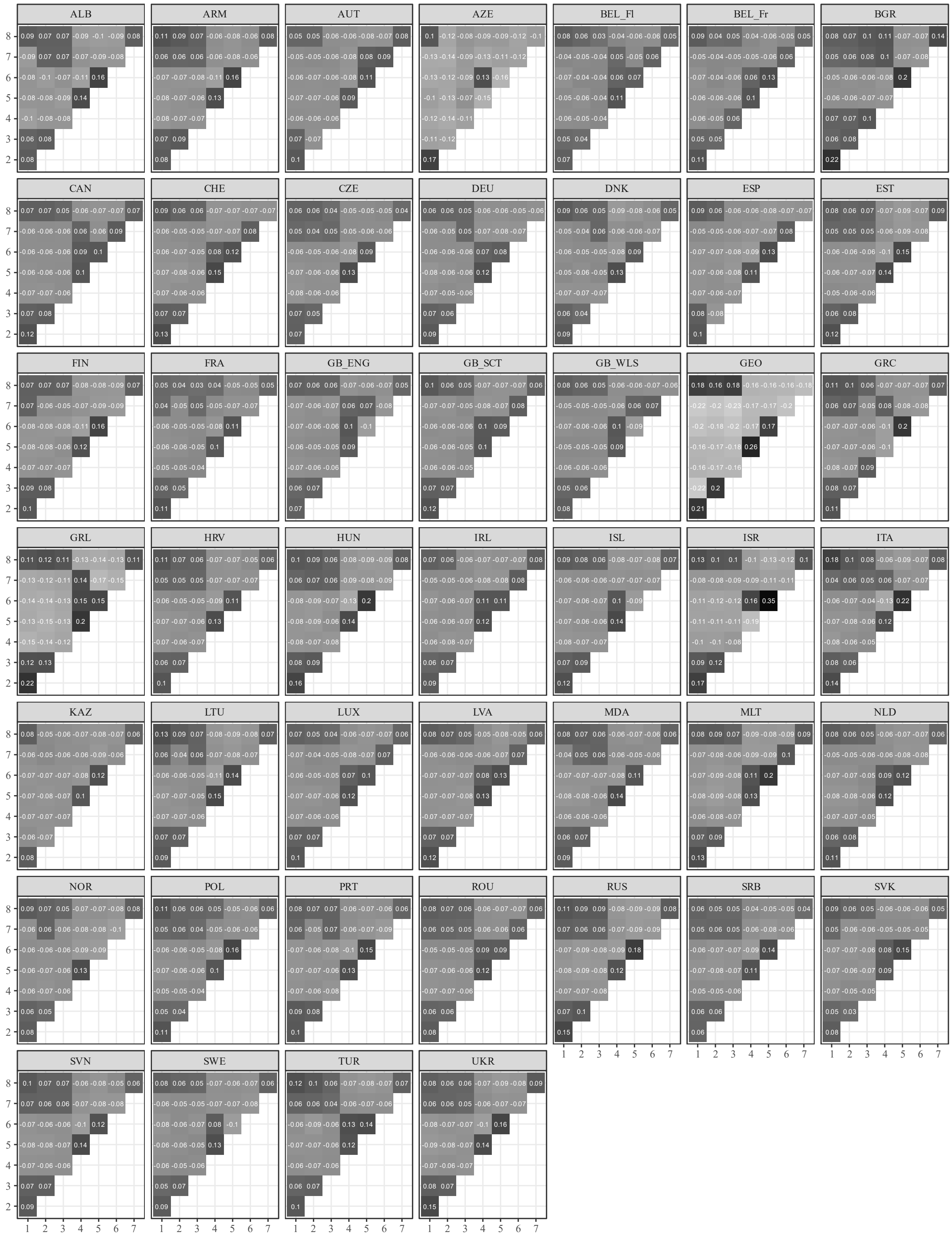

Notes. Values represent standardized residuals between items in terms of Cramer's *V*. Items (1) *headache*, (2) *stomachache*, (3) *backache*, (4) *feeling low*, (5) *irritability/bad temper*, (6) *feeling nervous*, (7) *difficulties in getting to sleep*, (8) *feeling dizzy*.

Figure A4. Item fit statistics of the unidimensional GRM.

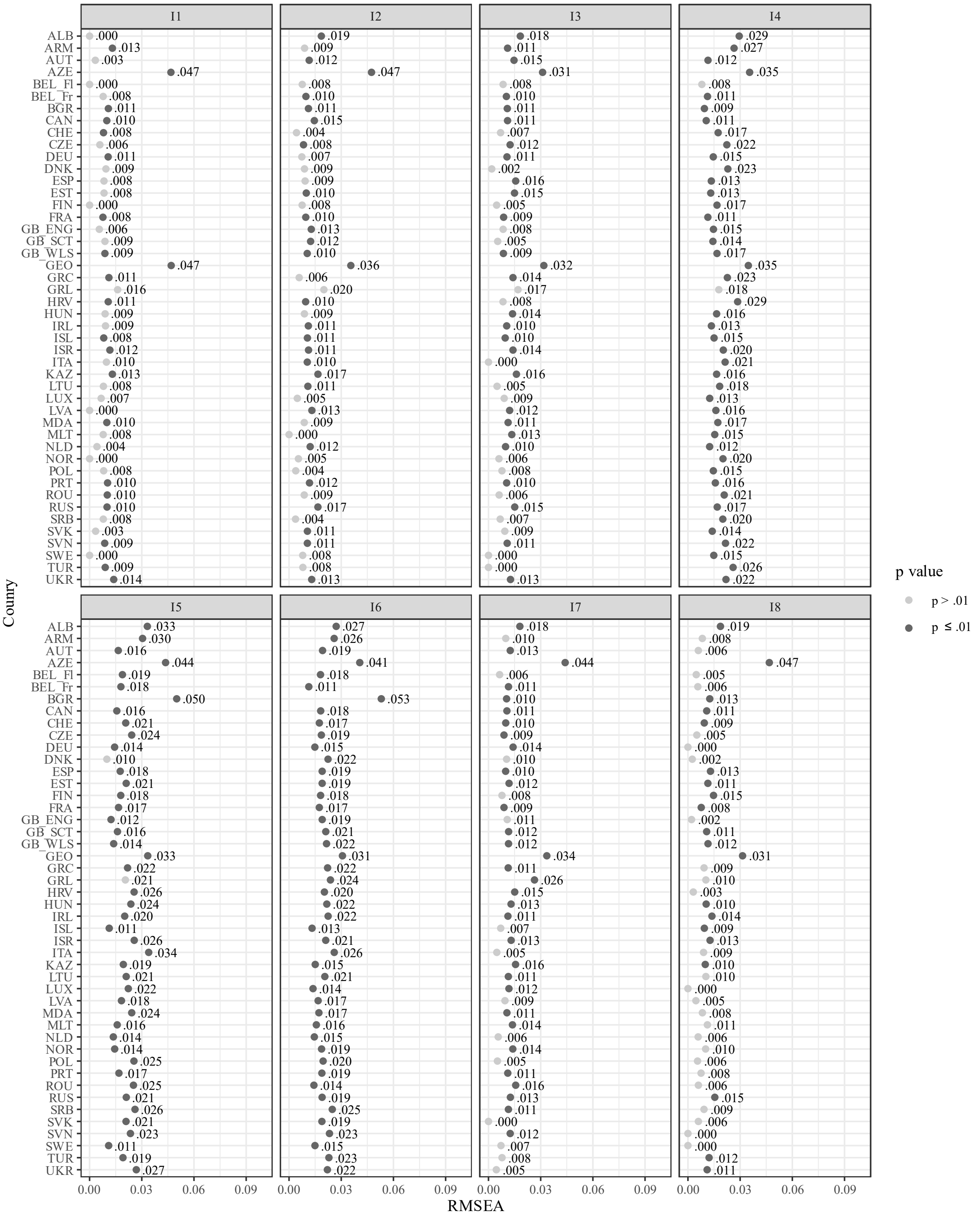

Notes. Values represent the item-level *RMSEA*. The significance test is based on the generalized S-X² statistic for polytomous items with adjusted p values based on the Benjamini-Hochberg (B-H) procedure. Items (1) *headache*, (2) *stomachache*, (3) *backache*, (4) *feeling low*, (5) *irritability/bad temper*, (6) *feeling nervous*, (7) *difficulties in getting to sleep*, (8) *feeling dizzy*.

Figure A5. Residual plots of the unidimensional GRM for ALB.

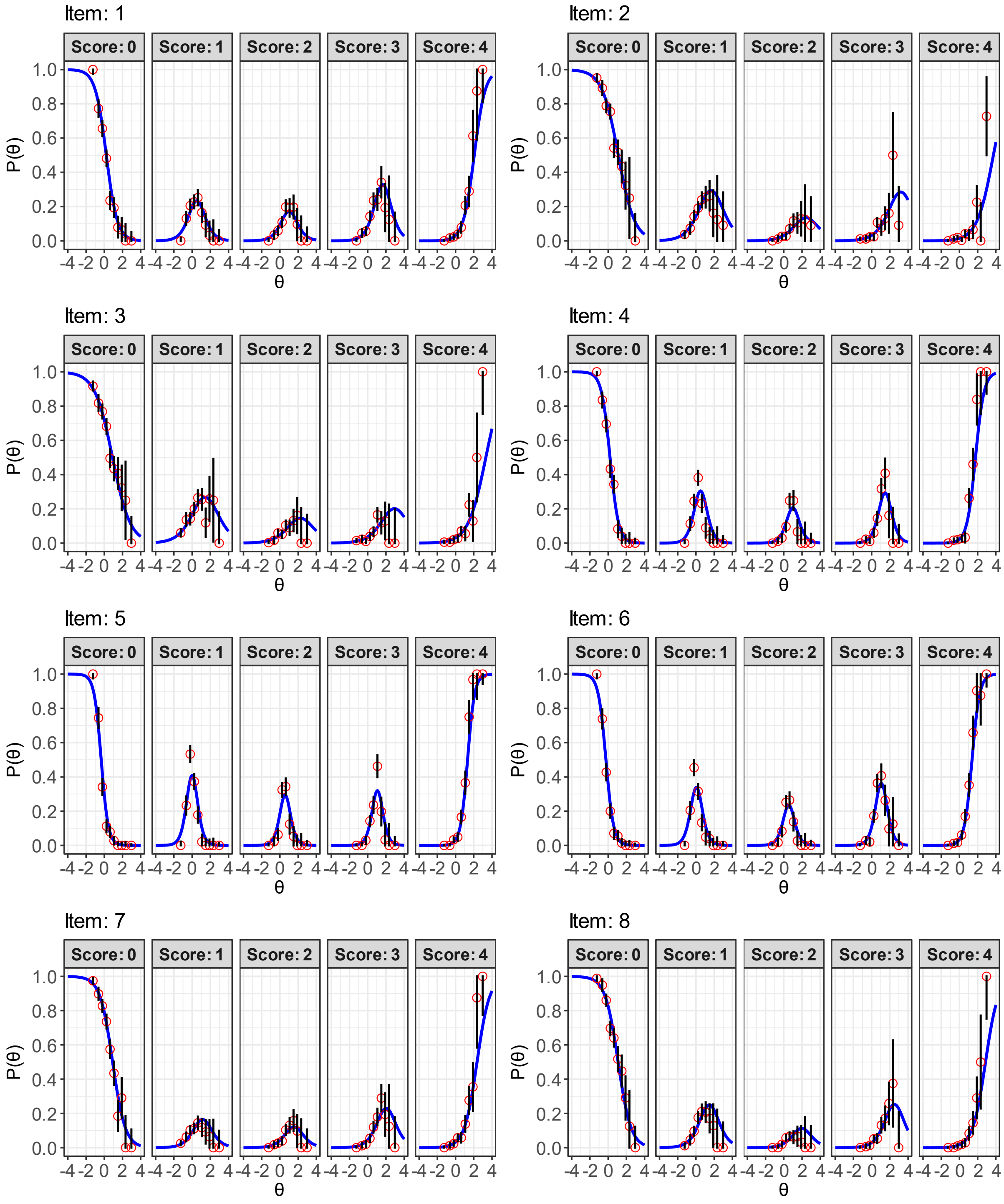

Notes. Response function and observed proportions with 95% CI. Items (1) *headache*, (2) *stomachache*, (3) *backache*, (4) *feeling low*, (5) *irritability/bad temper*, (6) *feeling nervous*, (7) *difficulties in getting to sleep*, (8) *feeling dizzy*.

Figure A6. Residual plots of the unidimensional GRM for ARM.

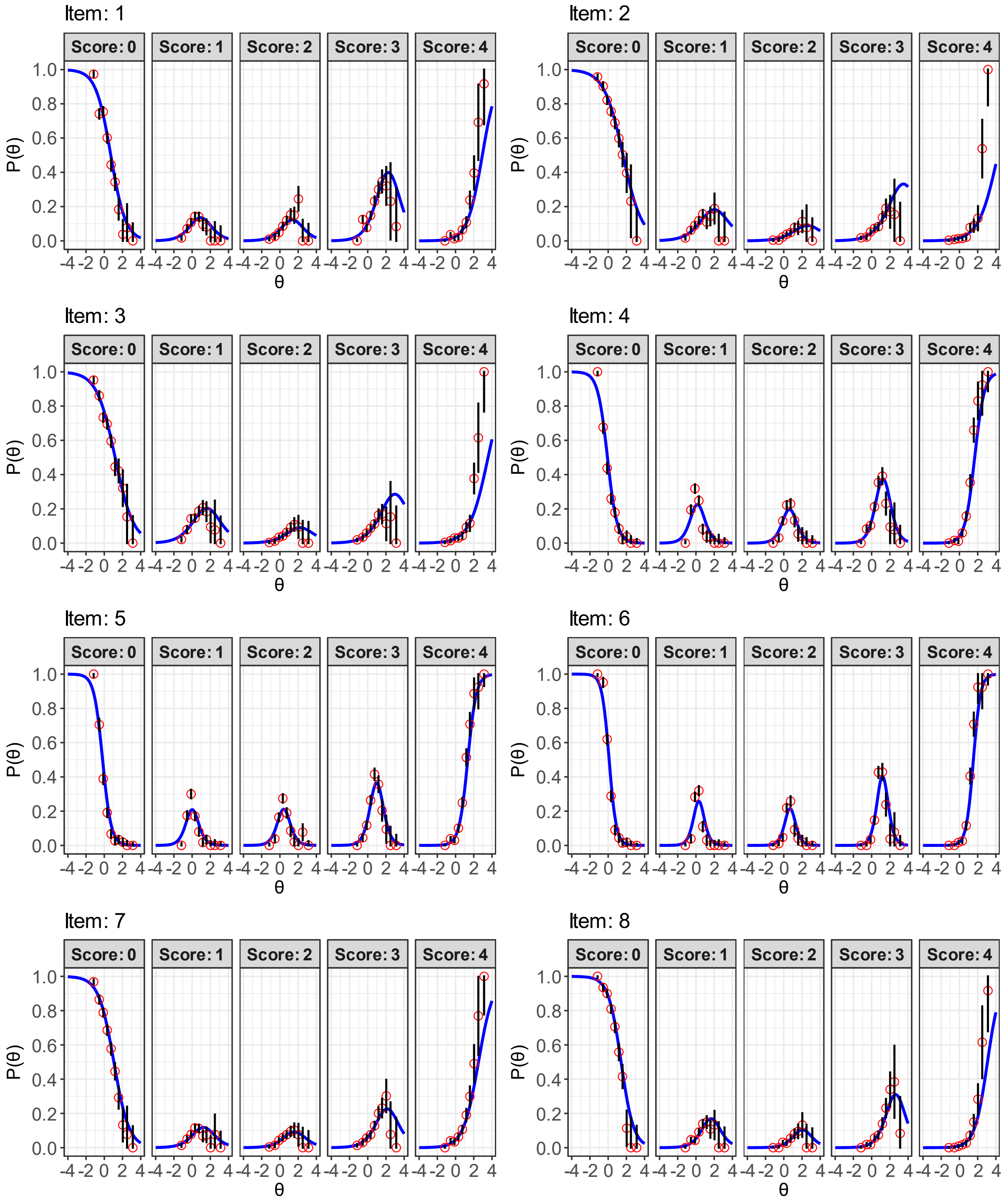

Notes. Response function and observed proportions with 95% CI. Items (1) *headache*, (2) *stomachache*, (3) *backache*, (4) *feeling low*, (5) *irritability/bad temper*, (6) *feeling nervous*, (7) *difficulties in getting to sleep*, (8) *feeling dizzy*.

Figure A7. Residual plots of the unidimensional GRM for AUT.

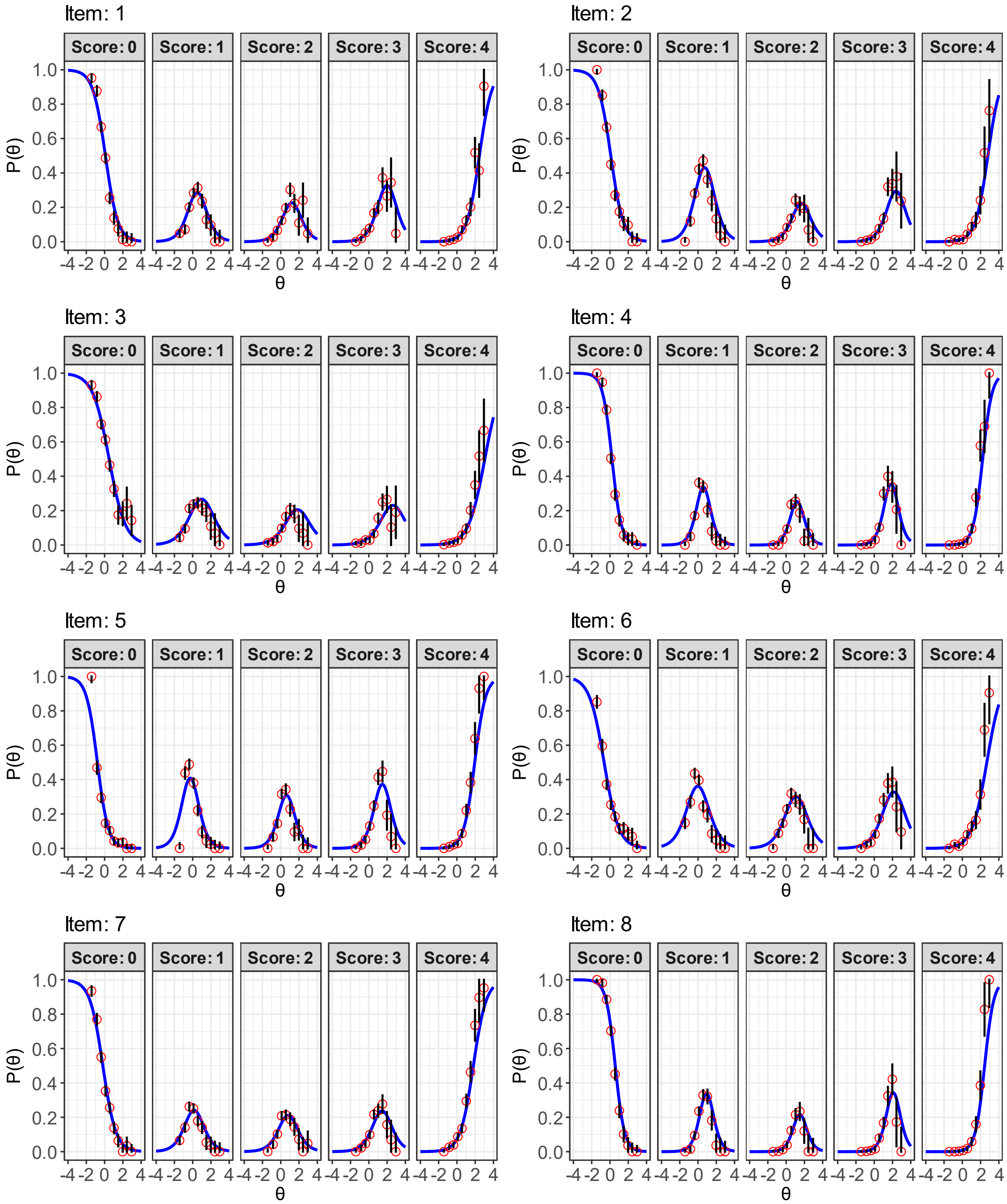

Notes. Response function and observed proportions with 95% CI. Items (1) *headache*, (2) *stomachache*, (3) *backache*, (4) *feeling low*, (5) *irritability/bad temper*, (6) *feeling nervous*, (7) *difficulties in getting to sleep*, (8) *feeling dizzy*.

Figure A8. Residual plots of the unidimensional GRM for AZE.

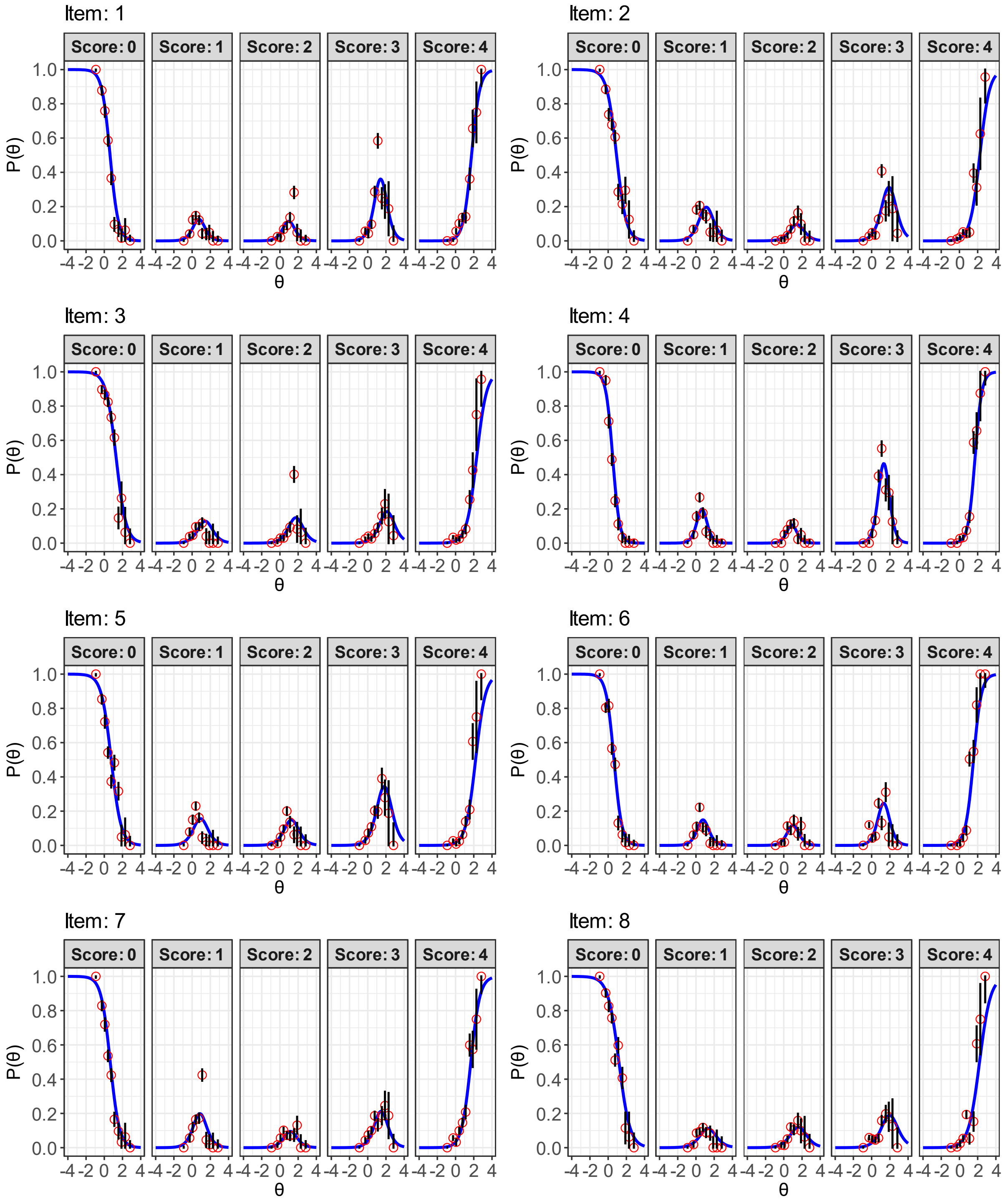

Notes. Response function and observed proportions with 95% CI. Items (1) *headache*, (2) *stomachache*, (3) *backache*, (4) *feeling low*, (5) *irritability/bad temper*, (6) *feeling nervous*, (7) *difficulties in getting to sleep*, (8) *feeling dizzy*.

Figure A9. Residual plots of the unidimensional GRM for BEL_FL.

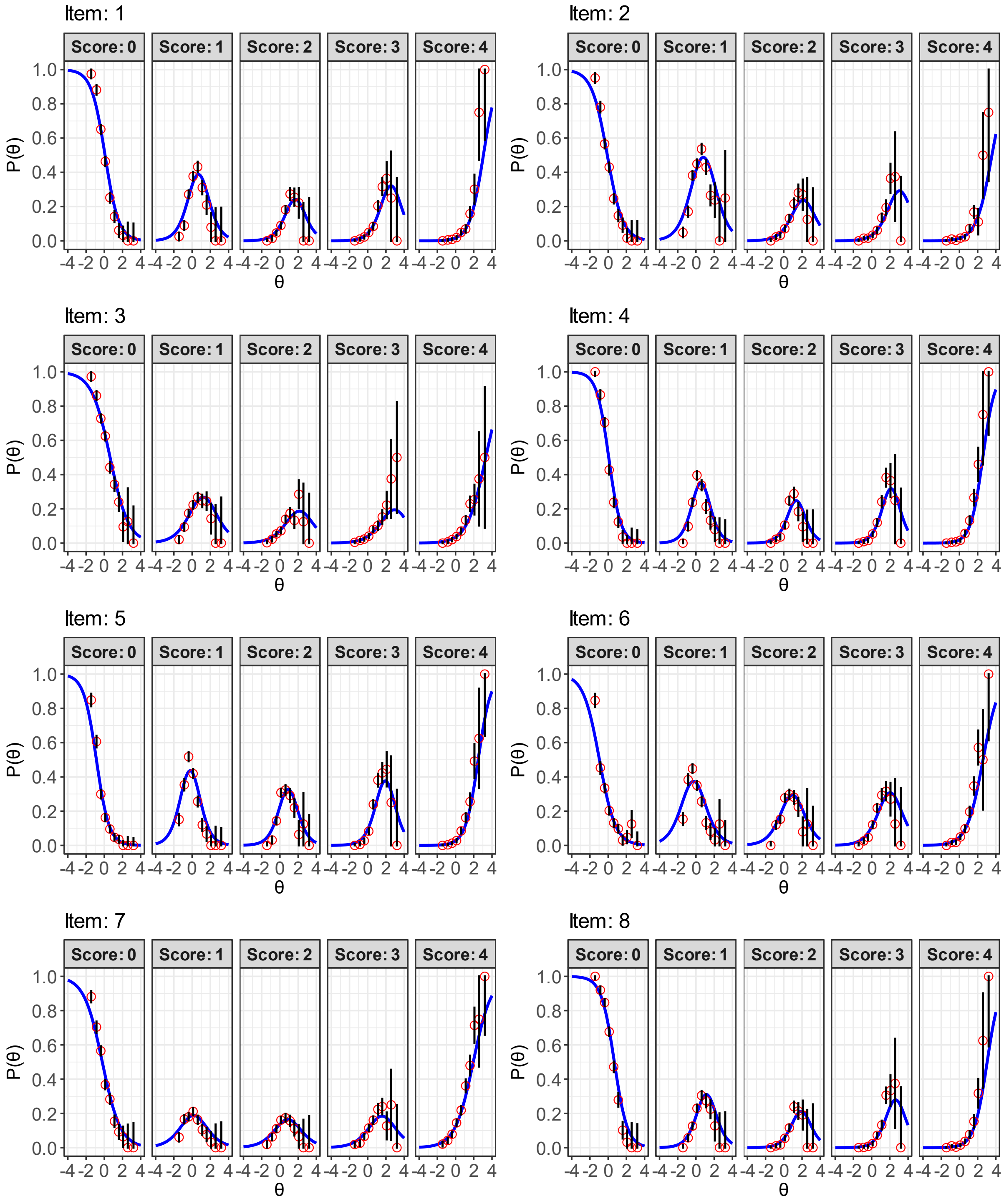

Notes. Response function and observed proportions with 95% CI. Items (1) *headache*, (2) *stomachache*, (3) *backache*, (4) *feeling low*, (5) *irritability/bad temper*, (6) *feeling nervous*, (7) *difficulties in getting to sleep*, (8) *feeling dizzy*.

Figure A10. Residual plots of the unidimensional GRM for BEL_FR.

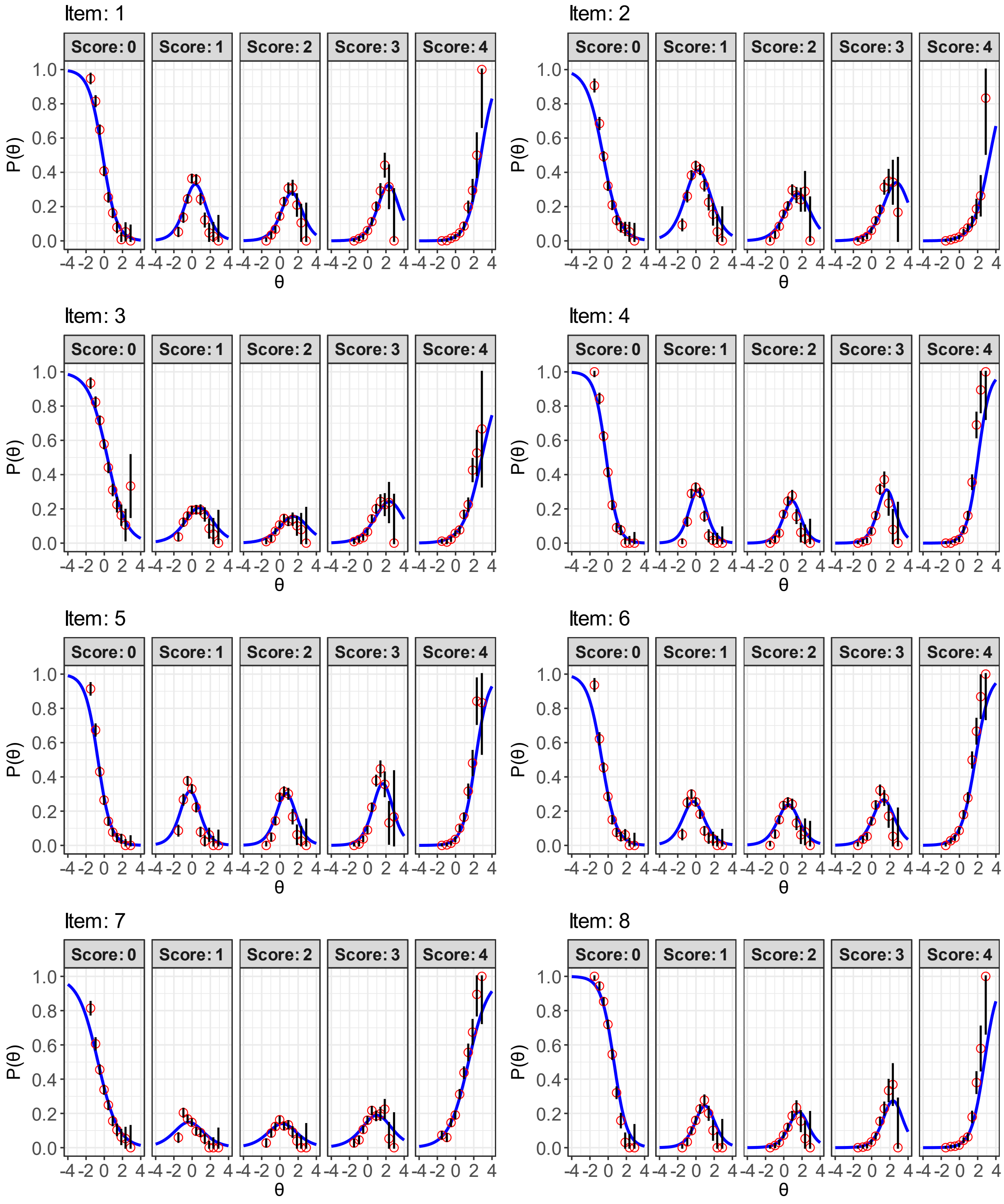

Notes. Response function and observed proportions with 95% CI. Items (1) *headache*, (2) *stomachache*, (3) *backache*, (4) *feeling low*, (5) *irritability/bad temper*, (6) *feeling nervous*, (7) *difficulties in getting to sleep*, (8) *feeling dizzy*.

Figure A11. Residual plots of the unidimensional GRM for BGR.

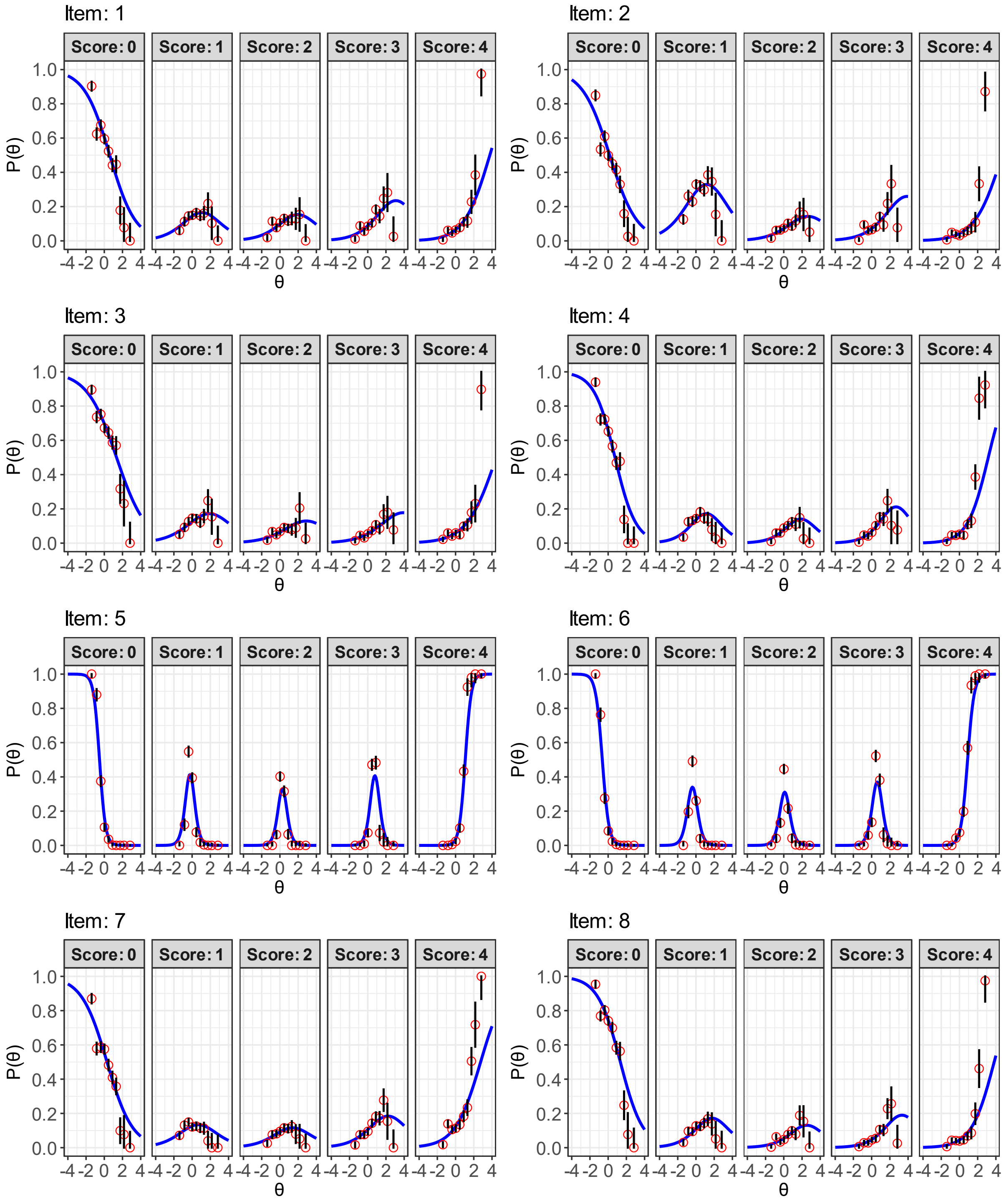

Notes. Response function and observed proportions with 95% CI. Items (1) *headache*, (2) *stomachache*, (3) *backache*, (4) *feeling low*, (5) *irritability/bad temper*, (6) *feeling nervous*, (7) *difficulties in getting to sleep*, (8) *feeling dizzy*.

Figure A12. Residual plots of the unidimensional GRM for CAN.

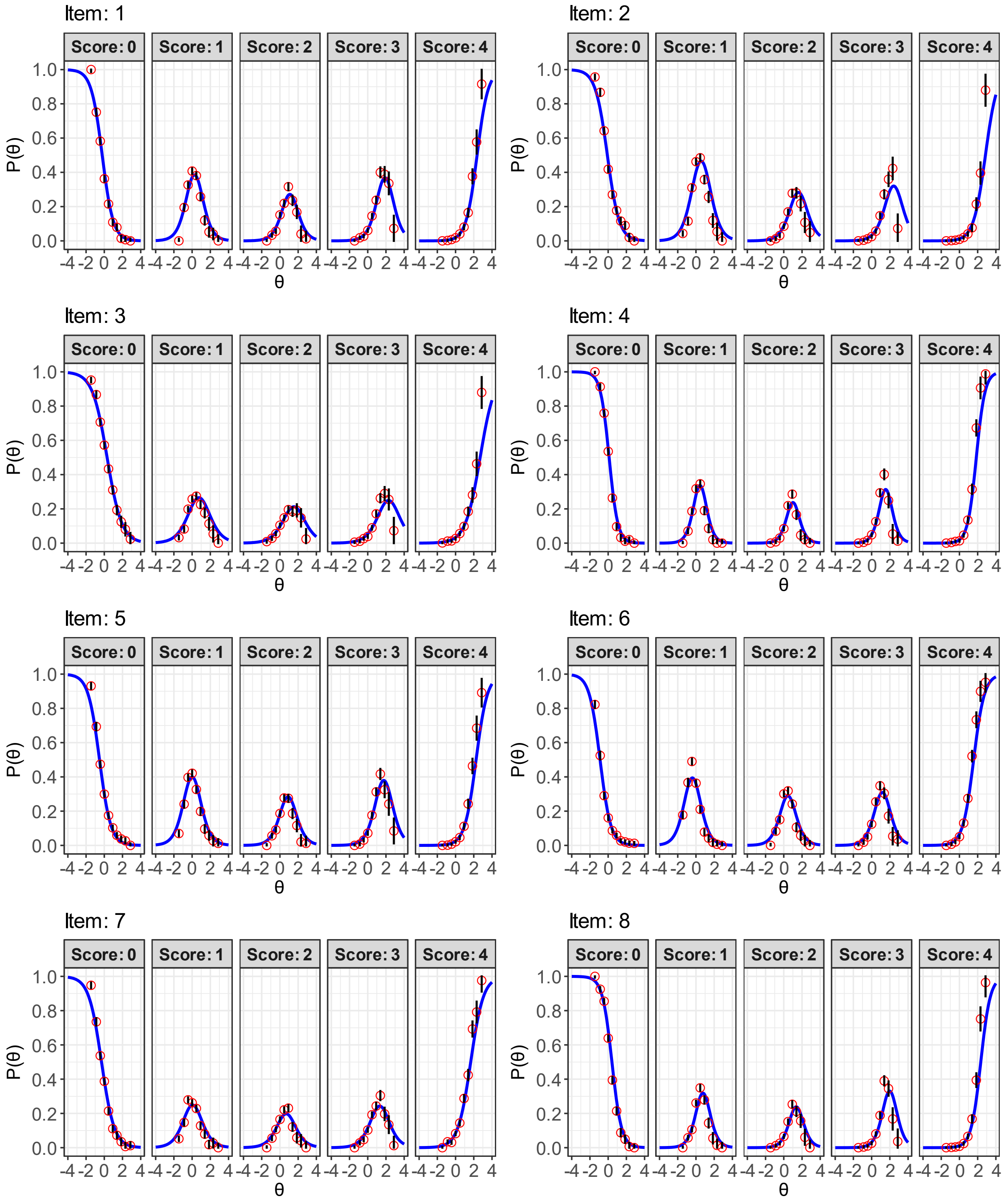

Notes. Response function and observed proportions with 95% CI. Items (1) *headache*, (2) *stomachache*, (3) *backache*, (4) *feeling low*, (5) *irritability/bad temper*, (6) *feeling nervous*, (7) *difficulties in getting to sleep*, (8) *feeling dizzy*.

Figure A13. Residual plots of the unidimensional GRM for CHE.

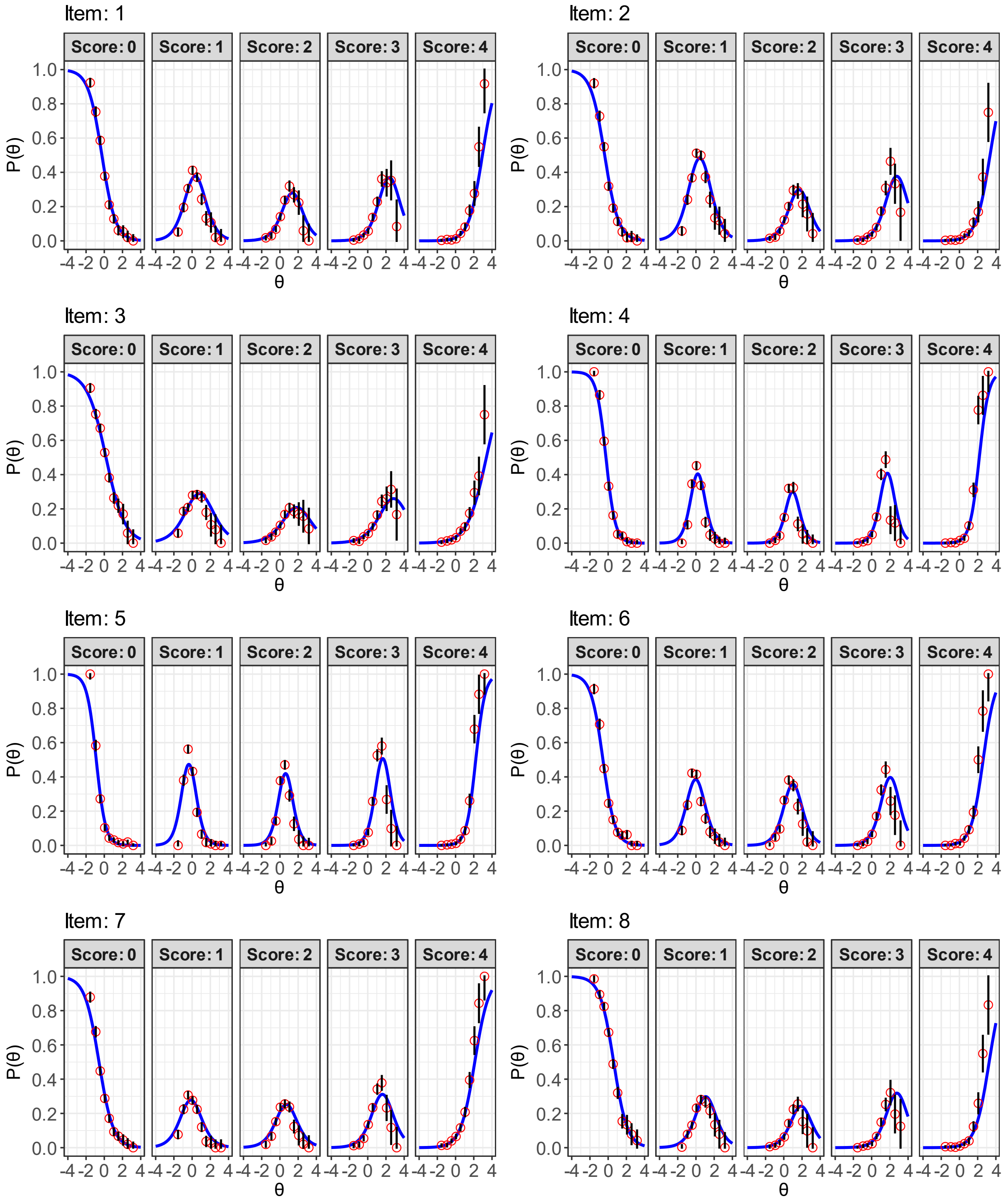

Notes. Response function and observed proportions with 95% CI. Items (1) *headache*, (2) *stomachache*, (3) *backache*, (4) *feeling low*, (5) *irritability/bad temper*, (6) *feeling nervous*, (7) *difficulties in getting to sleep*, (8) *feeling dizzy*.

Figure A14. Residual plots of the unidimensional GRM for CZE.

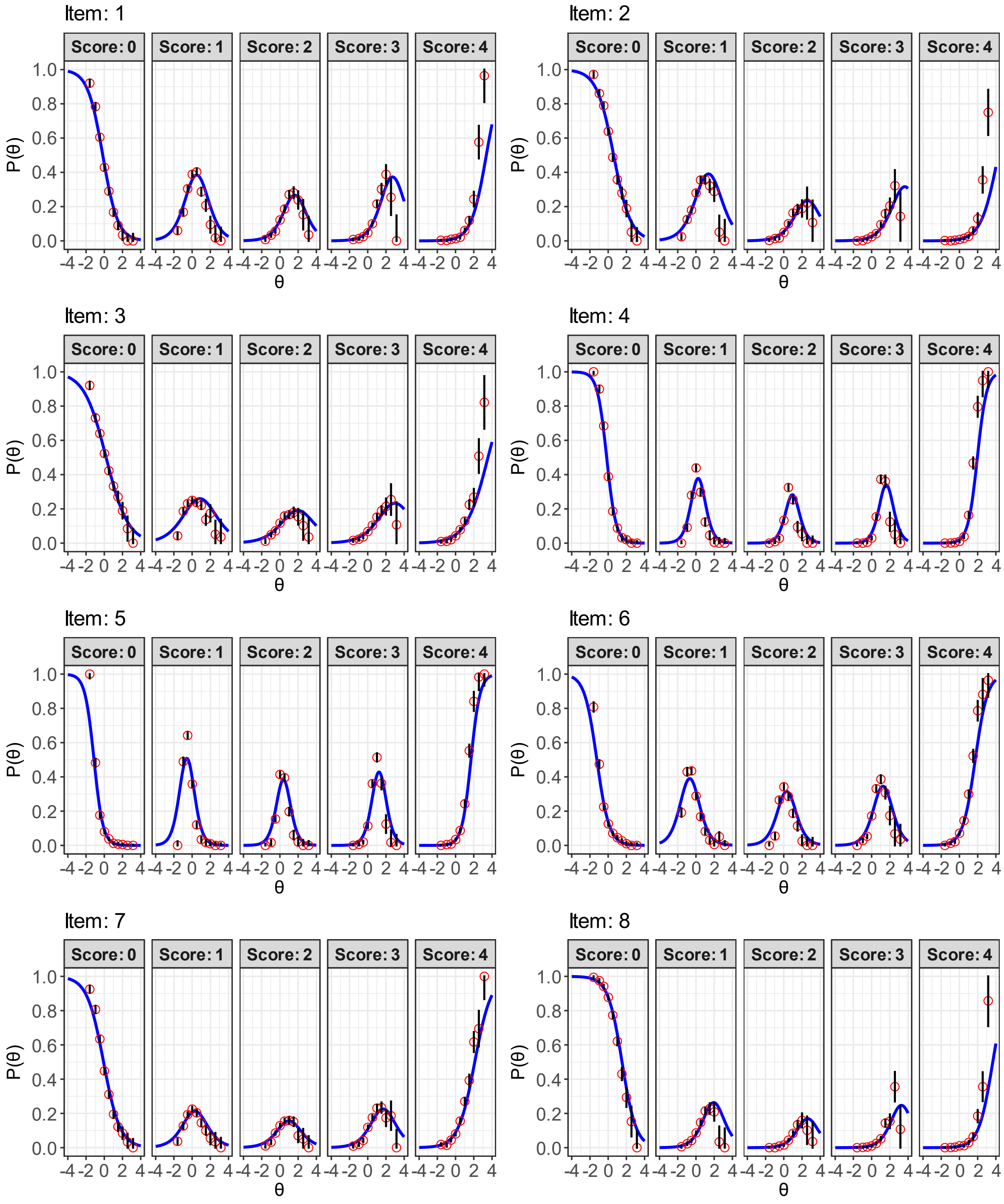

Notes. Response function and observed proportions with 95% CI. Items (1) *headache*, (2) *stomachache*, (3) *backache*, (4) *feeling low*, (5) *irritability/bad temper*, (6) *feeling nervous*, (7) *difficulties in getting to sleep*, (8) *feeling dizzy*.

Figure A15. Residual plots of the unidimensional GRM for DEU.

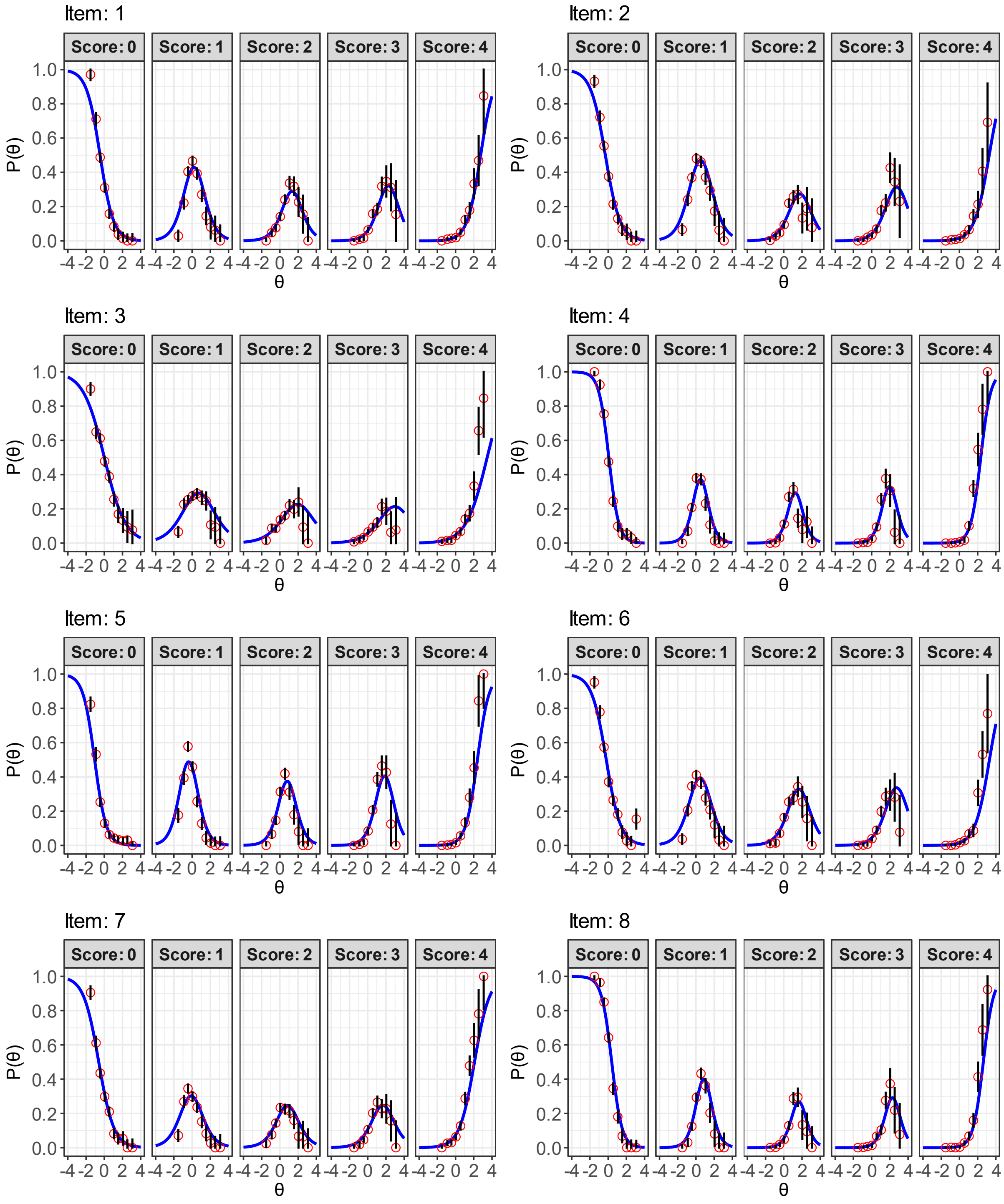

Notes. Response function and observed proportions with 95% CI. Items (1) *headache*, (2) *stomachache*, (3) *backache*, (4) *feeling low*, (5) *irritability/bad temper*, (6) *feeling nervous*, (7) *difficulties in getting to sleep*, (8) *feeling dizzy*.

Figure A16. Residual plots of the unidimensional GRM for DNK.

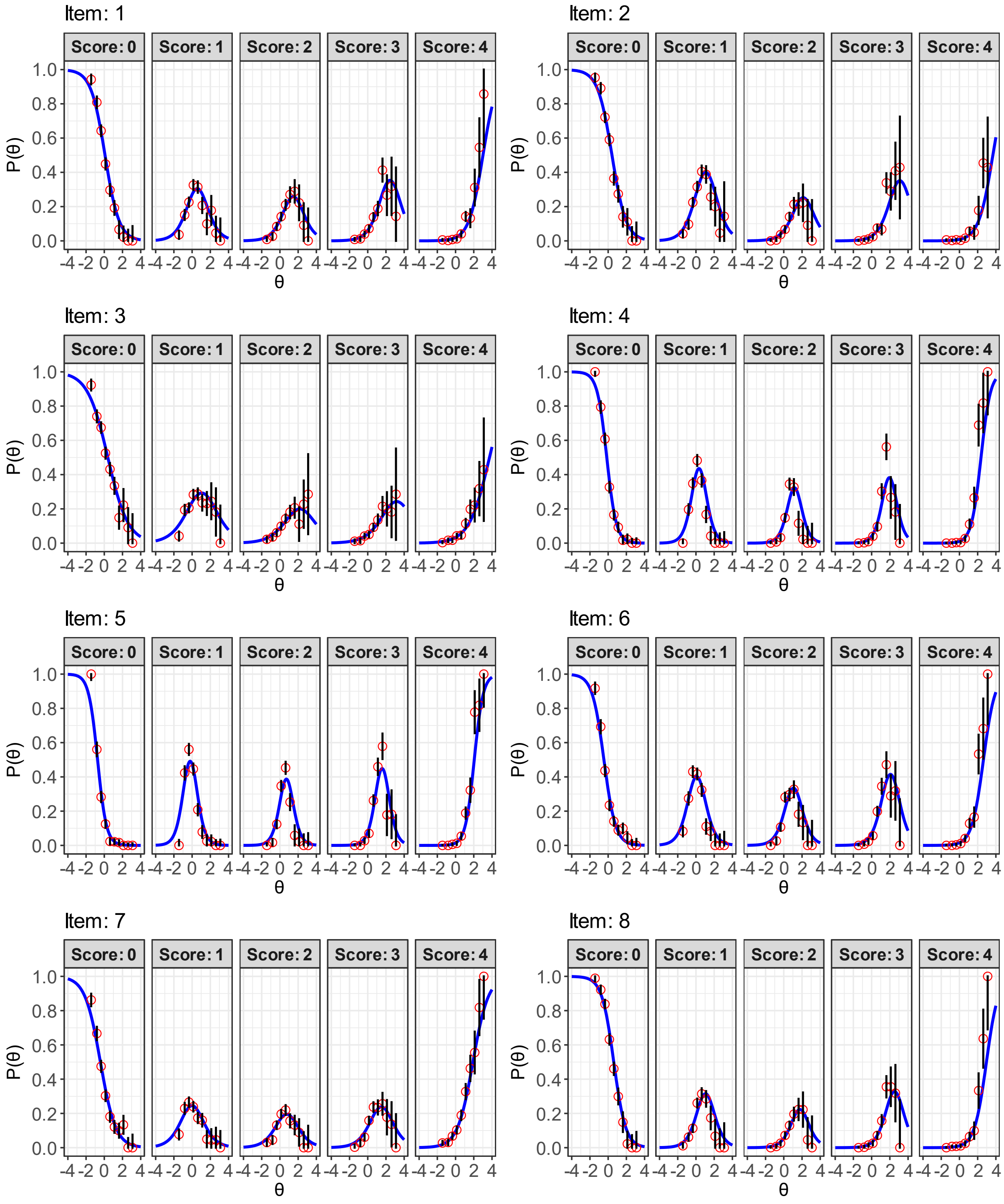

Notes. Response function and observed proportions with 95% CI. Items (1) *headache*, (2) *stomachache*, (3) *backache*, (4) *feeling low*, (5) *irritability/bad temper*, (6) *feeling nervous*, (7) *difficulties in getting to sleep*, (8) *feeling dizzy*.

Figure A17. Residual plots of the unidimensional GRM for ESP.

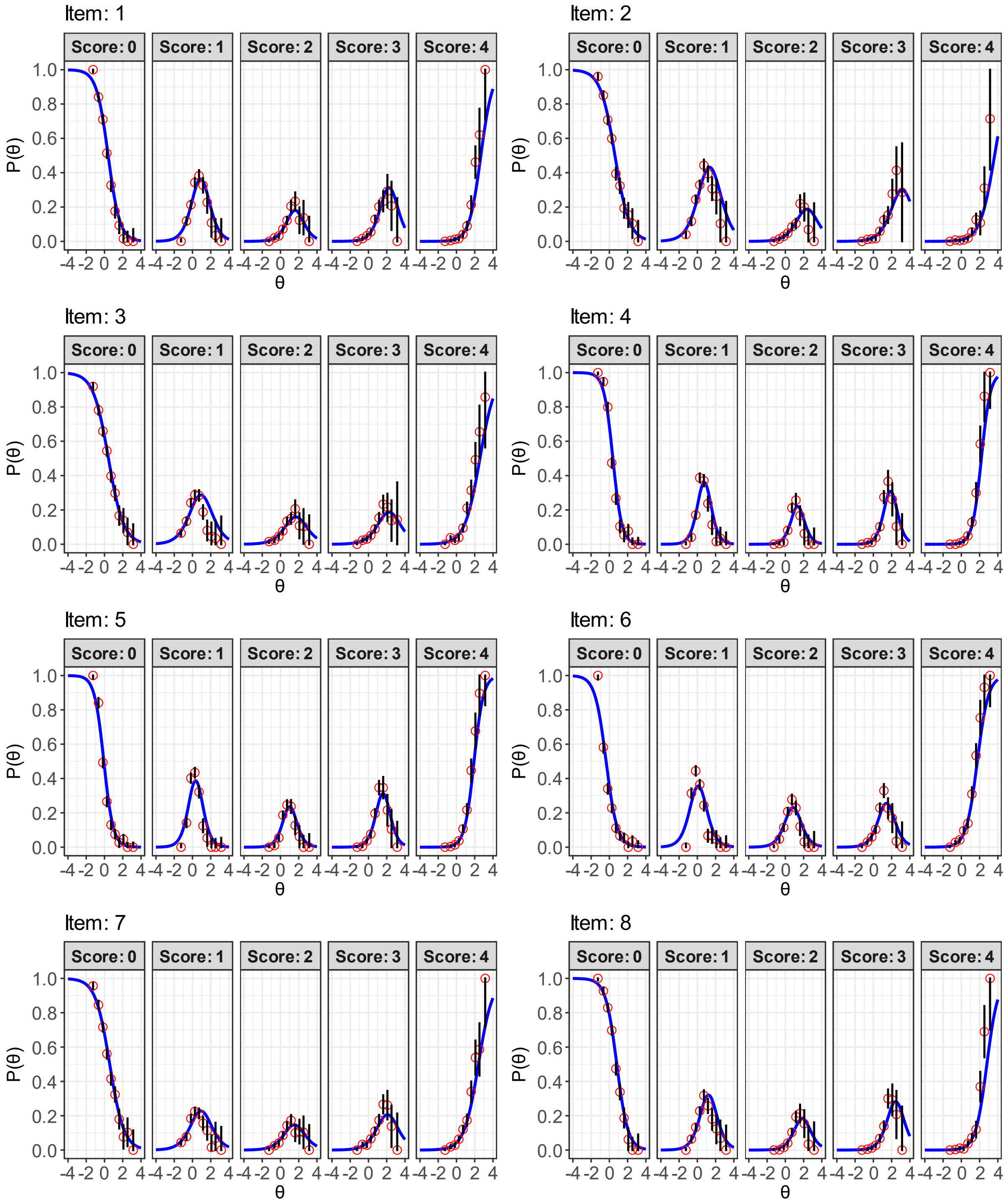

Notes. Response function and observed proportions with 95% CI. Items (1) *headache*, (2) *stomachache*, (3) *backache*, (4) *feeling low*, (5) *irritability/bad temper*, (6) *feeling nervous*, (7) *difficulties in getting to sleep*, (8) *feeling dizzy*.

Figure A18. Residual plots of the unidimensional GRM for EST.

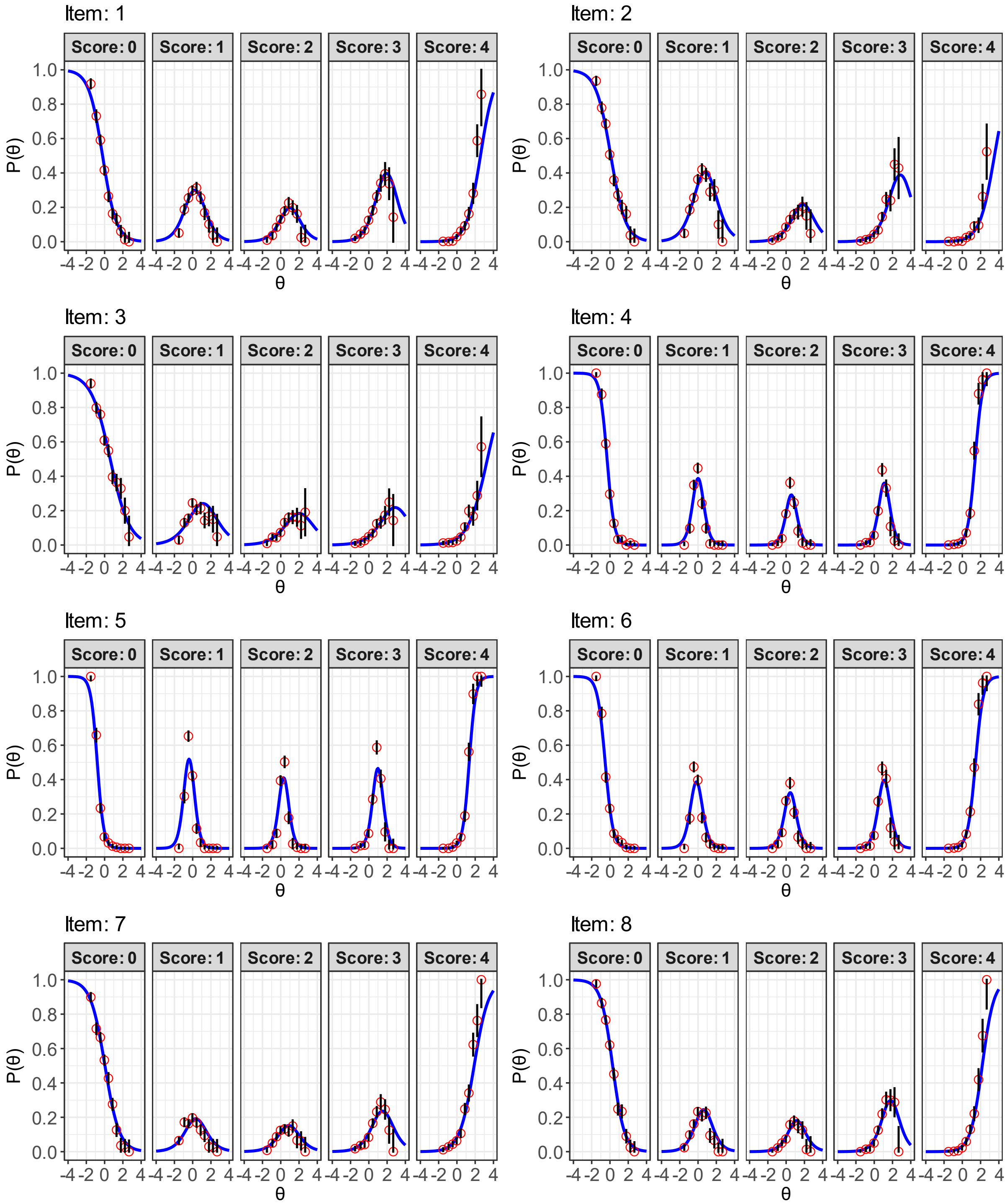

Notes. Response function and observed proportions with 95% CI. Items (1) *headache*, (2) *stomachache*, (3) *backache*, (4) *feeling low*, (5) *irritability/bad temper*, (6) *feeling nervous*, (7) *difficulties in getting to sleep*, (8) *feeling dizzy*.

Figure A19. Residual plots of the unidimensional GRM for FIN.

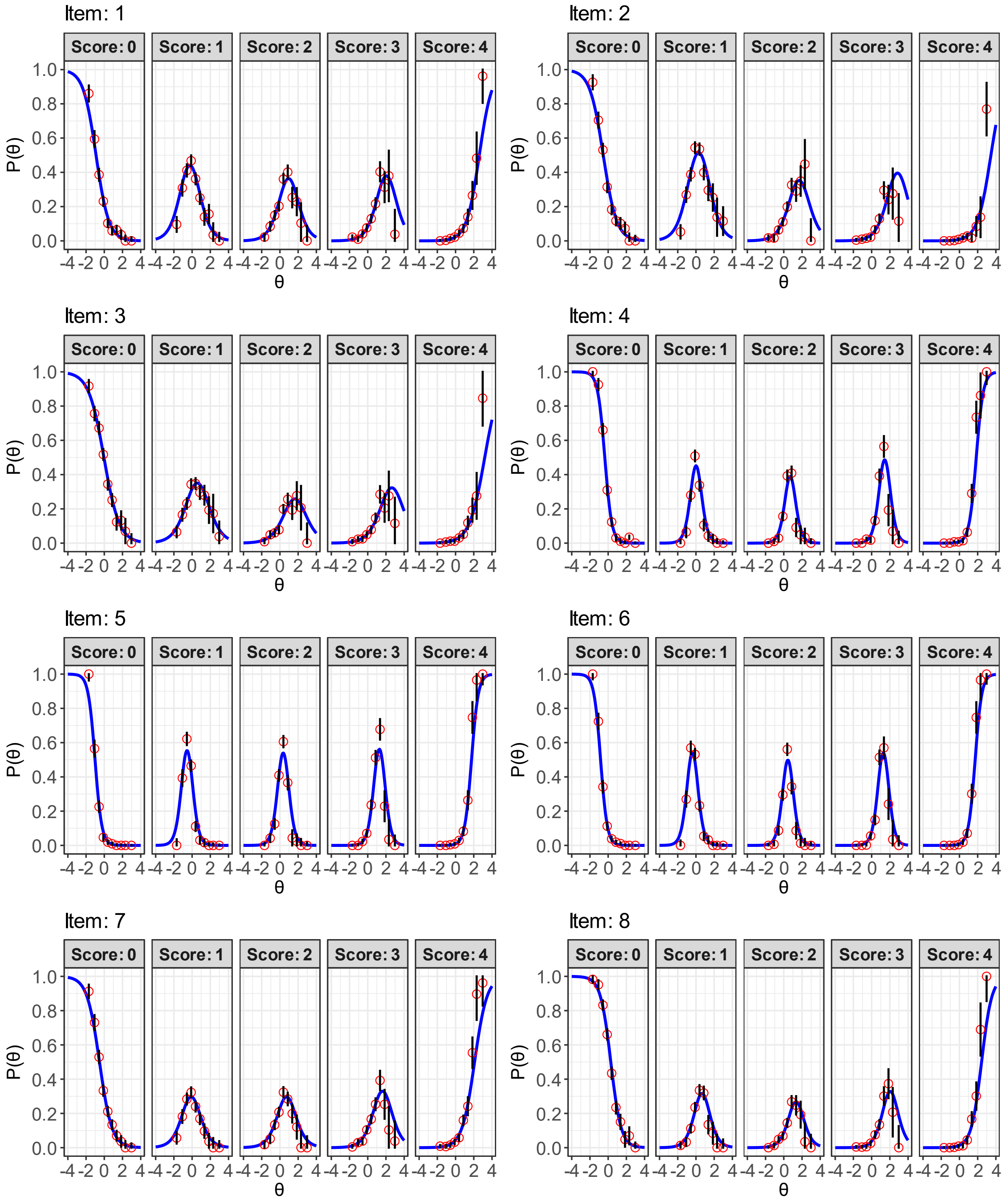

Notes. Response function and observed proportions with 95% CI. Items (1) *headache*, (2) *stomachache*, (3) *backache*, (4) *feeling low*, (5) *irritability/bad temper*, (6) *feeling nervous*, (7) *difficulties in getting to sleep*, (8) *feeling dizzy*.

Figure A20. Residual plots of the unidimensional GRM for FRA.

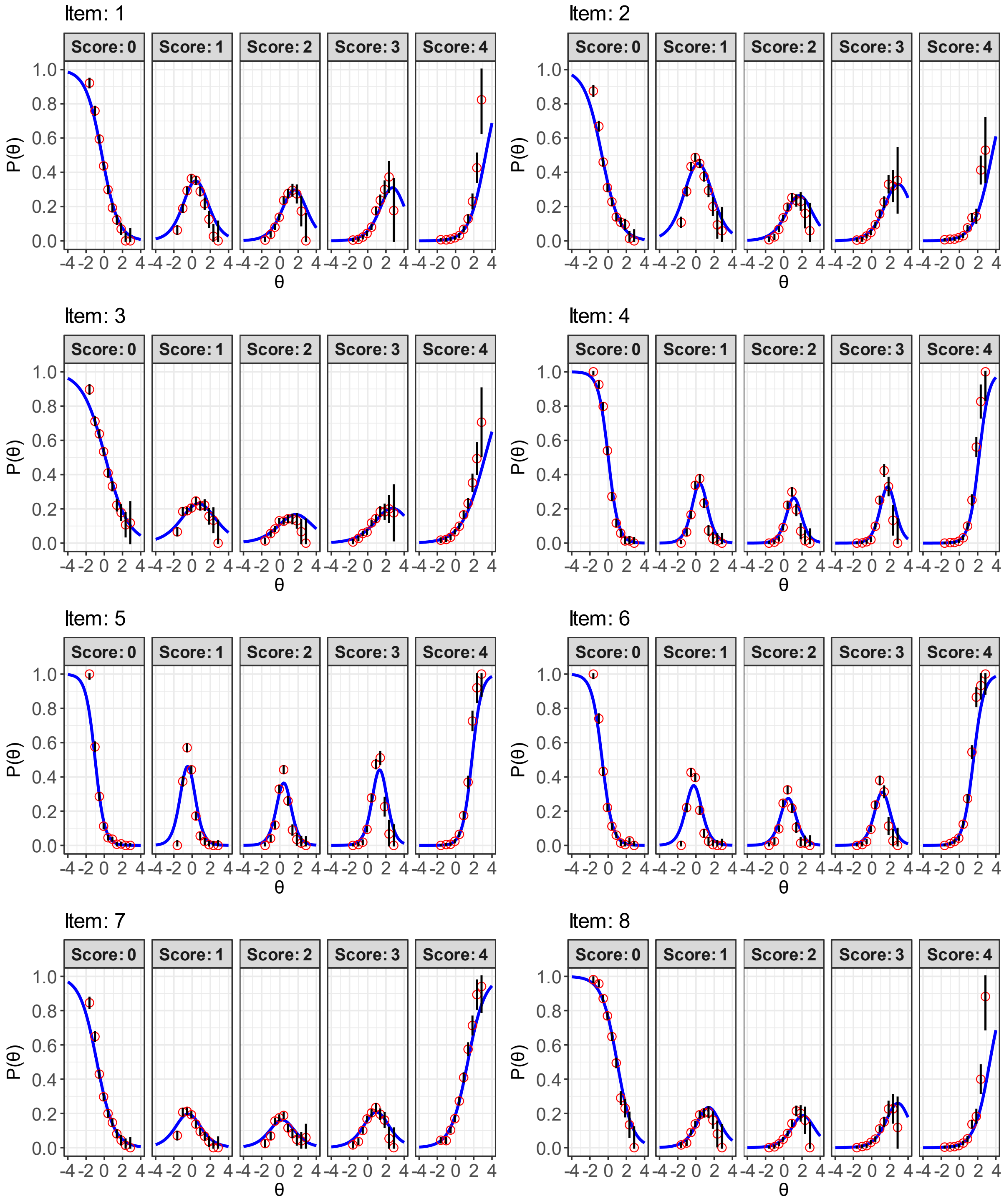

Notes. Response function and observed proportions with 95% CI. Items (1) *headache*, (2) *stomachache*, (3) *backache*, (4) *feeling low*, (5) *irritability/bad temper*, (6) *feeling nervous*, (7) *difficulties in getting to sleep*, (8) *feeling dizzy*.

Figure A21. Residual plots of the unidimensional GRM for GB_ENG.

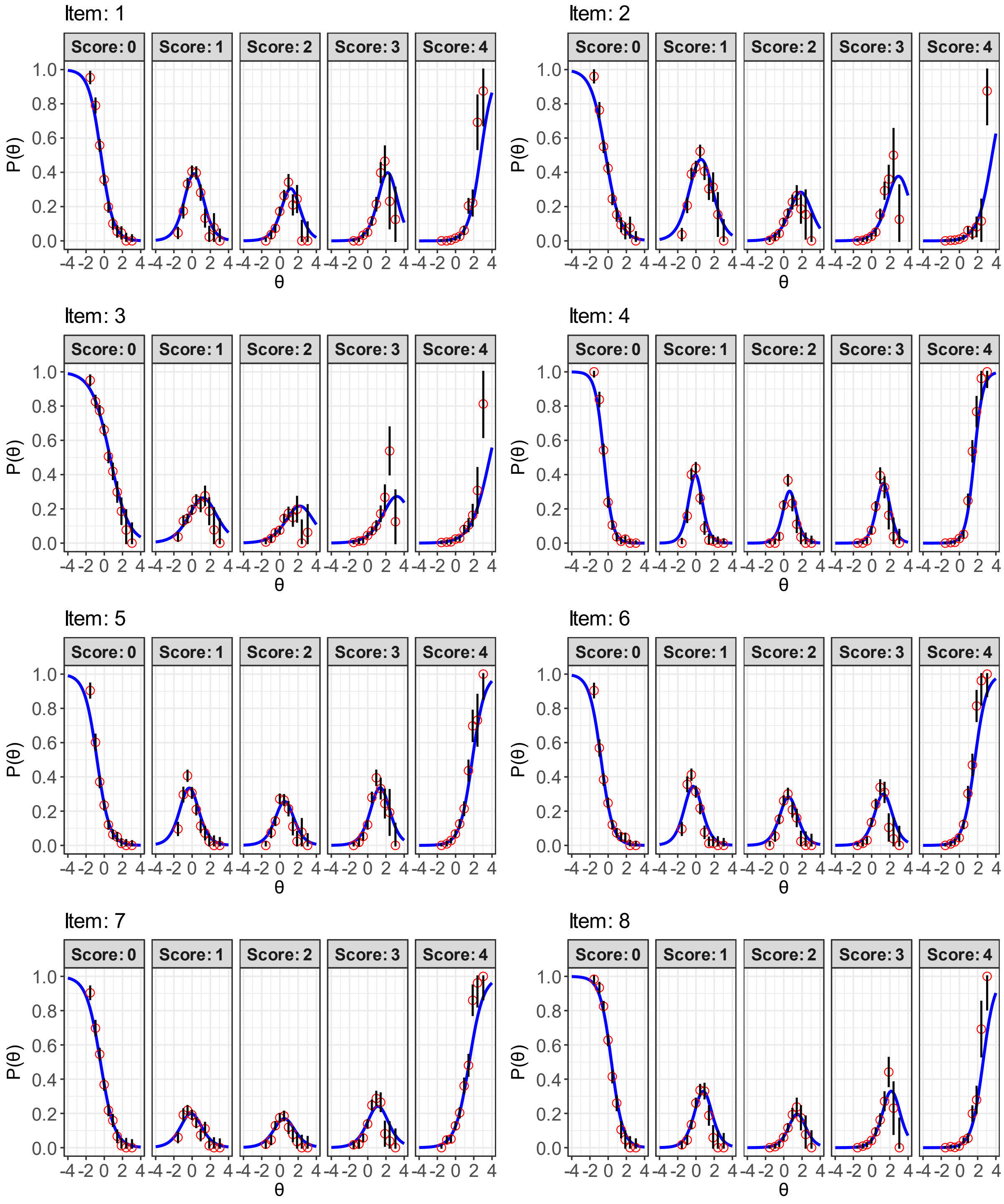

Notes. Response function and observed proportions with 95% CI. Items (1) *headache*, (2) *stomachache*, (3) *backache*, (4) *feeling low*, (5) *irritability/bad temper*, (6) *feeling nervous*, (7) *difficulties in getting to sleep*, (8) *feeling dizzy*.

Figure A22. Residual plots of the unidimensional GRM for GB_SCT.

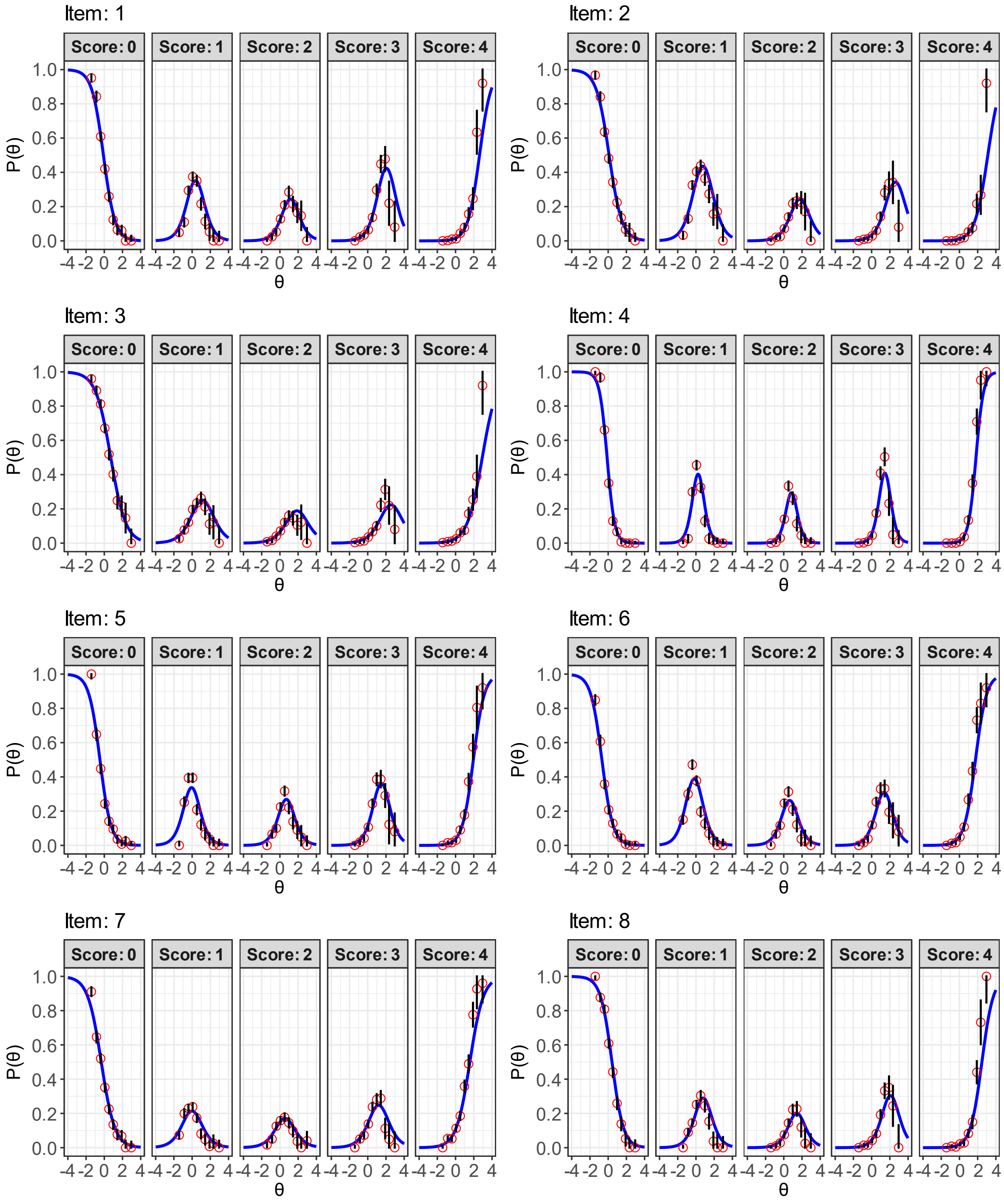

Notes. Response function and observed proportions with 95% CI. Items (1) *headache*, (2) *stomachache*, (3) *backache*, (4) *feeling low*, (5) *irritability/bad temper*, (6) *feeling nervous*, (7) *difficulties in getting to sleep*, (8) *feeling dizzy*.

Figure A23. Residual plots of the unidimensional GRM for GB_WLS.

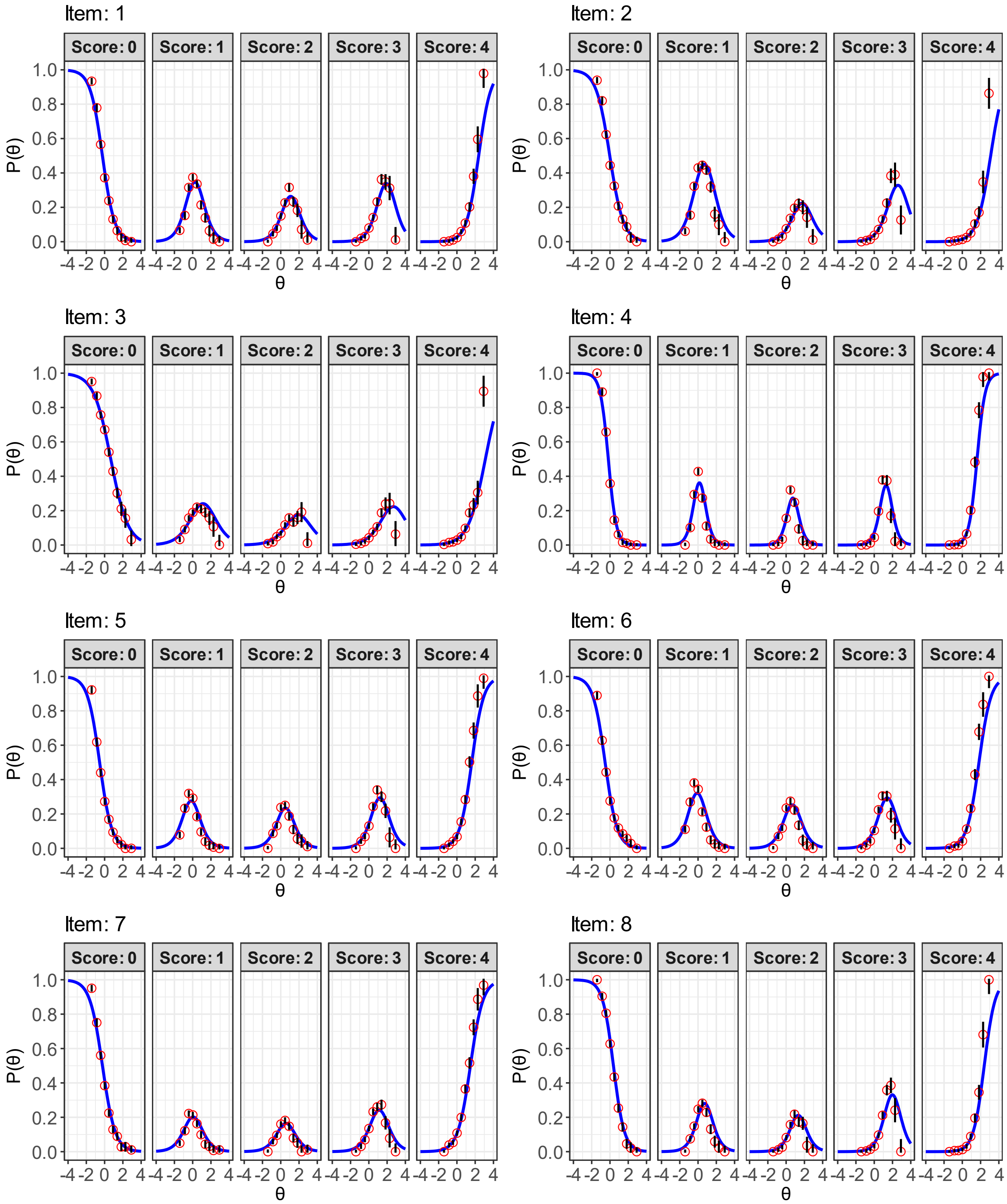

Notes. Response function and observed proportions with 95% CI. Items (1) *headache*, (2) *stomachache*, (3) *backache*, (4) *feeling low*, (5) *irritability/bad temper*, (6) *feeling nervous*, (7) *difficulties in getting to sleep*, (8) *feeling dizzy*.

Figure A24. Residual plots of the unidimensional GRM for GEO.

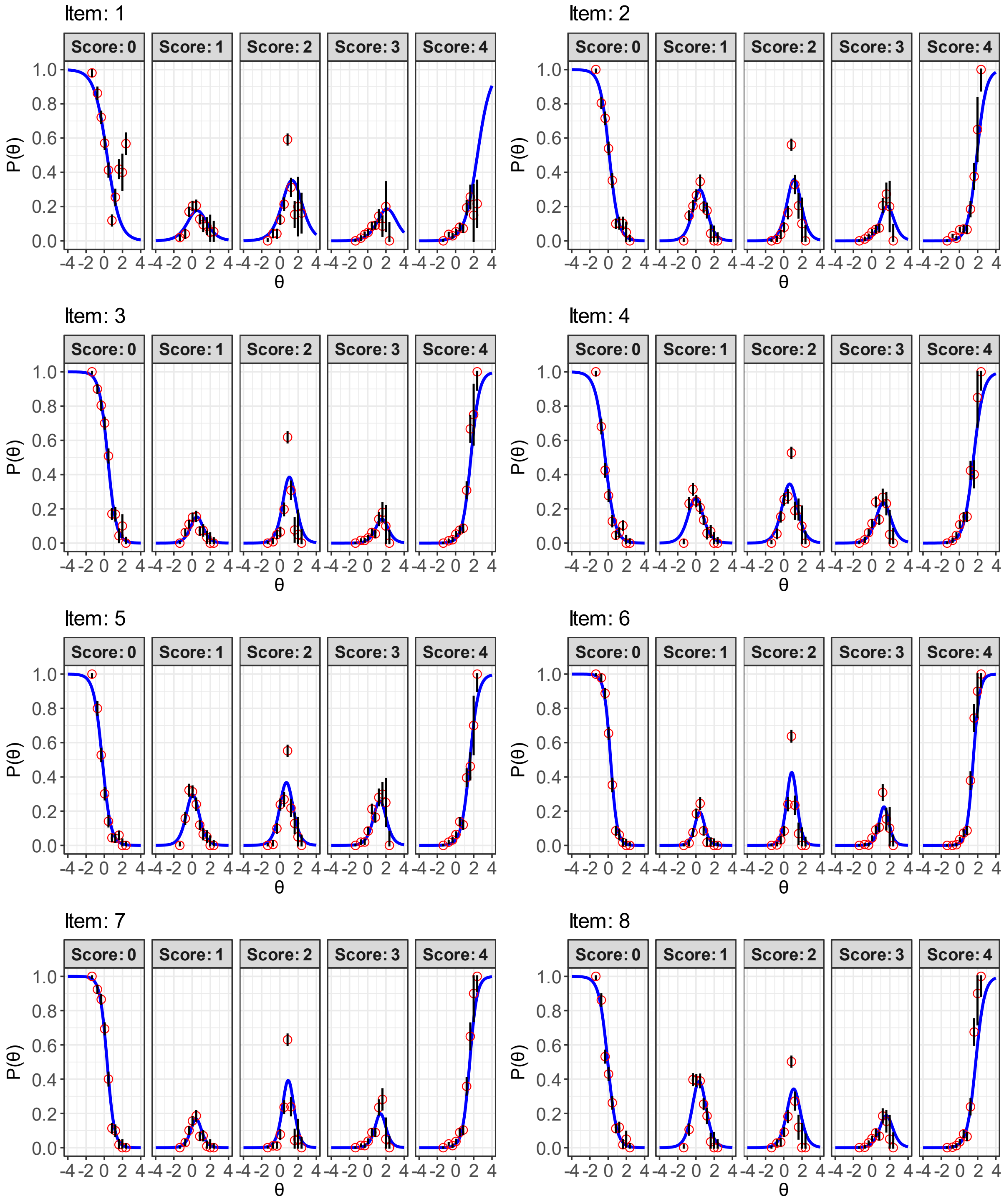

Notes. Response function and observed proportions with 95% CI. Items (1) *headache*, (2) *stomachache*, (3) *backache*, (4) *feeling low*, (5) *irritability/bad temper*, (6) *feeling nervous*, (7) *difficulties in getting to sleep*, (8) *feeling dizzy*.

Figure A25. Residual plots of the unidimensional GRM for GRC.

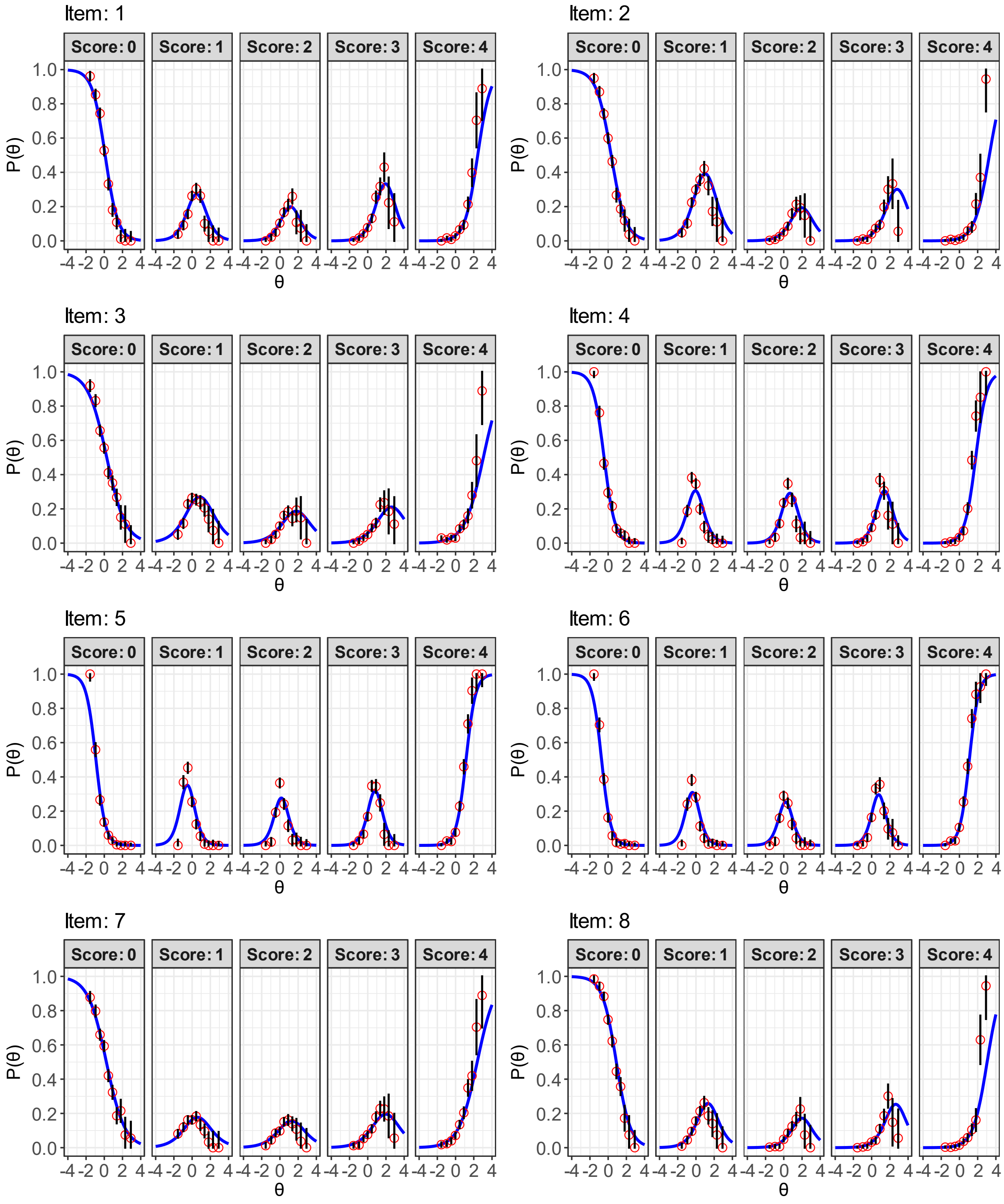

Notes. Response function and observed proportions with 95% CI. Items (1) *headache*, (2) *stomachache*, (3) *backache*, (4) *feeling low*, (5) *irritability/bad temper*, (6) *feeling nervous*, (7) *difficulties in getting to sleep*, (8) *feeling dizzy*.

Figure A26. Residual plots of the unidimensional GRM for GRL.

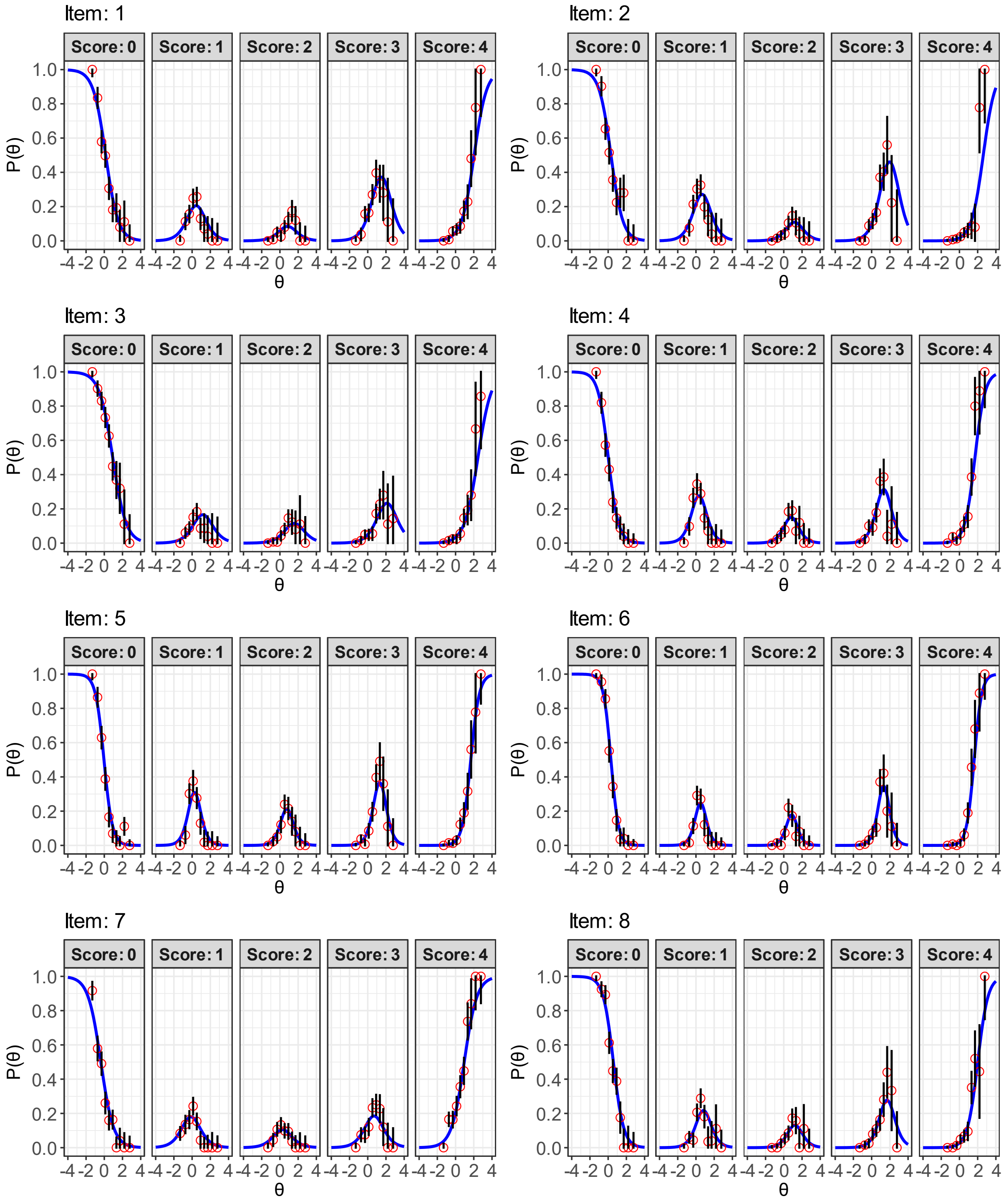

Notes. Response function and observed proportions with 95% CI. Items (1) *headache*, (2) *stomachache*, (3) *backache*, (4) *feeling low*, (5) *irritability/bad temper*, (6) *feeling nervous*, (7) *difficulties in getting to sleep*, (8) *feeling dizzy*.

Figure A27. Residual plots of the unidimensional GRM for HRV.

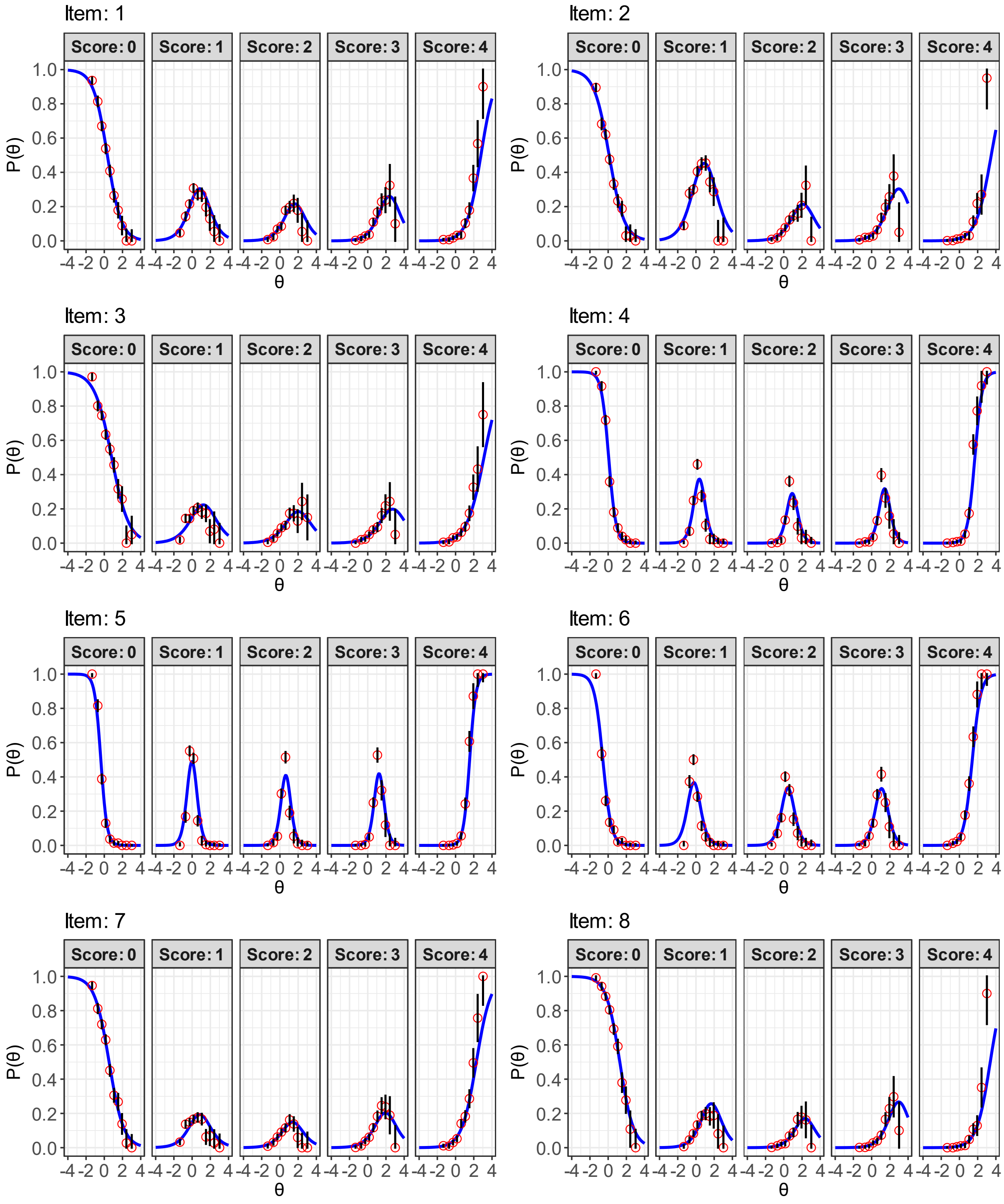

Notes. Response function and observed proportions with 95% CI. Items (1) *headache*, (2) *stomachache*, (3) *backache*, (4) *feeling low*, (5) *irritability/bad temper*, (6) *feeling nervous*, (7) *difficulties in getting to sleep*, (8) *feeling dizzy*.

Figure A28. Residual plots of the unidimensional GRM for HUN.

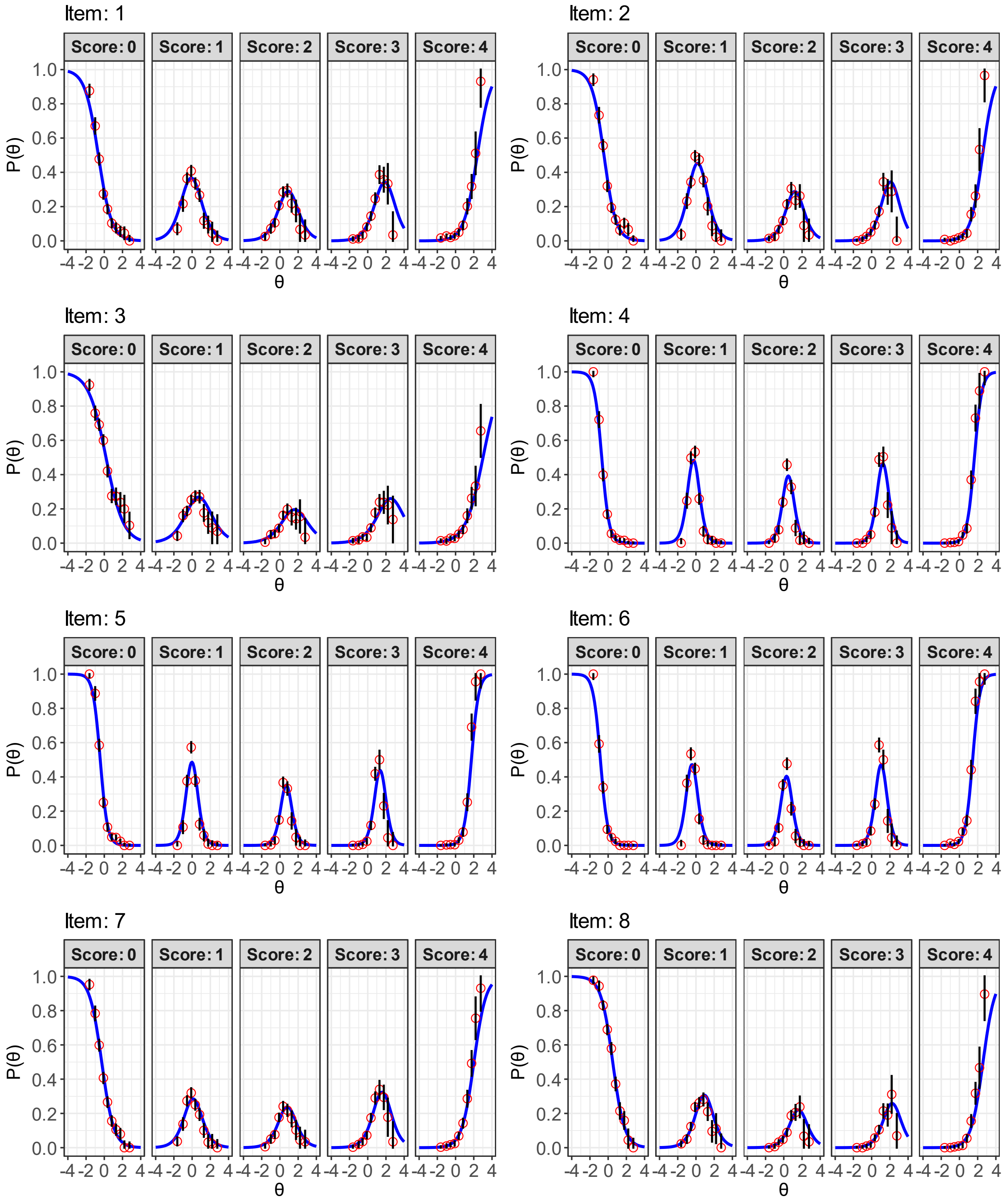

Notes. Response function and observed proportions with 95% CI. Items (1) *headache*, (2) *stomachache*, (3) *backache*, (4) *feeling low*, (5) *irritability/bad temper*, (6) *feeling nervous*, (7) *difficulties in getting to sleep*, (8) *feeling dizzy*.

Figure A29. Residual plots of the unidimensional GRM for IRL.

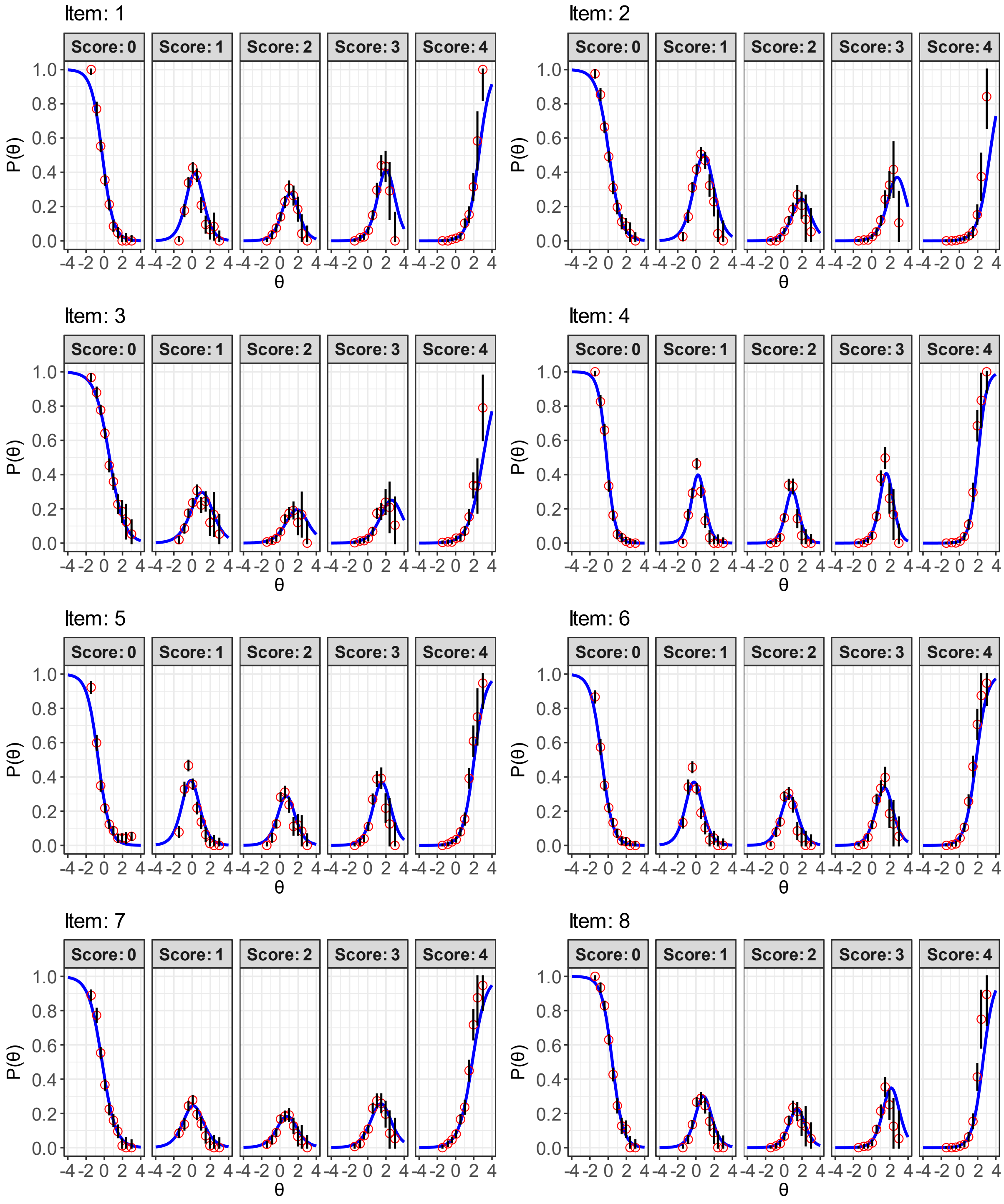

Notes. Response function and observed proportions with 95% CI. Items (1) *headache*, (2) *stomachache*, (3) *backache*, (4) *feeling low*, (5) *irritability/bad temper*, (6) *feeling nervous*, (7) *difficulties in getting to sleep*, (8) *feeling dizzy*.

Figure A30. Residual plots of the unidimensional GRM for ISL.

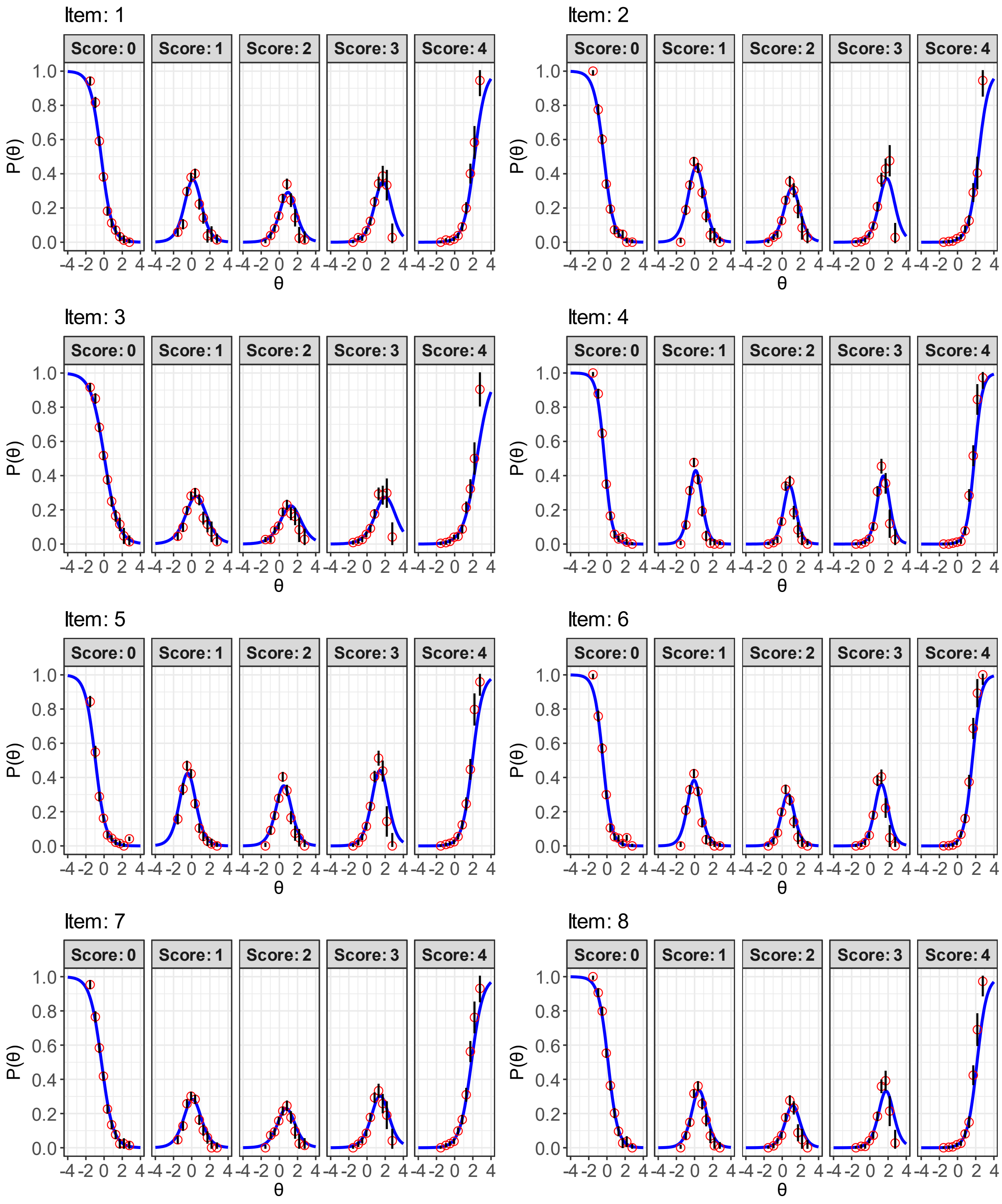

Notes. Response function and observed proportions with 95% CI. Items (1) *headache*, (2) *stomachache*, (3) *backache*, (4) *feeling low*, (5) *irritability/bad temper*, (6) *feeling nervous*, (7) *difficulties in getting to sleep*, (8) *feeling dizzy*.

Figure A31. Residual plots of the unidimensional GRM for ISR.

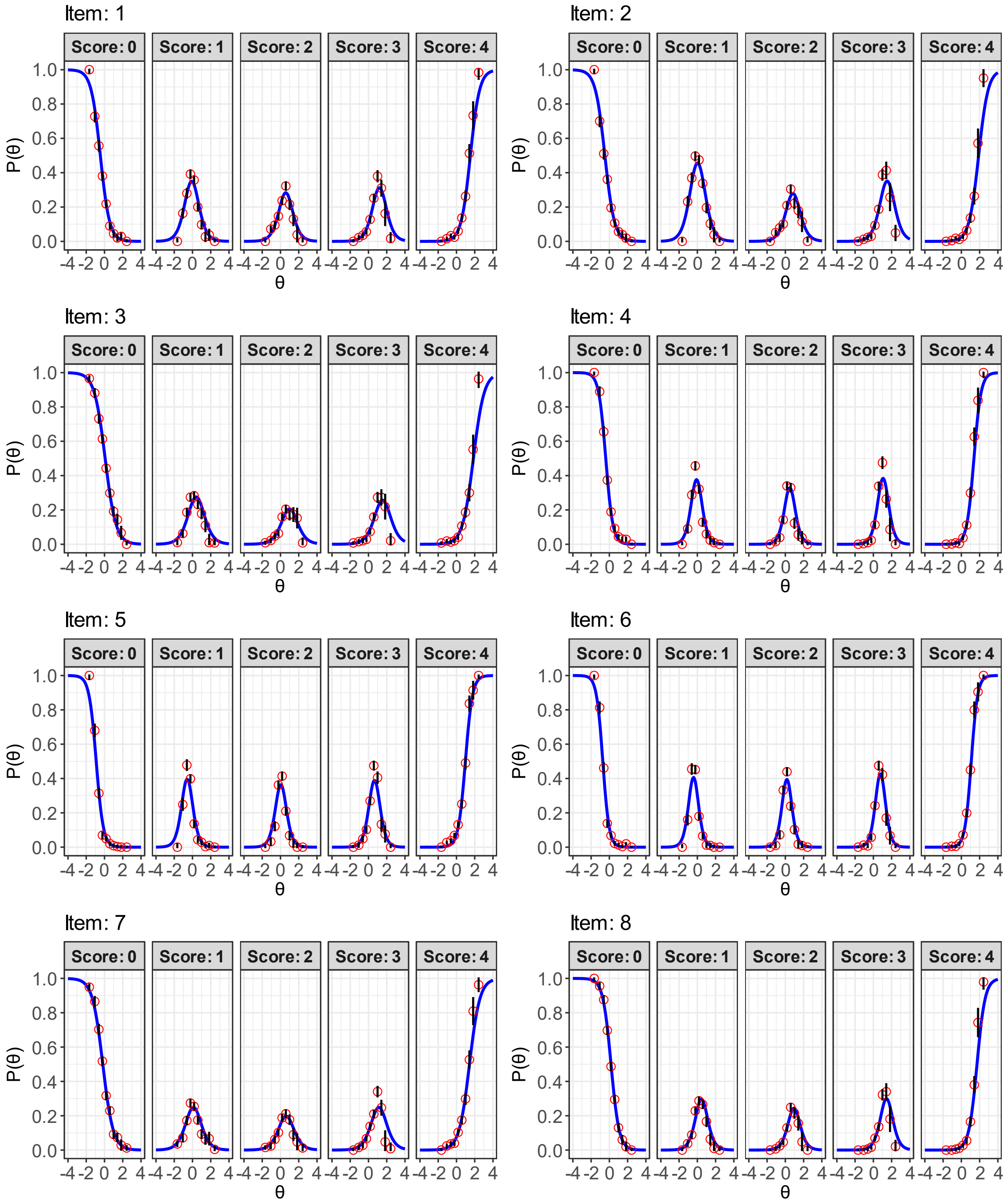

Notes. Response function and observed proportions with 95% CI. Items (1) *headache*, (2) *stomachache*, (3) *backache*, (4) *feeling low*, (5) *irritability/bad temper*, (6) *feeling nervous*, (7) *difficulties in getting to sleep*, (8) *feeling dizzy*.

Figure A32. Residual plots of the unidimensional GRM for ITA.

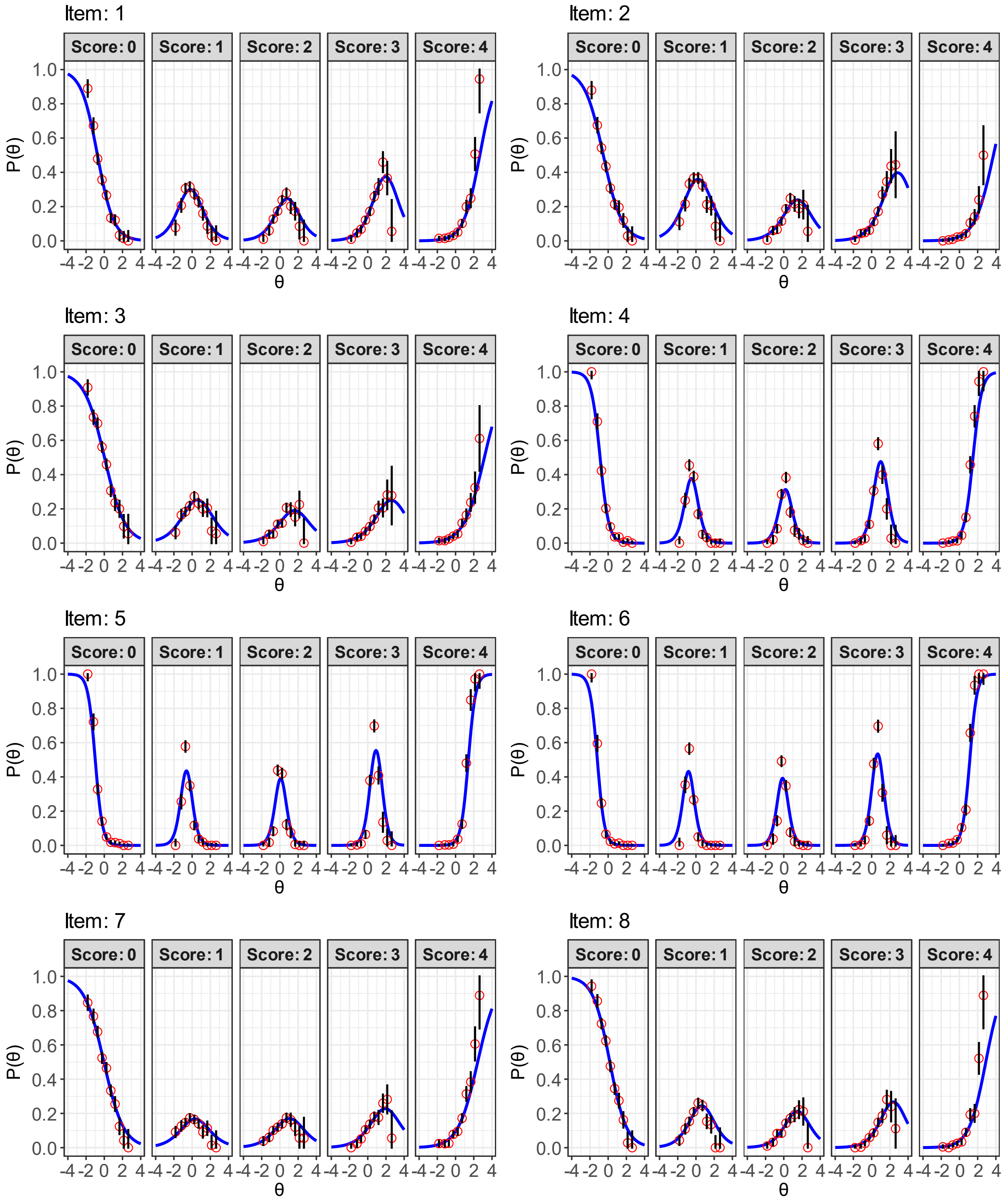

Notes. Response function and observed proportions with 95% CI. Items (1) *headache*, (2) *stomachache*, (3) *backache*, (4) *feeling low*, (5) *irritability/bad temper*, (6) *feeling nervous*, (7) *difficulties in getting to sleep*, (8) *feeling dizzy*.

Figure A33. Residual plots of the unidimensional GRM for KAZ.

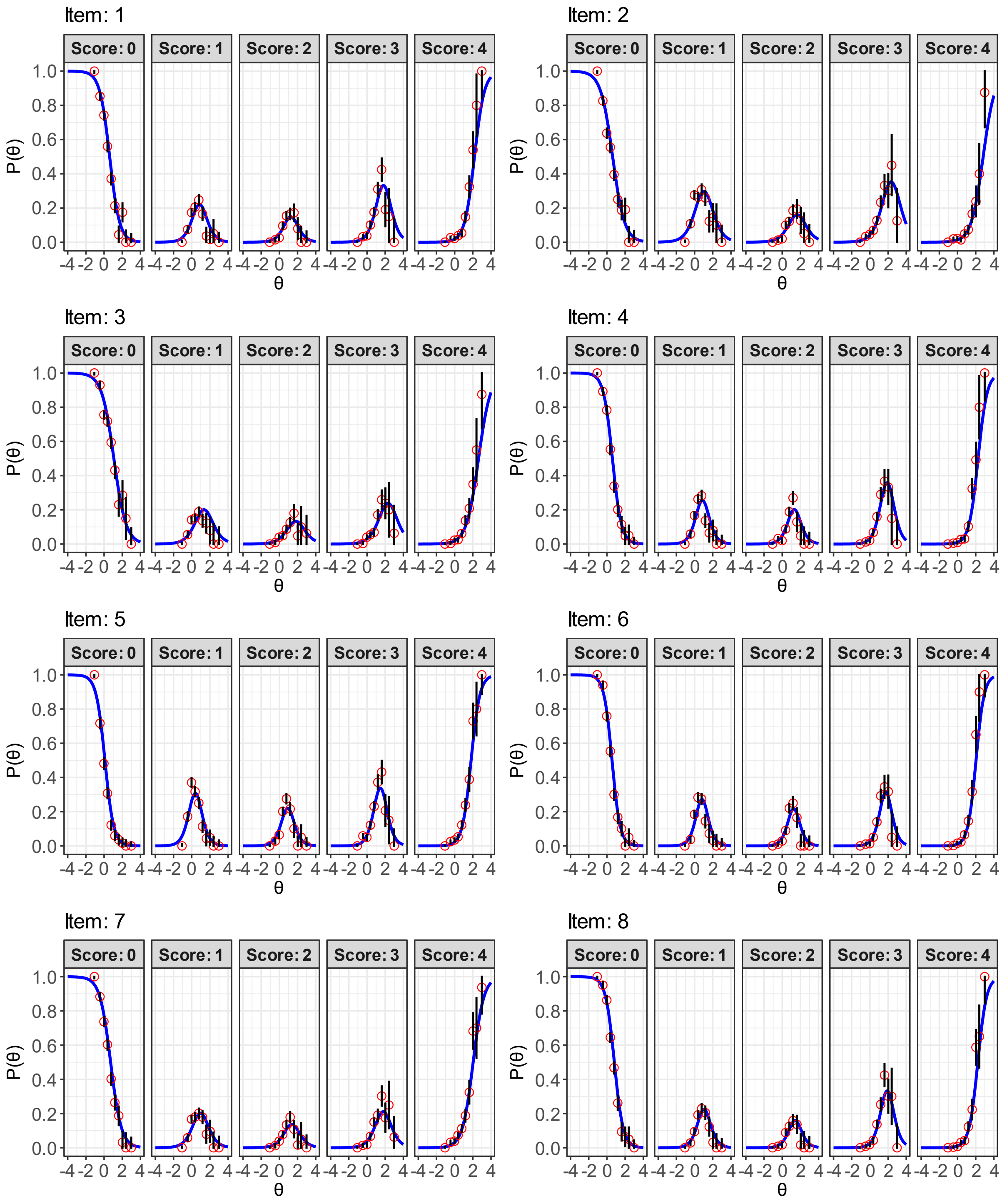

Notes. Response function and observed proportions with 95% CI. Items (1) *headache*, (2) *stomachache*, (3) *backache*, (4) *feeling low*, (5) *irritability/bad temper*, (6) *feeling nervous*, (7) *difficulties in getting to sleep*, (8) *feeling dizzy*.

Figure A34. Residual plots of the unidimensional GRM for LTU.

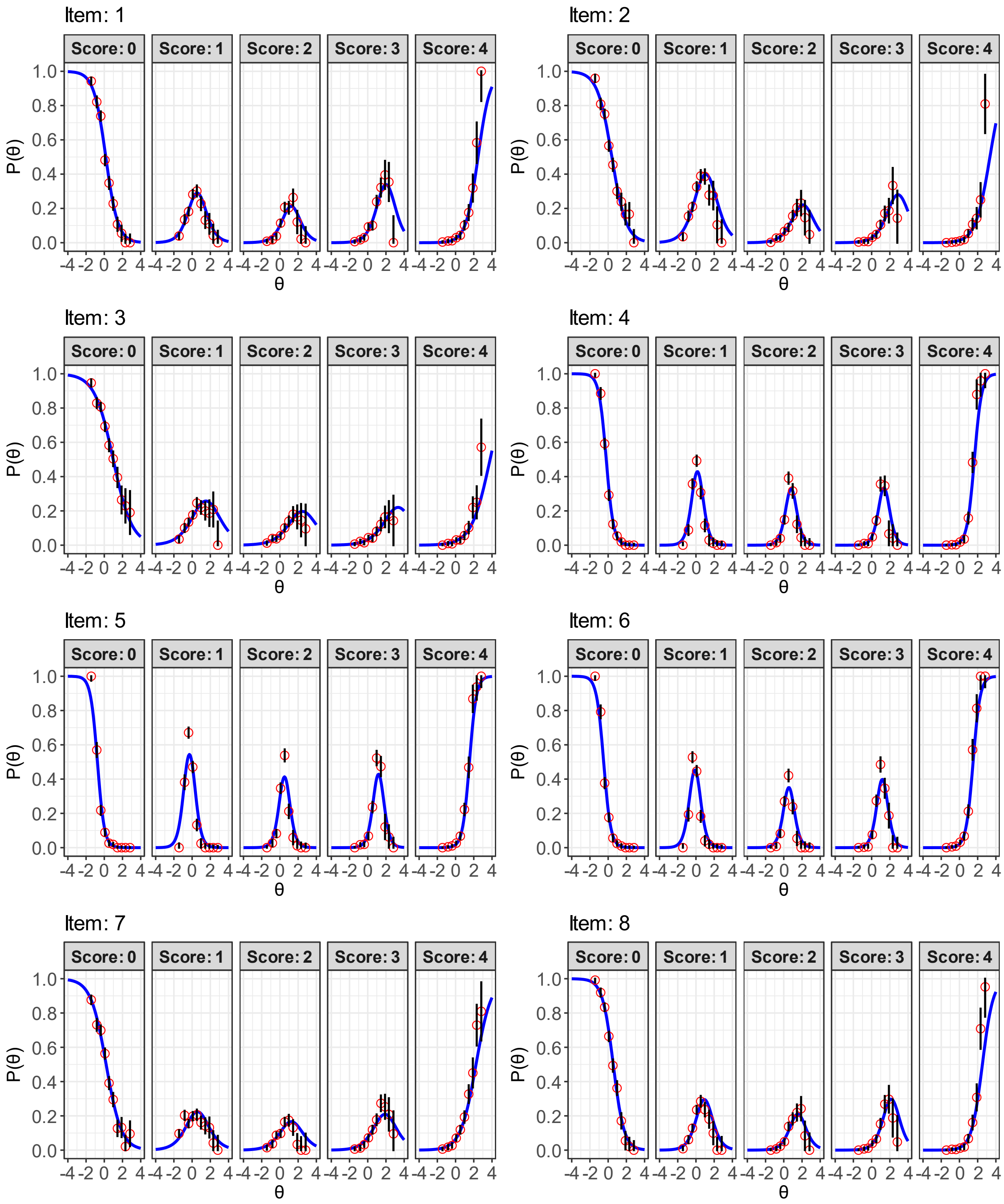

Notes. Response function and observed proportions with 95% CI. Items (1) *headache*, (2) *stomachache*, (3) *backache*, (4) *feeling low*, (5) *irritability/bad temper*, (6) *feeling nervous*, (7) *difficulties in getting to sleep*, (8) *feeling dizzy*.

Figure A35. Residual plots of the unidimensional GRM for LUX.

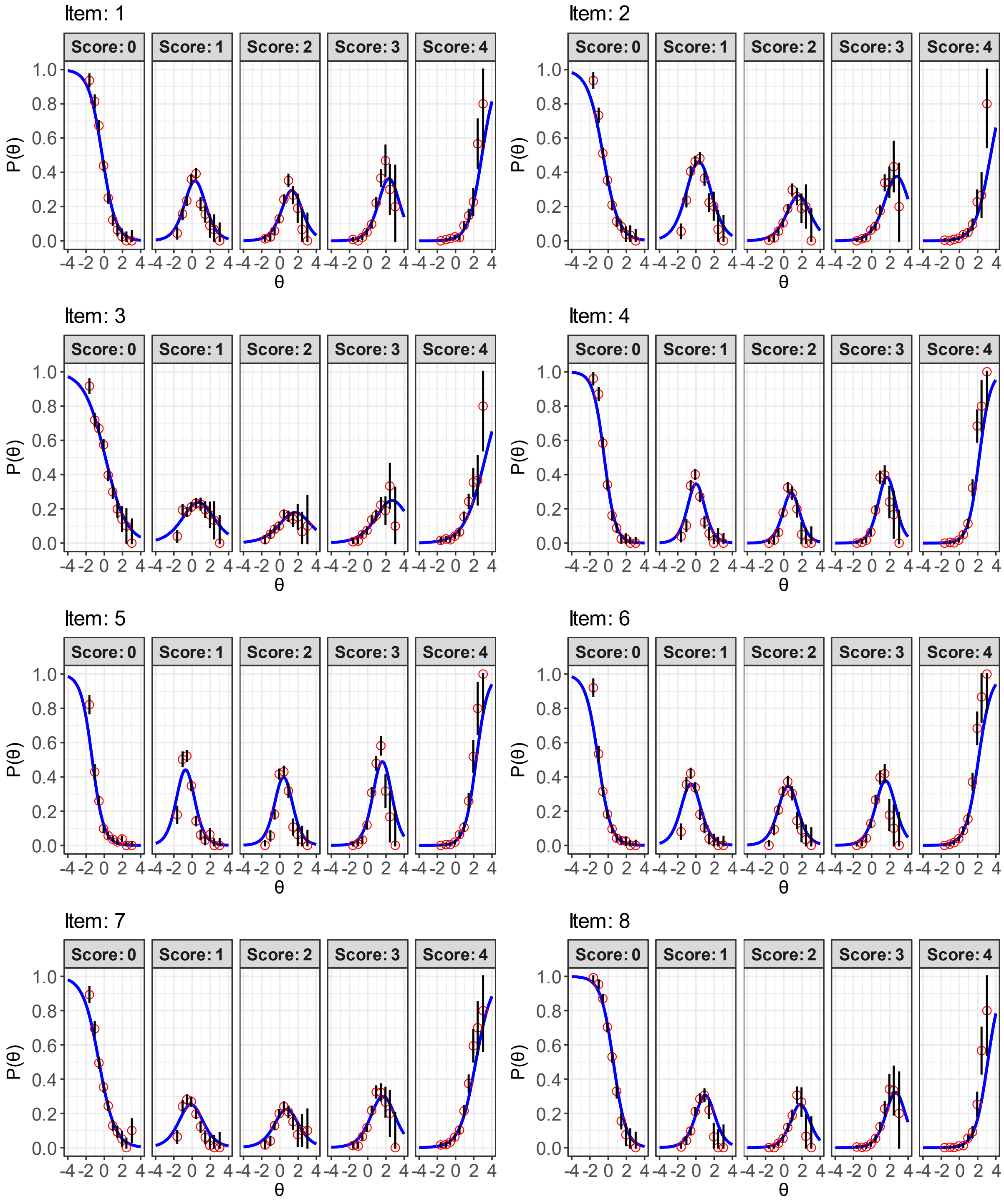

Notes. Response function and observed proportions with 95% CI. Items (1) *headache*, (2) *stomachache*, (3) *backache*, (4) *feeling low*, (5) *irritability/bad temper*, (6) *feeling nervous*, (7) *difficulties in getting to sleep*, (8) *feeling dizzy*.

Figure A36. Residual plots of the unidimensional GRM for LVA.

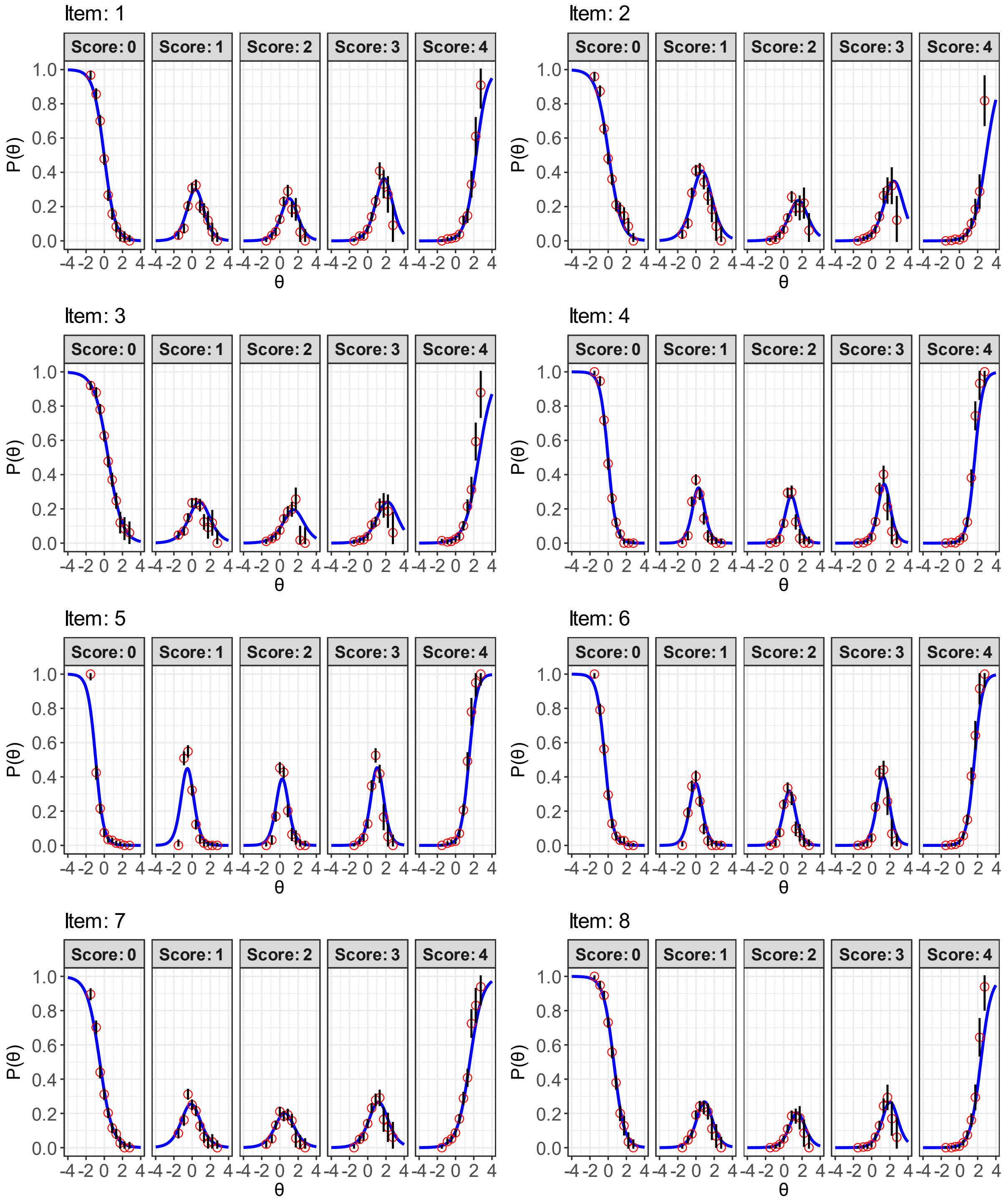

Notes. Response function and observed proportions with 95% CI. Items (1) *headache*, (2) *stomachache*, (3) *backache*, (4) *feeling low*, (5) *irritability/bad temper*, (6) *feeling nervous*, (7) *difficulties in getting to sleep*, (8) *feeling dizzy*.

Figure A37. Residual plots of the unidimensional GRM for MDA.

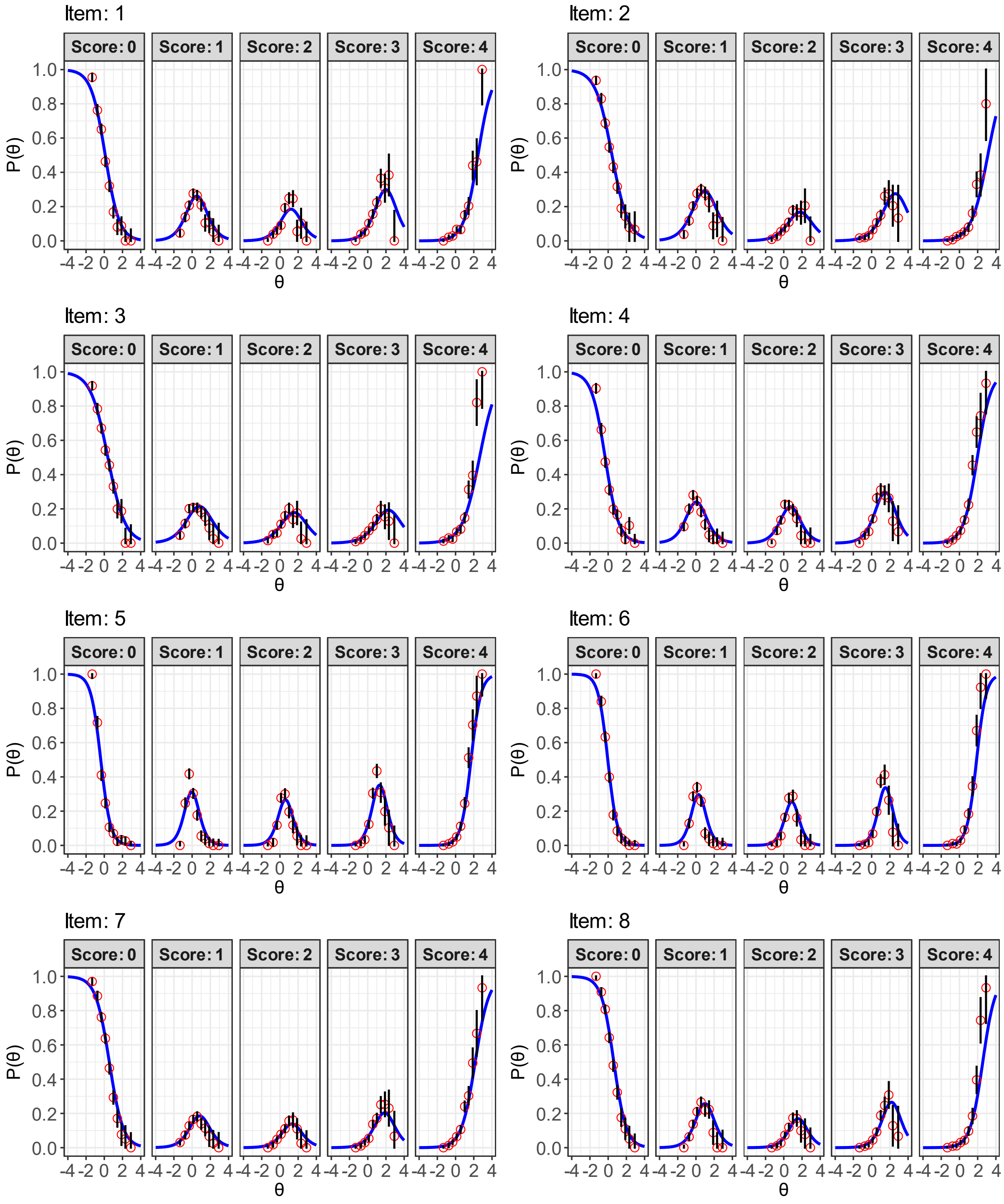

Notes. Response function and observed proportions with 95% CI. Items (1) *headache*, (2) *stomachache*, (3) *backache*, (4) *feeling low*, (5) *irritability/bad temper*, (6) *feeling nervous*, (7) *difficulties in getting to sleep*, (8) *feeling dizzy*.

Figure A38. Residual plots of the unidimensional GRM for MLT.

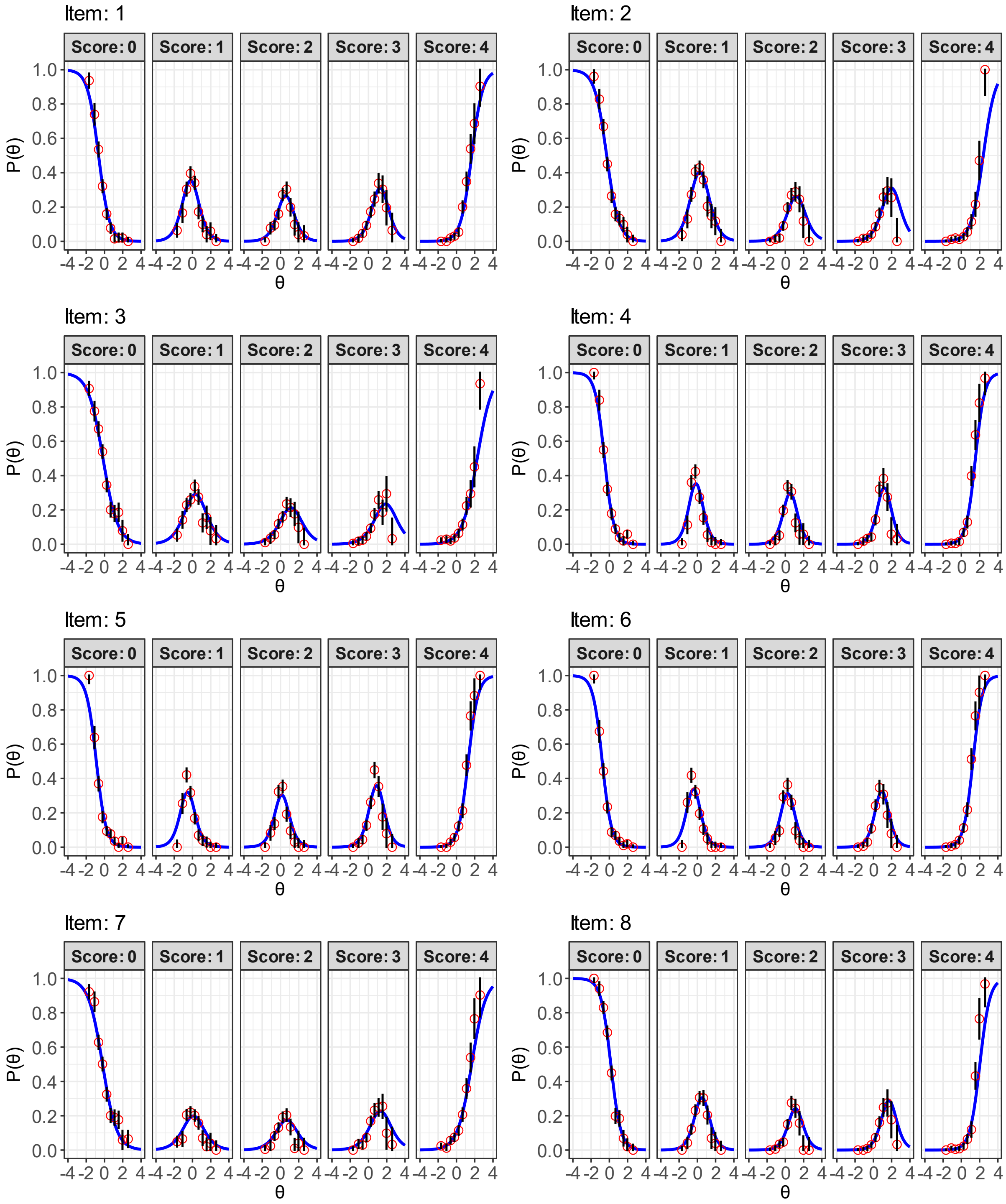

Notes. Response function and observed proportions with 95% CI. Items (1) *headache*, (2) *stomachache*, (3) *backache*, (4) *feeling low*, (5) *irritability/bad temper*, (6) *feeling nervous*, (7) *difficulties in getting to sleep*, (8) *feeling dizzy*.

Figure A39. Residual plots of the unidimensional GRM for NLD.

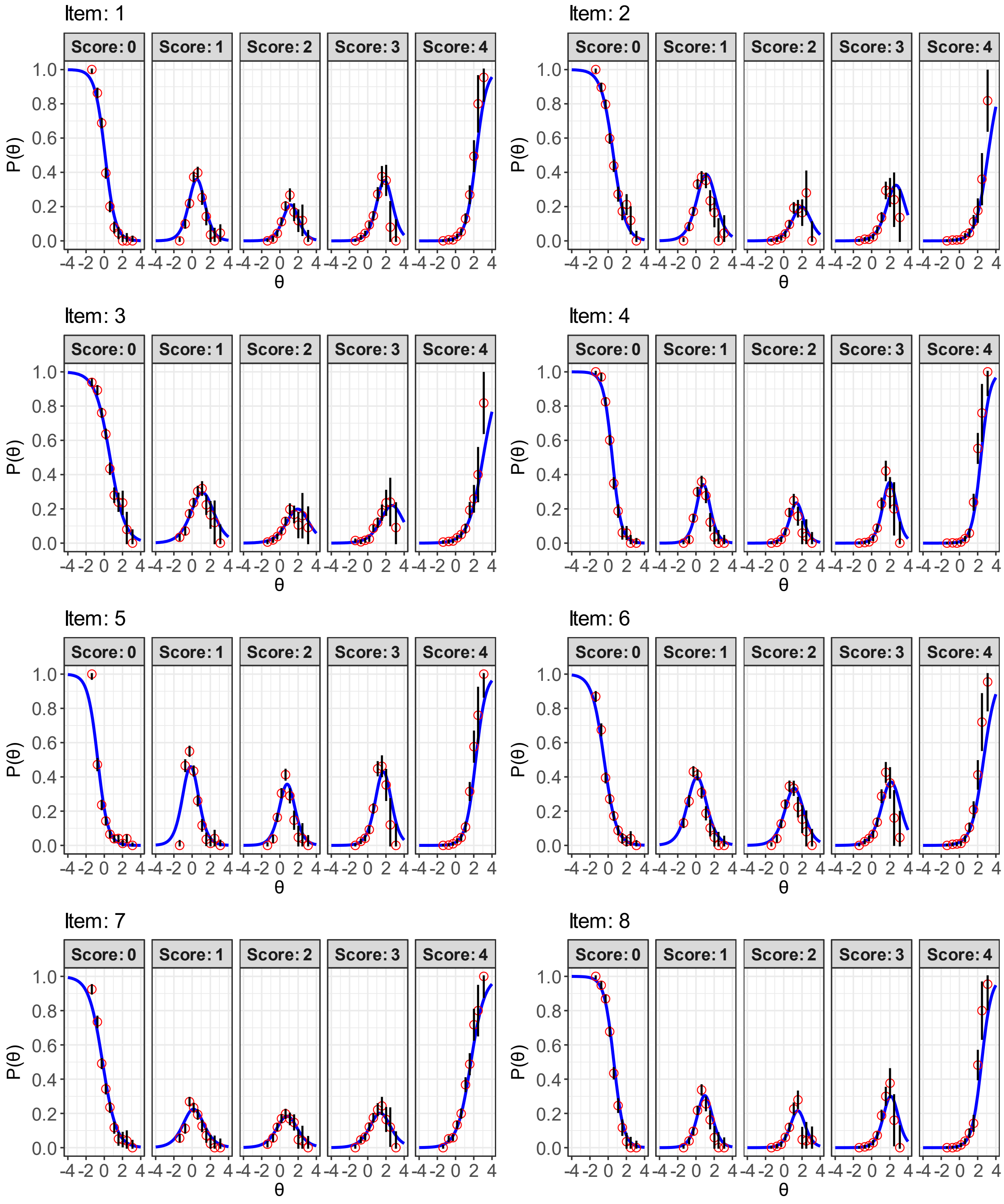

Notes. Response function and observed proportions with 95% CI. Items (1) *headache*, (2) *stomachache*, (3) *backache*, (4) *feeling low*, (5) *irritability/bad temper*, (6) *feeling nervous*, (7) *difficulties in getting to sleep*, (8) *feeling dizzy*.

Figure A40. Residual plots of the unidimensional GRM for NOR.

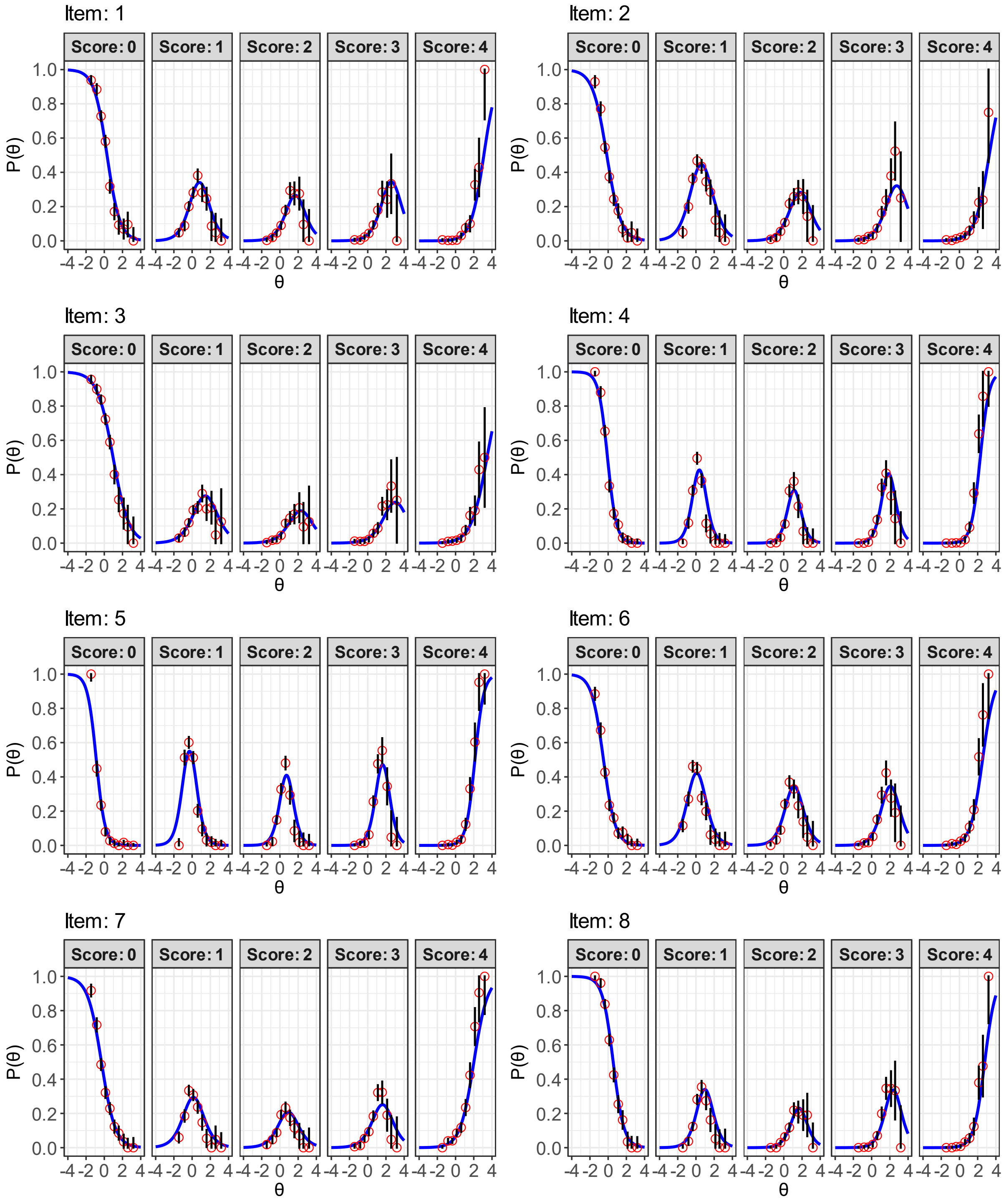

Notes. Response function and observed proportions with 95% CI. Items (1) *headache*, (2) *stomachache*, (3) *backache*, (4) *feeling low*, (5) *irritability/bad temper*, (6) *feeling nervous*, (7) *difficulties in getting to sleep*, (8) *feeling dizzy*.

Figure A41. Residual plots of the unidimensional GRM for POL.

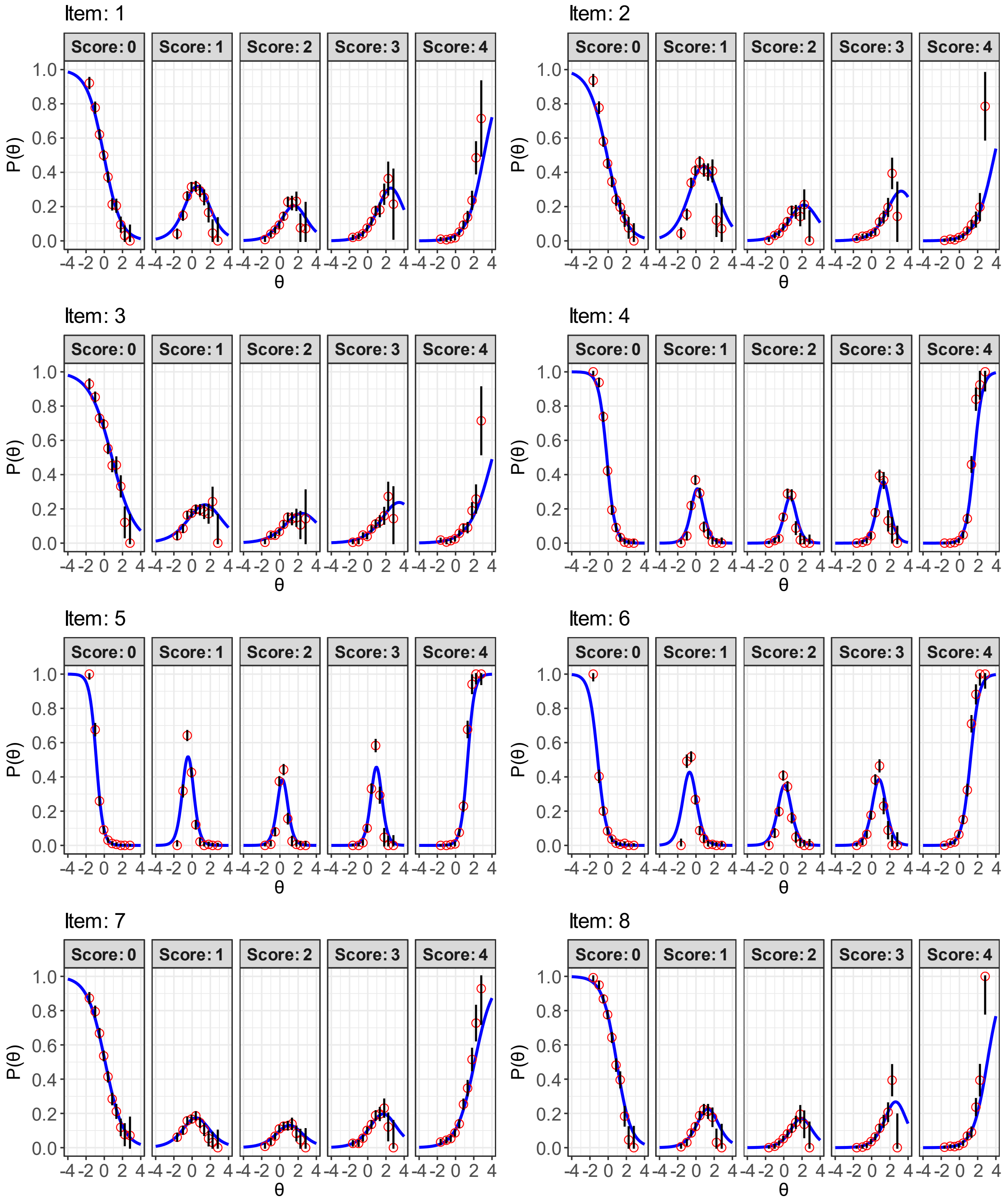

Notes. Response function and observed proportions with 95% CI. Items (1) *headache*, (2) *stomachache*, (3) *backache*, (4) *feeling low*, (5) *irritability/bad temper*, (6) *feeling nervous*, (7) *difficulties in getting to sleep*, (8) *feeling dizzy*.

Figure A42. Residual plots of the unidimensional GRM for PRT.

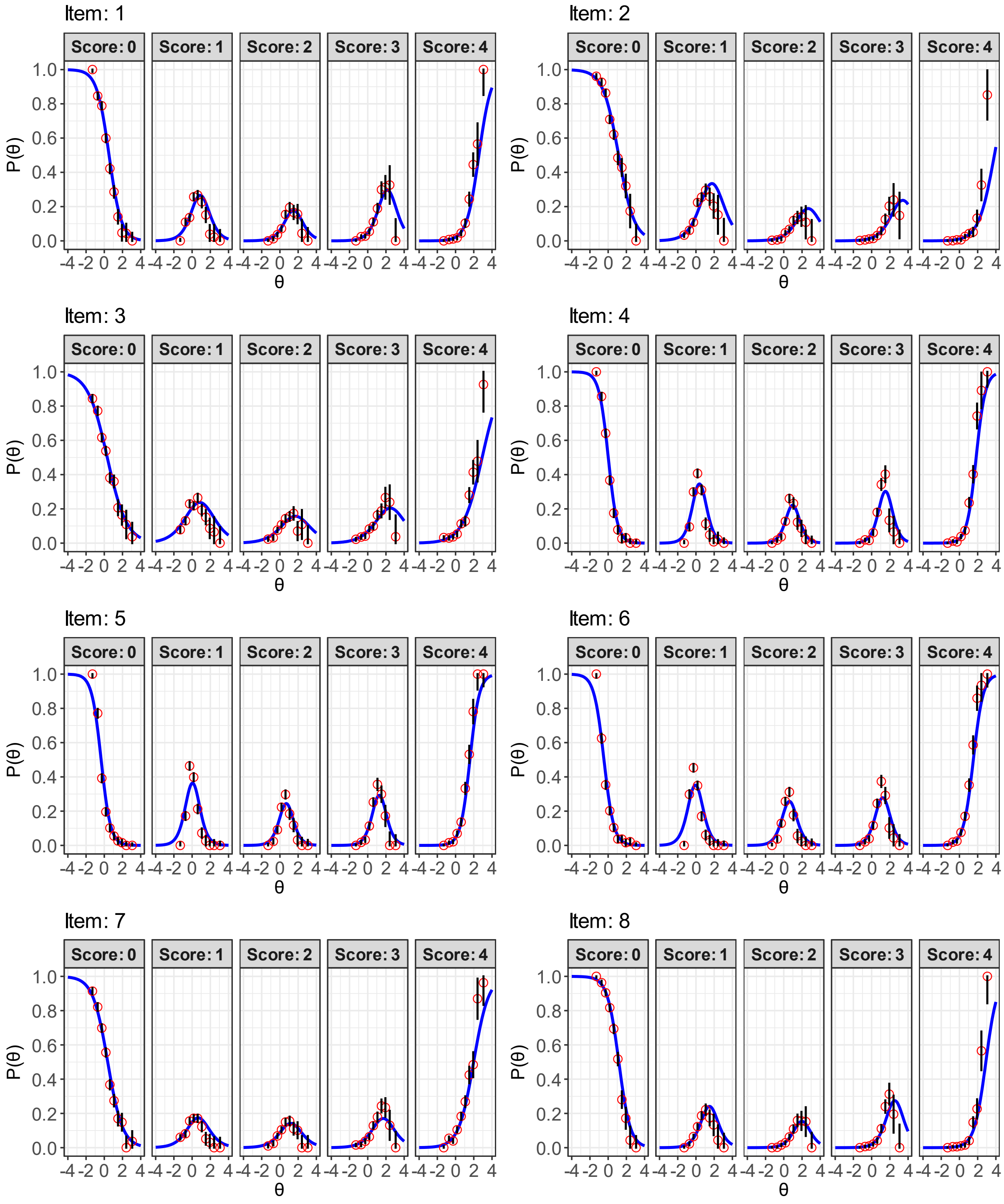

Notes. Response function and observed proportions with 95% CI. Items (1) *headache*, (2) *stomachache*, (3) *backache*, (4) *feeling low*, (5) *irritability/bad temper*, (6) *feeling nervous*, (7) *difficulties in getting to sleep*, (8) *feeling dizzy*.

Figure A43. Residual plots of the unidimensional GRM for ROU.

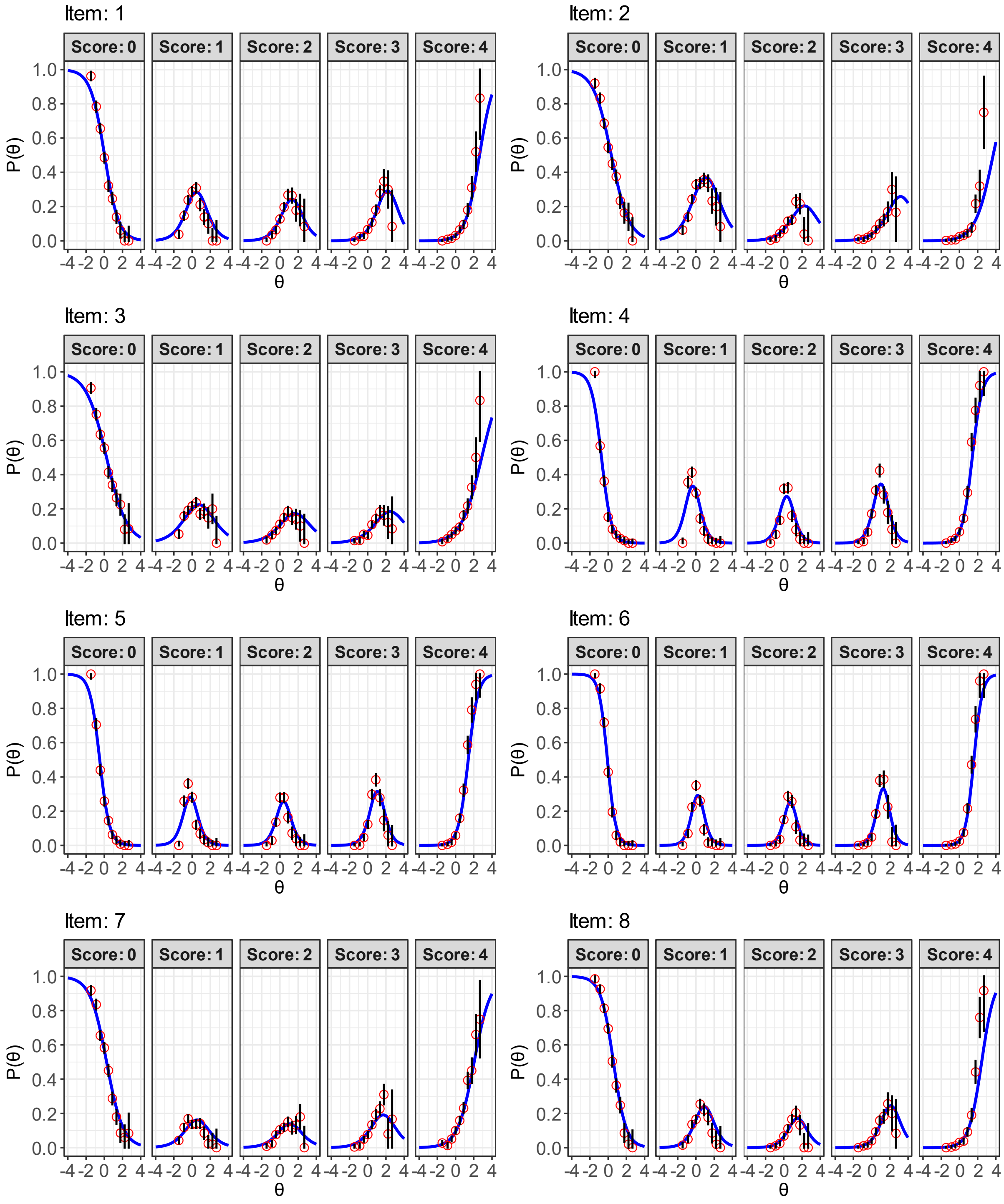

Notes. Response function and observed proportions with 95% CI. Items (1) *headache*, (2) *stomachache*, (3) *backache*, (4) *feeling low*, (5) *irritability/bad temper*, (6) *feeling nervous*, (7) *difficulties in getting to sleep*, (8) *feeling dizzy*.

Figure A44. Residual plots of the unidimensional GRM for RUS.

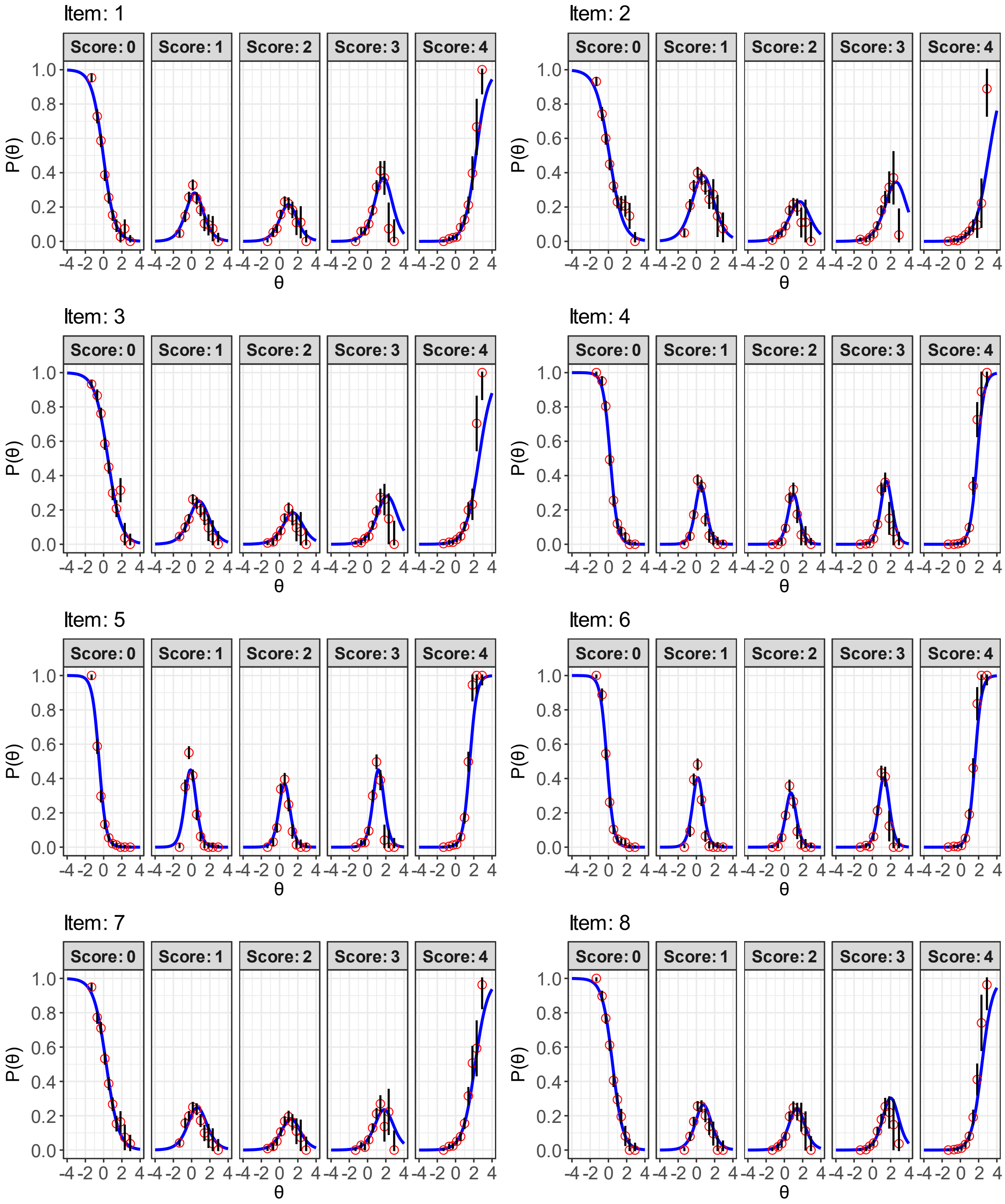

Notes. Response function and observed proportions with 95% CI. Items (1) *headache*, (2) *stomachache*, (3) *backache*, (4) *feeling low*, (5) *irritability/bad temper*, (6) *feeling nervous*, (7) *difficulties in getting to sleep*, (8) *feeling dizzy*.

Figure A45. Residual plots of the unidimensional GRM for SRB.

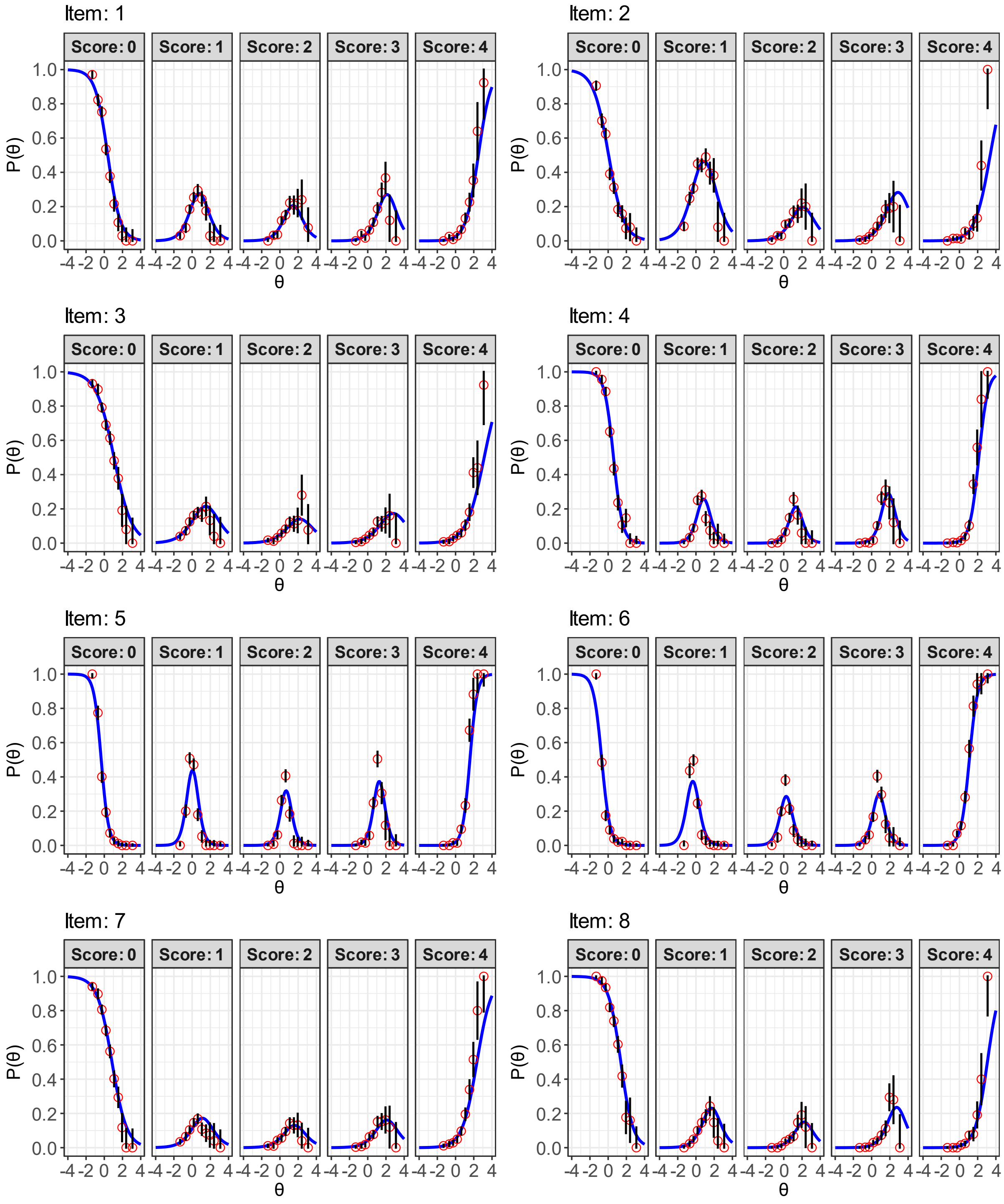

Notes. Response function and observed proportions with 95% CI. Items (1) *headache*, (2) *stomachache*, (3) *backache*, (4) *feeling low*, (5) *irritability/bad temper*, (6) *feeling nervous*, (7) *difficulties in getting to sleep*, (8) *feeling dizzy*.

Figure A46. Residual plots of the unidimensional GRM for SVK.

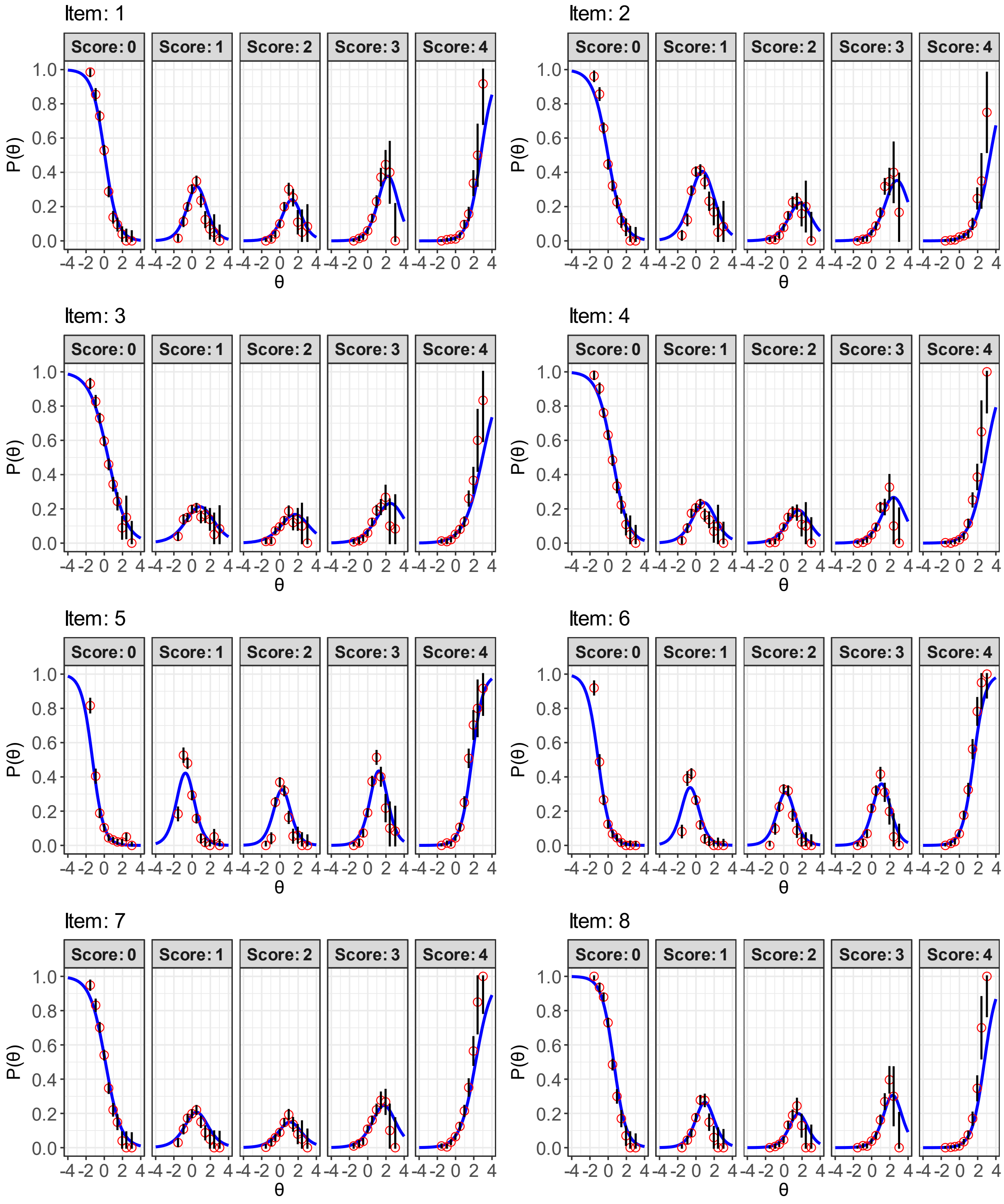

Notes. Response function and observed proportions with 95% CI. Items (1) *headache*, (2) *stomachache*, (3) *backache*, (4) *feeling low*, (5) *irritability/bad temper*, (6) *feeling nervous*, (7) *difficulties in getting to sleep*, (8) *feeling dizzy*.

Figure A47. Residual plots of the unidimensional GRM for SVN.

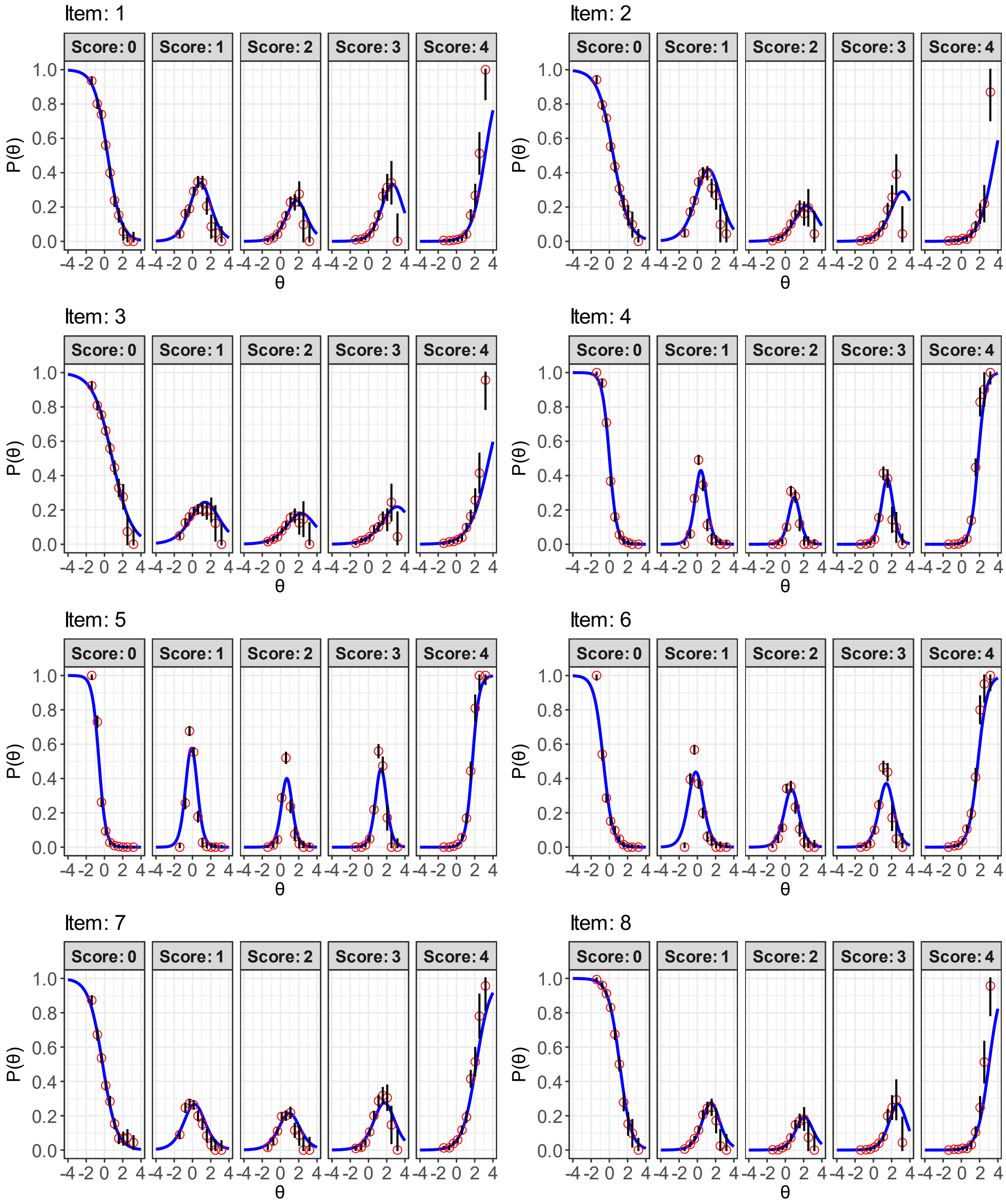

Notes. Response function and observed proportions with 95% CI. Items (1) *headache*, (2) *stomachache*, (3) *backache*, (4) *feeling low*, (5) *irritability/bad temper*, (6) *feeling nervous*, (7) *difficulties in getting to sleep*, (8) *feeling dizzy*.

Figure A48. Residual plots of the unidimensional GRM for SWE.

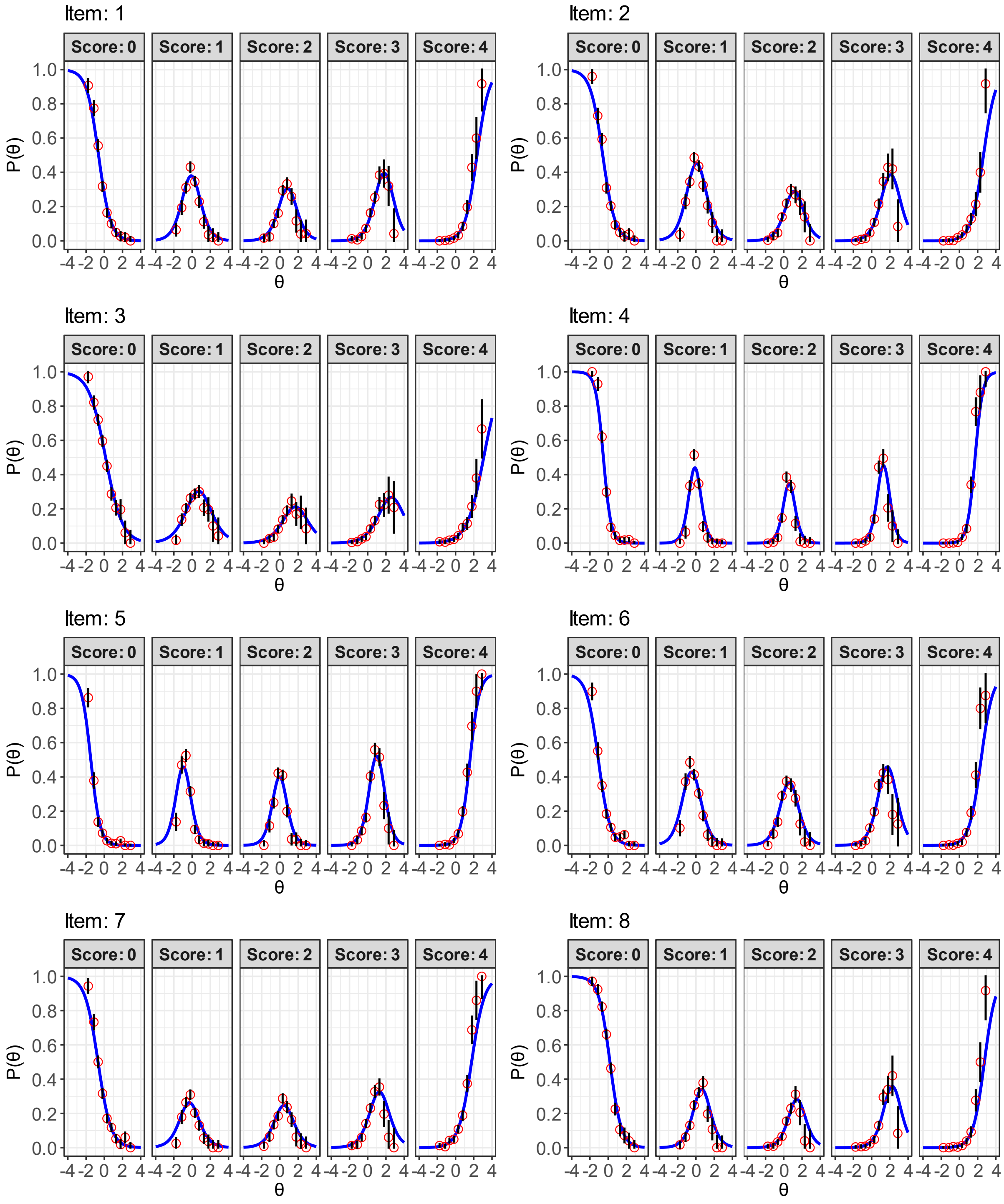

Notes. Response function and observed proportions with 95% CI. Items (1) *headache*, (2) *stomachache*, (3) *backache*, (4) *feeling low*, (5) *irritability/bad temper*, (6) *feeling nervous*, (7) *difficulties in getting to sleep*, (8) *feeling dizzy*.

Figure A49. Residual plots of the unidimensional GRM for TUR.

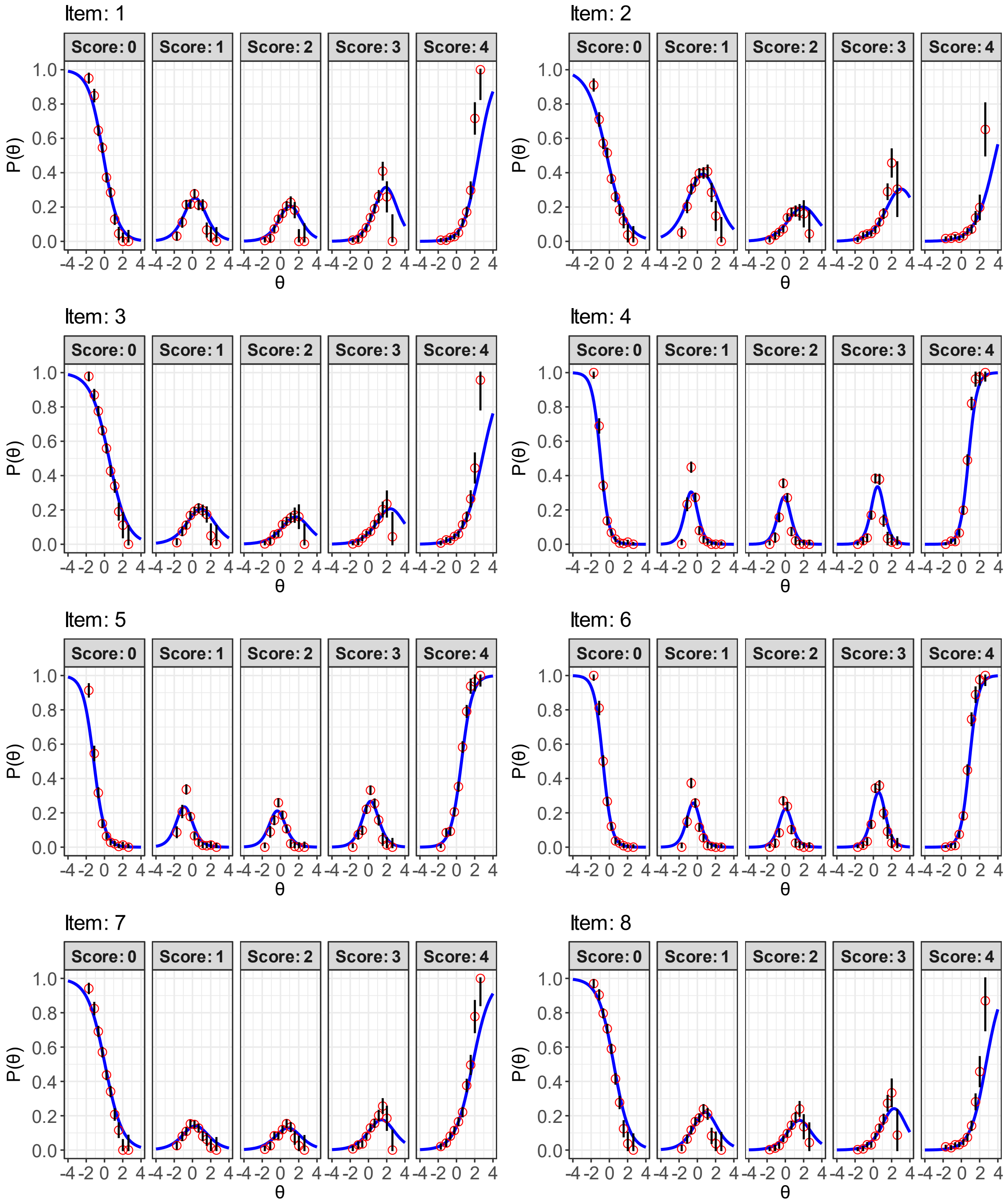

Notes. Response function and observed proportions with 95% CI. Items (1) *headache*, (2) *stomachache*, (3) *backache*, (4) *feeling low*, (5) *irritability/bad temper*, (6) *feeling nervous*, (7) *difficulties in getting to sleep*, (8) *feeling dizzy*.

Figure A50. Residual plots of the unidimensional GRM for UKR.

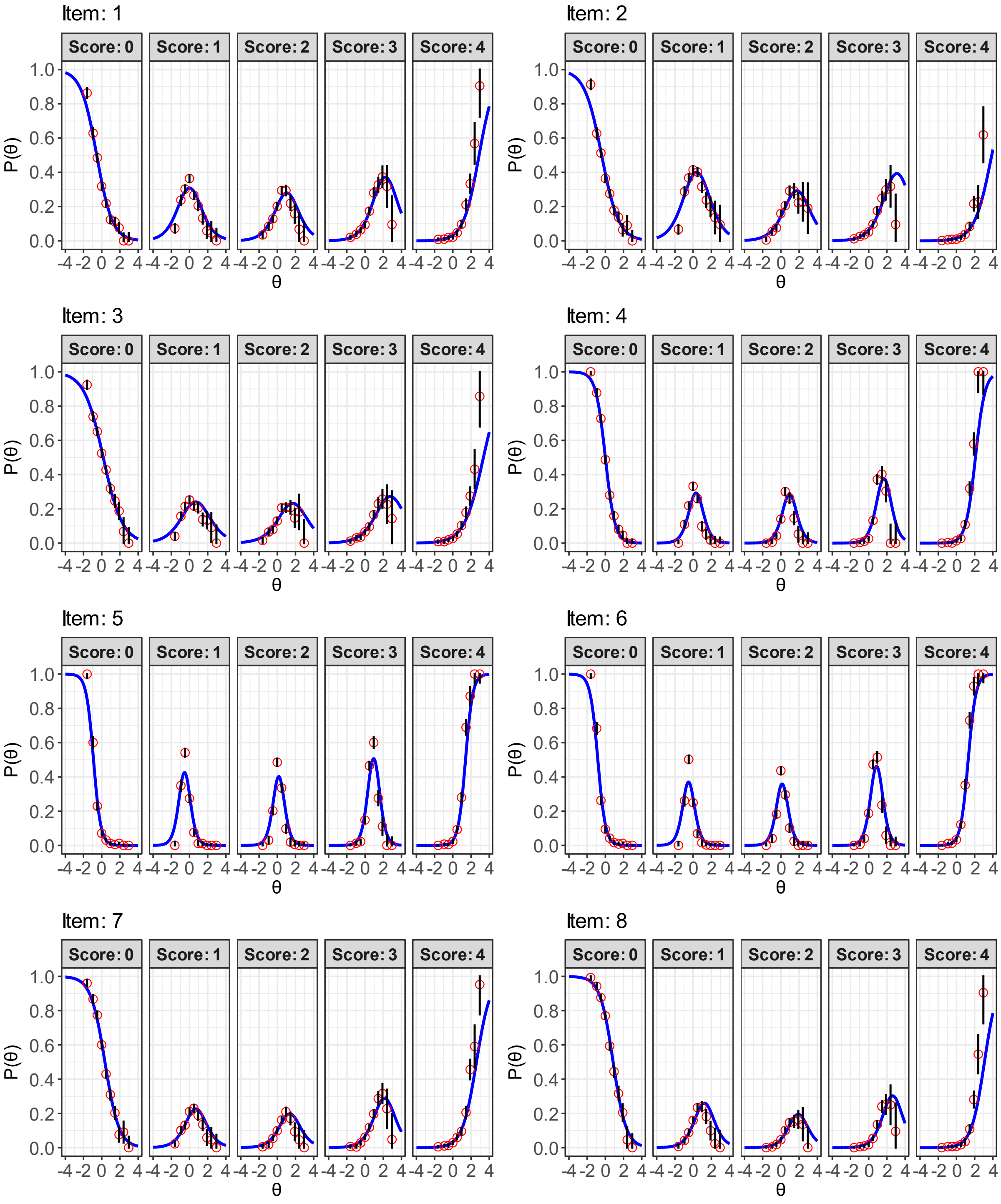

Notes. Response function and observed proportions with 95% CI. Items (1) *headache*, (2) *stomachache*, (3) *backache*, (4) *feeling low*, (5) *irritability/bad temper*, (6) *feeling nervous*, (7) *difficulties in getting to sleep*, (8) *feeling dizzy*.

Figure A51. Item characteristic curves of the unidimensional GRM.

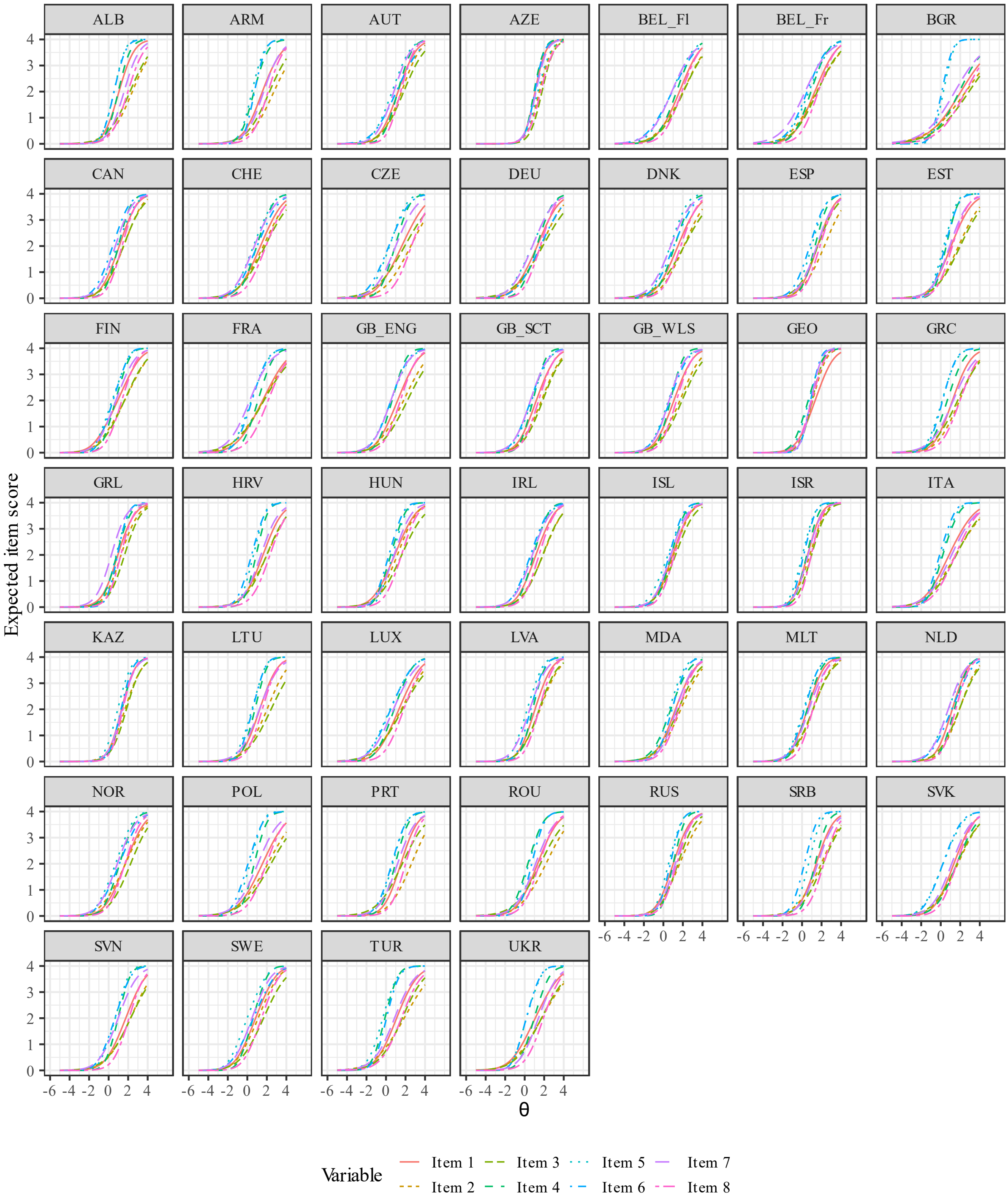

Notes. Items (1) *headache*, (2) *stomachache*, (3) *backache*, (4) *feeling low*, (5) *irritability/bad temper*, (6) *feeling nervous*, (7) *difficulties in getting to sleep*, (8) *feeling dizzy*.

Figure A52. Test characteristic curves of the unidimensional GRM.

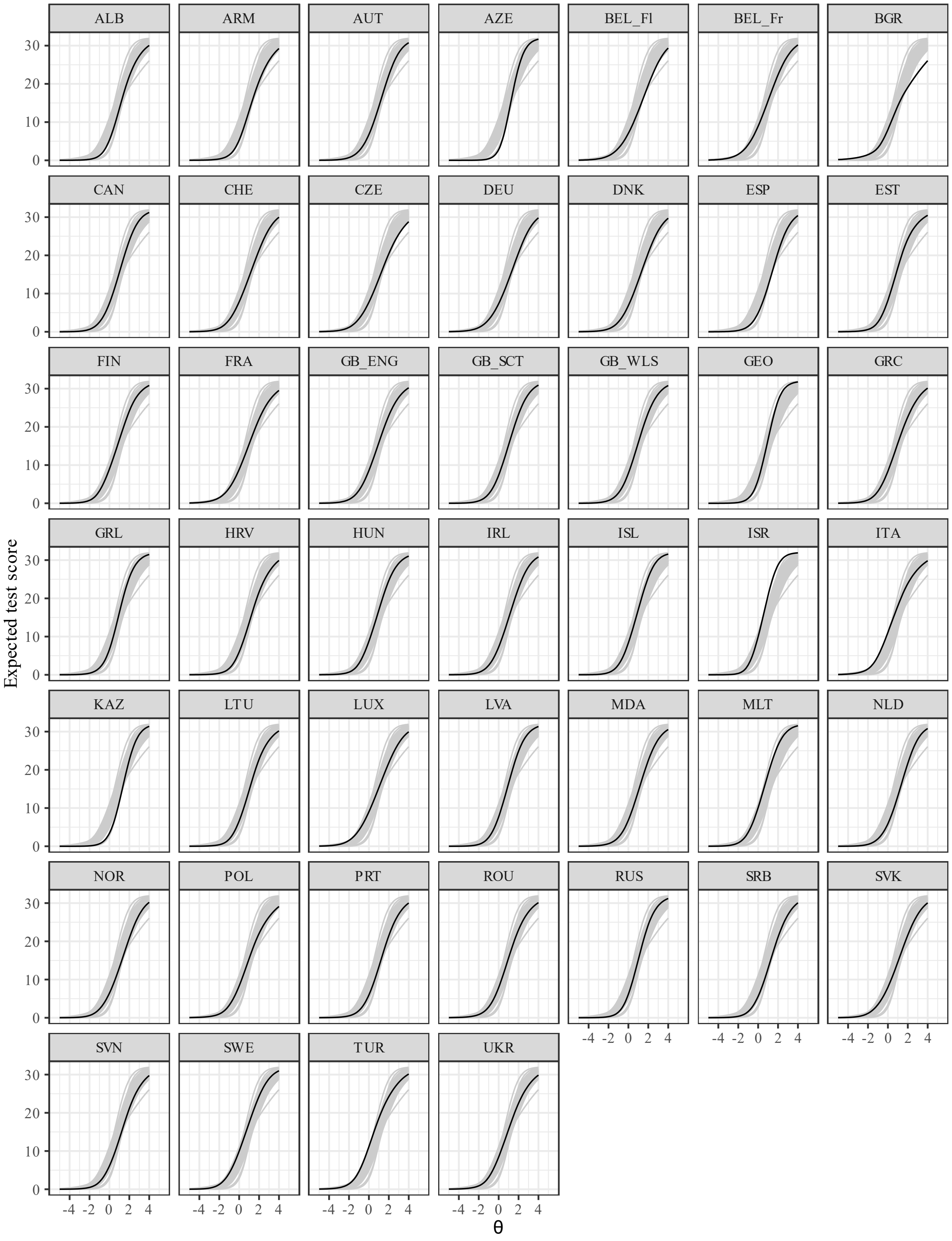

Notes. The black line represents the test characteristic curve in the respective country whereas the grey lines indicate the test characteristic curves in the remaining countries.

Figure A53. Item information functions of the unidimensional GRM.

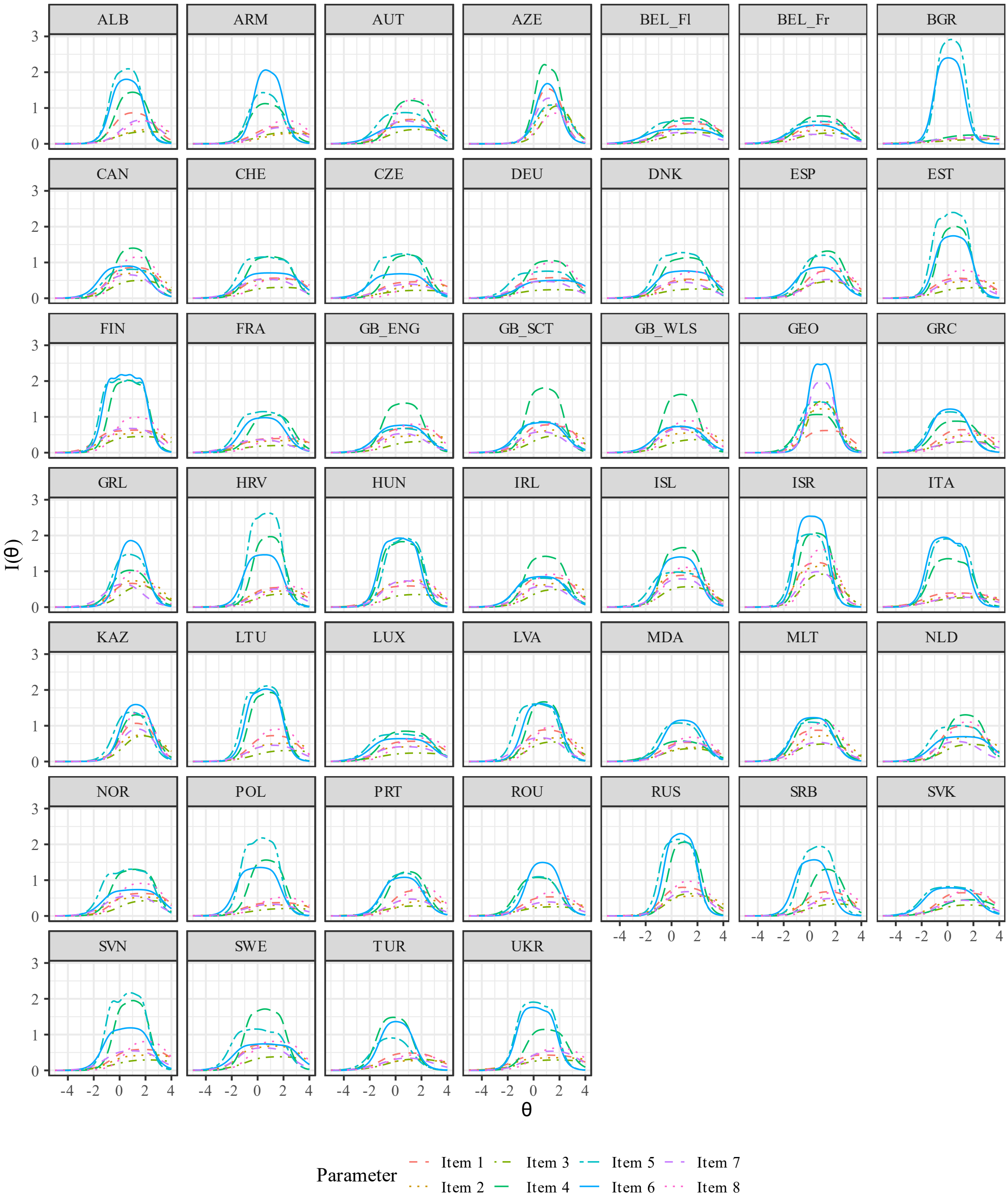

Notes. Items (1) *headache*, (2) *stomachache*, (3) *backache*, (4) *feeling low*, (5) *irritability/bad temper*, (6) *feeling nervous*, (7) *difficulties in getting to sleep*, (8) *feeling dizzy*.

Figure A54. Person-item map for the unidimensional GRM (ALB-DNK).

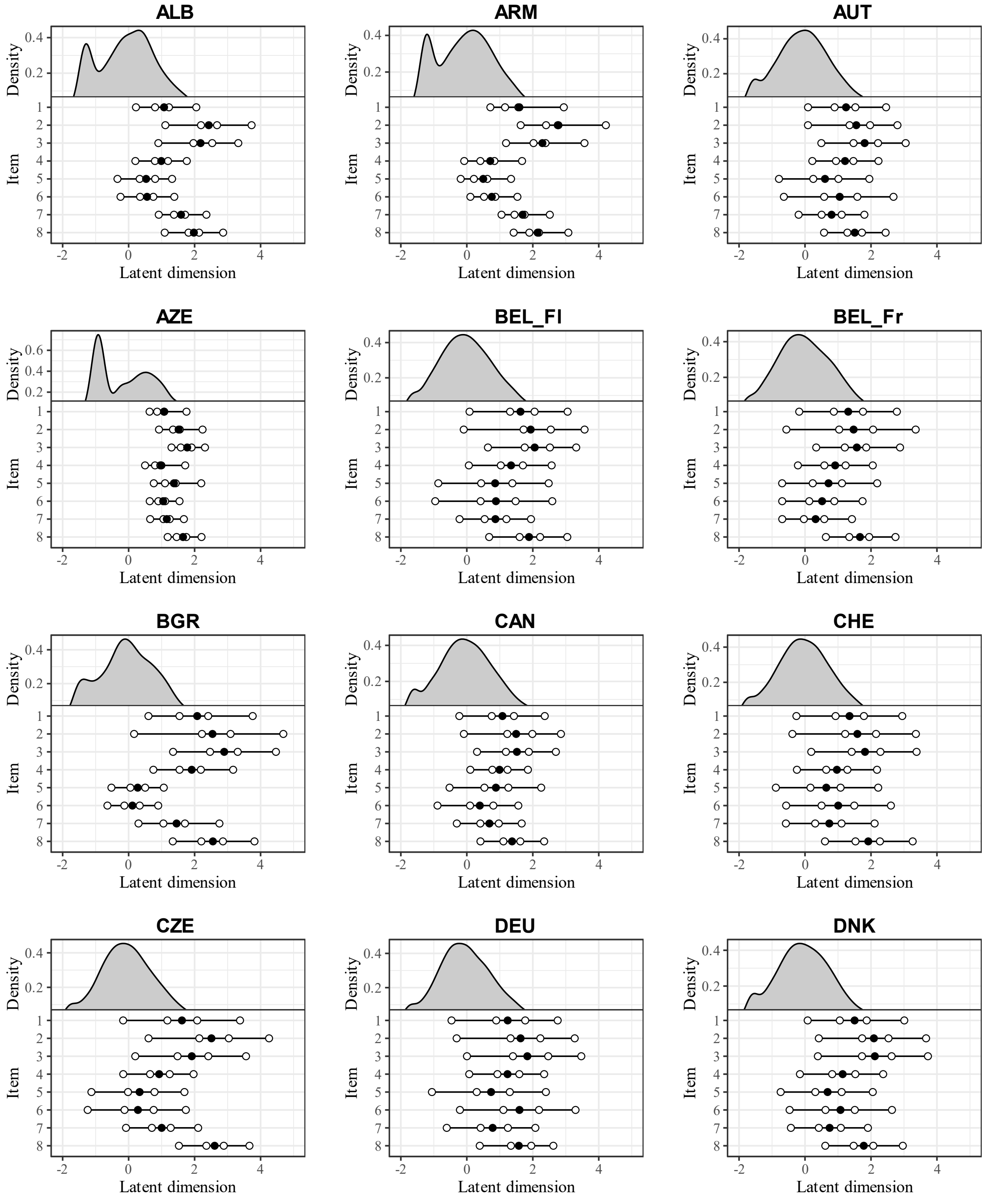

Notes. The plots show the distribution of the factor scores (above) and the item threshold parameters (below) estimated by the unidimensional GRM. Items (1) *headache*, (2) *stomachache*, (3) *backache*, (4) *feeling low*, (5) *irritability/bad temper*, (6) *feeling nervous*, (7) *difficulties in getting to sleep*, (8) *feeling dizzy*.

Figure A55. Person-item map for the unidimensional GRM (ESP-HUN).

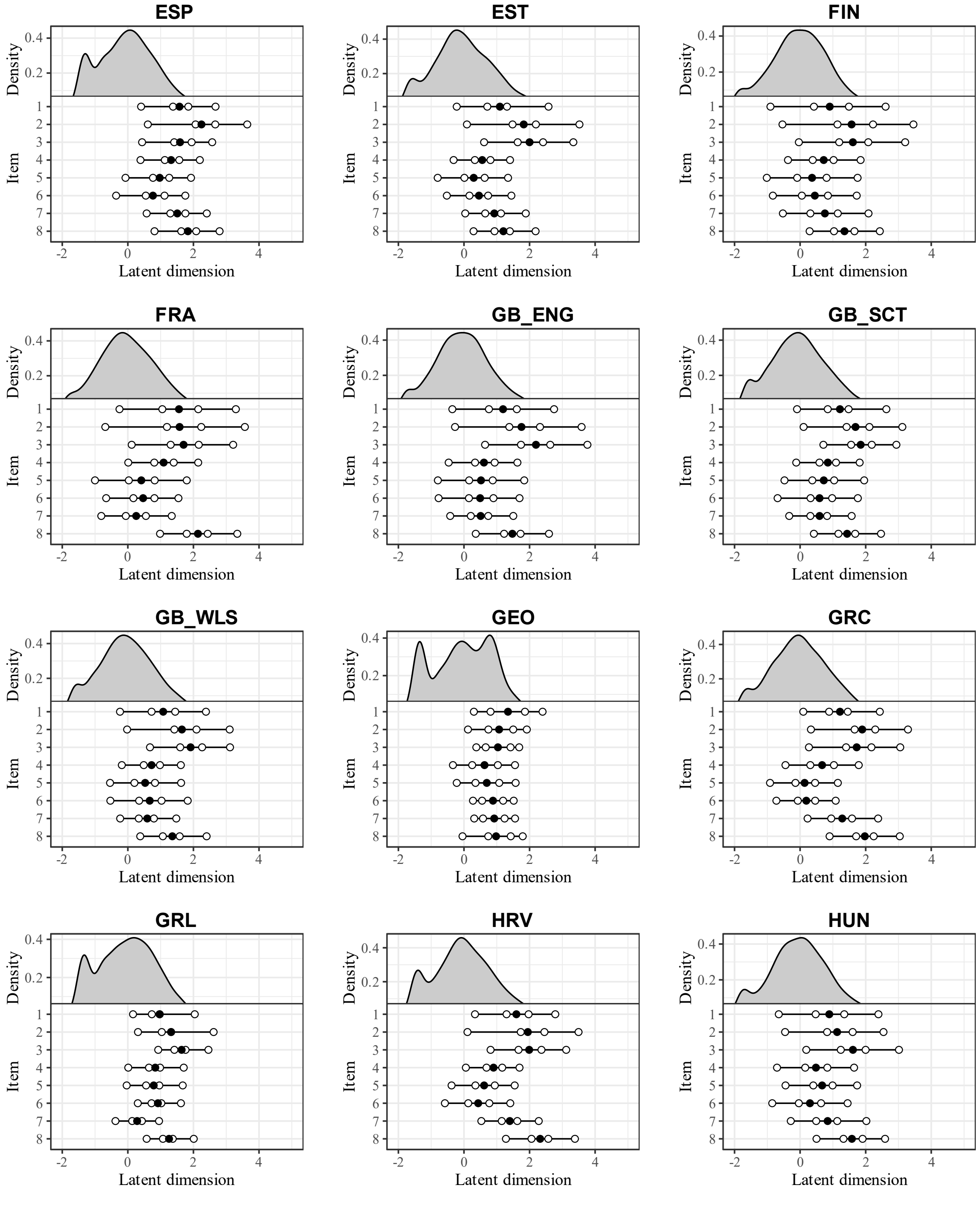

Notes. The plots show the distribution of the factor scores (above) and the item threshold parameters (below) estimated by the unidimensional GRM. Items (1) *headache*, (2) *stomachache*, (3) *backache*, (4) *feeling low*, (5) *irritability/bad temper*, (6) *feeling nervous*, (7) *difficulties in getting to sleep*, (8) *feeling dizzy*.

Figure A56. Person-item map for the unidimensional GRM (IRL-NOR).

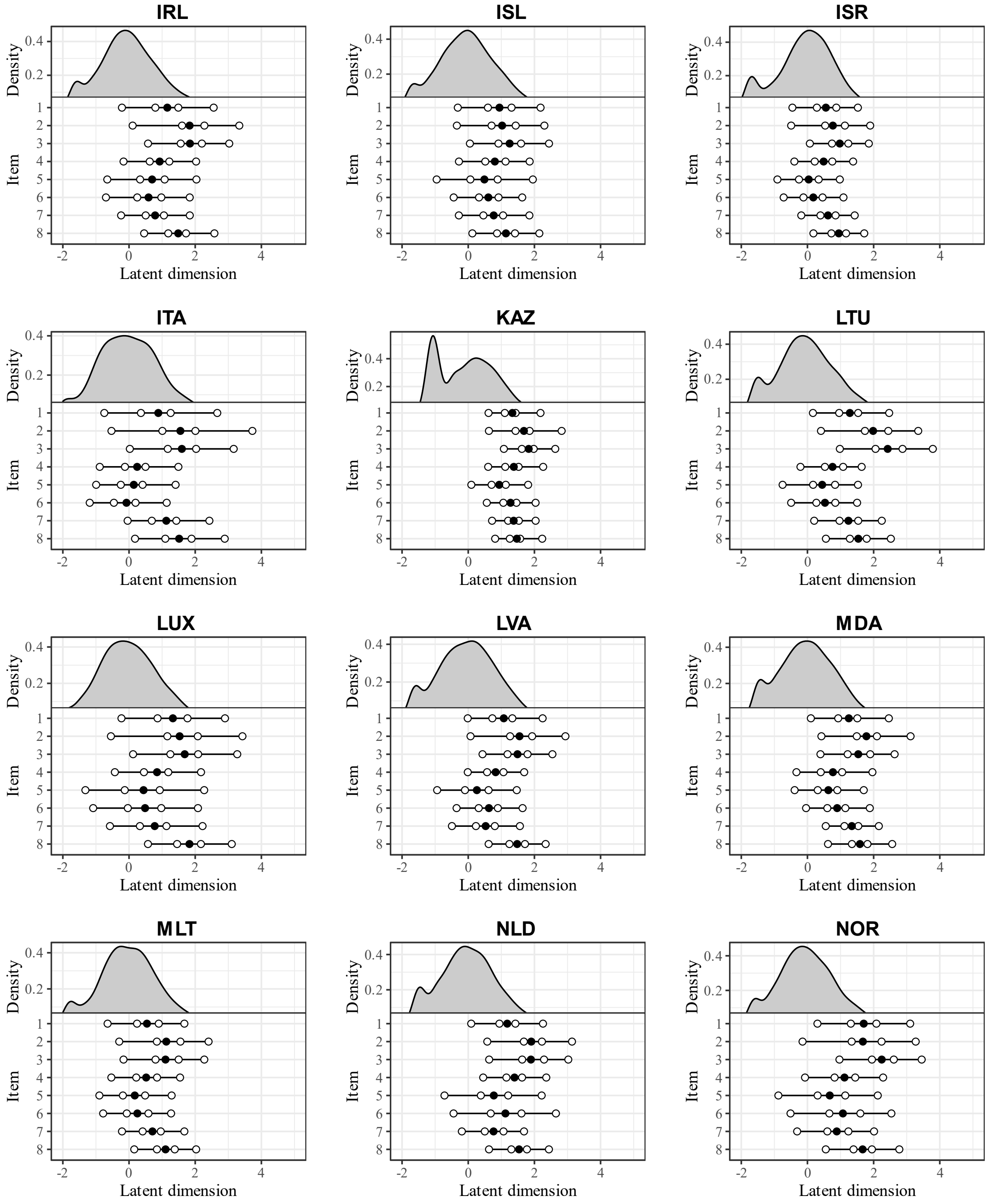

Notes. The plots show the distribution of the factor scores (above) and the item threshold parameters (below) estimated by the unidimensional GRM. Items (1) *headache*, (2) *stomachache*, (3) *backache*, (4) *feeling low*, (5) *irritability/bad temper*, (6) *feeling nervous*, (7) *difficulties in getting to sleep*, (8) *feeling dizzy*.

Figure A57. Person-item map for the unidimensional GRM (POL-UKR).

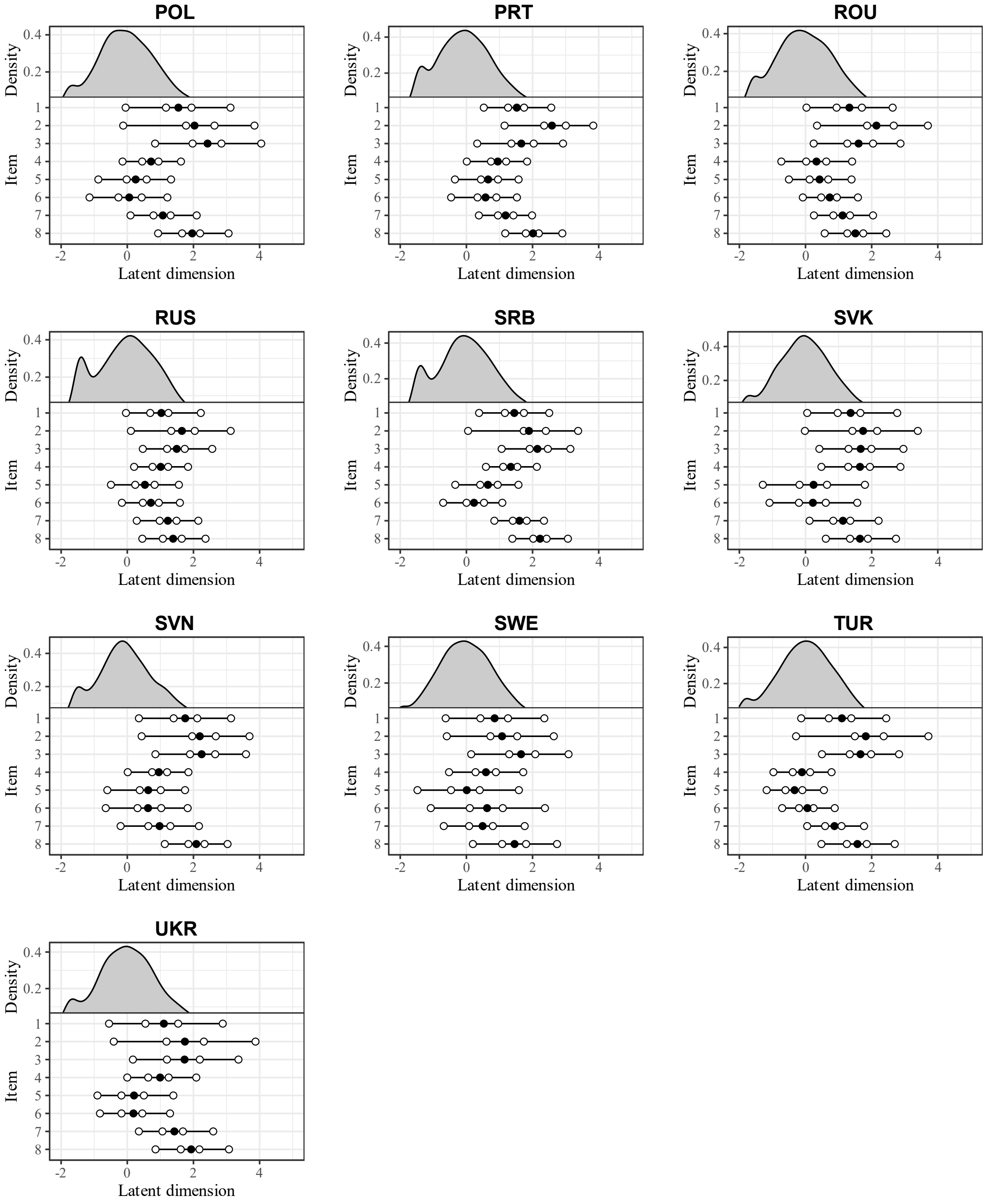

Notes. The plots show the distribution of the factor scores (above) and the item threshold parameters (below) estimated by the unidimensional GRM. Items (1) *headache*, (2) *stomachache*, (3) *backache*, (4) *feeling low*, (5) *irritability/bad temper*, (6) *feeling nervous*, (7) *difficulties in getting to sleep*, (8) *feeling dizzy*.

Figure A58. Comparing local dependency between items for the unidimensional and the bifactor GRM.

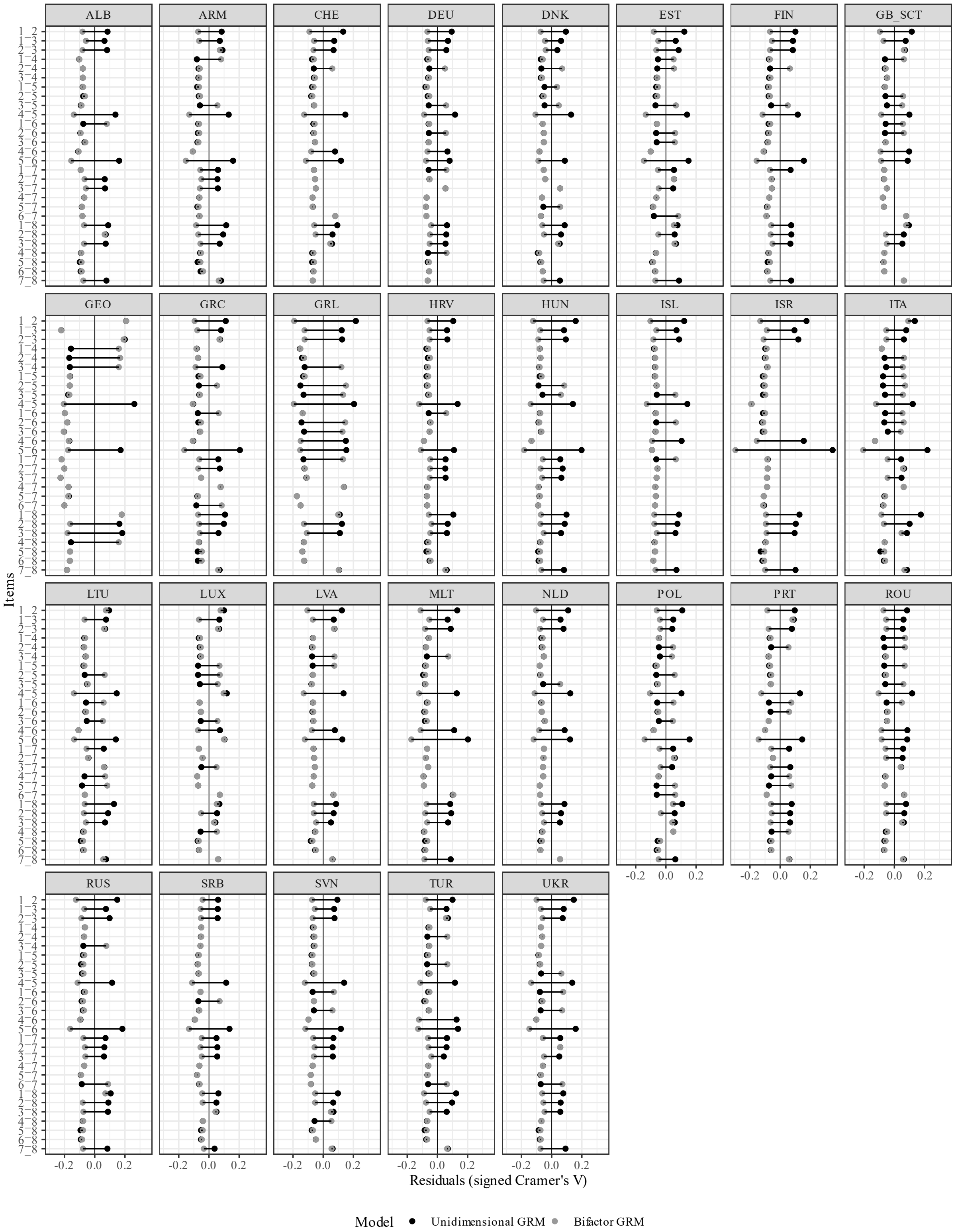

Notes. Values represent standardized residuals between items in terms of Cramer's V. Items (1) *headache*, (2) *stomachache*, (3) *backache*, (4) *feeling low*, (5) *irritability/bad temper*, (6) *feeling nervous,* (7) *difficulties in getting to sleep*, (8) *feeling dizzy*.

Figure A59. Comparing item fit between the unidimensional and the bifactor GRM.

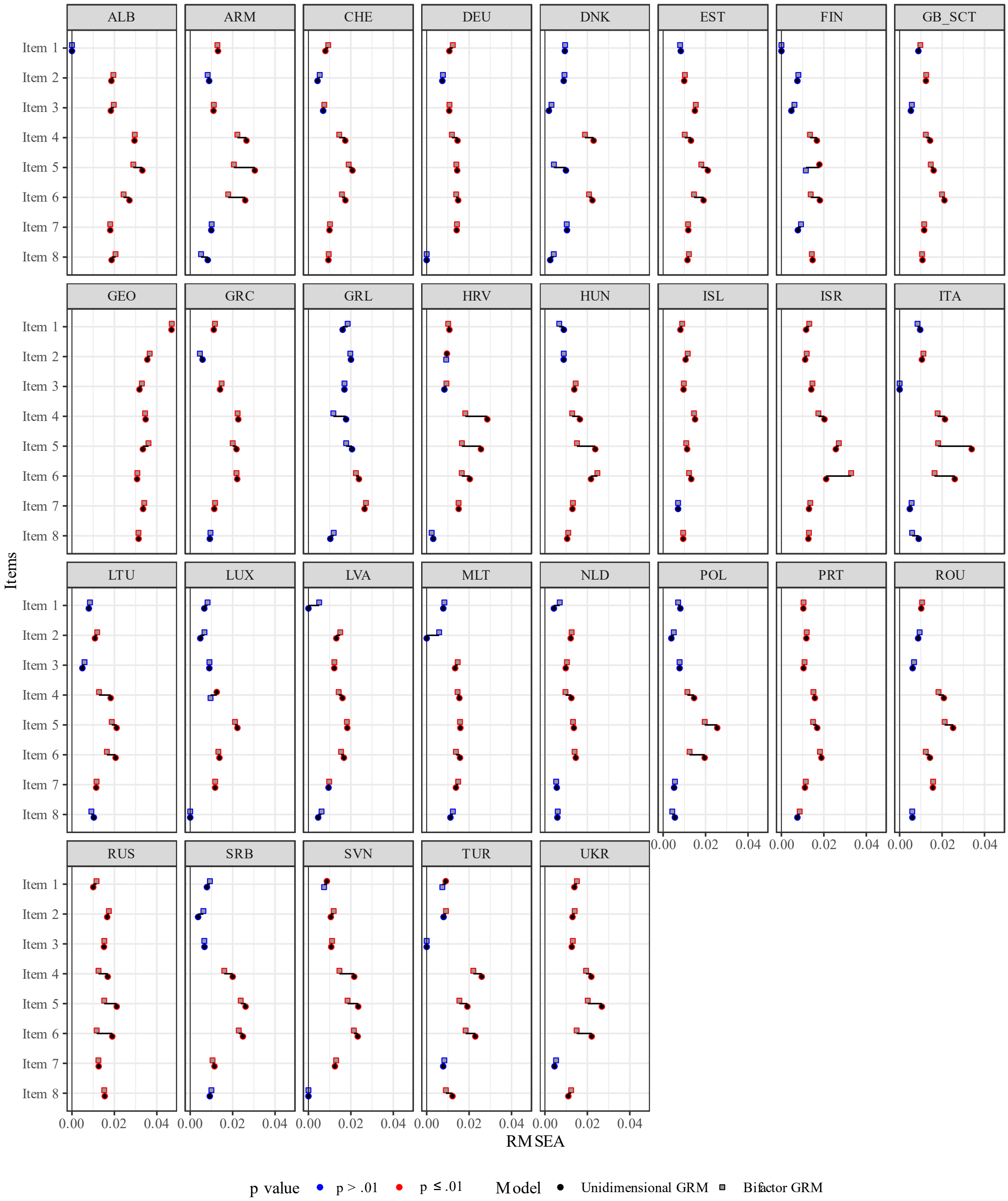

Notes. Values represent the item-level *RMSEA*. The significance test is based on the generalized S-X² statistic for polytomous items with adjusted p values based on the Benjamini-Hochberg (B-H) procedure. Items (1) *headache*, (2) *stomachache*, (3) *backache*, (4) *feeling low*, (5) *irritability/bad temper*, (6) *feeling nervous*, (7) *difficulties in getting to sleep*, (8) *feeling dizzy*.

Figure A60. Comparing item discrimination parameters between the unidimensional and the bifactor GRM.

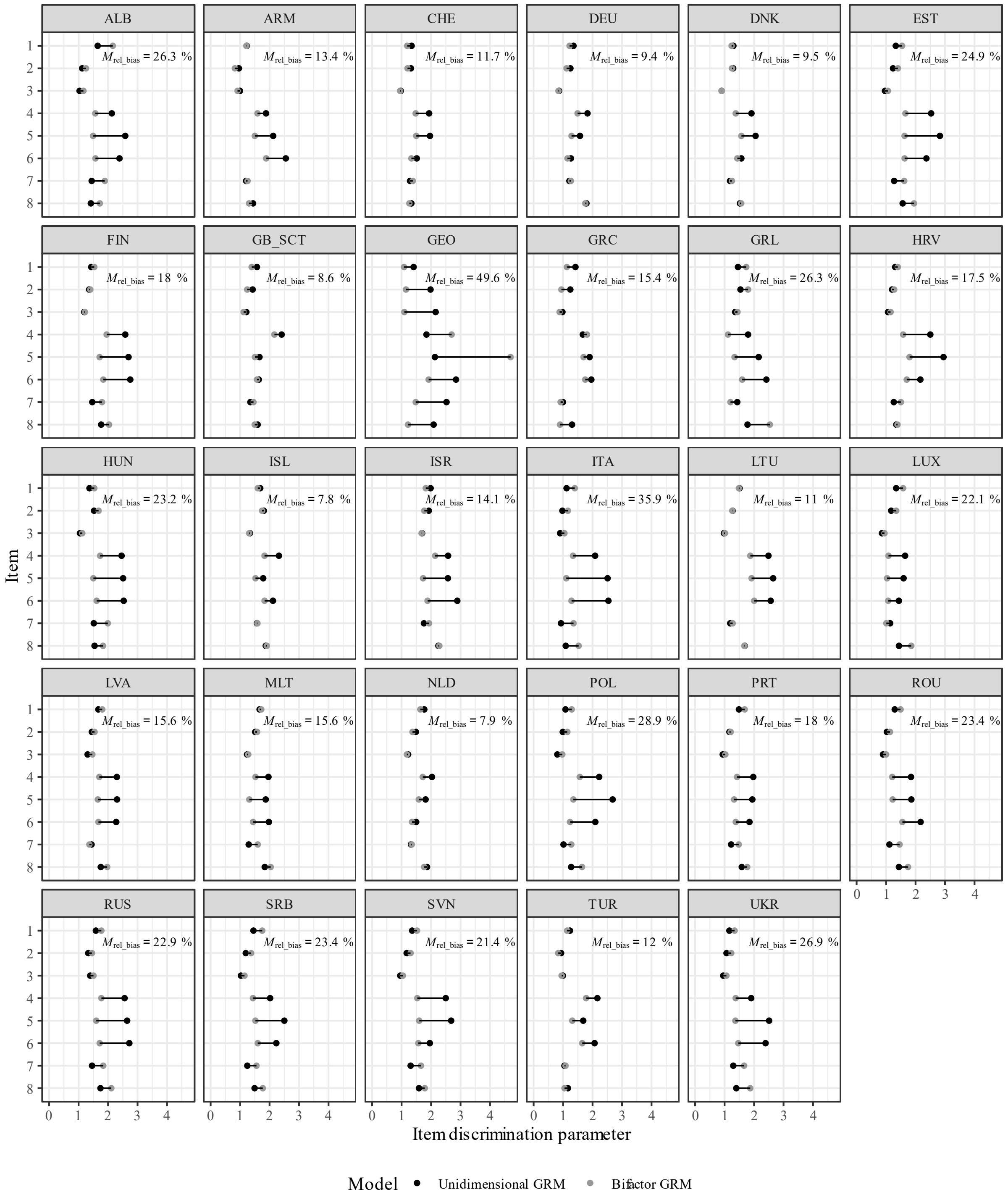

Notes. Values represent the item discrimination parameters of the unidimensional GRM and the marginal item discrimination parameters from the bifactor GRM. M_{rel_bias} represents the average relative bias across item parameters. Items (1) *headache*, (2) *stomachache*, (3) *backache*, (4) *feeling low*, (5) *irritability/bad temper*, (6) *feeling nervous*, (7) *difficulties in getting to sleep*, (8) *feeling dizzy*.

Figure A61. Comparing test characteristic curves between the unidimensional and the bifactor GRM.

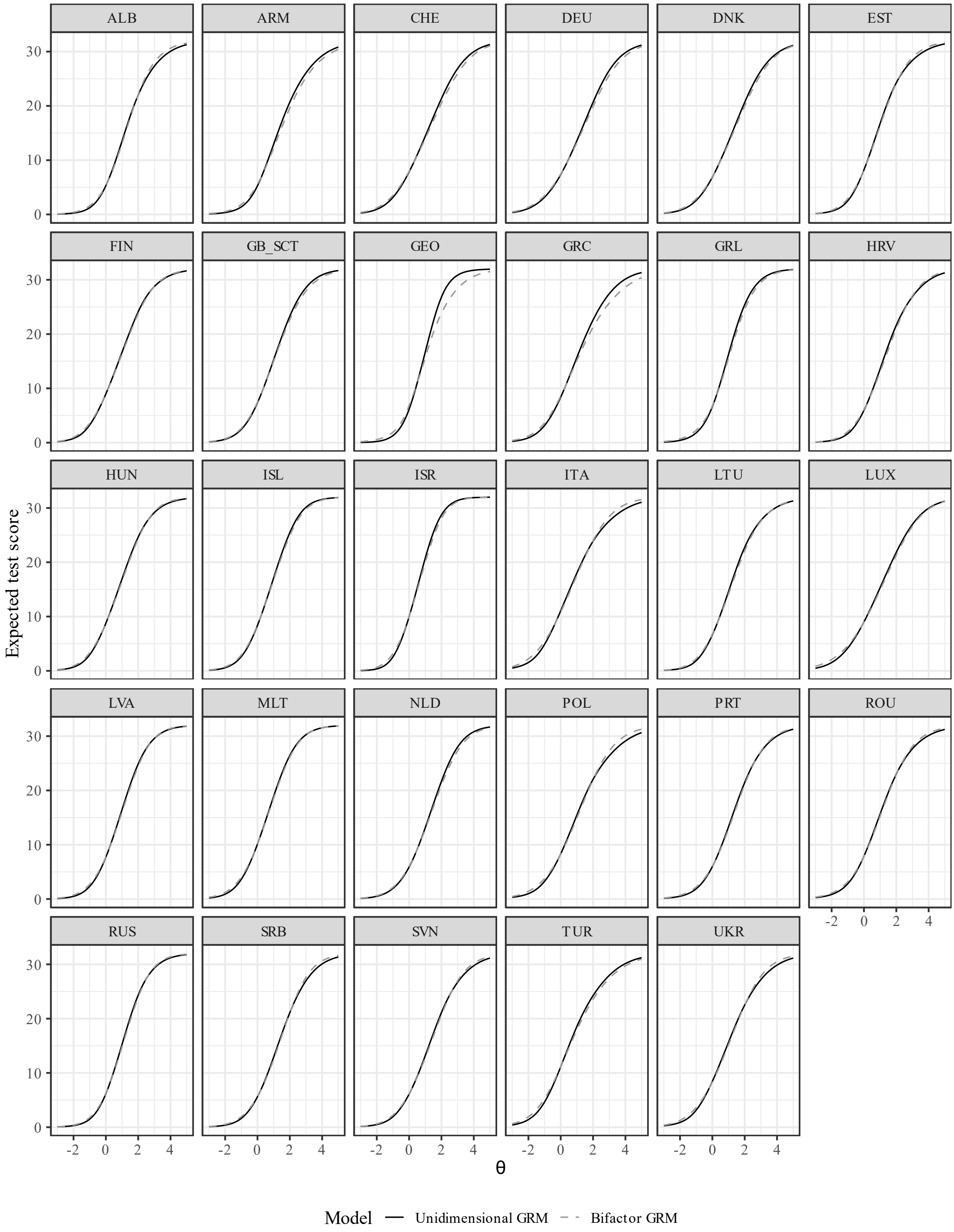

Figure A62. Comparing factor scores between the unidimensional and the bifactor GRM.

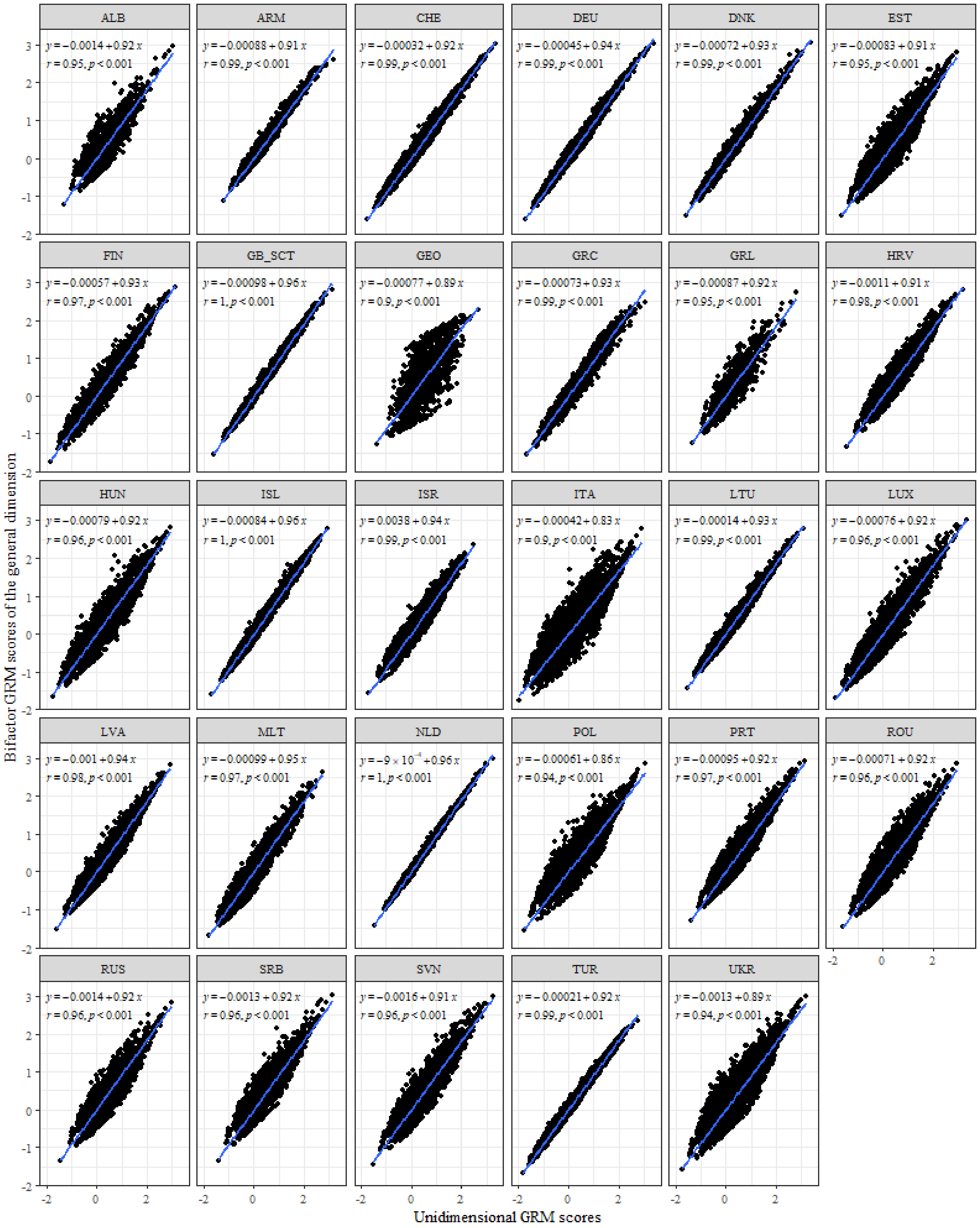

Notes. Values represent factor score estimates via expected a-posterior (EAP) method. The regression equations and correlation coefficients are shown.

Figure A63. Comparing test information functions between the unidimensional and the bifactor GRM.

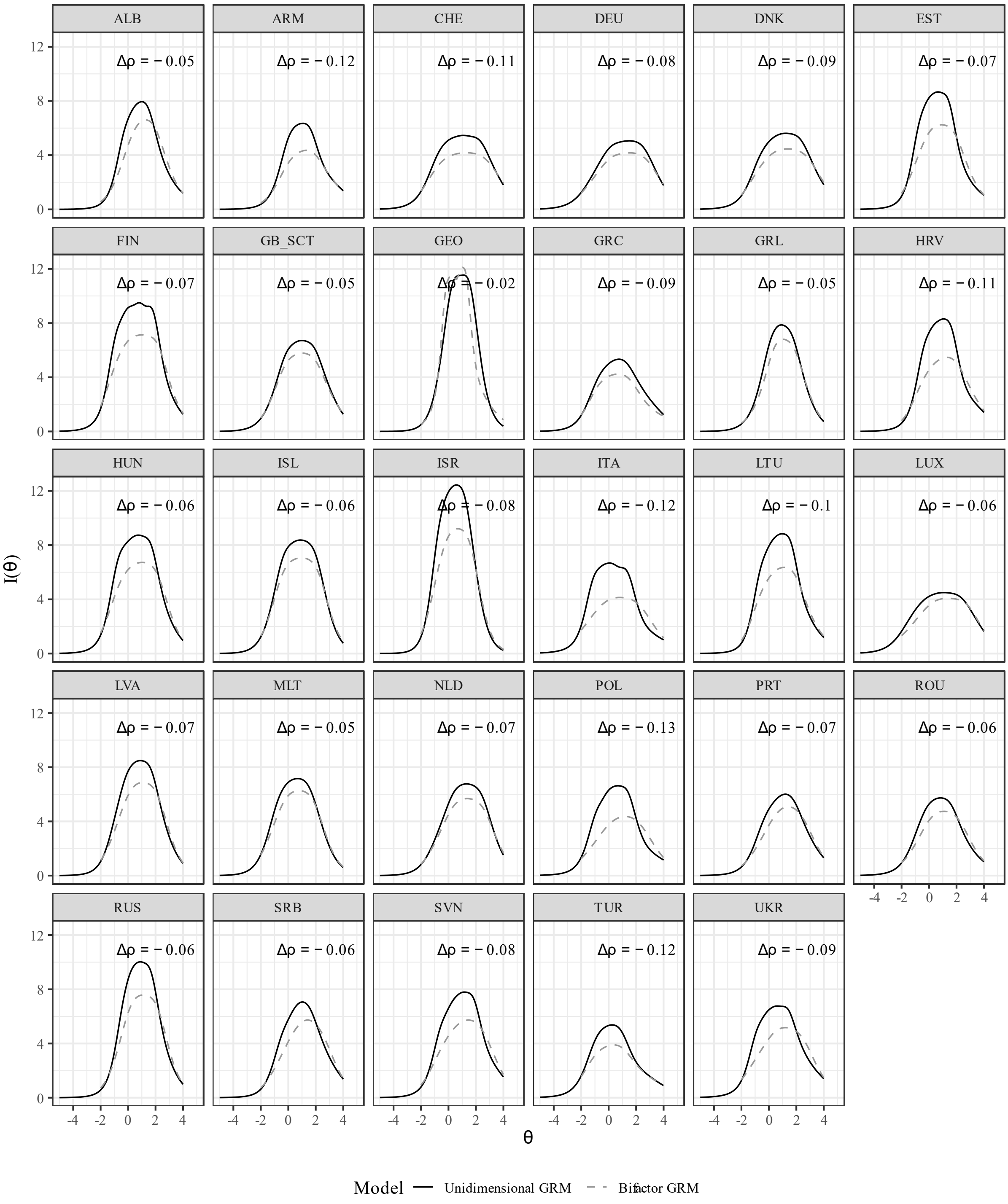

Notes. Δρ represents the change of the empirical marginal reliability between the unidimensional GRM and the bifactor GRM.

Figure A64. Approximate (non-)invariant parameters across countries.

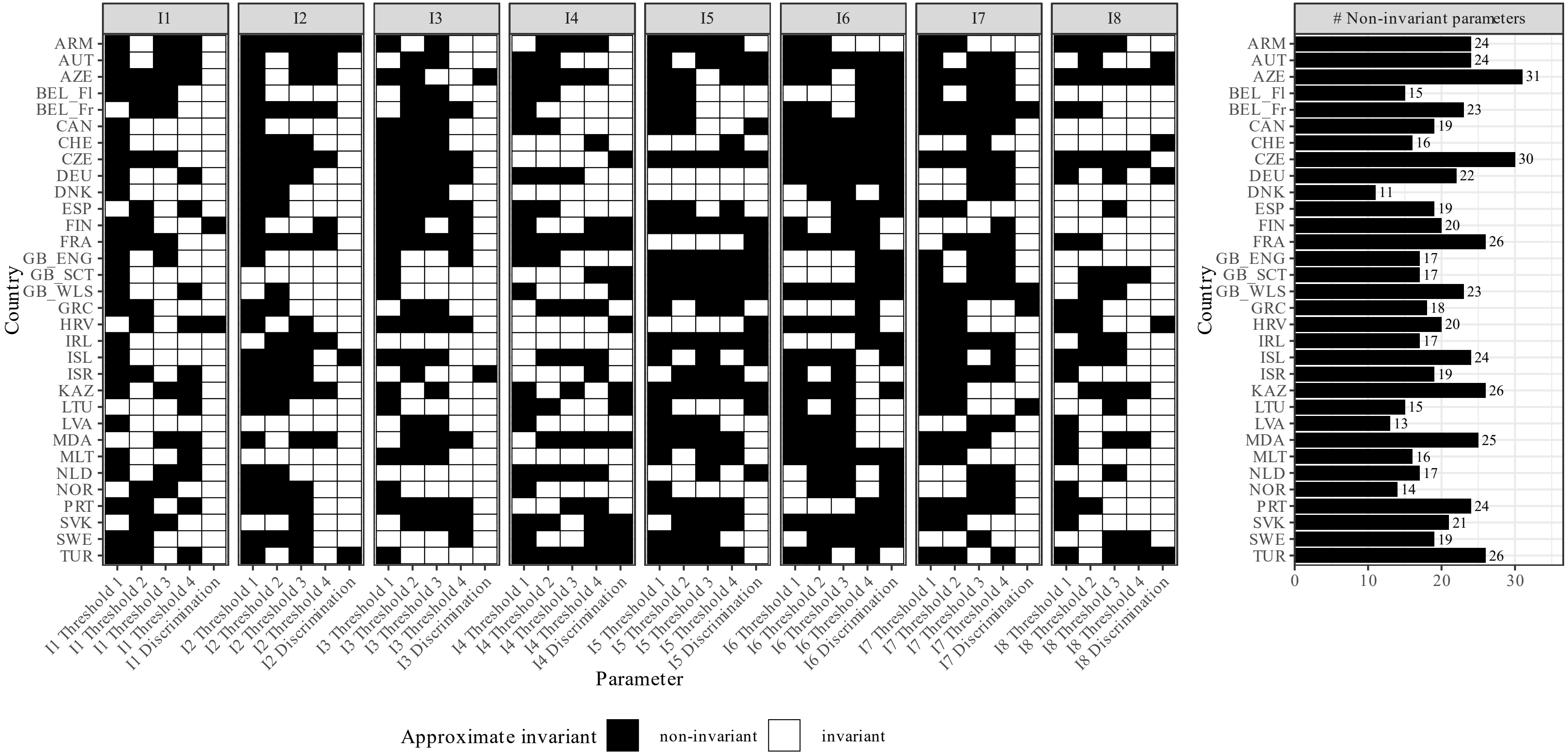

Notes. Results of the alignment analysis; *MLR* estimator; FIXED approach. Items (1) *headache*, (2) *stomachache*, (3) *backache*, (4) *feeling low*, (5) *irritability/bad temper*, (6) *feeling nervous*, (7) *difficulties in getting to sleep*, (8) *feeling dizzy*.

Figure A65. Differential test functioning: Reference group: ARM.

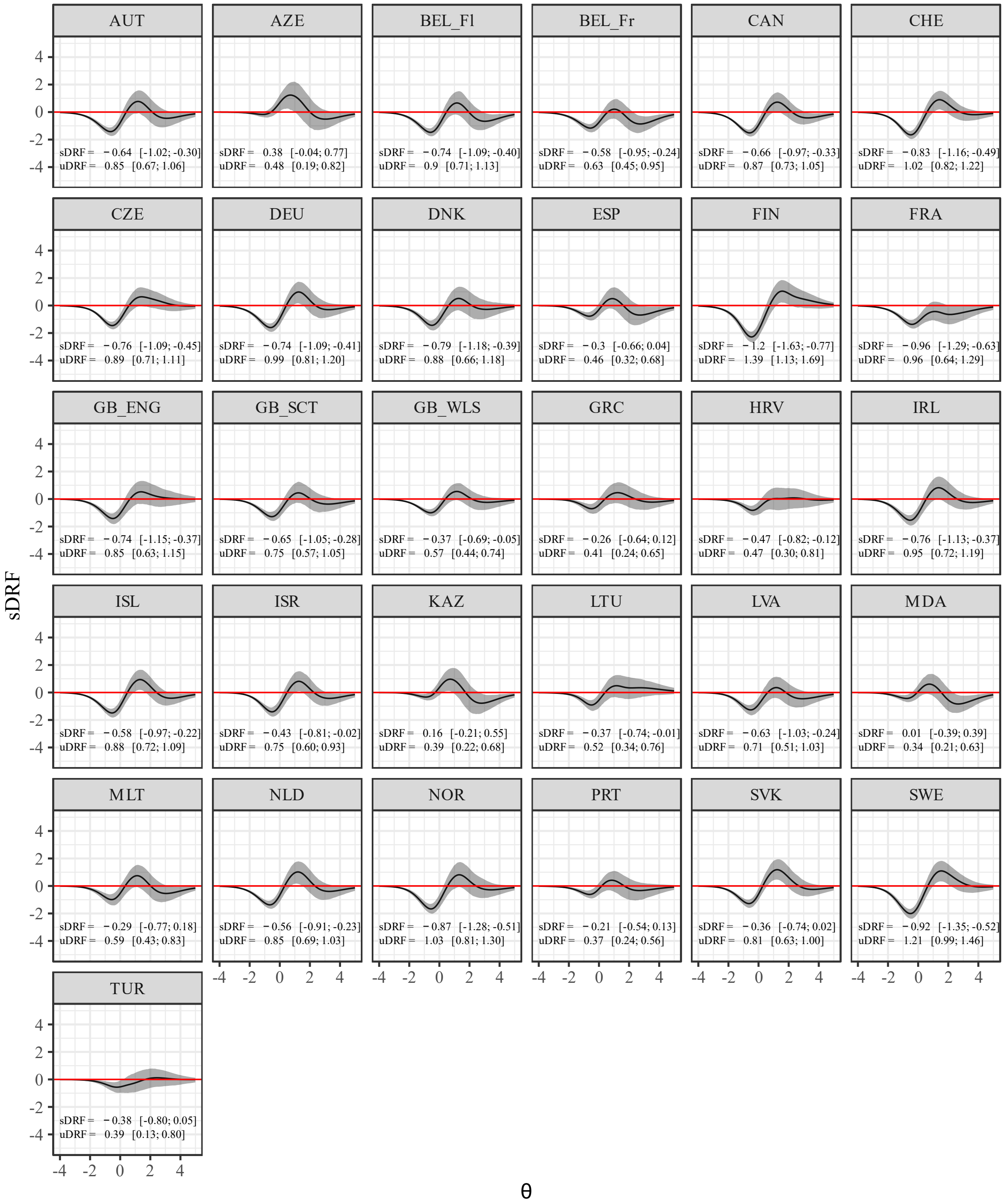

Notes. The curves show differences in expected test scores (with 99% CI) dependent on the level of the latent variable (ARM as reference group), *sDRF* = compensatory differential response functioning statistic with 99% CI, *uDRF* = non-compensatory differential response functioning statistic with 99% CI.

Figure A66. Differential test functioning: Reference group: AUT.

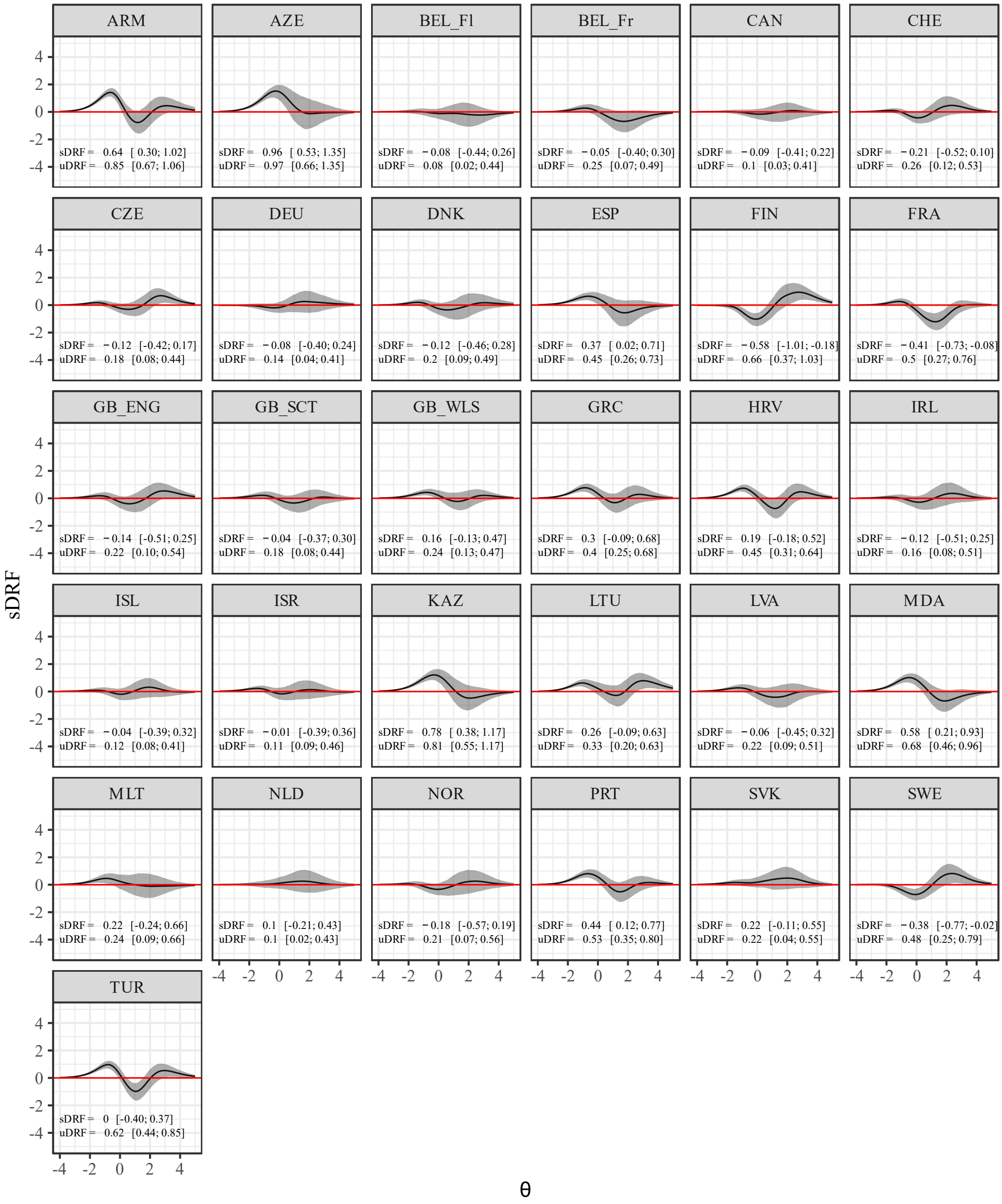

Notes. The curves show differences in expected test scores (with 99% CI) dependent on the level of the latent variable (AUT as reference group), *sDRF* = compensatory differential response functioning statistic with 99% CI, *uDRF* = non-compensatory differential response functioning statistic with 99% CI.

Figure A67. Differential test functioning: Reference group: AZE.

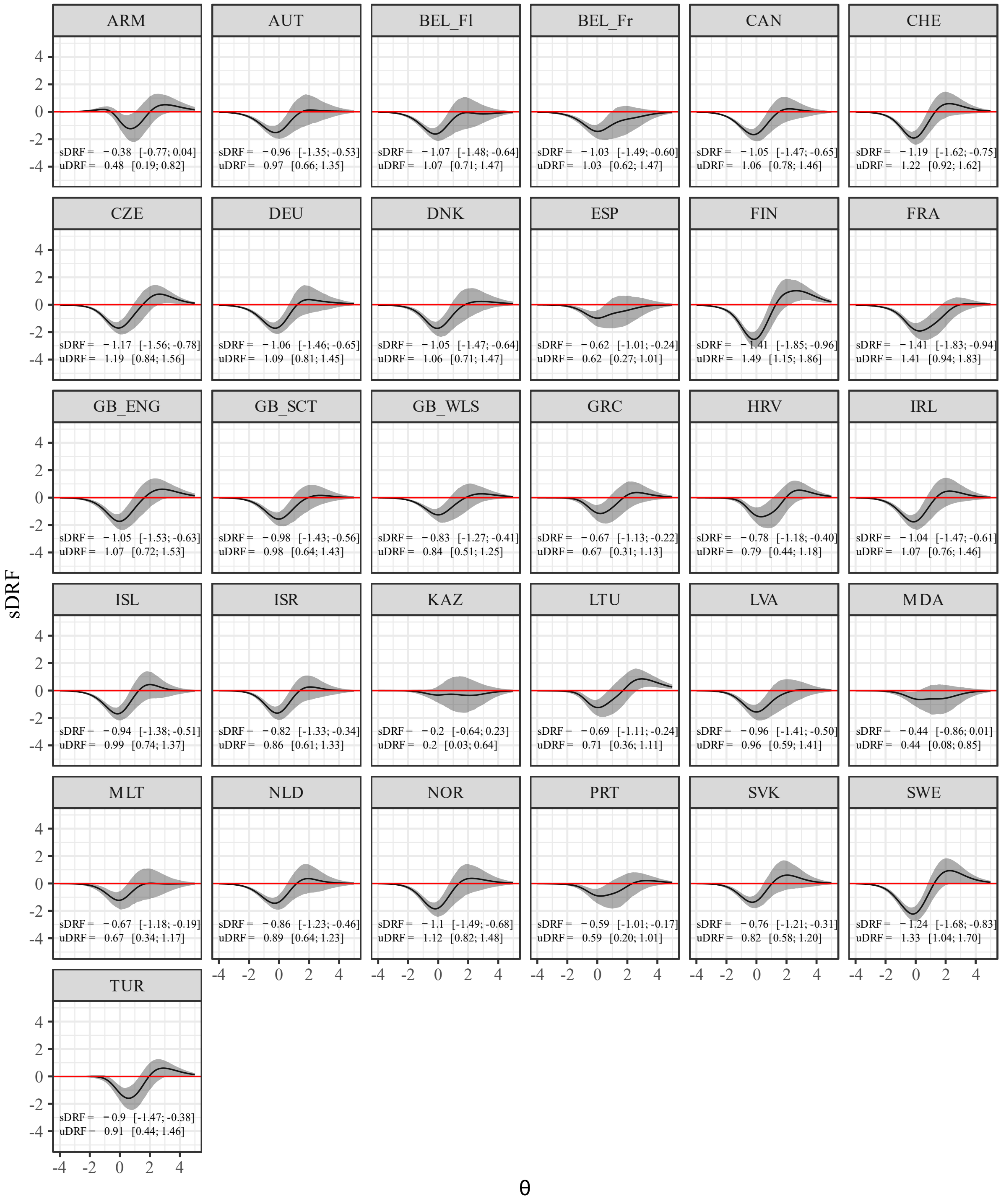

Notes. The curves show differences in expected test scores (with 99% CI) dependent on the level of the latent variable (AZE as reference group), *sDRF* = compensatory differential response functioning statistic with 99% CI, *uDRF* = non-compensatory differential response functioning statistic with 99% CI.

Figure A68. Differential test functioning: Reference group: BEL_FL.

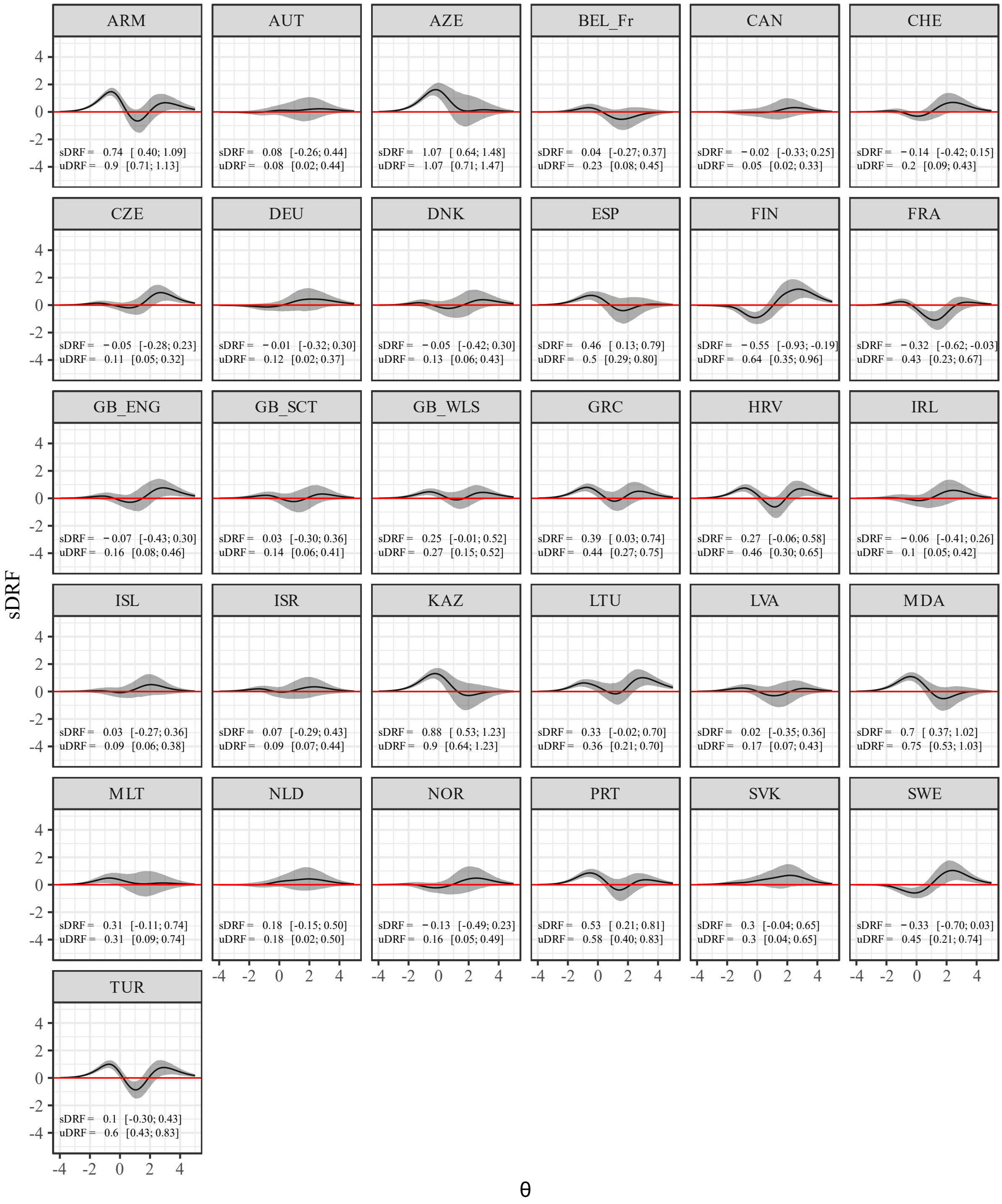

Notes. The curves show differences in expected test scores (with 99% CI) dependent on the level of the latent variable (BEL_FL as reference group), *sDRF* = compensatory differential response functioning statistic with 99% CI, *uDRF* = non-compensatory differential response functioning statistic with 99% CI.

Figure A69. Differential test functioning: Reference group: BEL_FR.

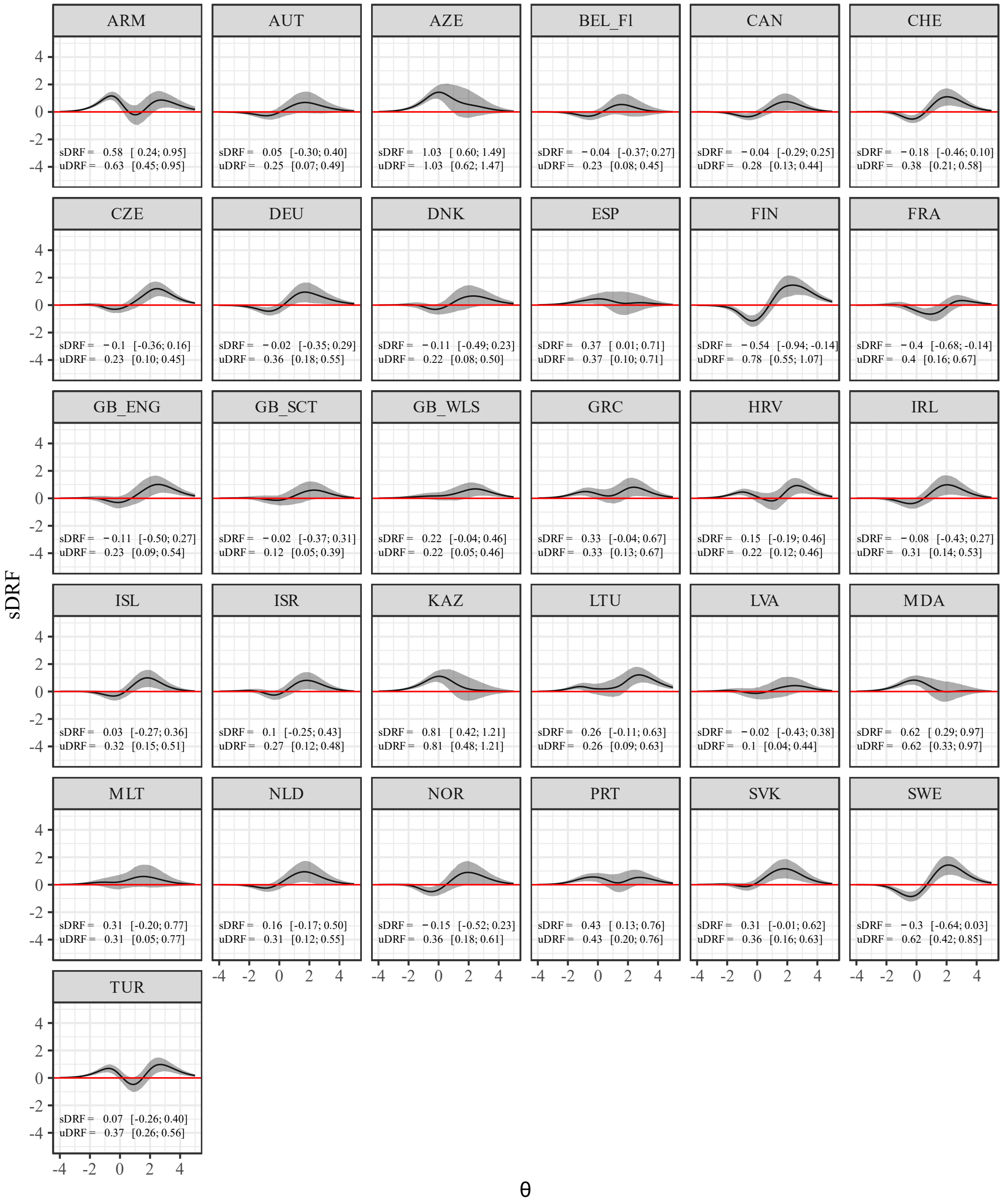

Notes. The curves show differences in expected test scores (with 99% CI) dependent on the level of the latent variable (BEL_FR as reference group), *sDRF* = compensatory differential response functioning statistic with 99% CI, *uDRF* = non-compensatory differential response functioning statistic with 99% CI.

Figure A70. Differential test functioning: Reference group: CAN.

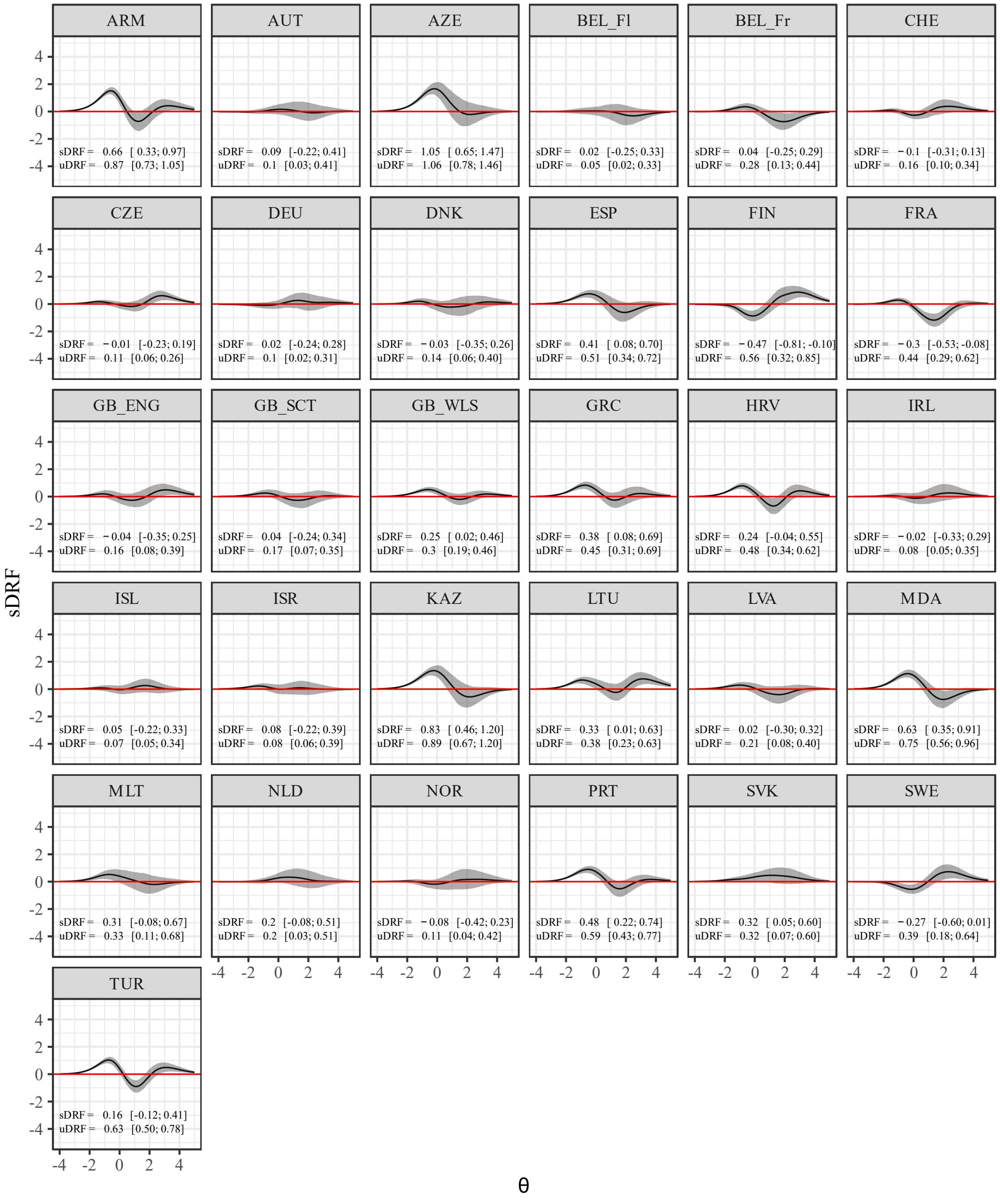

Notes. The curves show differences in expected test scores (with 99% CI) dependent on the level of the latent variable (CAN as reference group), *sDRF* = compensatory differential response functioning statistic with 99% CI, *uDRF* = non-compensatory differential response functioning statistic with 99% CI.

Figure A71. Differential test functioning: Reference group: CHE.

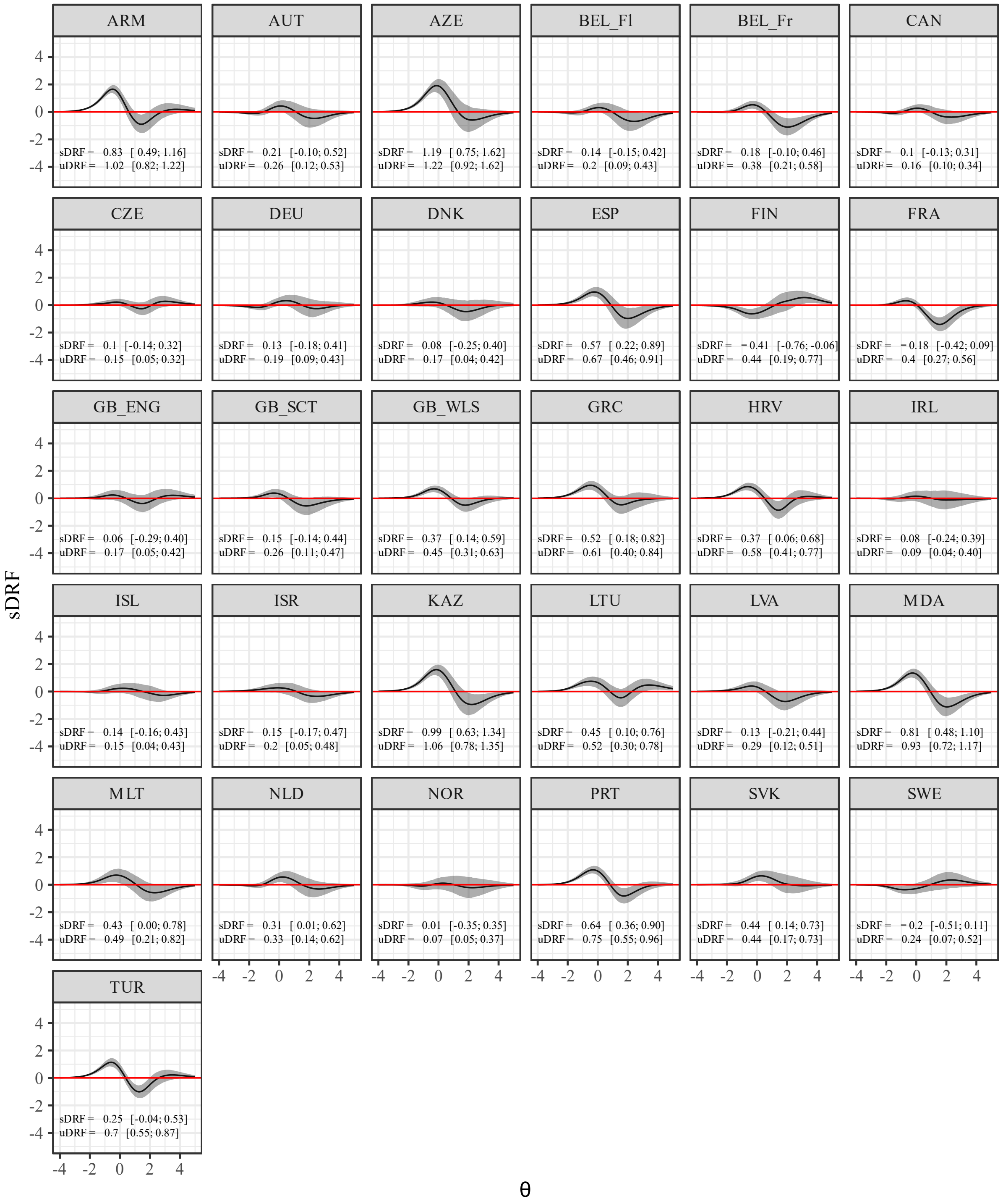

Notes. The curves show differences in expected test scores (with 99% CI) dependent on the level of the latent variable (CHE as reference group), *sDRF* = compensatory differential response functioning statistic with 99% CI, *uDRF* = non-compensatory differential response functioning statistic with 99% CI.

Figure A72. Differential test functioning: Reference group: CZE.

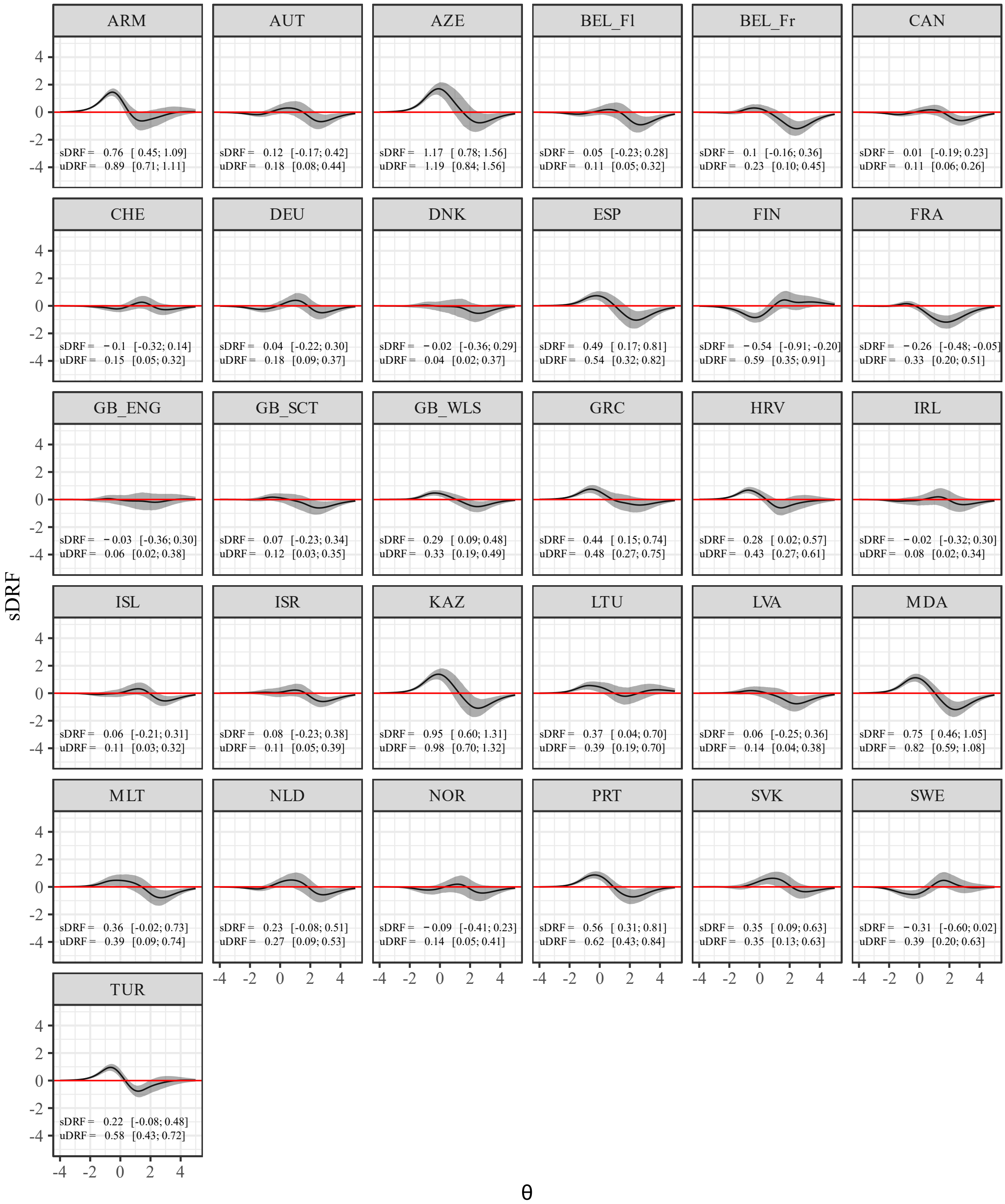

Notes. The curves show differences in expected test scores (with 99% CI) dependent on the level of the latent variable (CZE as reference group), *sDRF* = compensatory differential response functioning statistic with 99% CI, *uDRF* = non-compensatory differential response functioning statistic with 99% CI.

Figure A73. Differential test functioning: Reference group: DEU.

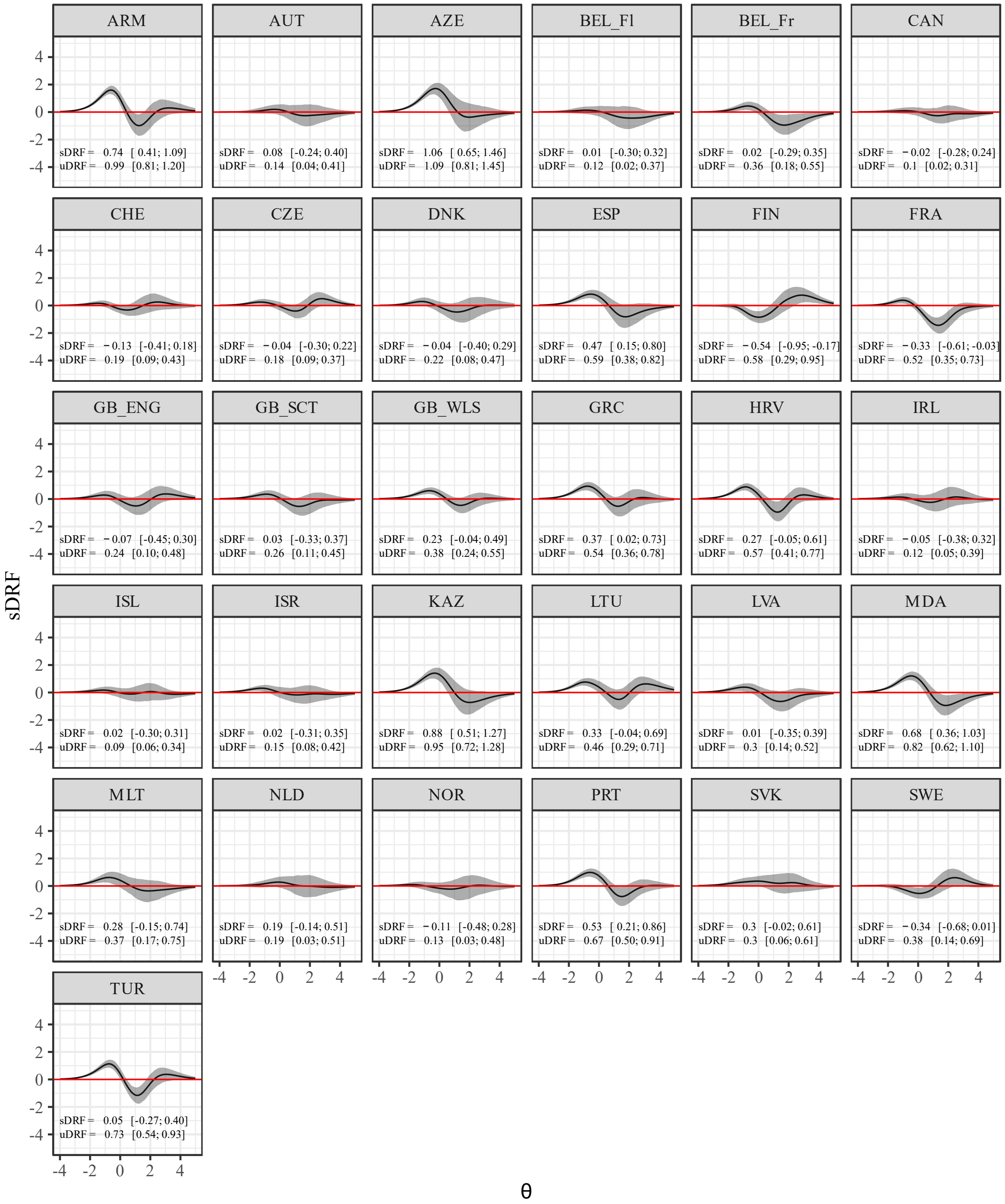

Notes. The curves show differences in expected test scores (with 99% CI) dependent on the level of the latent variable (DEU as reference group), *sDRF* = compensatory differential response functioning statistic with 99% CI, *uDRF* = non-compensatory differential response functioning statistic with 99% CI.

Figure A74. Differential test functioning: Reference group: DNK.

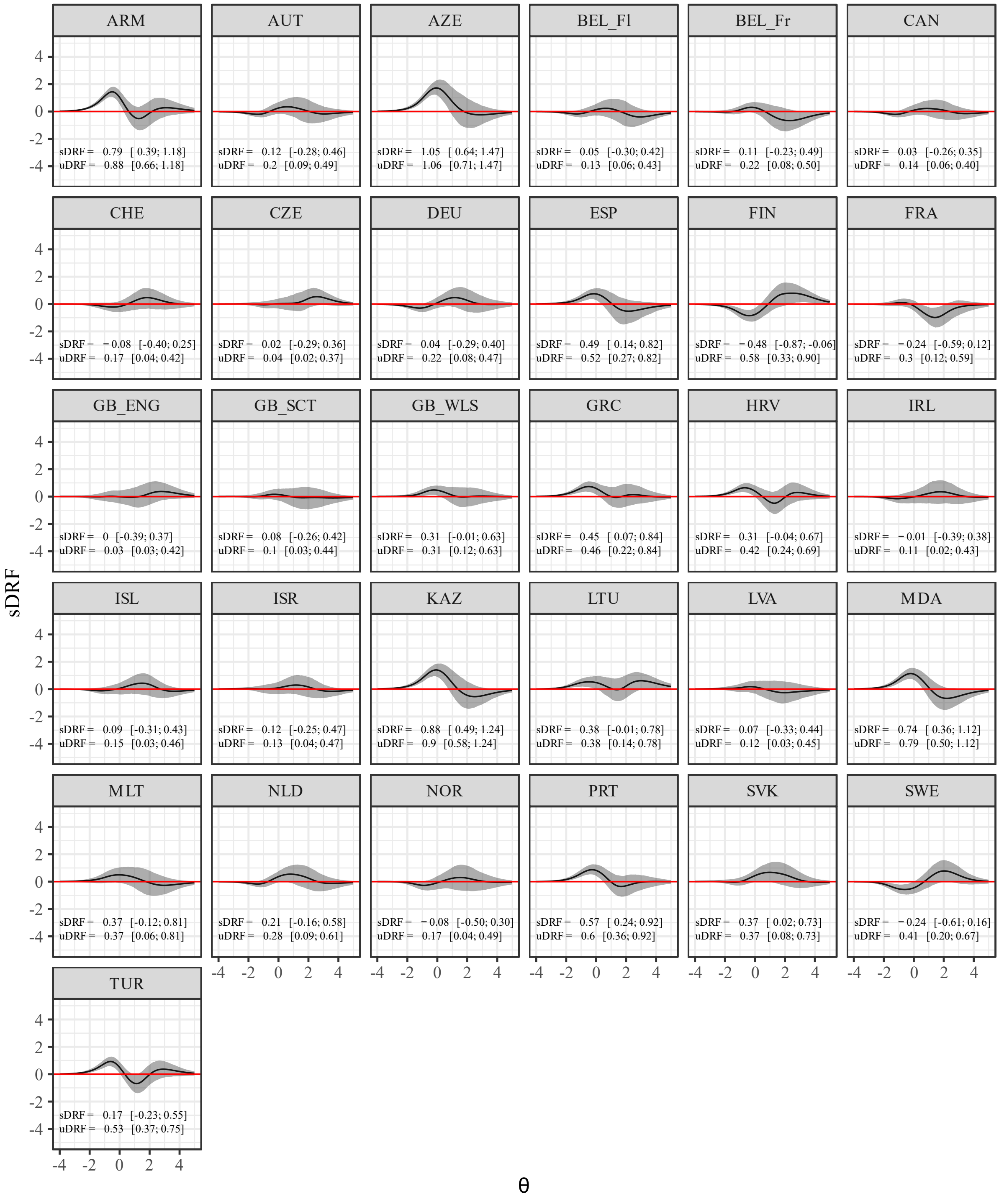

Notes. The curves show differences in expected test scores (with 99% CI) dependent on the level of the latent variable (DNK as reference group), *sDRF* = compensatory differential response functioning statistic with 99% CI, *uDRF* = non-compensatory differential response functioning statistic with 99% CI.

Figure A75. Differential test functioning: Reference group: ESP.

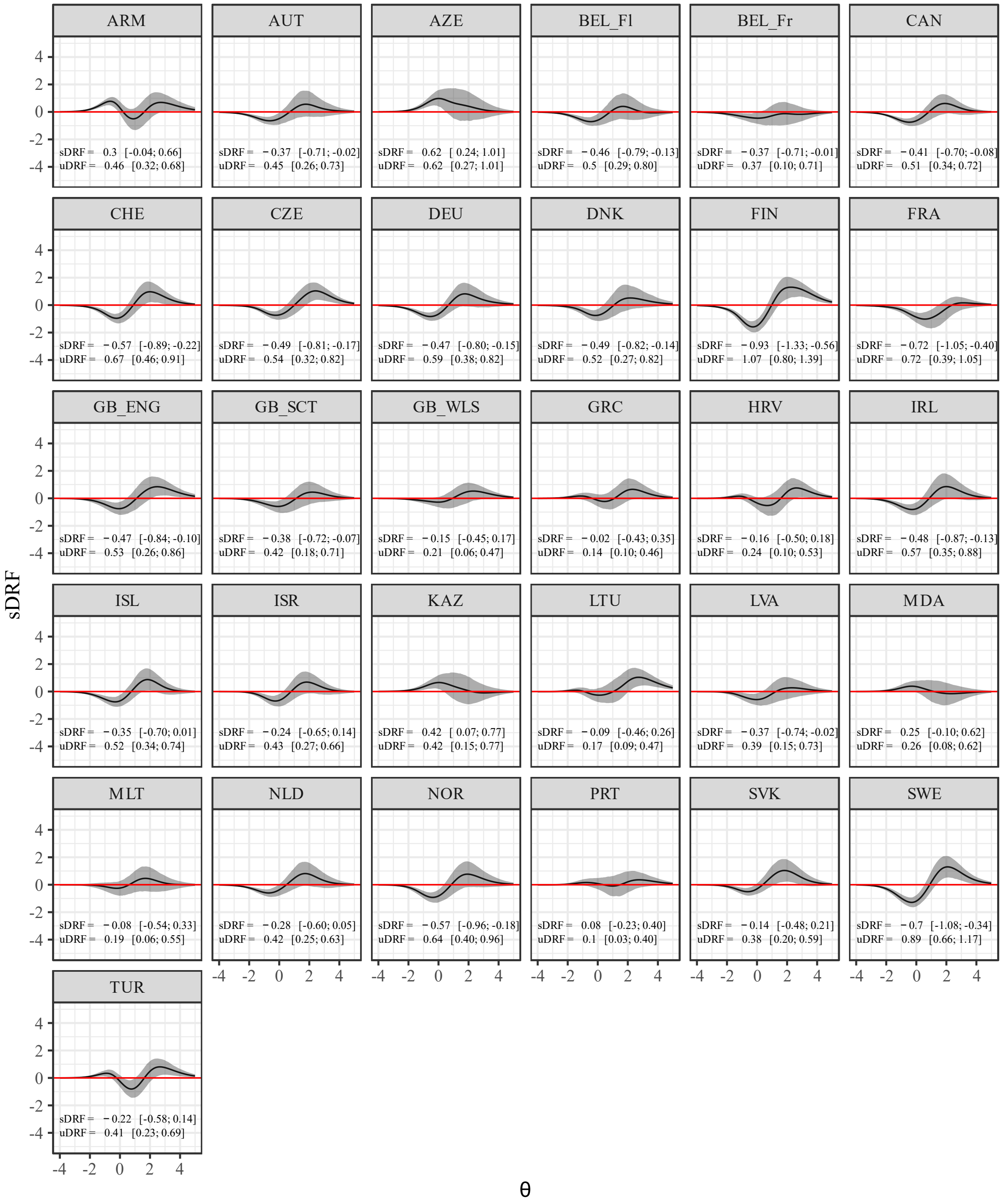

Notes. The curves show differences in expected test scores (with 99% CI) dependent on the level of the latent variable (ESP as reference group), *sDRF* = compensatory differential response functioning statistic with 99% CI, *uDRF* = non-compensatory differential response functioning statistic with 99% CI.

Figure A76. Differential test functioning: Reference group: FIN.

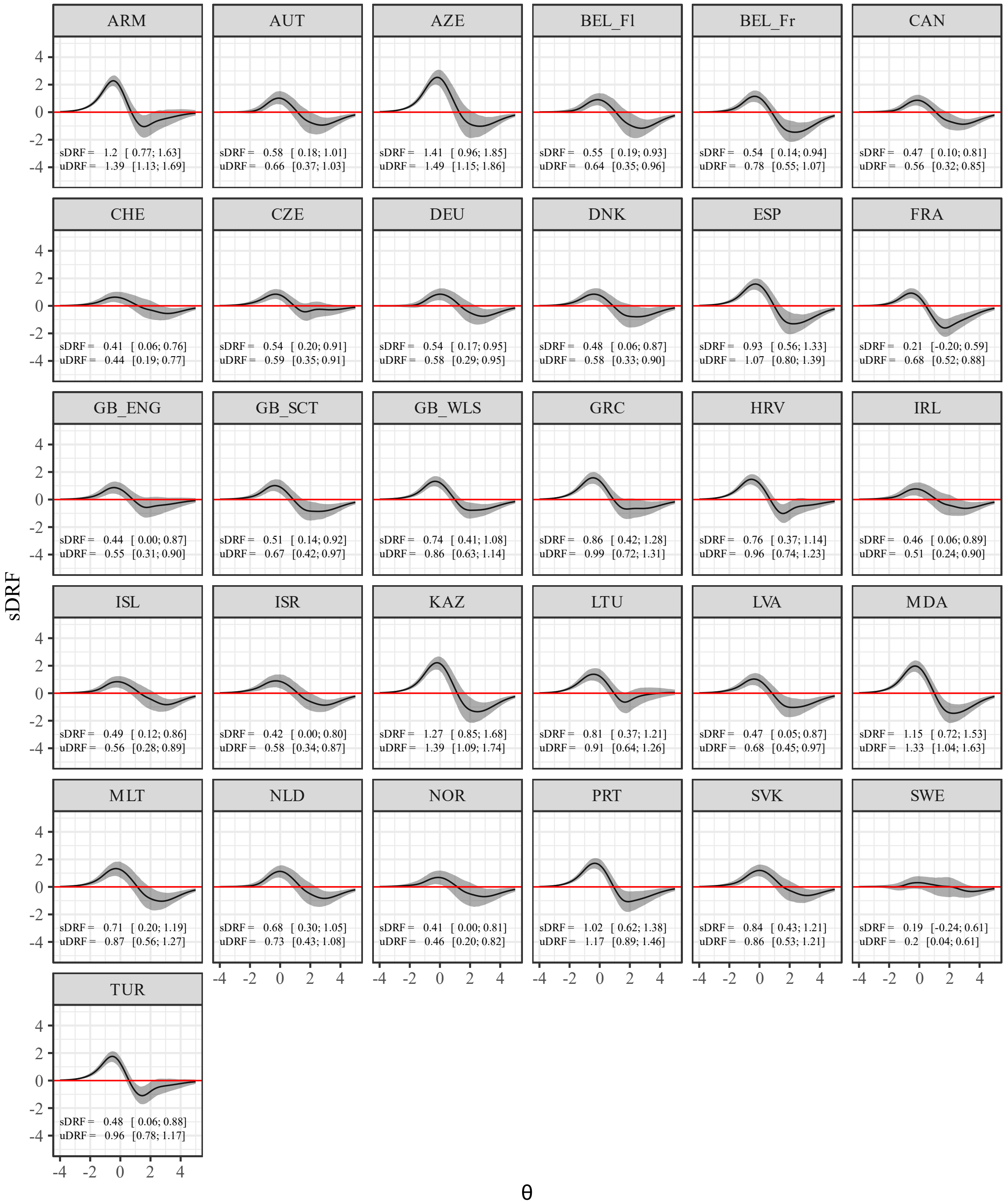

Notes. The curves show differences in expected test scores (with 99% CI) dependent on the level of the latent variable (FIN as reference group), *sDRF* = compensatory differential response functioning statistic with 99% CI, *uDRF* = non-compensatory differential response functioning statistic with 99% CI.

Figure A77. Differential test functioning: Reference group: FRA.

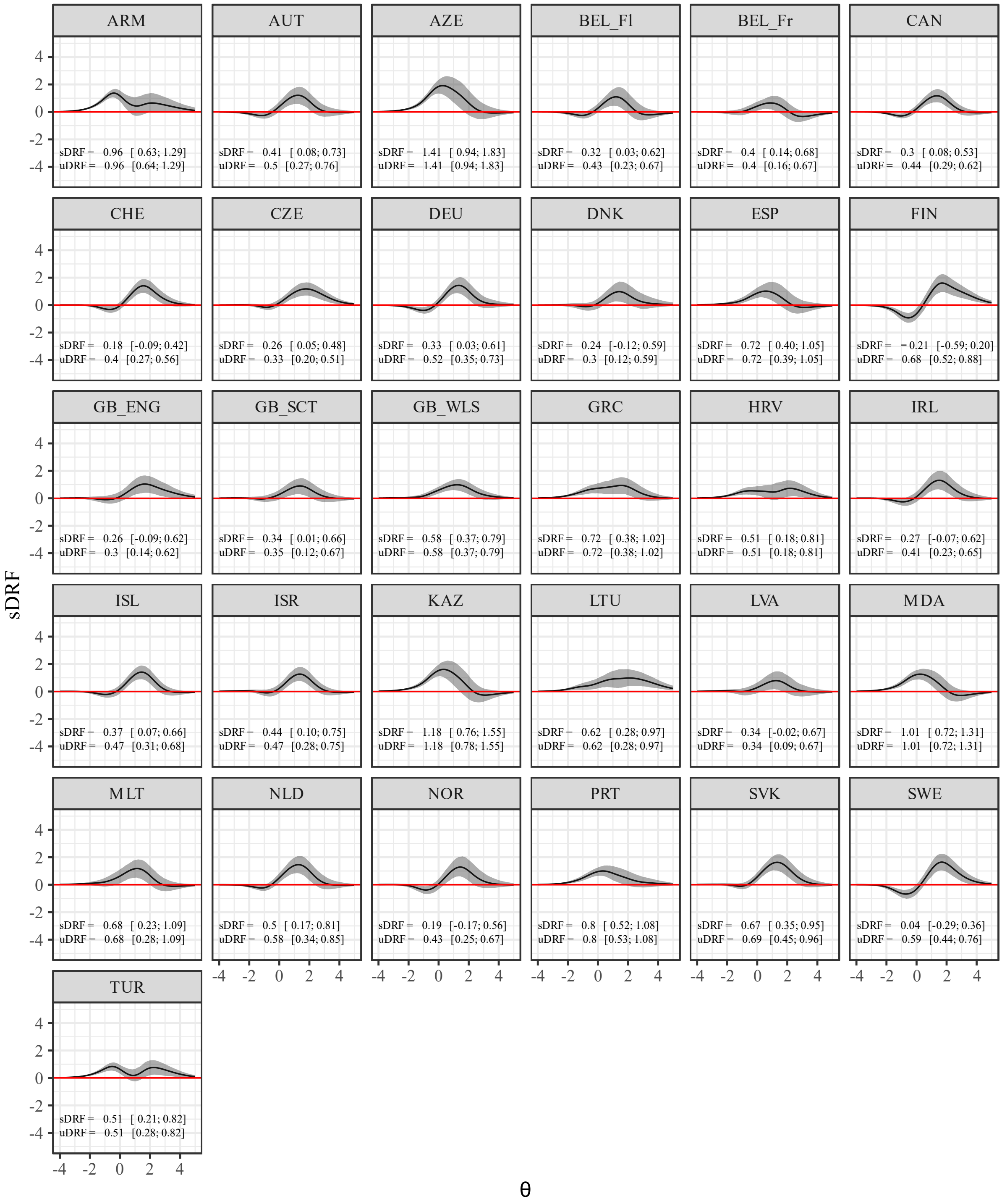

Notes. The curves show differences in expected test scores (with 99% CI) dependent on the level of the latent variable (FRA as reference group), *sDRF* = compensatory differential response functioning statistic with 99% CI, *uDRF* = non-compensatory differential response functioning statistic with 99% CI.

Figure A78. Differential test functioning: Reference group: GB_SCT.

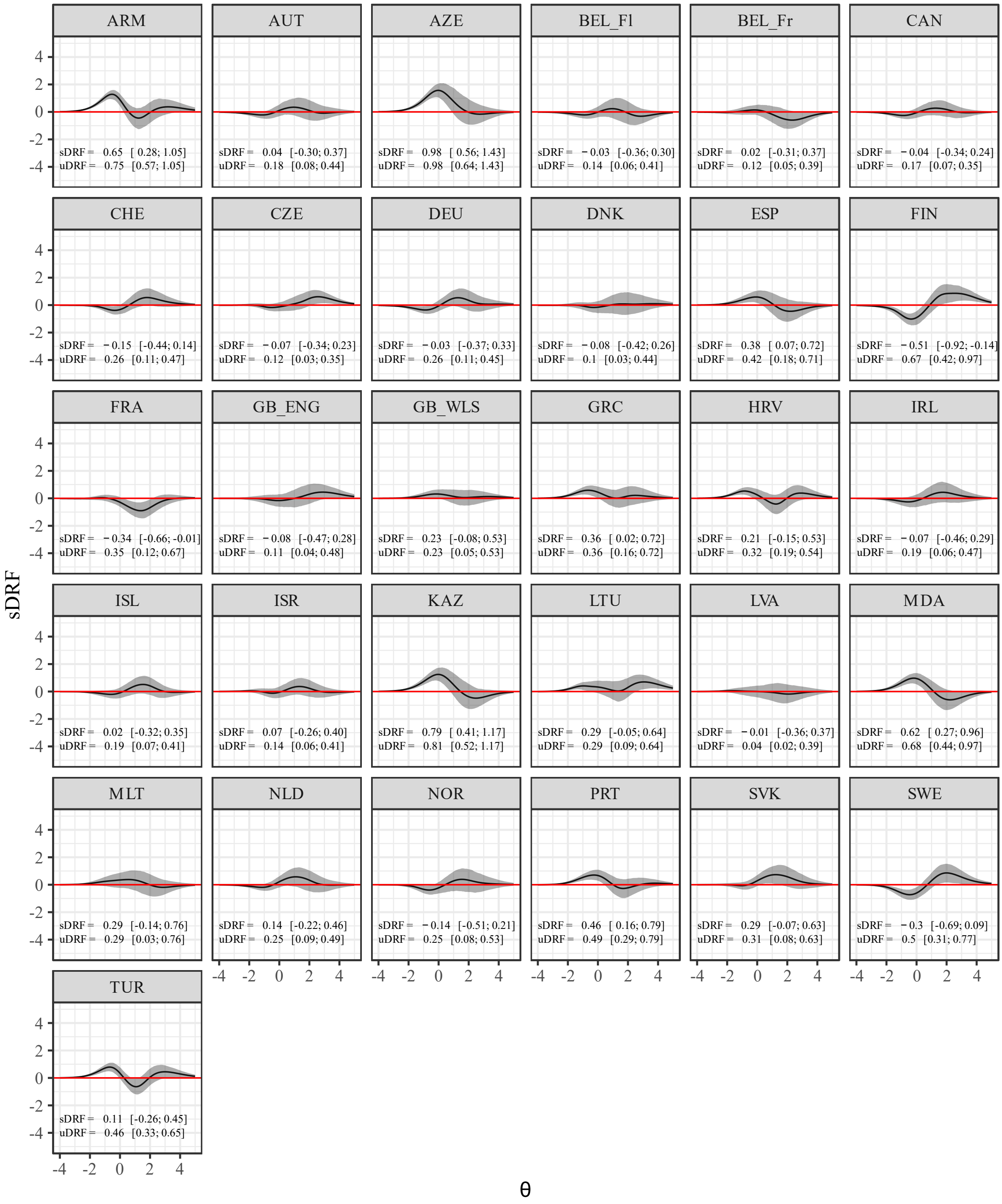

Notes. The curves show differences in expected test scores (with 99% CI) dependent on the level of the latent variable (GB_SCT as reference group), *sDRF* = compensatory differential response functioning statistic with 99% CI, *uDRF* = non-compensatory differential response functioning statistic with 99% CI.

Figure A79. Differential test functioning: Reference group: GB_WLS.

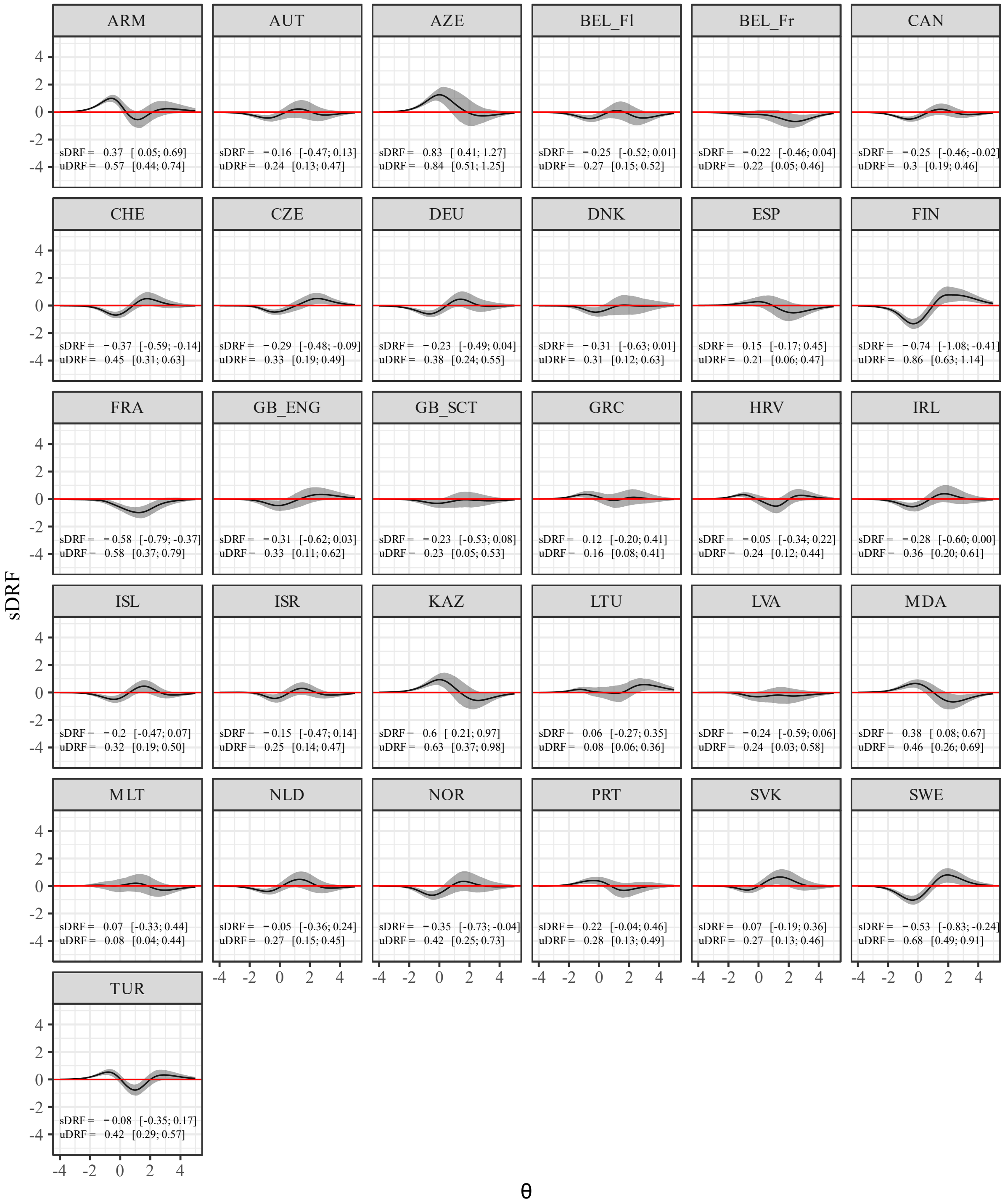

Notes. The curves show differences in expected test scores (with 99% CI) dependent on the level of the latent variable (GB_WLS as reference group), *sDRF* = compensatory differential response functioning statistic with 99% CI, *uDRF* = non-compensatory differential response functioning statistic with 99% CI.

Figure A80. Differential test functioning: Reference group: GRC.

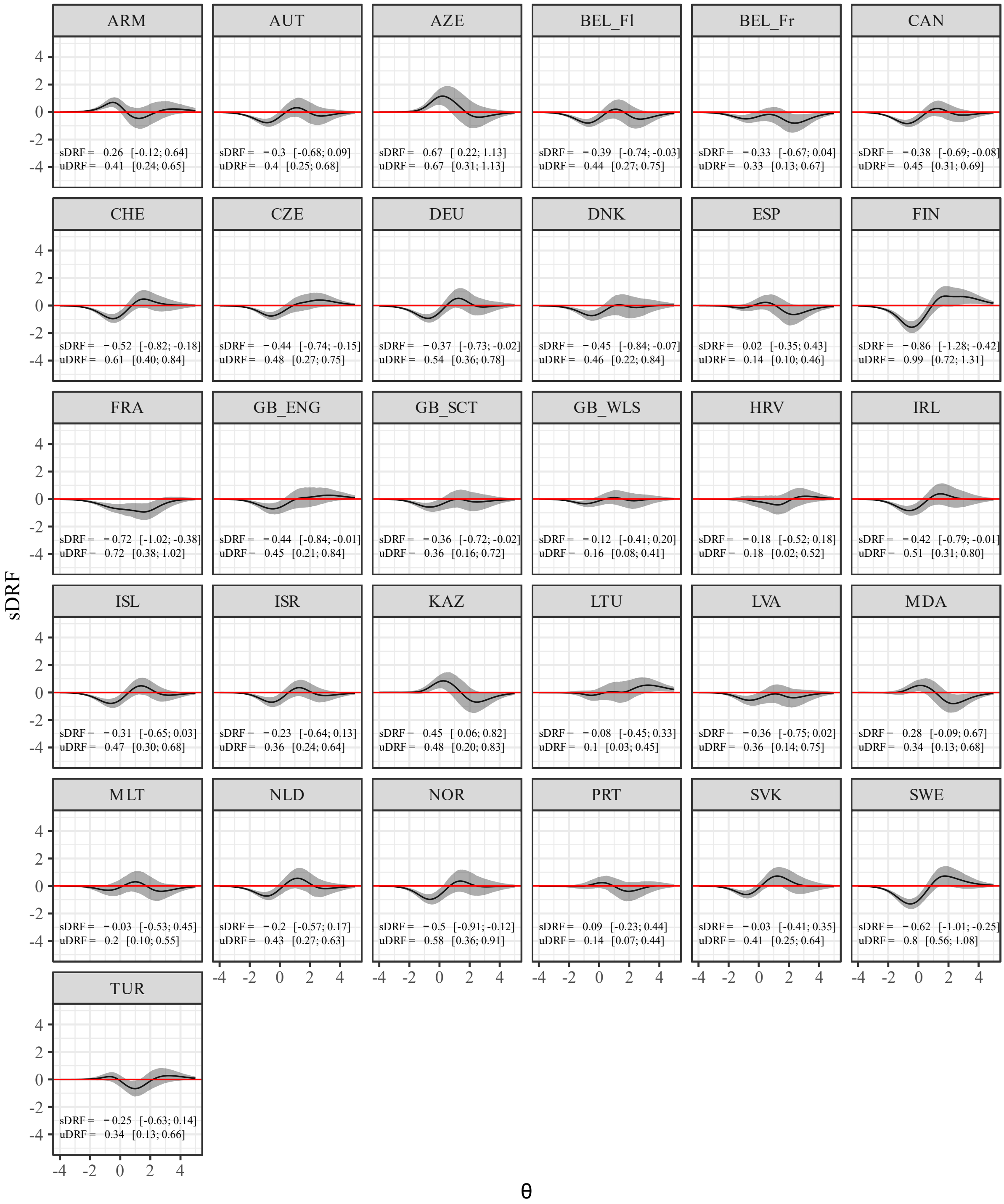

Notes. The curves show differences in expected test scores (with 99% CI) dependent on the level of the latent variable (GRC as reference group), *sDRF* = compensatory differential response functioning statistic with 99% CI, *uDRF* = non-compensatory differential response functioning statistic with 99% CI.

Figure A81. Differential test functioning: Reference group: HRV.

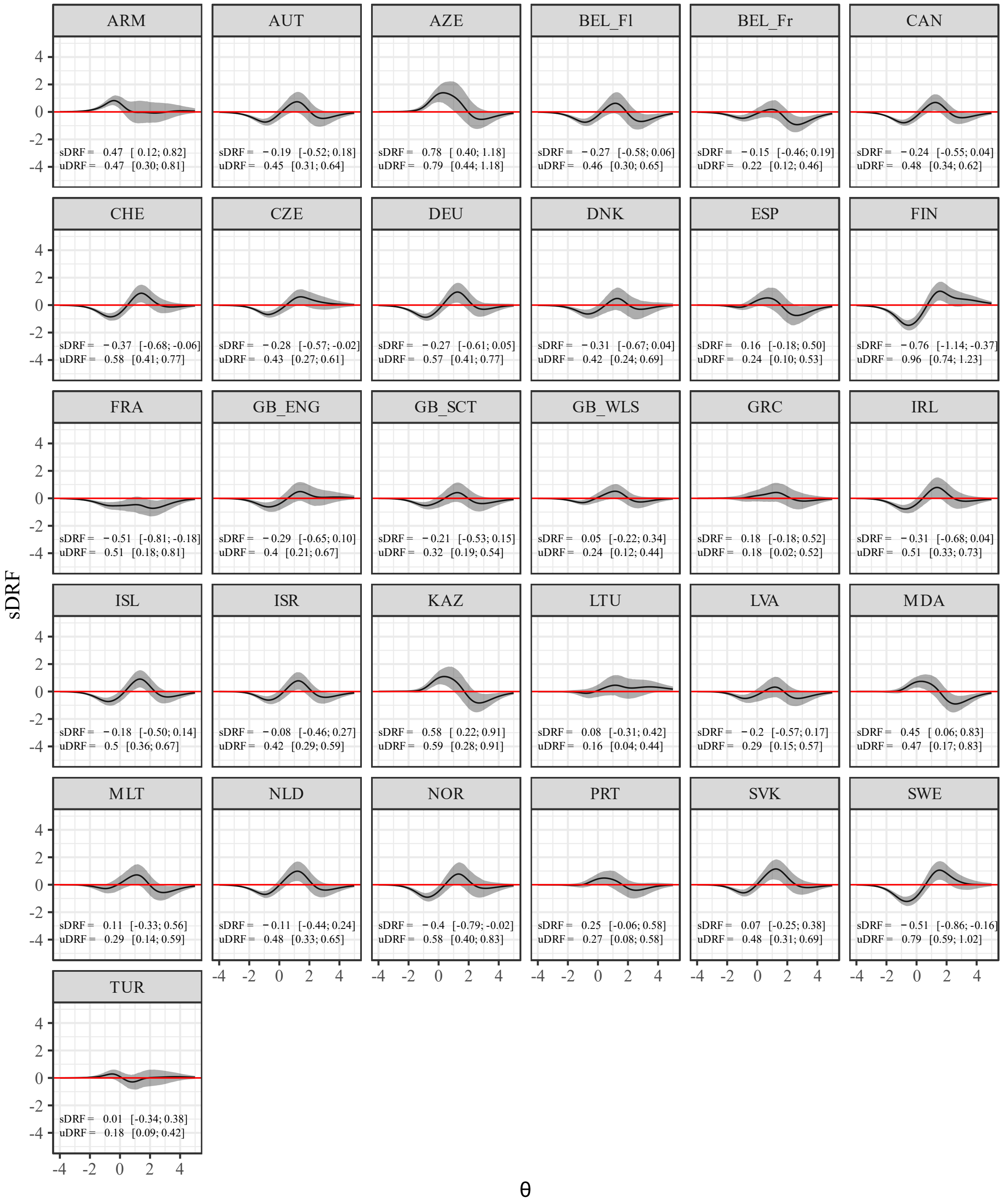

Notes. The curves show differences in expected test scores (with 99% CI) dependent on the level of the latent variable (HRV as reference group), *sDRF* = compensatory differential response functioning statistic with 99% CI, *uDRF* = non-compensatory differential response functioning statistic with 99% CI.

Figure A82. Differential test functioning: Reference group: IRL.

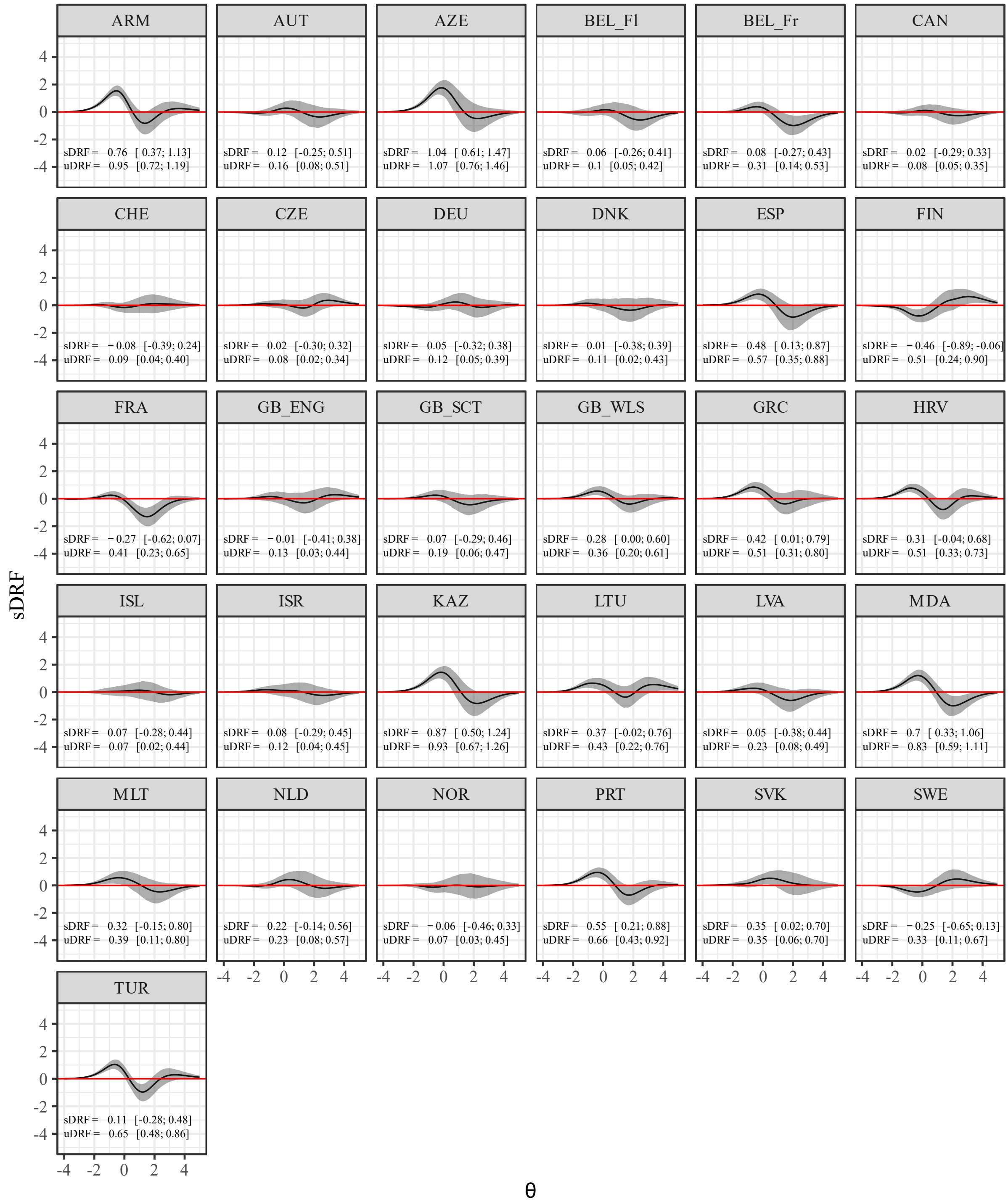

Notes. The curves show differences in expected test scores (with 99% CI) dependent on the level of the latent variable (IRL as reference group), *sDRF* = compensatory differential response functioning statistic with 99% CI, *uDRF* = non-compensatory differential response functioning statistic with 99% CI.

Figure A83. Differential test functioning: Reference group: ISL.

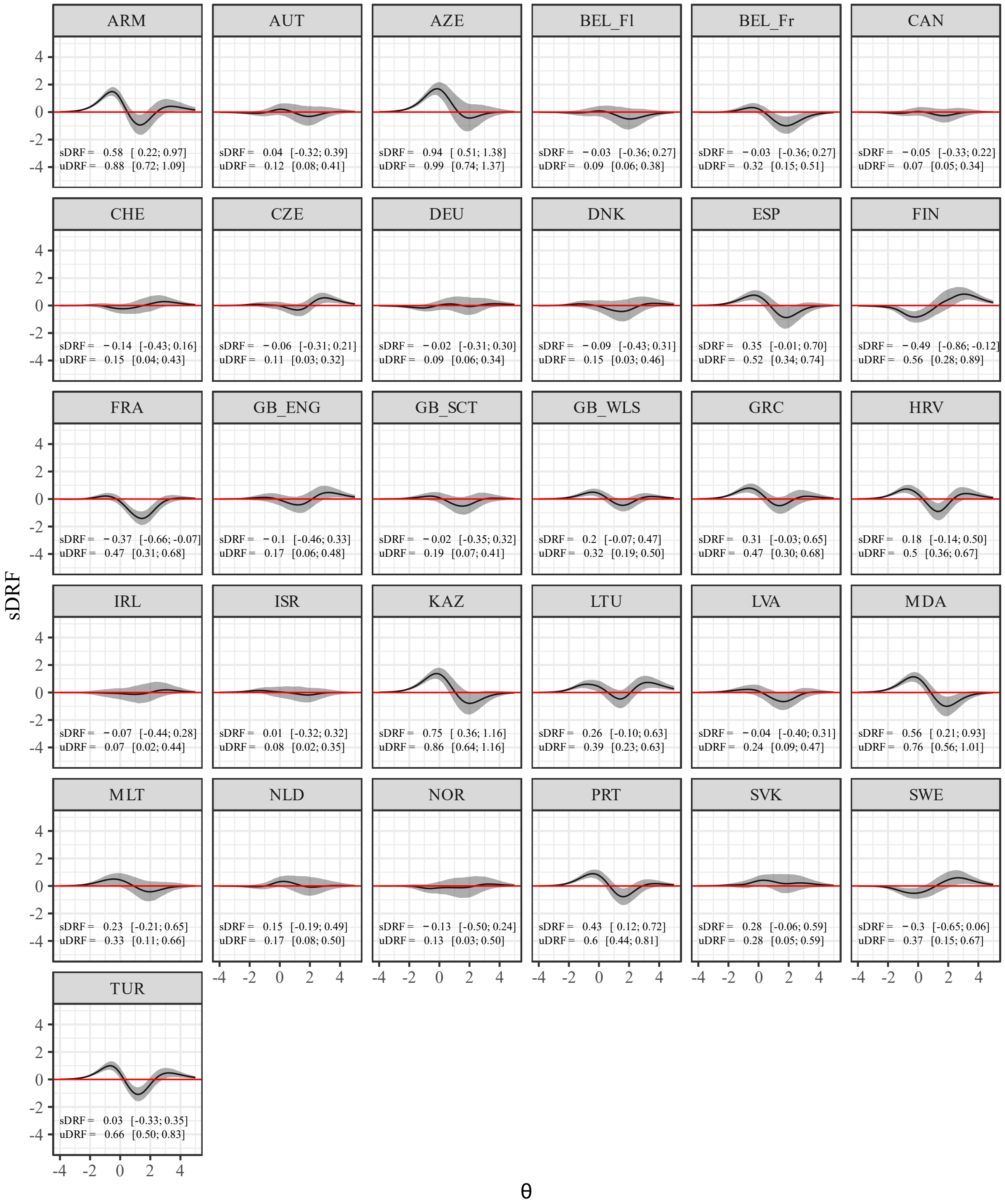

Notes. The curves show differences in expected test scores (with 99% CI) dependent on the level of the latent variable (ISL as reference group), *sDRF* = compensatory differential response functioning statistic with 99% CI, *uDRF* = non-compensatory differential response functioning statistic with 99% CI.

Figure A84. Differential test functioning: Reference group: ISR.

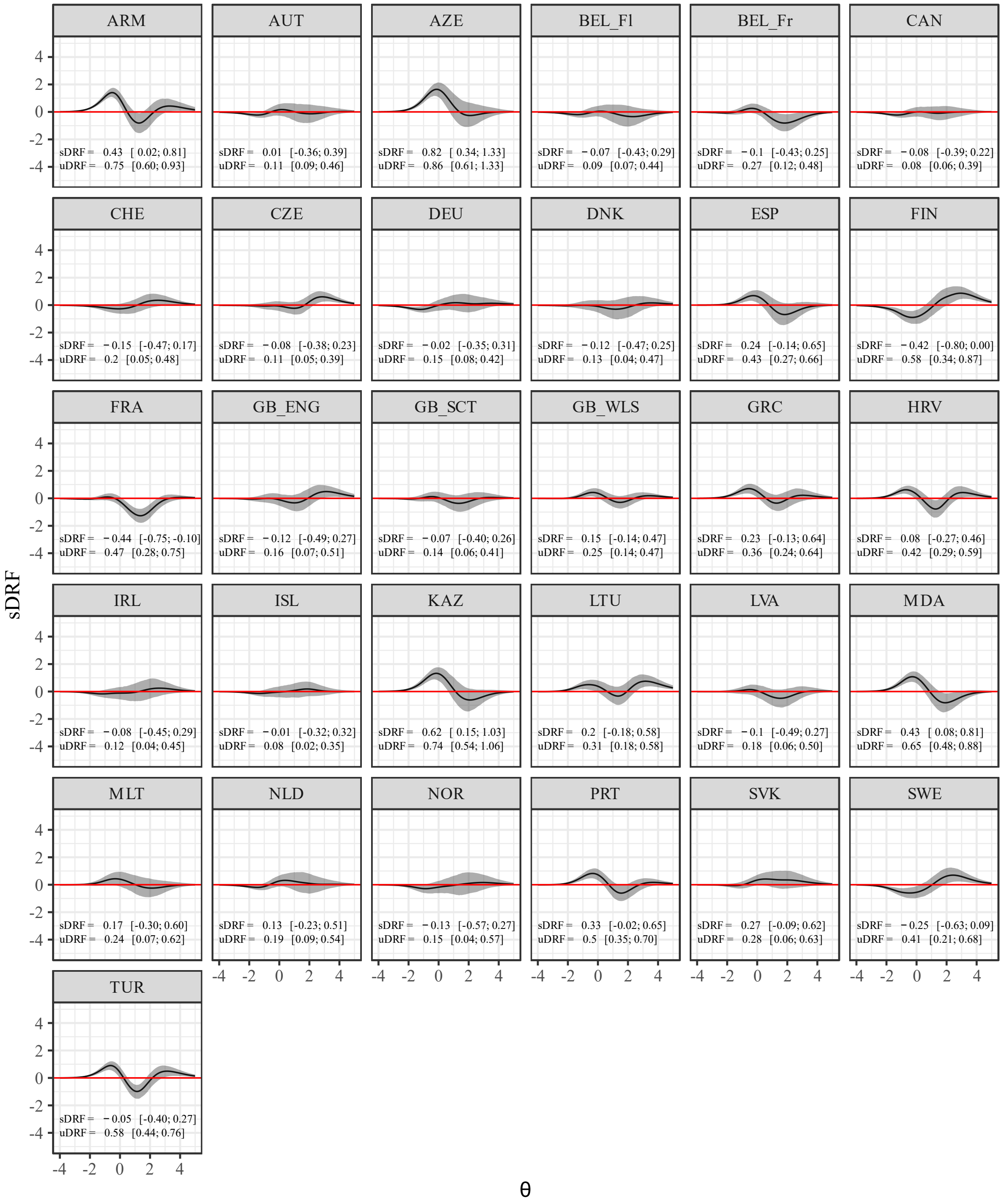

Notes. The curves show differences in expected test scores (with 99% CI) dependent on the level of the latent variable (ISR as reference group), *sDRF* = compensatory differential response functioning statistic with 99% CI, *uDRF* = non-compensatory differential response functioning statistic with 99% CI.

Figure A85. Differential test functioning: Reference group: KAZ.

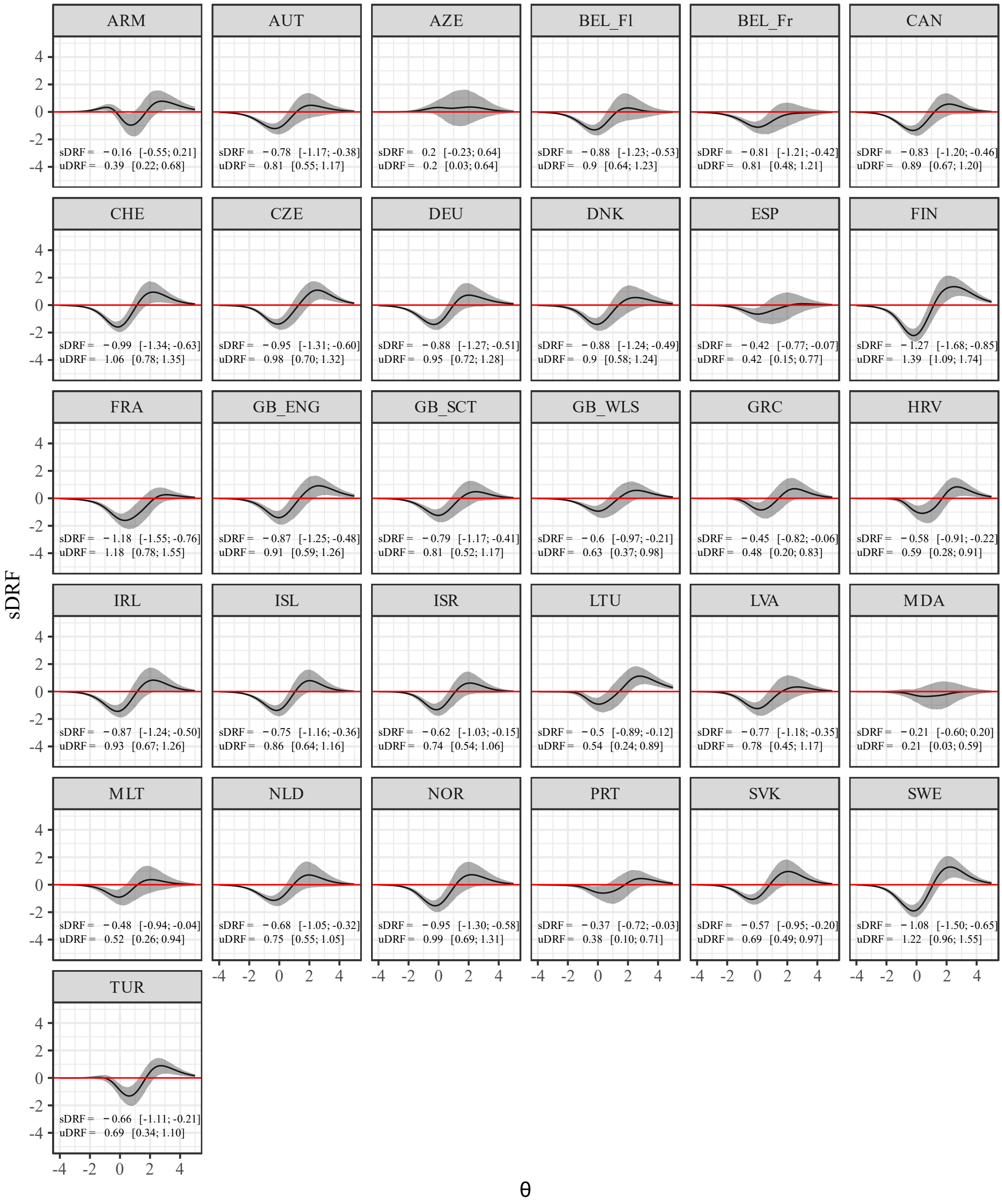

Notes. The curves show differences in expected test scores (with 99% CI) dependent on the level of the latent variable (KAZ as reference group), *sDRF* = compensatory differential response functioning statistic with 99% CI, *uDRF* = non-compensatory differential response functioning statistic with 99% CI.

Figure A86. Differential test functioning: Reference group: LTU.

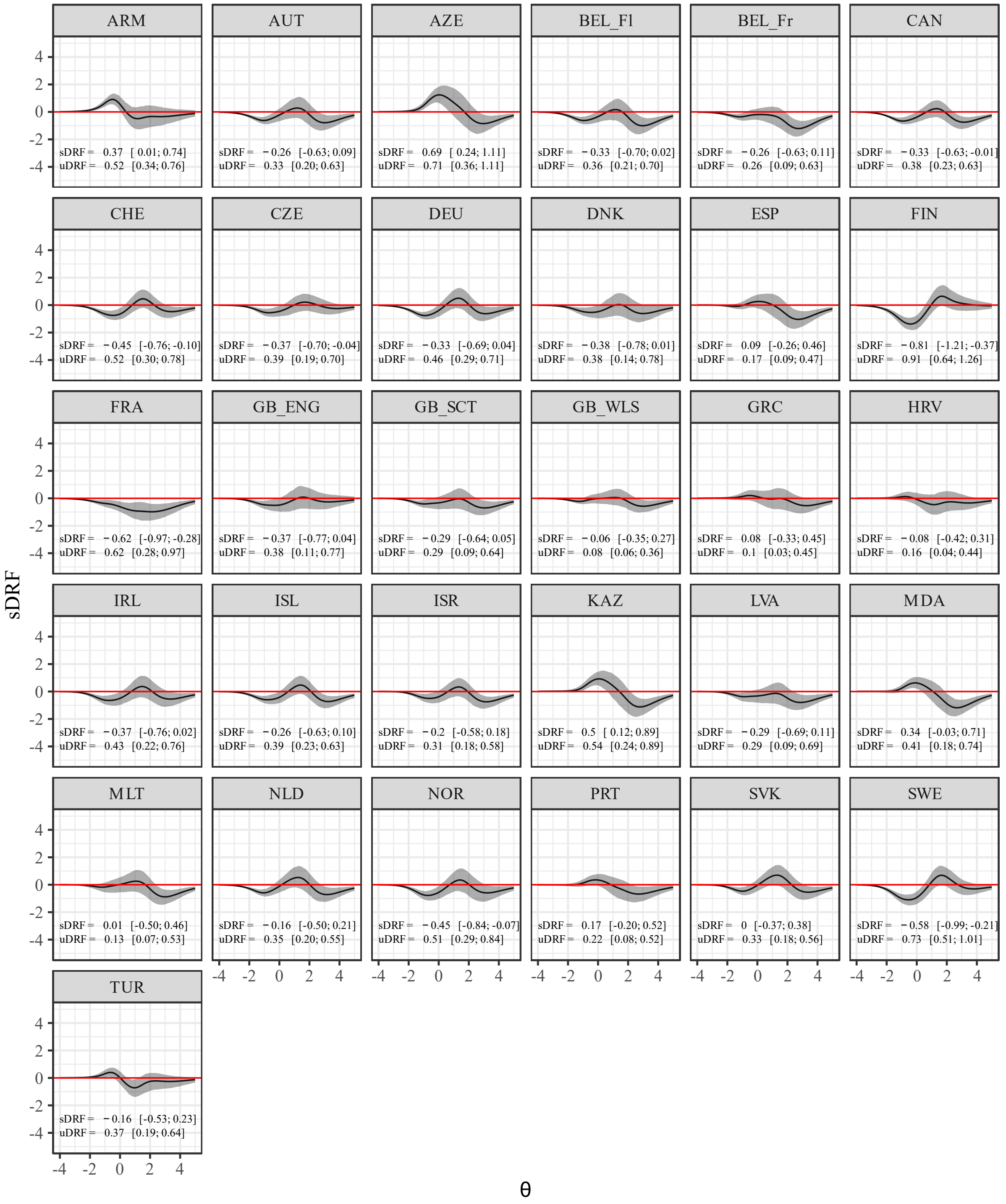

Notes. The curves show differences in expected test scores (with 99% CI) dependent on the level of the latent variable (LTU as reference group), *sDRF* = compensatory differential response functioning statistic with 99% CI, *uDRF* = non-compensatory differential response functioning statistic with 99% CI.

Figure A87. Differential test functioning: Reference group: LVA.

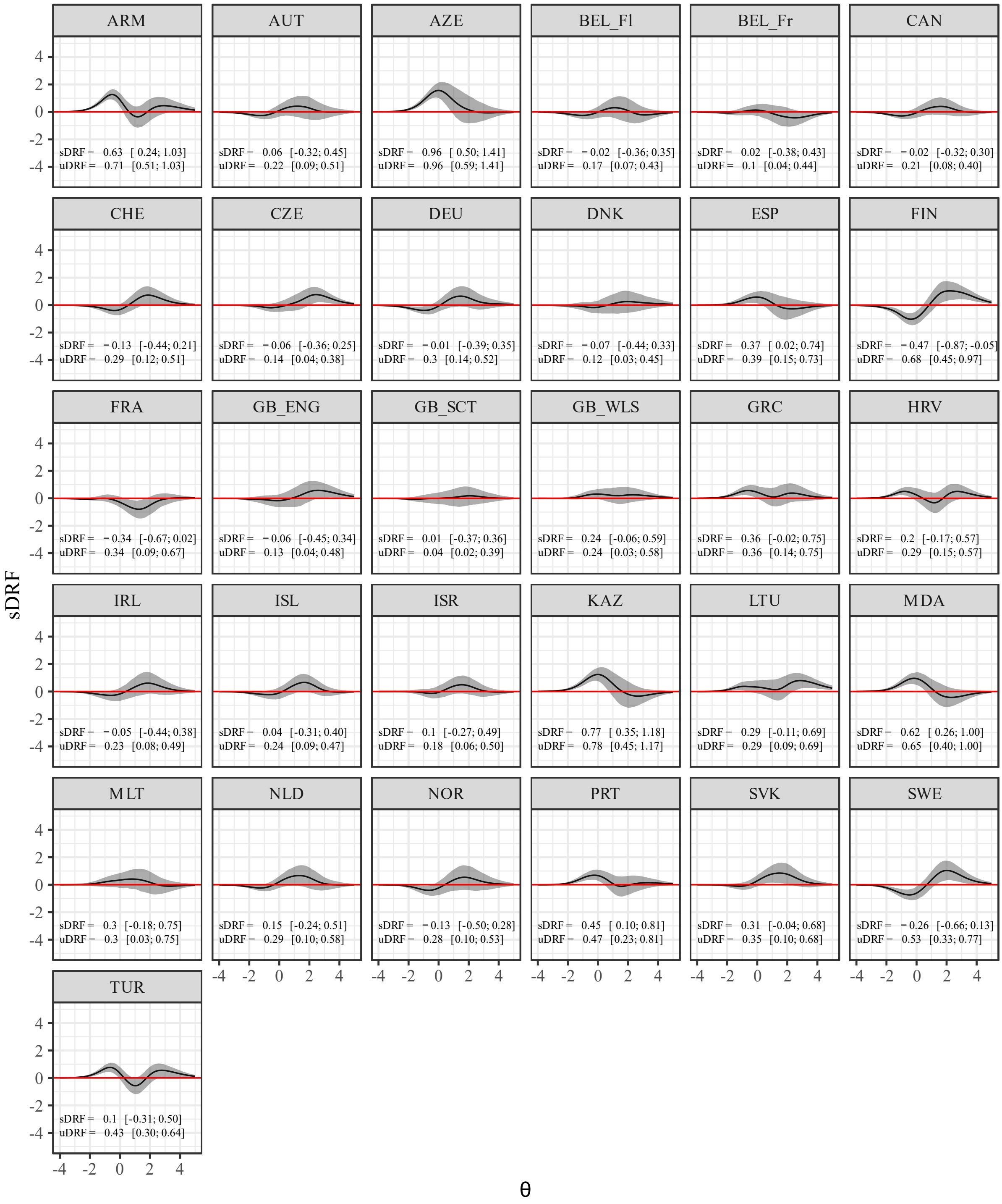

Notes. The curves show differences in expected test scores (with 99% CI) dependent on the level of the latent variable (LVA as reference group), *sDRF* = compensatory differential response functioning statistic with 99% CI, *uDRF* = non-compensatory differential response functioning statistic with 99% CI.

Figure A88. Differential test functioning: Reference group: MDA.

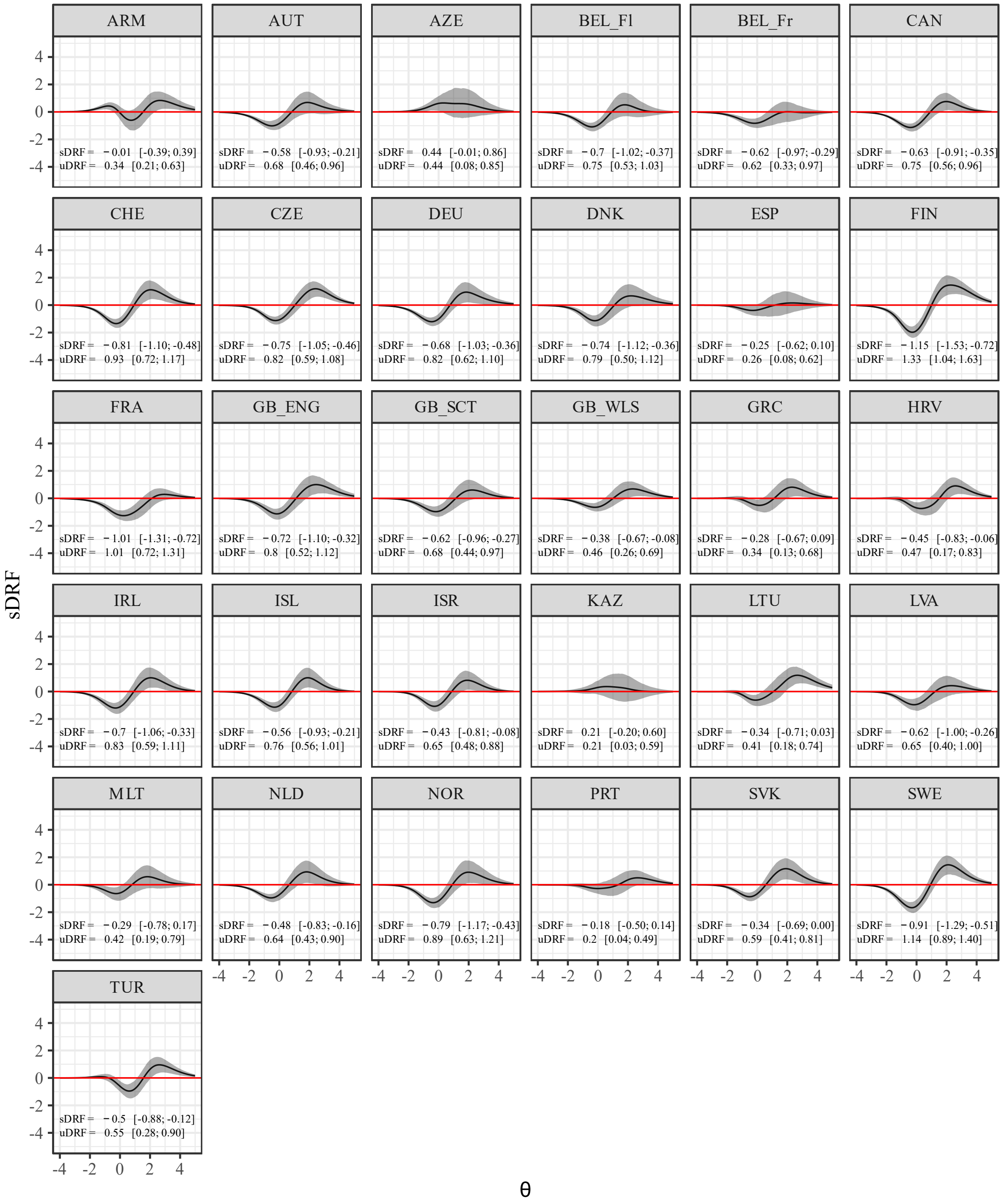

Notes. The curves show differences in expected test scores (with 99% CI) dependent on the level of the latent variable (MDA as reference group), *sDRF* = compensatory differential response functioning statistic with 99% CI, *uDRF* = non-compensatory differential response functioning statistic with 99% CI.

Figure A89. Differential test functioning: Reference group: MLT.

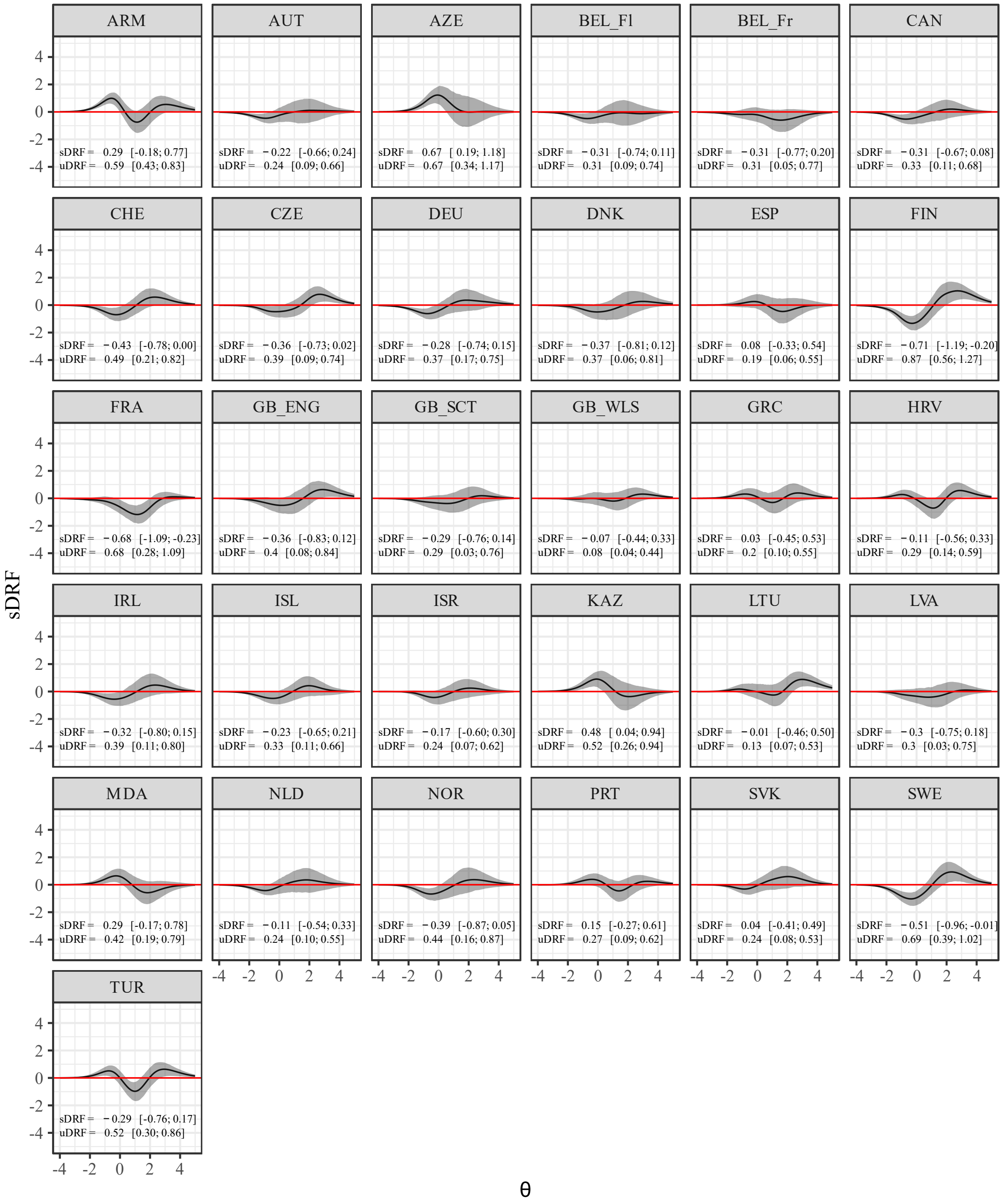

Notes. The curves show differences in expected test scores (with 99% CI) dependent on the level of the latent variable (MLT as reference group), *sDRF* = compensatory differential response functioning statistic with 99% CI, *uDRF* = non-compensatory differential response functioning statistic with 99% CI.

Figure A90. Differential test functioning: Reference group: NLD.

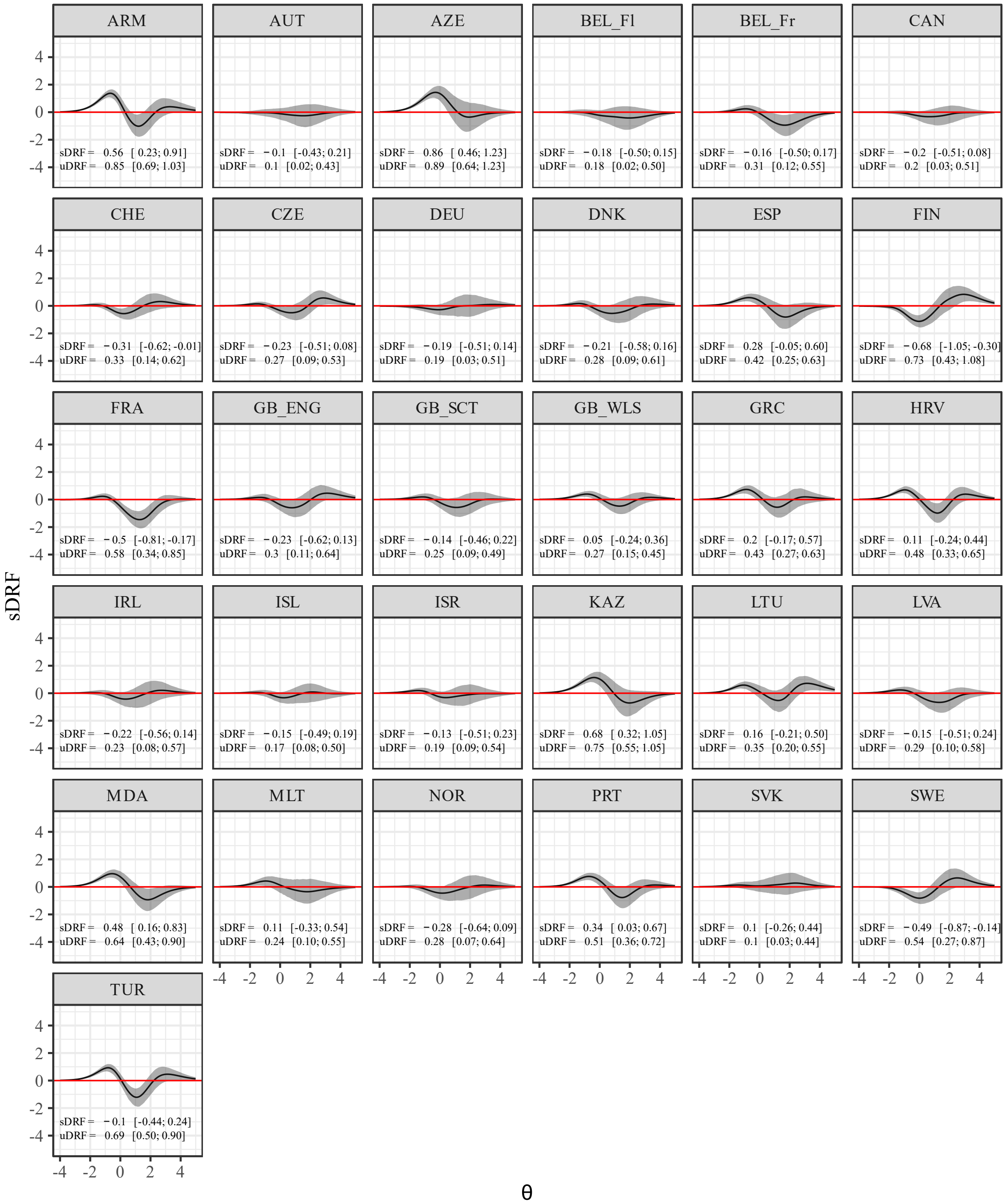

Notes. The curves show differences in expected test scores (with 99% CI) dependent on the level of the latent variable (NLD as reference group), *sDRF* = compensatory differential response functioning statistic with 99% CI, *uDRF* = non-compensatory differential response functioning statistic with 99% CI.

Figure A91. Differential test functioning: Reference group: NOR.

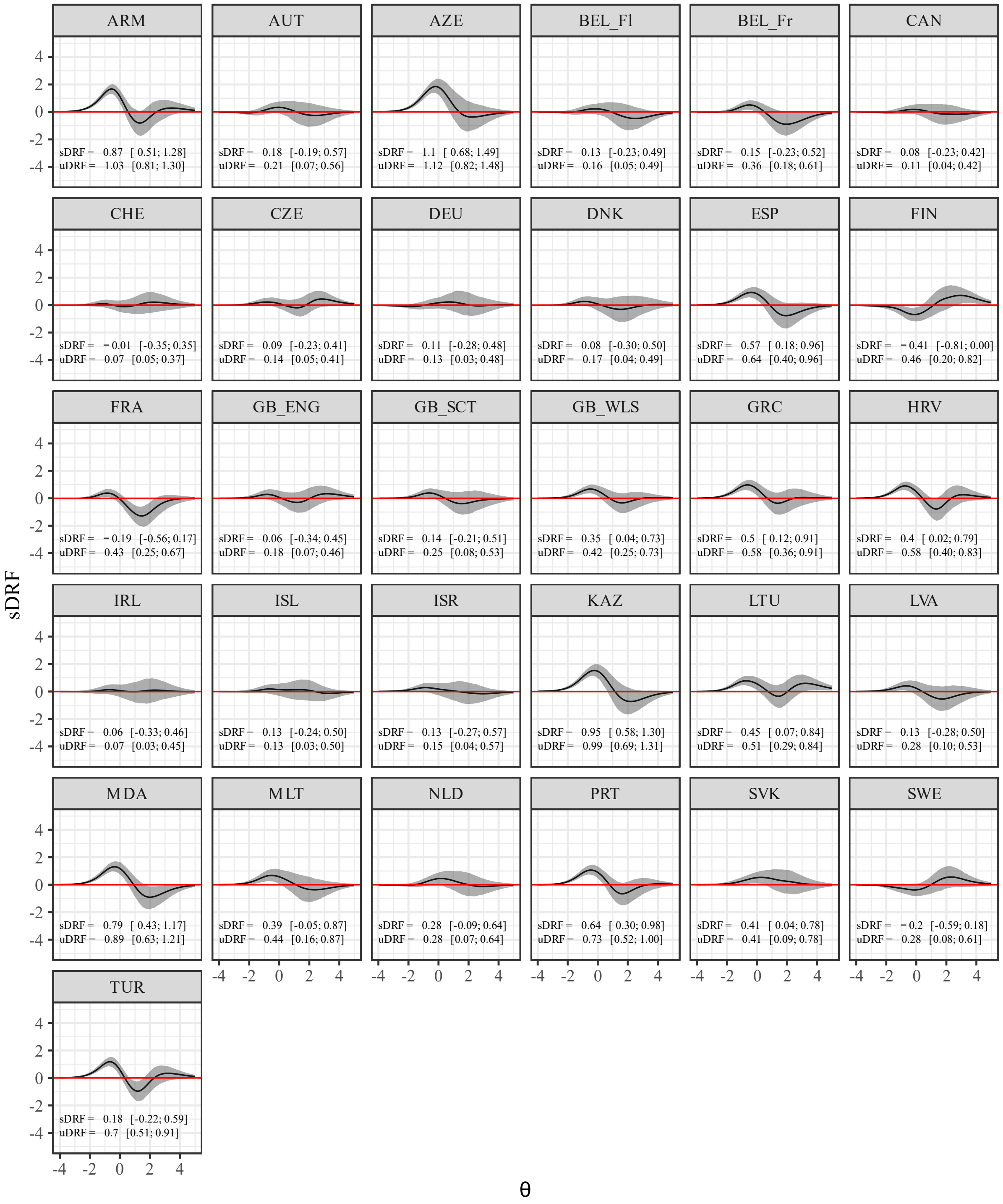

Notes. The curves show differences in expected test scores (with 99% CI) dependent on the level of the latent variable (NOR as reference group), *sDRF* = compensatory differential response functioning statistic with 99% CI, *uDRF* = non-compensatory differential response functioning statistic with 99% CI.

Figure A92. Differential test functioning: Reference group: PRT.

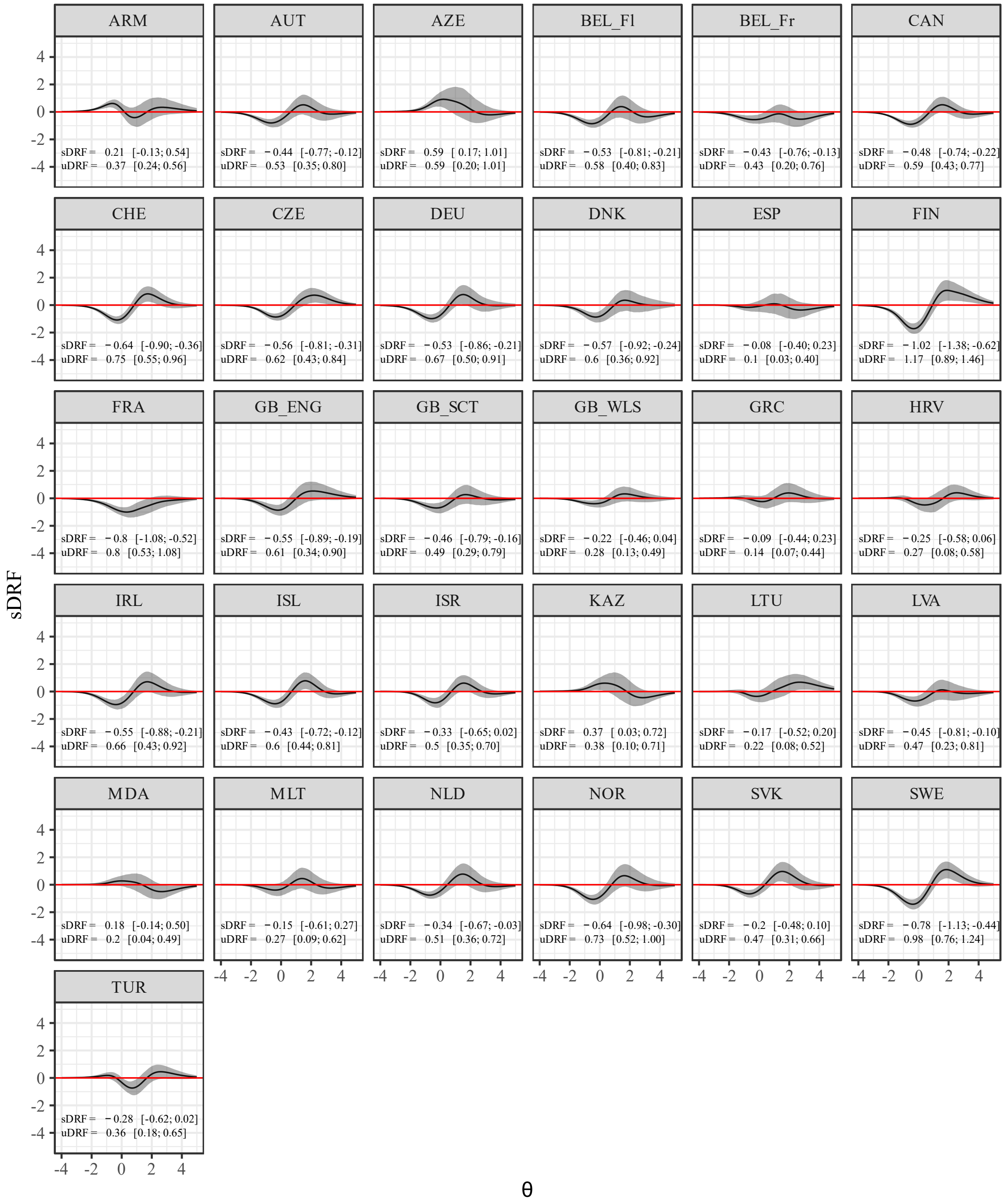

Notes. The curves show differences in expected test scores (with 99% CI) dependent on the level of the latent variable (PRT as reference group), *sDRF* = compensatory differential response functioning statistic with 99% CI, *uDRF* = non-compensatory differential response functioning statistic with 99% CI.

Figure A93. Differential test functioning: Reference group: SVK.

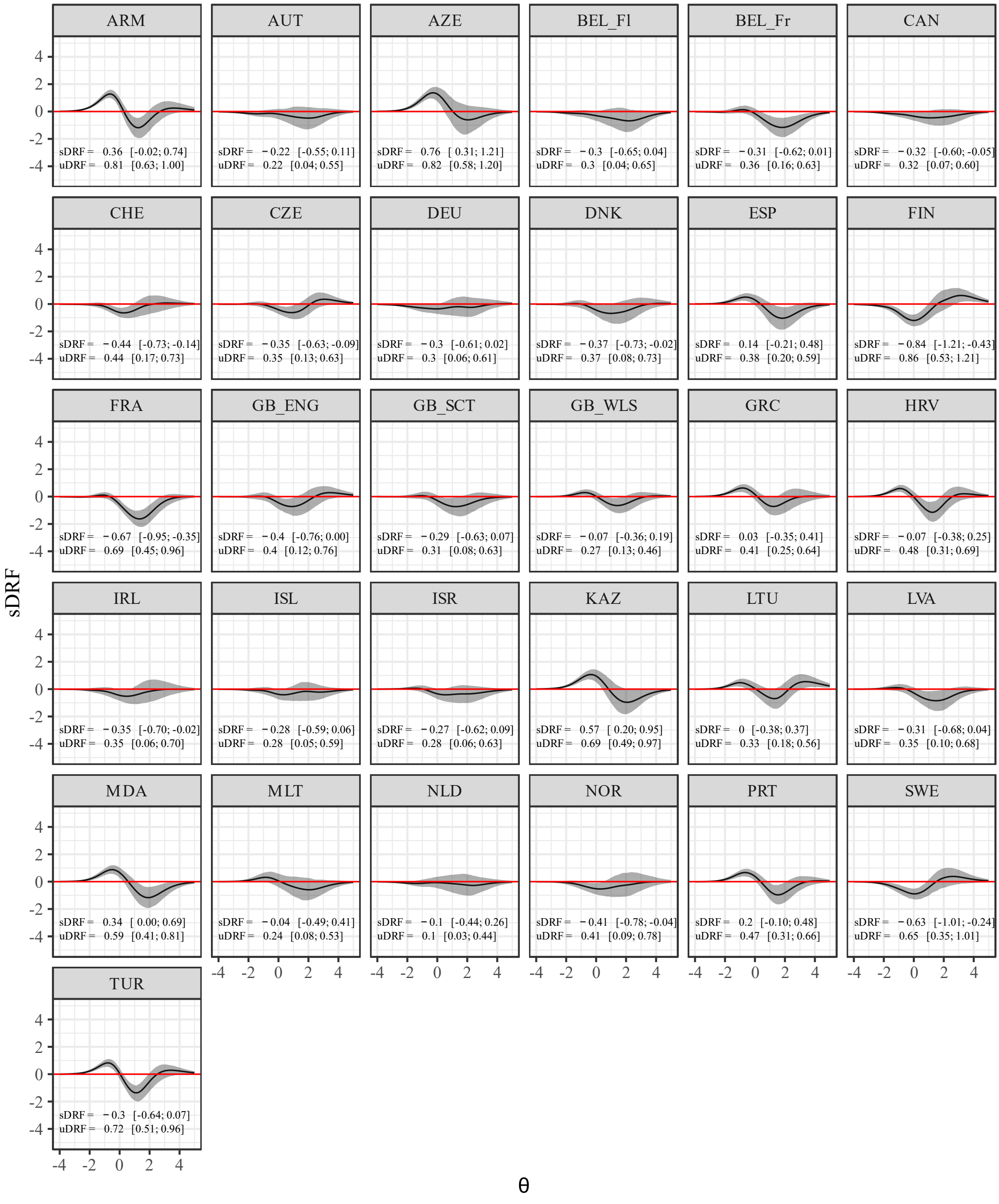

Notes. The curves show differences in expected test scores (with 99% CI) dependent on the level of the latent variable (SVK as reference group), *sDRF* = compensatory differential response functioning statistic with 99% CI, *uDRF* = non-compensatory differential response functioning statistic with 99% CI.

Figure A94. Differential test functioning: Reference group: SWE.

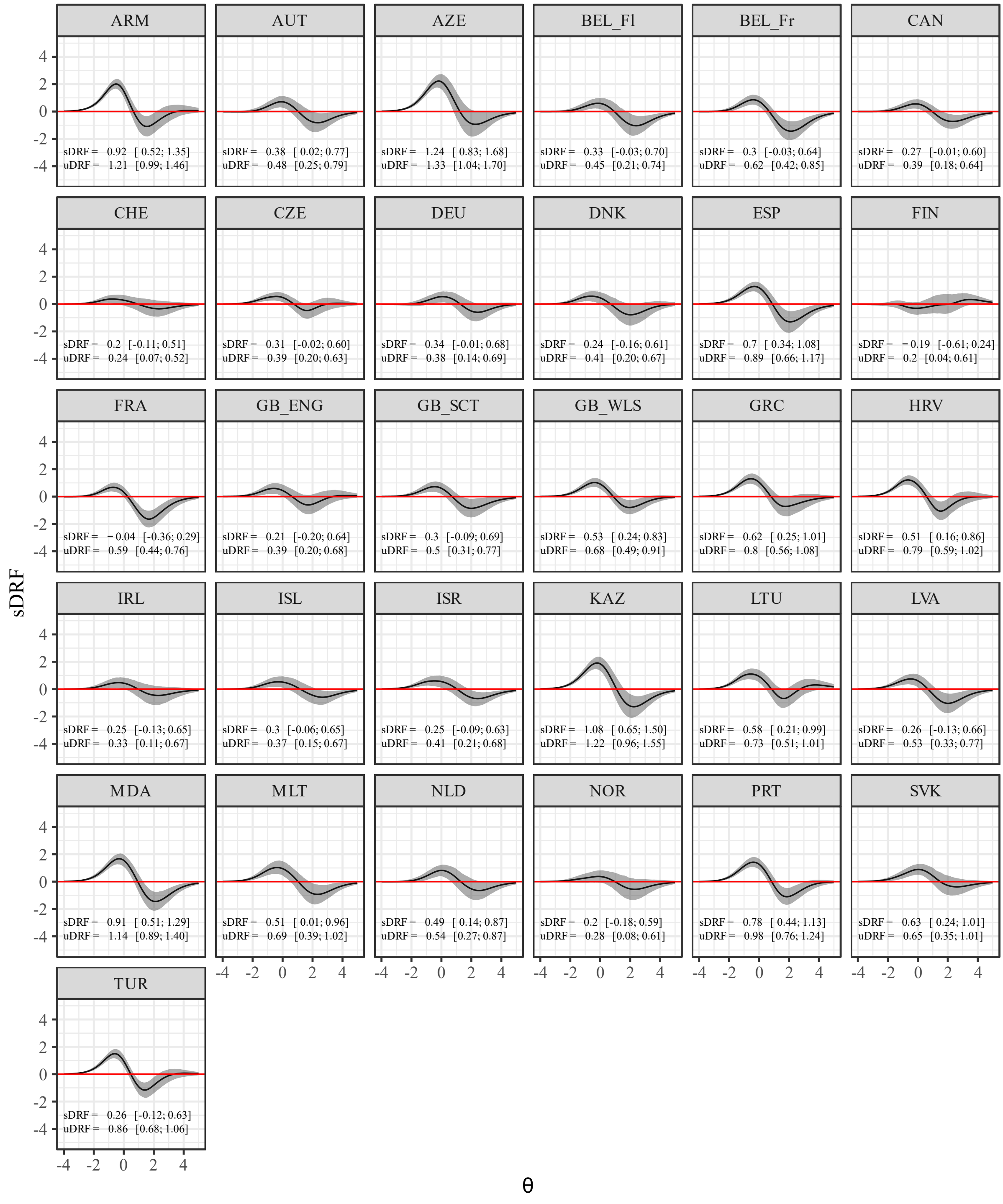

Notes. The curves show differences in expected test scores (with 99% CI) dependent on the level of the latent variable (SWE as reference group), *sDRF* = compensatory differential response functioning statistic with 99% CI, *uDRF* = non-compensatory differential response functioning statistic with 99% CI.

Figure A95. Differential test functioning: Reference group: TUR.

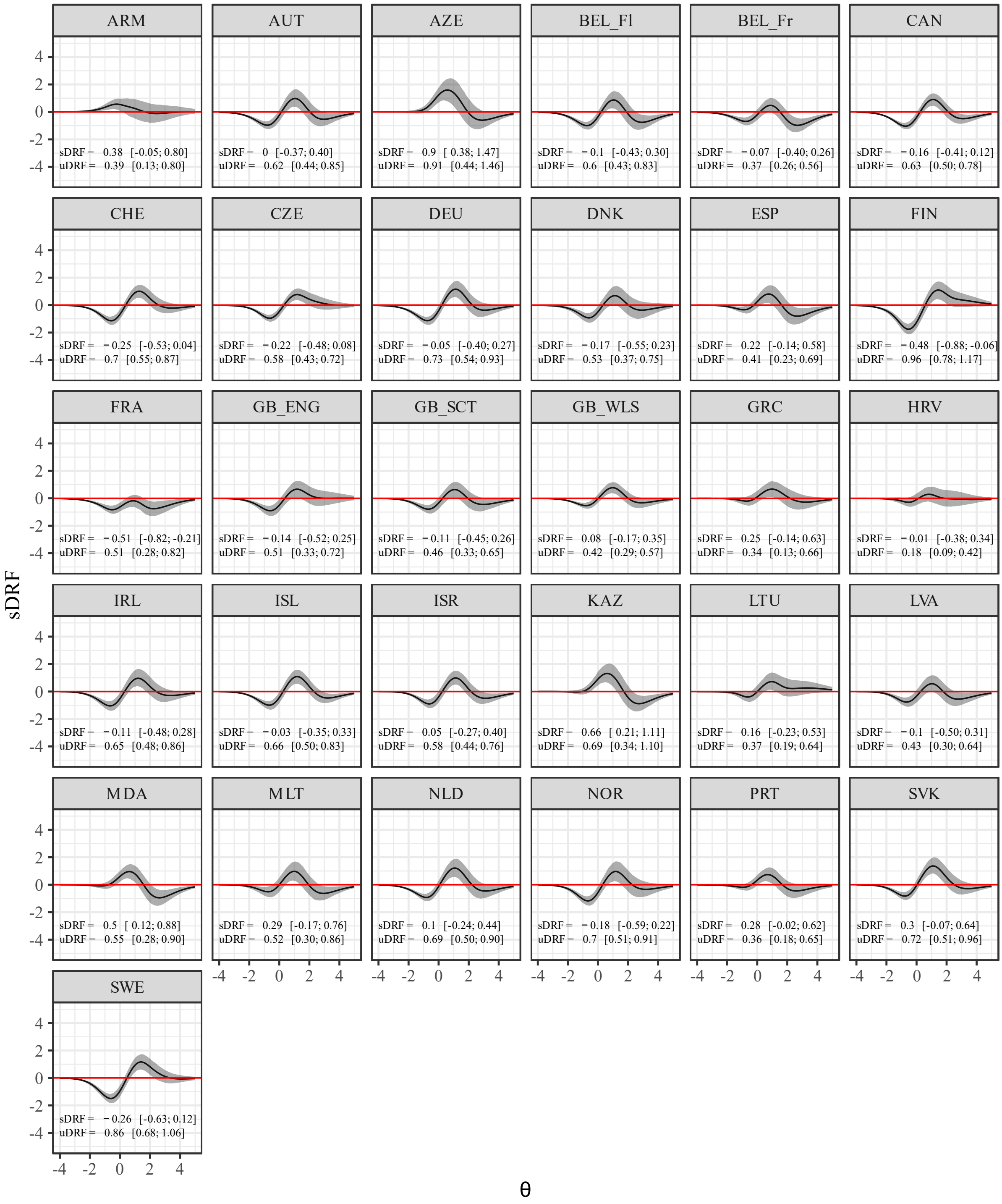

Notes. The curves show differences in expected test scores (with 99% CI) dependent on the level of the latent variable (TUR as reference group), *sDRF* = compensatory differential response functioning statistic with 99% CI, *uDRF* = non-compensatory differential response functioning statistic with 99% CI.

Figure A96. Heatmap of *sDRF* and *uDRF*.

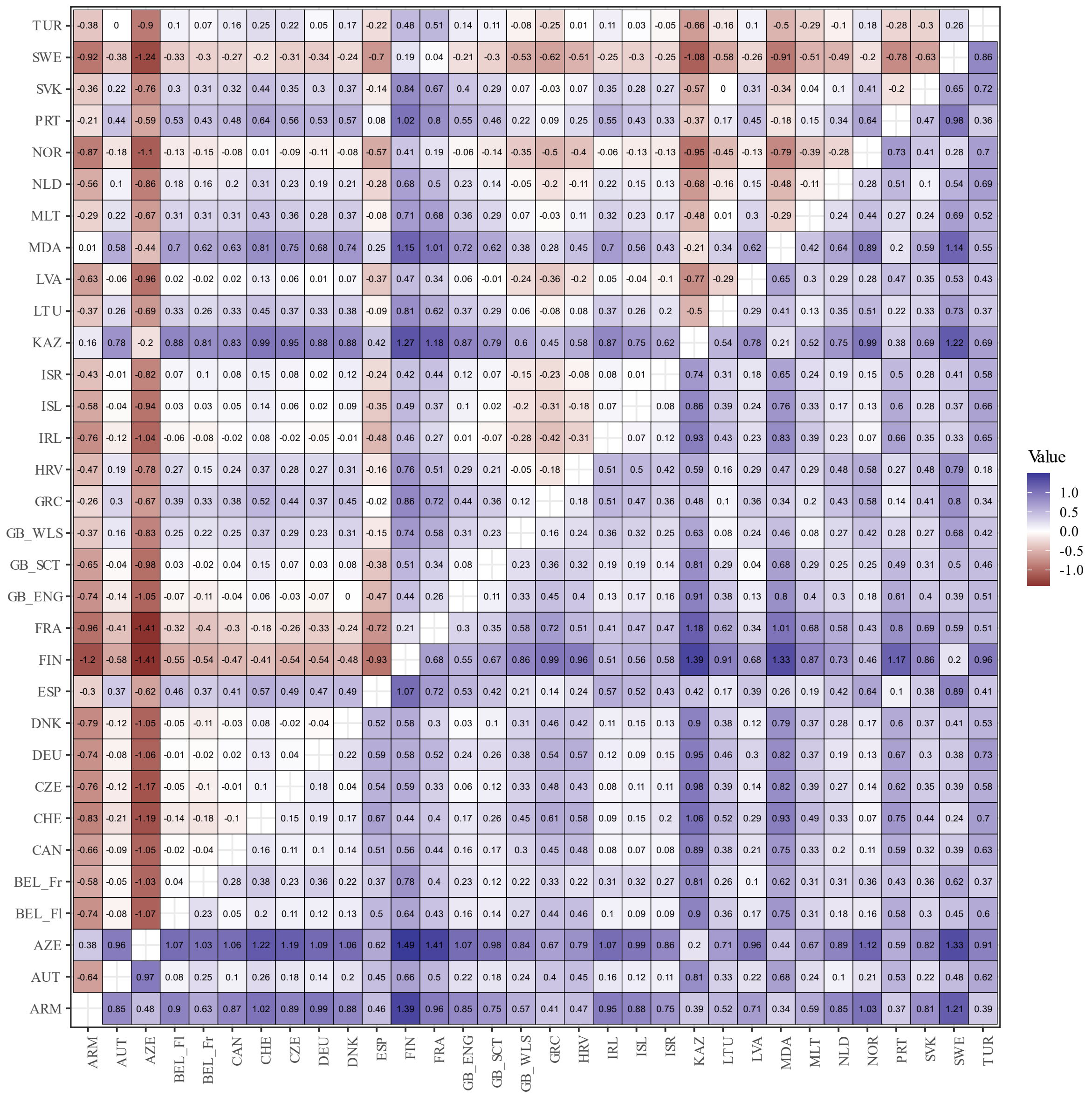

Notes. *sDRF* = compensatory differential response functioning statistic with 99% CI, *uDRF* = non-compensatory differential response functioning statistic with 99% CI. *sDRF* coefficients are above the diagonal, *uDRF* coefficients are below the diagonal.

Figure A97. Scatterplot with factor scores and manifest sum scores.

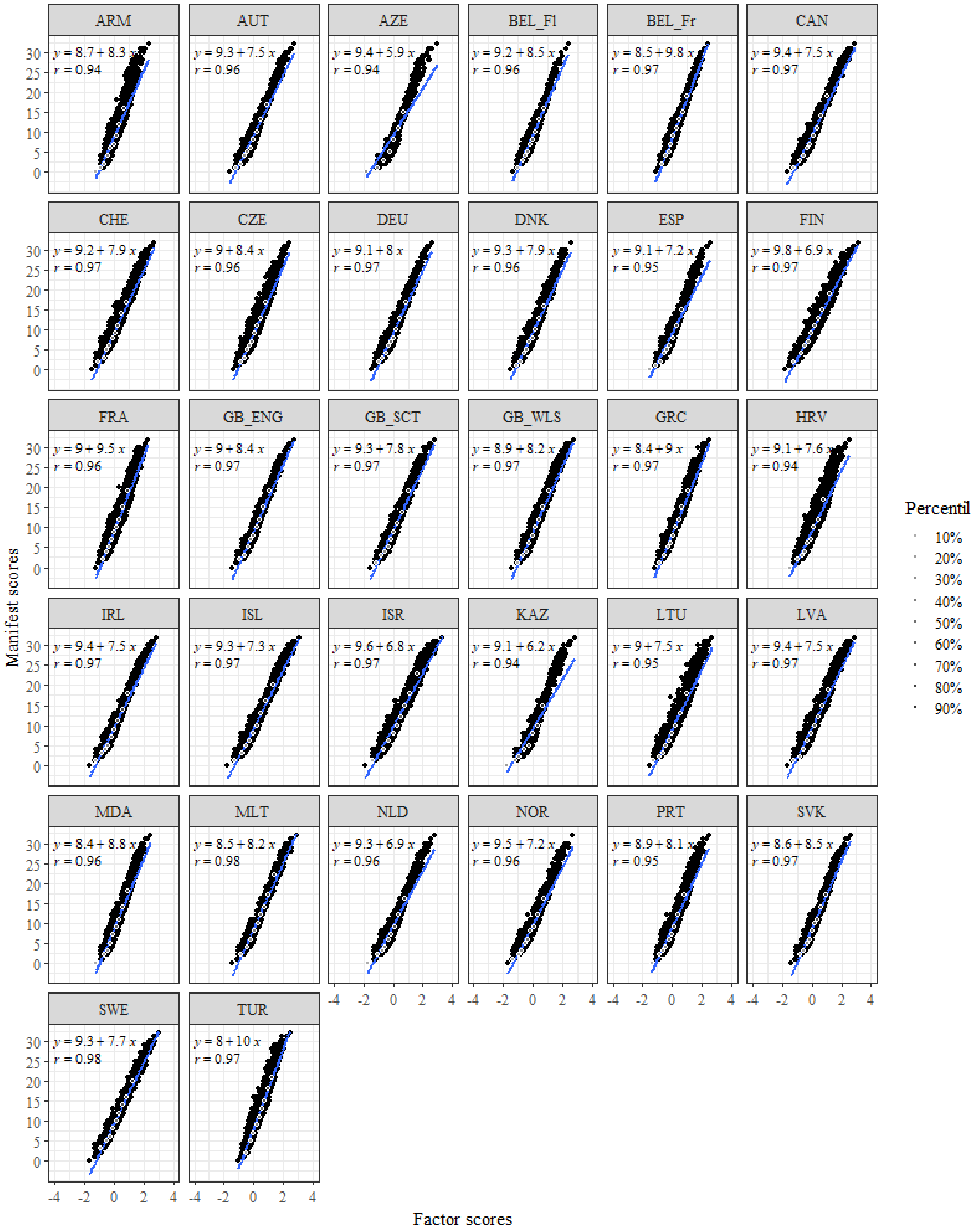

Notes. Factor scores were estimates via expected a-posterior (EAP) method. The regression equation and correlation coefficient are shown.

Figure A98. Means of manifest sum scores and factor scores.

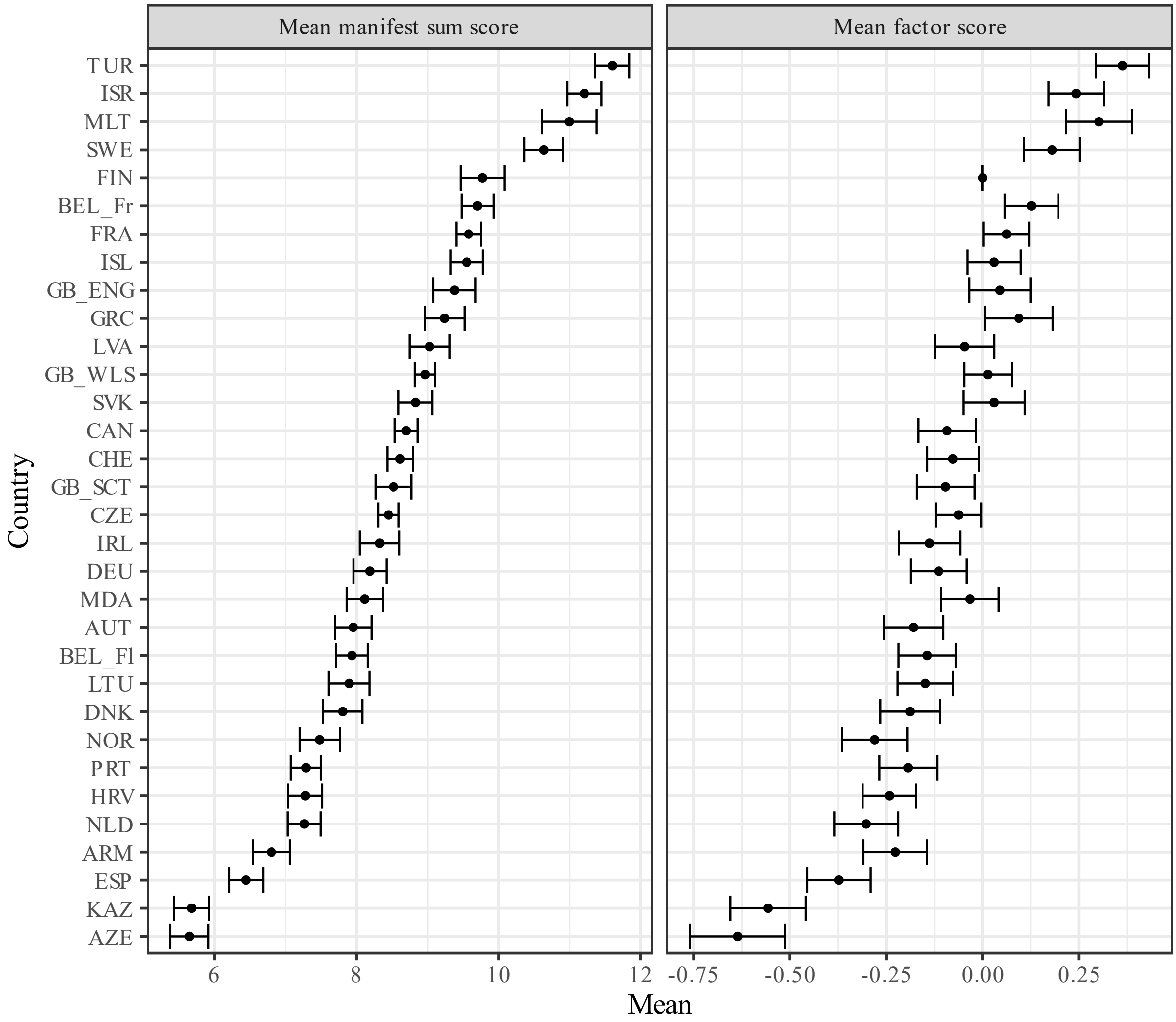

Notes. Means of factor scores and manifest sum scores with 99% CI. Factor scores were estimated via expected a-posterior (EAP) method.

Figure A99. Factor score distribution.

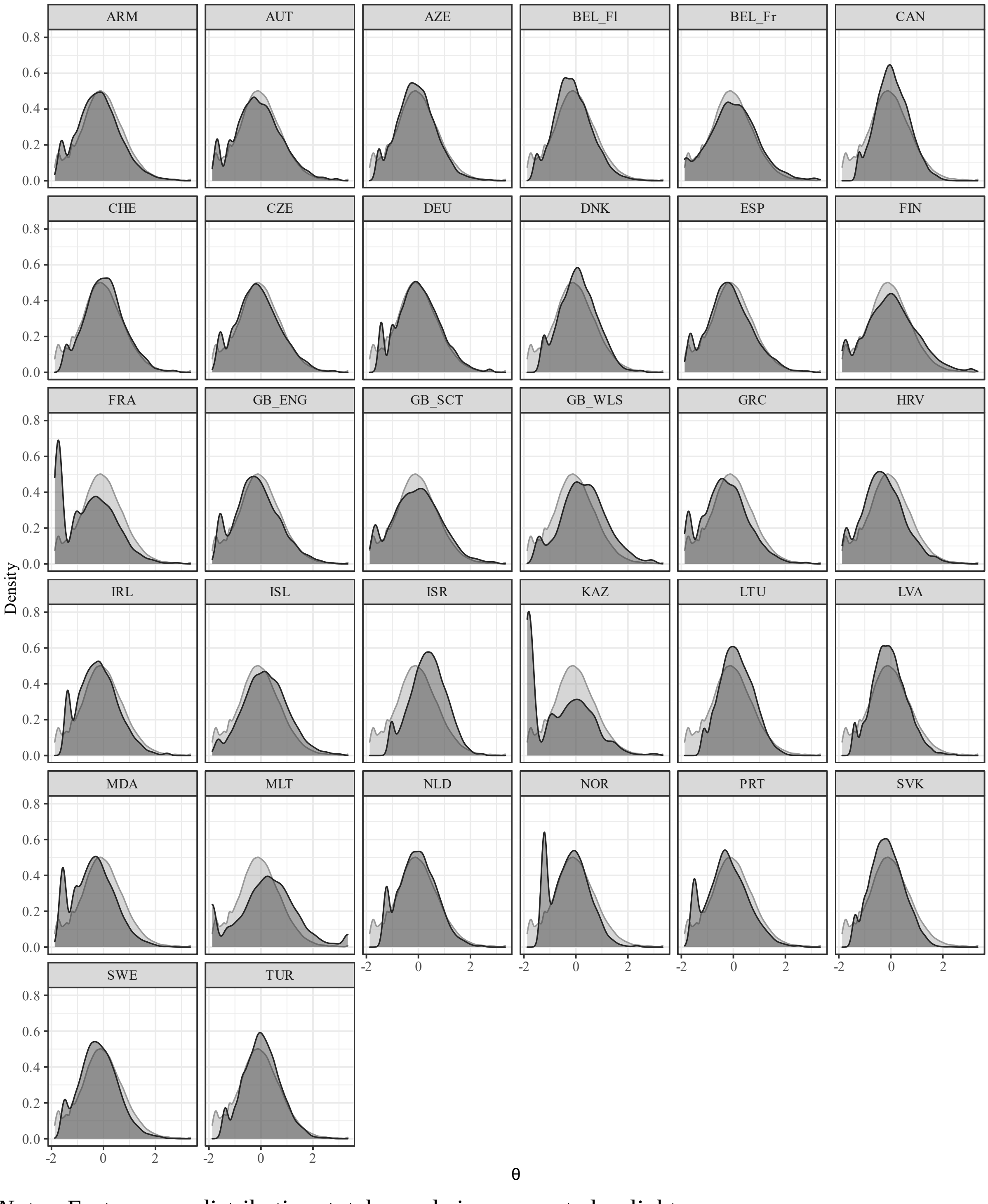

Notes. Factor score distribution; total sample is represented as light gray area.

Figure A100. Item and test information functions of the alignment model.

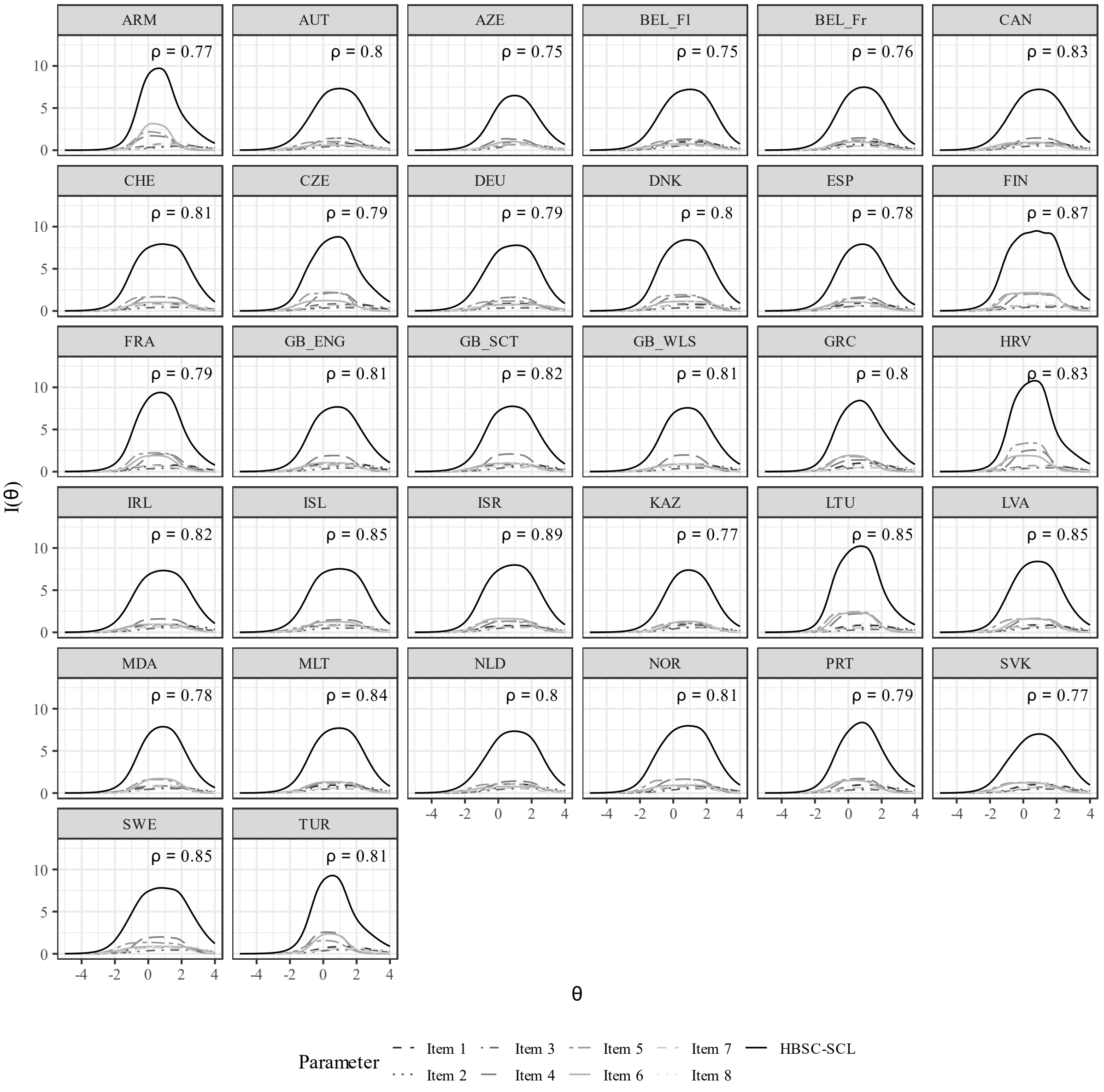

Notes. ρ represents the empirical marginal reliability. Items (1) headache, (2) stomachache, (3) backache, (4) feeling low, (5) irritability/bad temper, (6) feeling nervous, (7) difficulties in getting to sleep, (8) feeling dizzy.
